# Late-Stage Functionalization via Thianthrenation: A Reaction Enabling Rapid Preparation of Drug Beads for Chemical Pulldown

**DOI:** 10.1021/acs.jmedchem.6c01571

**Published:** 2026-07-21

**Authors:** Robert J. Smith, Laurine Brouck, Chloe Pelletier, Sandra Carvalho, Gustavo Bravo Ruiz, Victoriano Corpas-Lopez, Gourav Dey, Stephen Patterson, Susan Wyllie

**Affiliations:** Drug Discovery Unit, Division of Drug Discovery, Faculty of Life Sciences, 98264 University of Dundee, Dow Street, Dundee DD1 5EH, United Kingdom

## Abstract

A major bottleneck in identifying the molecular targets of phenotypically active compounds via affinity pulldown is the chemical synthesis of functionalized probes suitable for attachment to solid supports. This process often requires time-intensive, multistep synthesis. A more direct approach would be to use late-stage functionalization chemistry to directly modify the phenotypic hit/lead. We report a proof-of-concept workflow adapting the thianthrenation late-stage functionalization reaction to expedite the preparation of probes. A high-throughput experimental approach allowed rapid assessment of substrate suitability for this chemical reaction. Twenty-four compounds with activity against the protozoan parasite *Leishmania donovani* were progressed through this workflow. Five compounds were identified as thianthrenation substrates, and four were functionalized to prepare affinity probes. Compound **9b** was advanced to target identification in *L. donovani*, with molecular targets identified with high confidence. These studies highlight the utility of late-stage functionalization reactions to expedite the synthesis of functionalized tool compounds for multiple downstream applications.

## Graphical abstract

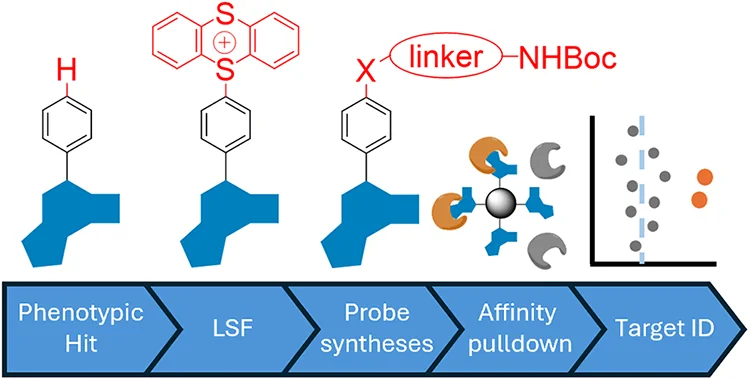

## Introduction

Neglected tropical diseases, such as Chagas disease, the leishmaniases and human African trypanosomiasis, inflict a major health burden on low- and middle-income countries throughout the world.^1^ There are few efficacious, affordable treatments available for these diseases^1^ and emerging drug resistance is a constant threat.^2,3^ To compound these issues, there is a severe lack of robustly validated drug targets, hindering the development of new therapeutics.^1^ The discovery of new start points to initiate drug discovery for these parasitic diseases has predominantly relied upon phenotypic screening,^4^ leading to the identification of hit compounds with little supporting information regarding mode of action and/or molecular target.^5^ Knowledge of a hit compound’s molecular target(s) is deemed as highly advantageous since subsequent development and optimization of the compound series can be supported by target structure information, enabling structure-based rational design. In addition, knowledge of the molecular target can facilitate drug discovery portfolio management, allowing prioritisation of chemical series with overlapping modes of action and early termination of series with undevelopable or undesirable modes of action.^6^

We have successfully employed multiple genetic and chemoproteomic approaches to identify the molecular targets of antiparasitic compounds.^7–11^ An important component of our drug target deconvolution toolkit is affinity-based chemical pulldown which can provide direct evidence of drug-target interaction, as well as an indication of target binding affinity.^8,12,13^ One of the major bottlenecks encountered in chemical pulldown is the requirement to develop bivalent chemical probes via attachment of a spacer (e.g., polyethylene glycol (PEG) chain) to the parent compound and a functional handle to attach to a solid support (e.g., agarose, Sepharose).^13^ This is not a trivial process since relatively few phenotypic hits possess a suitably positioned, orthogonally reactive functional group to enable attachment of a chemical spacer. The alternative strategy is to develop a functionalized probe analogue via bespoke synthesis, often requiring multiple synthetic steps. For example, a previous pulldown study aimed at identifying the molecular target of a promising antileishmanial required two synthetic sequences of 8–9 steps to produce three functionalized probes.^12^ Synthesis of each probe took >5 days to complete. Thus, including time for probe design, troubleshooting, and optimization, each synthetic sequence can take ≥ 1 month from conception to completion.

Late-stage C–H functionalization (LSF) chemistry has advanced significantly over the past two decades and the late-stage diversification of complex molecular scaffolds is now feasible.^14,15^ More recently, LSF chemistry has been increasingly employed by the medicinal chemistry field, allowing rapid structure–activity relationship (SAR) expansion and modulation of drug properties, without the need for *de novo* synthesis.^16,17^ One such LSF is the thianthrenation reaction (Figure 1).^18,19^ The reaction is a highly *par*-selective C–H functionalization of an aromatic ring, it requires moderately electron-rich aromatic rings and is compatible with a wide range of functional groups, including unprotected amines, alcohols and carboxylic acids. Indeed, compounds with multiple, complex functionalities tolerate this reaction well, including natural products with one or more stereo centers and drug-like molecules.^18^

**Figure 1:**
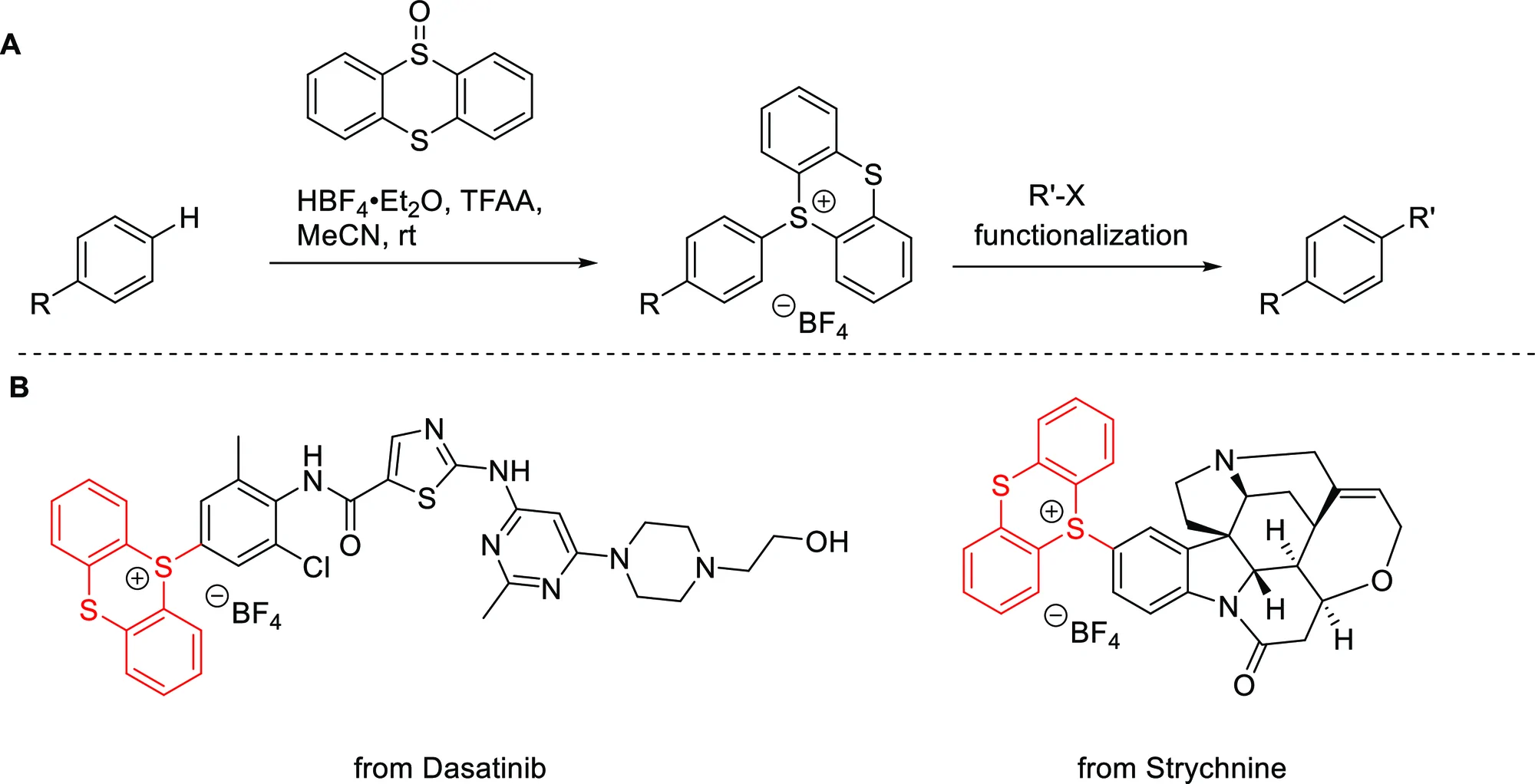
Thianthrenation late-stage functionalization. (A) General overview of thianthrenation and functionalization strategy. (B) Selected literature substrates, thianthrene group shown in red.

Aryl-thianthrene salts produced in this reaction are useful synthetic lynchpins that can be exploited in over a dozen subsequent chemical transformations, including most modern cross-coupling reactions such as the Heck, Negishi, Suzuki and Sonogashira reactions.^18,20–23^ This transformation is particularly powerful when you consider that aromatic rings are ubiquitous in drug discovery, with 76% of marketed drugs containing at least one aromatic ring.^17,24,25^ Additionally, a survey of the published outputs of the medicinal chemistry programs of 3 major pharmaceutical companies revealed that of >3500 compounds, 99% had at least one aromatic ring, and 94% had at least one benzene ring.^26^

To the best of our knowledge, there has been only one reported use of thianthrenation to facilitate probe preparation for chemical biology applications.^27^ Thus, late-stage functionalization is underutilized in chemical biology. Well validated methods allowing the rapid preparation of bifunctional probes directly from the parent compound without the need for laborious and expensive *de novo* synthesis would be of significant benefit. Here, we report our adaptation of the thianthrenation reaction to create a high-throughput platform aimed at rapidly assessing the tractability of compounds toward this reaction. Twenty-four compounds with confirmed antileishmanial activity *in vitro* were assessed through this platform and five scaffolds were deemed suitable for thianthrenation. PEG-linker analogues were synthesized and their phenotypic activities assessed. Following linker attachment via thianthrenation, five analogues were found to retain antileishmanial activity relative to their corresponding parent compounds and compound **9b** was progressed to target identification studies through chemical pulldown. This proof-of-concept workflow highlights the utility of late-stage functionalization to enable downstream applications requiring parent compound modification.

## Results and Discussion

### Diversification of Thianthrene Salts with PEG Linkers

To design an effective probe for affinity pulldown, a solvent-exposed vector suitable for attachment to an affinity matrix must be identified. It is vital that attachment of a PEG linker at the selected position does not severely impact the phenotypic activity of the probe relative to the parent. In the absence of an identified molecular target, as is the case for hits derived through phenotypic screening, probe design cannot be structure guided and is essentially blinded. To overcome this lack of structural information, PEG chains can be attached to a range of different positions and vectors on the active molecule, using structure–activity relationship data to identify a suitable location. Phenotypic activity of the resulting probe is determined, compared to that of the parent compound and used as a surrogate to confirm target engagement.

To incorporate thianthrenation LSF into a workflow suitable for high throughput assessment of compounds, it was first necessary to confirm that functionalized PEG linkers could be competent substrates in the cross-coupling reactions with aryl-thianthrene salts. Pyriproxyphen was selected to provide proof-of-concept, since thianthrenation of this compound has been extensively studied and the corresponding aryl-thianthrene salts are compatible with a wide-range of cross-coupling reactions.^18,19,22,23^ Five cross-coupling reactions were evaluated (Table 1) and a range of suitable *N*-Boc protected PEG compounds were selected as the cross-coupling partners.

**Table 1:**
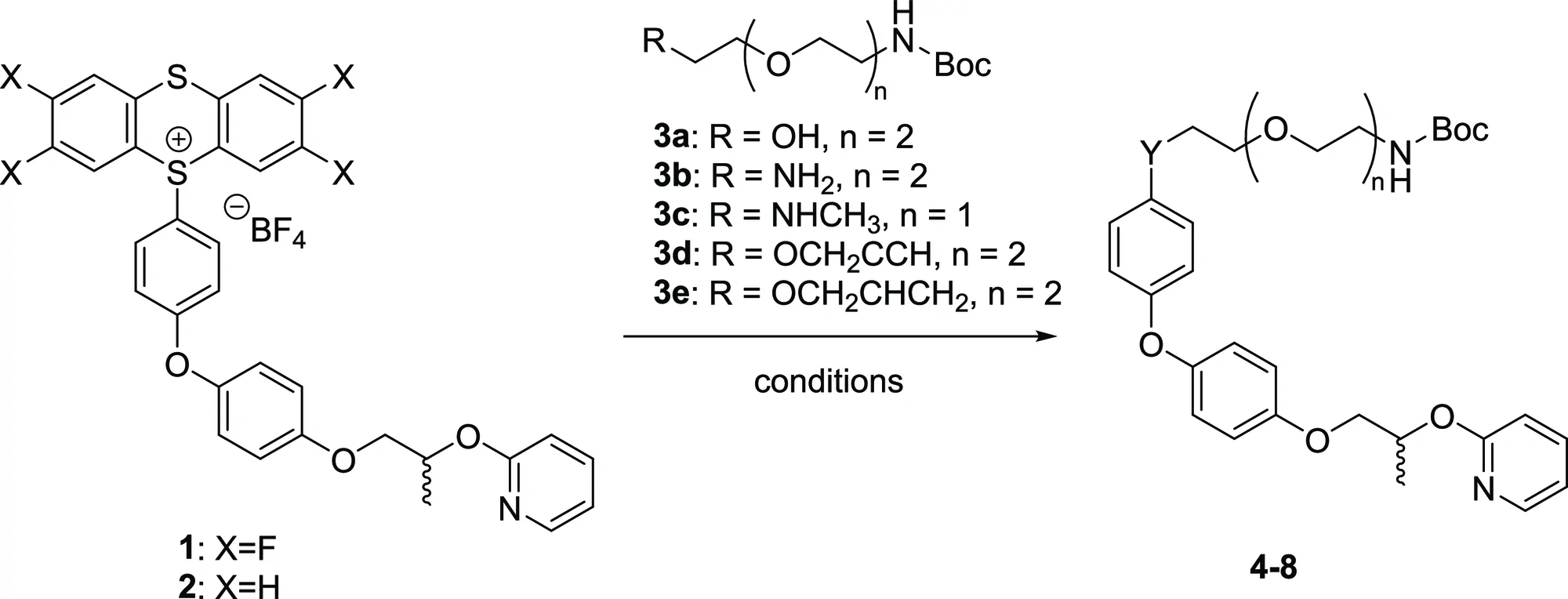

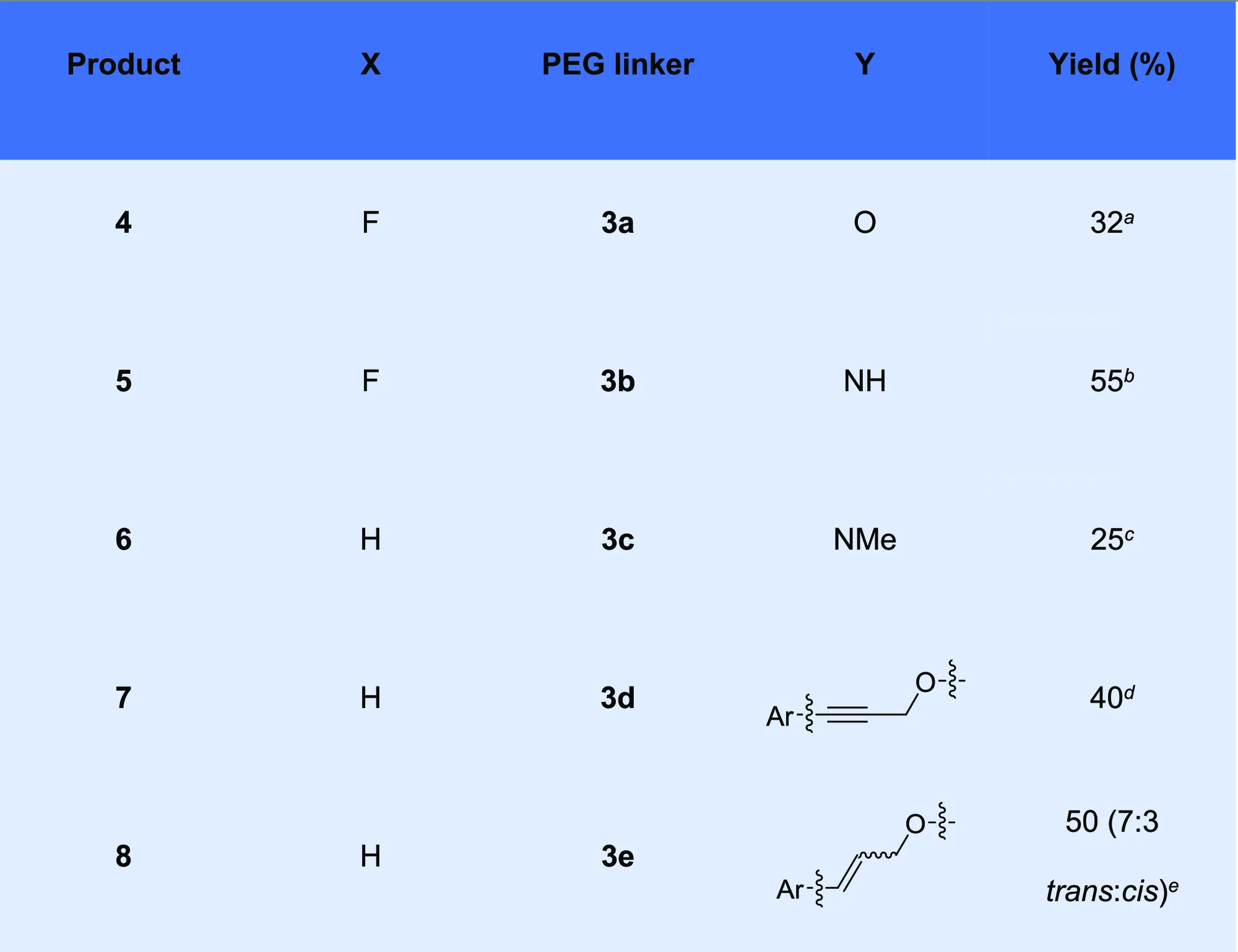
Pyriproxyphen Thianthrene Salt Cross Coupling with PEG Linkers

a [Ir­(dF­(CF_3_)­ppy)_2_(dtbbpy)]­PF_6_, CuTC, Na_2_CO_3_, MeCN, molecular sieves, 440 nm LED, rt, 16 h.

b Ir­(ppy)_3_, [Cu­(MeCN)_4_]­BF_4_, K_2_CO_3_, MeCN, 440 nm LED, rt, 18 h.

c Pd_2_(dba)_3_, RuPhos, Cs_2_CO_3_, DMF, 90 °C, 18 h.

d Pd­(dppf)­Cl_2_, CuI, NMM, dioxane, 40 °C, 48 h.

e Pd­(OAc)_2_, PPh_3_, NEt_3_, DMF, 100 °C, 24 h.

These specific PEG linkers were selected since they are frequently used to prepare bifunctional chemical probes, and in our experience, 1–2 PEG units minimally disrupt target binding if located at a solvent-exposed vector and commonly result in probes that retain favorable physicochemical properties.^12,28^ The specific linker lengths used in this study were informed primarily by commercial availability and expediency. For example, the secondary amine-containing linker **3c** was only available with one-PEG linkage between the secondary amine and Boc-protected ethylamine. The lengths of linkers **3d** and **3e** were informed by the synthetic route, with preparation from **3a** the most direct approach, and are slightly longer as a result. Differing linker lengths would not be expected to directly impact the success or failure of affinity pulldown, assuming similar phenotypic potency. The use of different cross-coupling reactions to install the linkers has two advantages; first, it enables the evaluation of different chemical bonds for attachment (ether, amine, carbon) that may influence the phenotypic potency of functionalized probes. Second, diverse thianthrene-containing substrates may not be compatible with all cross-coupling reactions (*vide infra*), hence multiple cross-couplings using different reaction conditions, would provide the greatest chance of successfully preparing a functionalized probe from the intermediate thianthrene salts.

Cross-coupling reactions with either the thianthrene or tetrafluorothianthrene salt of pyriproxyphen (**1**, **2**) were successful, *O-*linked probes (**4**) were prepared from a primary alcohol, *N*-linked from both primary (**5**) and secondary (**6**) amines, and *C*-linked through a Sonogashira (**7**) or Heck (**8**) reaction with acceptable yields. The Heck reaction provided an approximate 7:3 ratio of *trans:cis* isomers. Partial separation of the isomers was possible, however, to avoid complex product mixtures and lengthy purification procedures, this reaction was not investigated further. Both the tetrafluorothianthrene and thianthrene salts have been demonstrated to be competent and interchangeable in both transition metal-catalyzed and photoredox-coupling reactions.^18^

### Reaction Miniaturization and Screening of Phenotypically Active Compounds

Thianthrenation LSF reactions are considered to have a broad substrate scope.^18,19^ Ahmadli and colleagues recently parametrized arene reactivity and developed guidelines for selecting suitable thianthrenation conditions for a range of arene substrates.^19^ However, there will likely be chemical motifs outside of this substrate scope where the success and regioselectivity of the reaction will be difficult to predict. Scale down of the reaction to enable a high-throughput experimentation (HTE) approach would allow rapid assessment of the tractability of a large number of compounds toward thianthrenation, using submilligram quantities of material. During preparation of this manuscript, a machine-learning model capable of predicting the feasibility and regioselectivity of the thianthrenation reaction was reported.^29^ While a useful tool, the outputs still require experimental validation which could be achieved through our high throughput reaction format.

Pyriproxyphen was selected as a model substrate to investigate thianthrenation reaction miniaturization since it is known to provide high yields of the thianthrene product under standard reaction conditions.^18^ Starting with 10 mM pyriproxyphen (Table S1), replication of the batch-scale reaction conditions failed to form the thianthrenated product (**entry 1**), as determined by LC-MS. The expected color change did not occur under these conditions, in a standard mmol scale reaction a deep purple color is produced when thianthrene-*S*-oxide (TTO) is mixed with the HBF_4_·Et_2_O and trifluoroacetic anhydride (TFAA), indicating formation of reactive thianthrene species. On this smaller scale, 5 equiv of HBF_4_·Et_2_O (50 mM) and 12 equiv of TFAA (120 mM) were required for robust activation of TTO, with no formation of product detected below this level (**entries 2 and 3**). Further scale down of the reaction was required, initially we stepped down to 5 mM pyriproxyphen. Maintaining the optimal equivalents of HBF_4_·Et_2_O and TFAA from **entry 3** failed to produce the desired product (**entry 4**). When the concentration of HBF_4_·Et_2_O was increased (**entries 5 and 6**), partial conversion to product was observed, with complete conversion also requiring a higher concentration of TFAA (**entry 7**). These optimized conditions were also effective when the reaction was further scaled down to 1.25 mM pyriproxyphen (**entry 8**), while reducing the concentration of HBF_4_·Et_2_O impeded the reaction, with minimal formation of product (**entry 9**). Collectively, these data suggest that a minimal concentration of HBF_4_·Et_2_O (100 mM) and TFAA (120 mM) are required for robust activation of TTO on small scale, and that this concentration is well tolerated across a range of pyriproxyphen concentrations. The purple color of the activated thianthrenium species typically faded to colorless over 3 h, indicating complete conversion to product under these optimized conditions (**entries 7–8**). It should be noted that HBF_4_·Et_2_O is not the optimal acid for all substrates. With this in mind, optimized reaction conditions were also applied to reactions with BF_3_·Et_2_O and TfOH which are routinely used in reactions with basic *N*-heterocycles and electron-deficient arenes, respectively.^19^

### Substrate Scope of Miniaturized Reaction

To validate that this reaction format is broadly applicable, a range of aromatic compounds with varying electronic characteristics and substituents reported to participate in the thianthrenation reaction were subjected to our optimized reaction conditions in a plate-based format (Table S2). Initially, when setting up plate reactions, adding reaction components sequentially led to reaction initiation failures. Combining the HBF_4_·Et_2_O and TFAA into a single stock solution solved this issue and enabled robust activation across the plate. In general, compounds with electron donating groups proved to be good substrates under standard reaction conditions and were completely converted to their corresponding thianthrene product (**entries 1–5**). In contrast, *N*,*N*-dimethylaniline (**entry 6**) and toluene (**entry 7**) did not lead to appreciable amounts of product. For the former, it was hypothesized that this was due to protonation of the dimethylamine group deactivating the aromatic ring (the ammonium species being strongly electron withdrawing), an issue that has been a previously reported for basic heterocyclics.^18^ Replacement of HBF_4_·Et_2_O with a Lewis acid (BF_3_·Et_2_O) resulted in product formation, although conversion to product was incomplete within the 3 h of the reaction. Toluene (**entry 7**) has a very weak electron-donating group, and stronger acids (TfOH or BF_3_·Et_2_O) were required for product formation to be observed. All compounds with electron-withdrawing groups (**entries 8–11**) were unreactive under the standard reaction conditions, even when TfOH or BF_3_·Et_2_O were used in place of HBF_4_·Et_2_O. Electron-deficient substrates may react when using tetrafluorothianthrene-*S*-oxide, or a more reactive anhydride (e.g., TfO_2_), however, it has been reported tetrafluorothianthrene is not as well tolerated for subsequent functionalization chemistry^19^ and it was not deemed practical to use TfO_2_ on such a small scale without a significant increase in operational complexity due to its high reactivity with trace water. These results indicate that while it is possible to identify thianthrenation substrates using plate-based screening, there is a substrate limitation, particularly for electron-deficient aromatic rings. Since TfOH did not provide any advantage in substrate scope over HBF_4_·Et_2_O, or BF_3_·Et_2_O, it was excluded from further experiments.

### Miniaturised Thianthrenation of Phenotypically Active Compounds

Using compounds studied at the University of Dundee,^12,30–37^ we curated a set of 24 compounds previously demonstrating some degree of phenotypic activity against various kinetoplastid parasites. Here, we reassessed their potency specifically against *Leishmania donovani*, the causative agent of visceral leishmaniasis (Table 2). All 24 compounds in this set were active against *L. donovani* promastigotes, with EC_50_ values ranging from 0.002–40 μM. Next, we applied the miniaturized reaction format to this compound set (Table 2).^12,30–37^ When treated with HBF_4_·Et_2_O, seven compounds (**9, 15, 24, 26, 27, 29, 31**) formed the desired thianthrene product, as determined by LC-MS analysis, with conversions between 12 and 100%. The reaction with **15** resulted in low conversion to the product (12% conversion); however, the corresponding UV peak was <5% of the total area under the curve (AUC) and there was a significant amount of residual thianthrene due to unproductive reduction of thianthrene-*S*-oxide. This is an acknowledged side-reaction that can occur when an aromatic ring on the substrate is too electron rich, resulting in reaction failure and/or low conversion to product.^18^ Compound **9** provided modest conversion under these conditions (38%), with unreacted starting material and thianthrene-*S*-oxide. Notably, there was no significant amount of the reduced thianthrene species formed and after 3 h the reaction was still a deep purple color, indicative of a slower reaction. Reactions with the remaining compounds either failed or were contaminated with byproducts. Compounds **12, 13** and **15** contain very electron-rich aromatic rings; this has previously been identified as a limitation of the reaction substrate scope and may account for the failure of reactions **12** and **13**, and the low conversion to product observed for compound **15**.^18^ The remaining compounds generally lack any strong electron-donating substituents on aromatic rings, this low intrinsic reactivity accounted for the failure of these reactions. The use of a stronger activating agent, such as triflic anhydride could facilitate such reactions, although this was judged as impractical in a high-throughput format. It should be noted that compounds **10, 11, 18, 22, 23** and **25** contain motifs similar to aromatic substrates that have been successfully thianthrenated^18^ and while unreactive in this reaction format, could be suitable substrates under altered reaction conditions, with stronger activating agents such as triflic acid and triflic anhydride. This limitation was also observed for our test set of compounds (*vide supra*). When the reactions were instead performed with BF_3_·Et_2_O the reaction profile remained largely the same, with compounds **9**, **15**, **24, 26, 27, 29** and **31** providing a similar product profile, although with reduced conversion to the product for **9** (<5%). The product *m*/*z* was also detected for compounds **12**, **13**, **14** and **17**, although the corresponding UV signal was weak (<5% of the total AUC) and conversions were generally low (<20%), conversion for **12** and **13** are artificially high due to overlap with the reduced thianthrene byproduct. There was also significant formation of thianthrene from reduction of the thianthrene-*S*-oxide for these examples. The reaction with **31** also contained significant amounts of the thianthrene byproduct, despite a high apparent conversion (89%). A second examination of the LC-MS from the HBF_4_·Et_2_O reaction condition revealed that there was substantially more of this byproduct present than for the other successful reactions. Based on these observations, 5 compounds (**9**, **24**, **26, 27**, and **29**) that produced a moderate to high conversion to the thianthrenated product without significant byproduct contamination were progressed to scale up to determine the position of thianthrenation. Thianthrenation of electron-poor aromatic and heteroaromatic arenes is a limitation of this method, alternative strategies, such as the copper-catalyzed thianthrenation of arylborons could be used to extend the proposed workflow to electron-deficient arenes,^38^ with the requisite boron group installed via an iridium-catalyzed C–H borylation LSF reaction.^39^

**Table 2:**
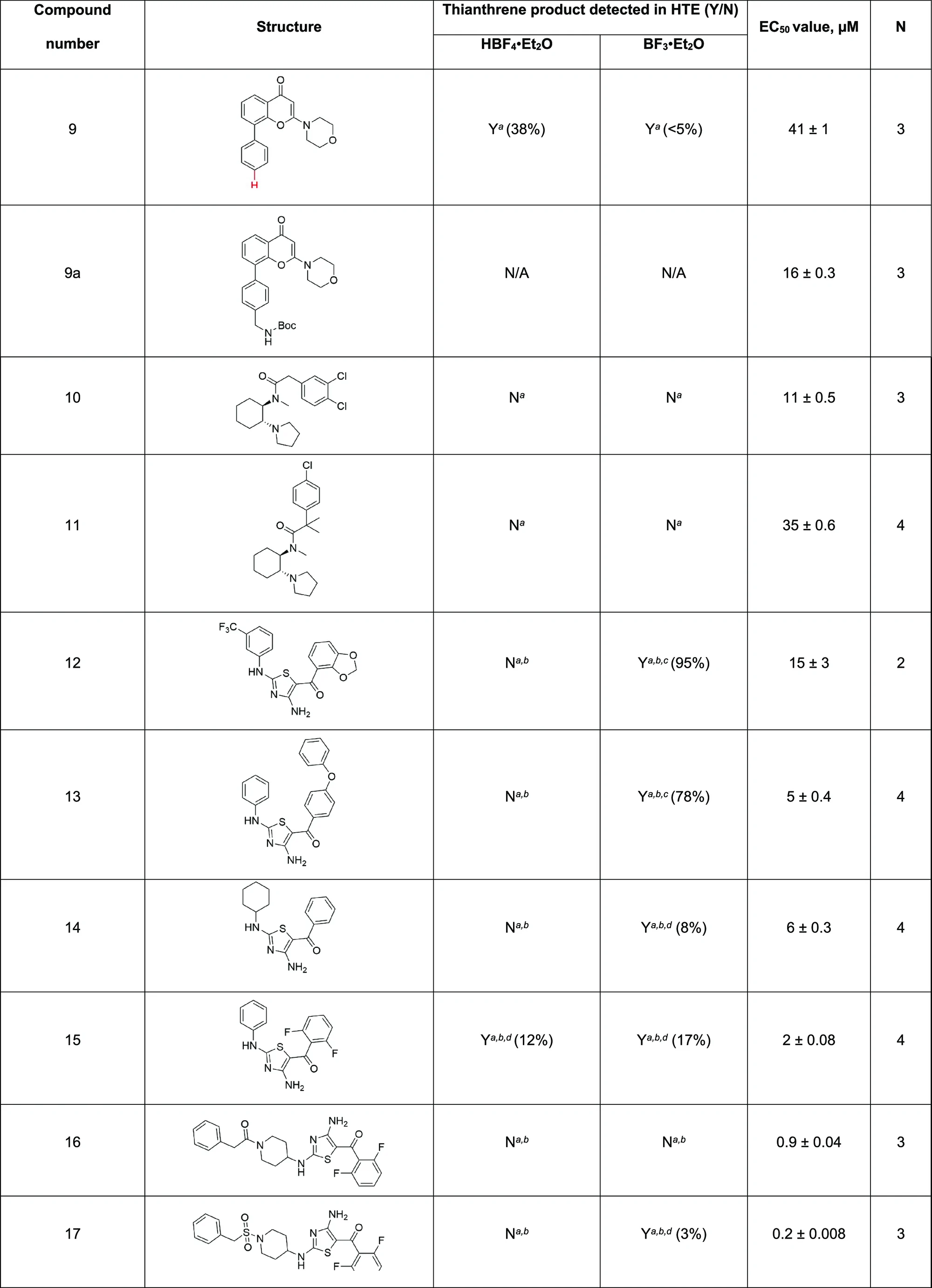

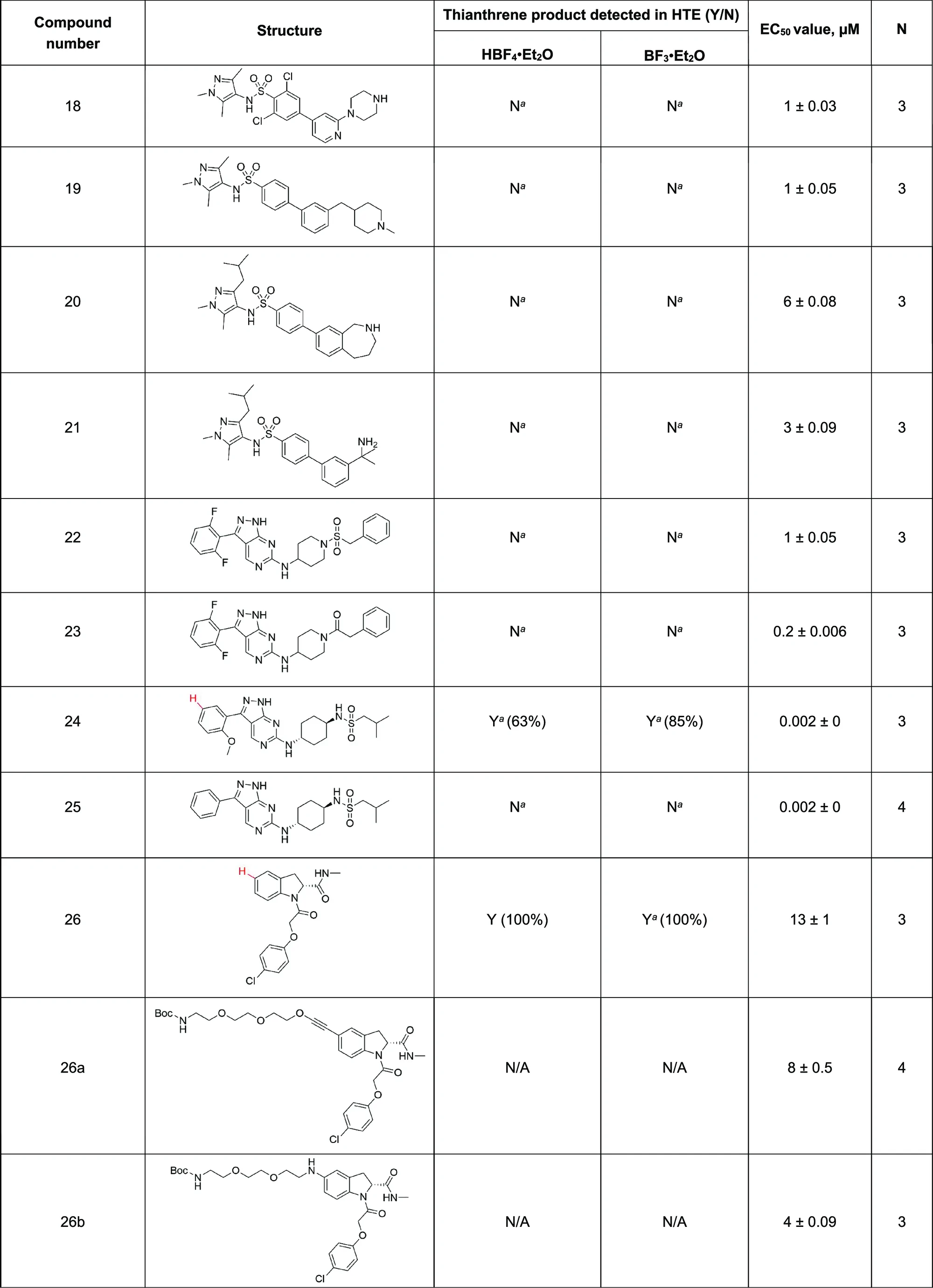

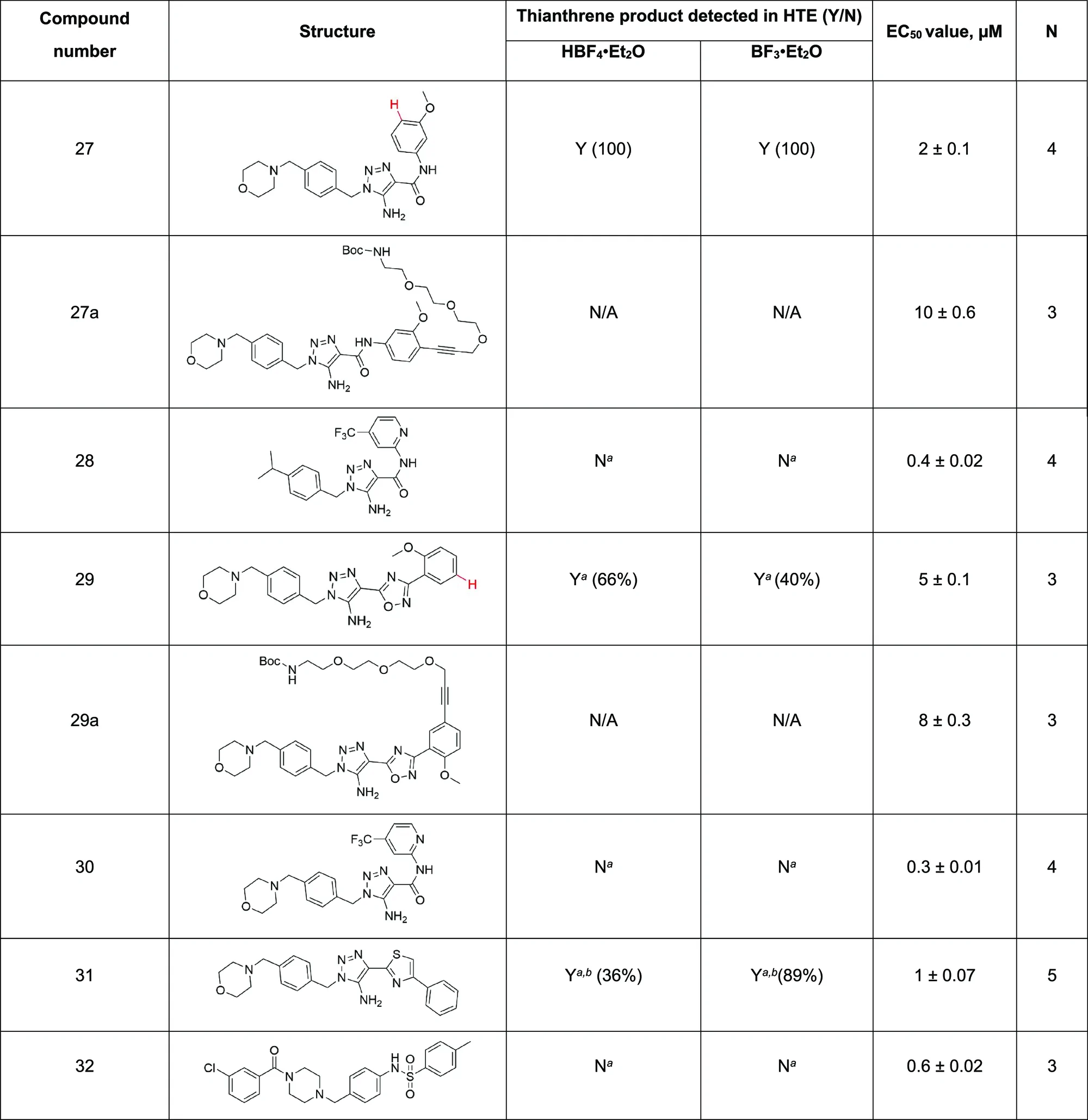
Structures, HTE Thianthrenation Outcome and EC_**50**_ Values of Selected Compound Library^e^

a Starting material present.

b Significant thianthrene byproduct (>30% total peak area at 254 nm).

c Product ion overlapped with thianthrene byproduct.

d Product <5% total peak area.

e EC_50_ values for selected compounds were determined against *L. donovani* promastigotes. Values represent the weighted mean ± SD of at least two biological replicates with each biological replicate composed of at least two technical replicates. HTE reaction conditions: 2.5 mM Ar–H susbtrate, 2.5 mM TTO, 200 mM acid, 240 mM TFAA, MeCN, 3 h; Y = yes, N = no, N/A = compound is a functionalized probe prepared from a thianthrenated compound and was not tested in the HTE workflow. Position of thianthrenation is highlighted in red for successful reactions that were progressed to scale up. Approximate conversion to product based on HPLC chromatogram is provided in brackets for successful reactions.

### Probe Synthesis and Biological Evaluation

Thianthrenation reactions with compounds **9**, **24**, **26**, **27** and **29** were scaled up, providing the corresponding thiathrene salts in moderate to good yields. 2D NMR analysis was used to confirm the position of thianthrenation (Figure 2, see SI for structural assignments). Compound **9**, also known as LY294002, is an established inhibitor of the phosphoinositde-3 kinase (PI3K) family and several bromodomain-containing proteins in mammalian cells.^40,41^ In addition, a modified derivative of **9**, CZC15098, has been used as a component of lipid kinobead technology.^42^ Compound **9** was elaborated to known affinity pulldown probe CZC15098 (**9b**) in three steps and 33% overall yield (Figure 3), a significant improvement on the reported six-step literature synthesis, which provided **9b** in 1.3% yield.^42^ Compound **24**, a confirmed inhibitor of the *L. donovani* Cdc-2-related kinase, CRK12 (*Ld*CRK12),^12^ underwent thianthrenation on its phenol-ether containing arene.

**Figure 2:**
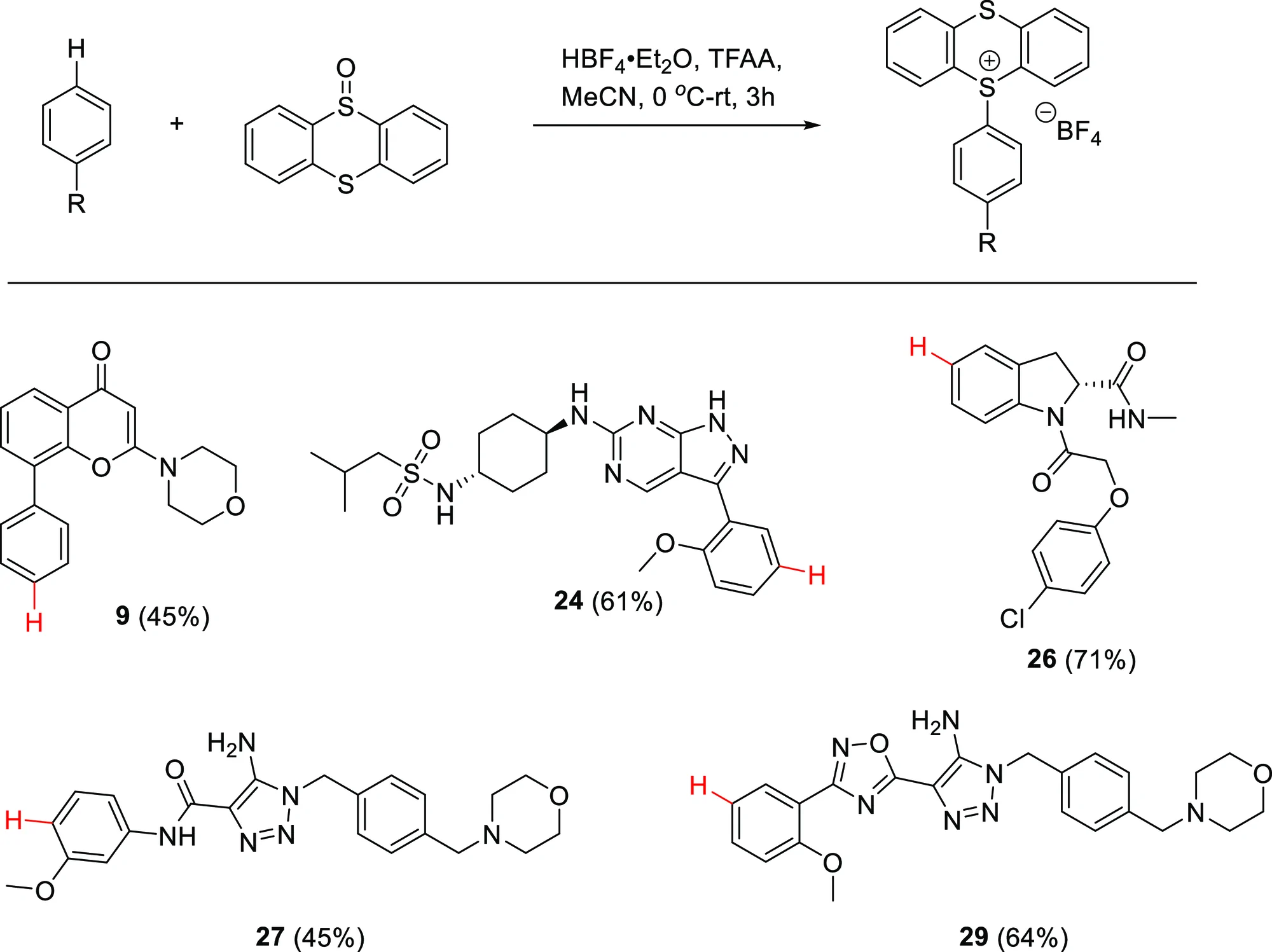
Phenotypically active compounds subjected to thianthrenation. Red colored hydrogen indicates the position of thianthrenation. Yields of thianthrenated products are indicated in parentheses.

**Figure 3:**
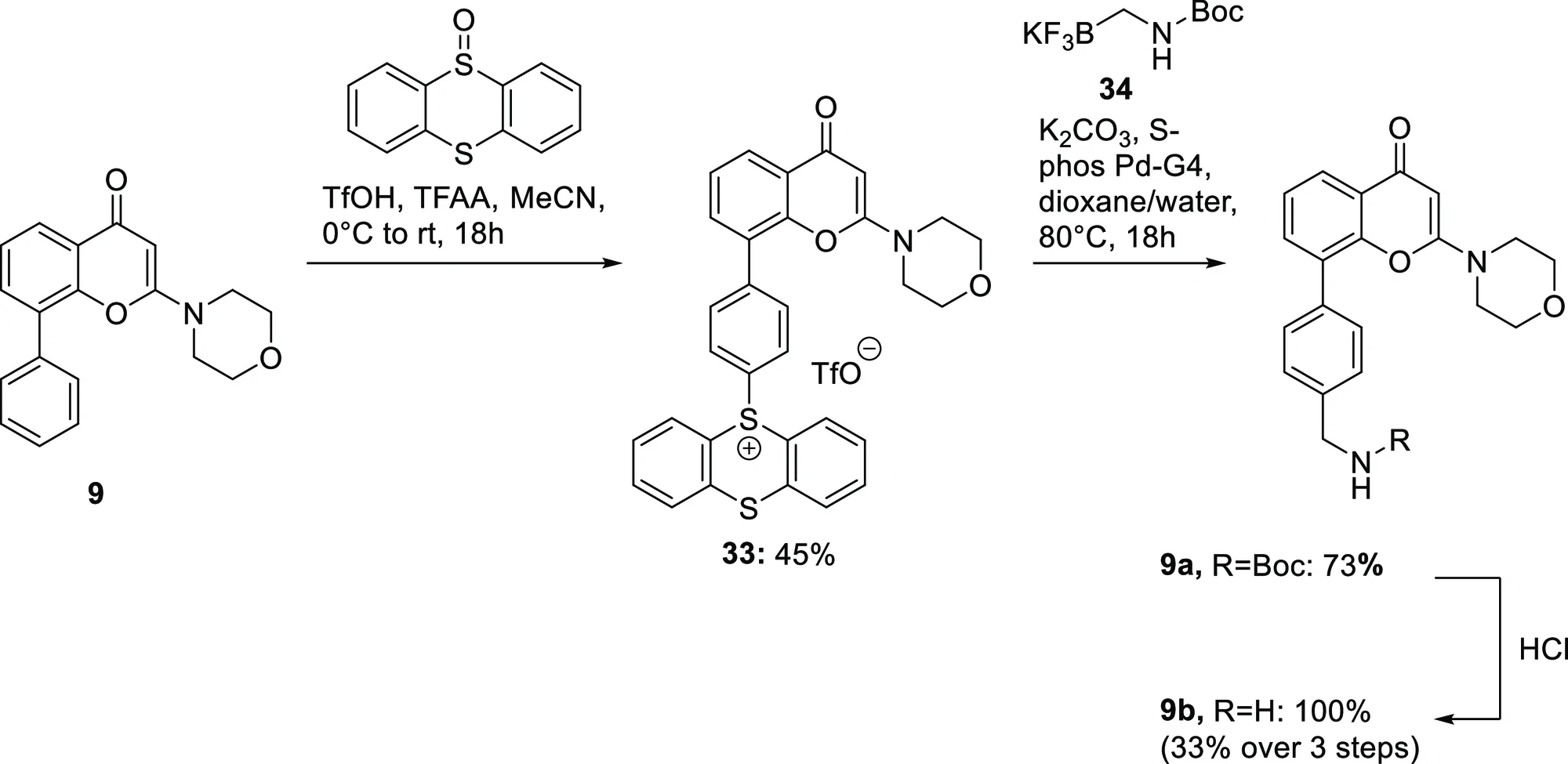
Thianthrenation of **9** and preparation of **9a** and **9b**.

Based on the crystal structure of *Ld*CRK12,^12^ incorporation of a linker at this position was expected to disrupt ligand binding and as a result this compound was not further elaborated. The remaining compounds (**26**, **27** and **29**) do not have an established molecular target and were subjected to the four previously identified cross-coupling reactions (Tables S3–S5). Four of the 12 cross-coupling reactions were successful, providing the PEG linker-functionalized probes **26a**, **26b**, **27a** and **29a**. Gratifyingly, there was at least one successful reaction for each substrate, highlighting the utility of having multiple chemistries to install a PEG spacer.

The phenotypic activity of probe-compounds **9a, 26a, 26b, 27a and 29a** was assessed against *L. donovani* (Table 2) and compared to the potency of the corresponding parent. Ideally, pulldown probes should retain phenotypic activity within ∼10-fold of the parent compound, with retained phenotypic activity acting as a surrogate for continued on-target activity. All of our pulldown probes met these criteria.

Probe **9a** was prioritized for a proof-of-concept chemical pulldown study in *L. donovani* (Figure 4A), due to knowledge of this compound’s mammalian targets.^42^ Target identification of compounds **26a, 26b**, **27a** and **29a** will be explored in future studies.

**Figure 4:**
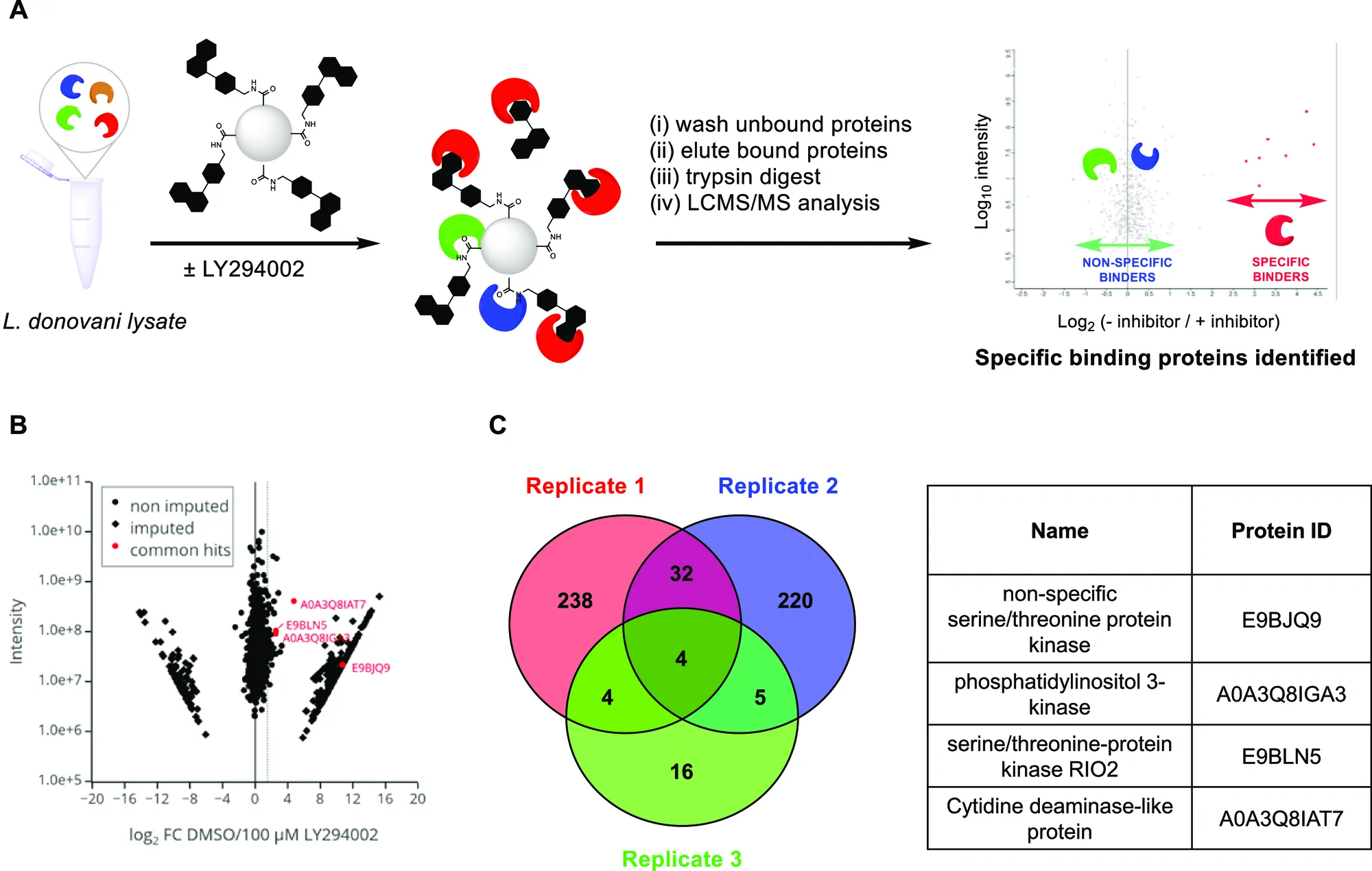
**LY294002 (9)** affinity pulldown. (A) Schematic representation of the affinity pulldown workflow. (B) Enrichment plot for replicate 1 of the **CZC15098 (9b)** affinity pulldown competed with 100 μM **LY294002 (9).** (C) Commonly enriched proteins in all three affinity pulldown replicates.

### Affinity Pulldown with **CZC15098** **(9b)** in *L. donovani* Cell Lysates

CZC15098 (**9b**) was attached to NHS-activated Sepharose resin using an established procedure,^13^ and incubated with *L. donovani* cell lysate in the presence or absence of LY294002 **(9)** (Figure 4A). Proteins bound to the affinity matrix were eluted, digested with trypsin and processed for mass spectrometry (LC-MS/MS)-based quantitative proteomics. Comparison of peptide intensities between LY294002-competed versus noncompeted samples identified proteins binding specifically to the affinity matrix. We employed data imputation, where the lowest observed mass spectrometry intensity is applied during data analysis to account for missing values caused by complete competition of some kinases in the LY294002-treated samples.^43^ *L. donovani* phosphoinositide kinases (PIKs) and PIK-related kinases (PIKKs) were identified through Gene Ontology (GO) term and InterPro domain searches (Table S6). Three replicates were performed (Figures 4B and S1) and four proteins were found to be consistently enriched across all replicates (Figure 4C). These proteins included a PI3K (A0A3Q8IGA3), and a nonspecific serine/threonine protein kinase which was annotated as a PI3K complex subunit (E9BJQ9). Our hypothesis is that this subunit may have been coenriched alongside PI3K A0A3Q8IGA3 as part of a protein complex, rather than being a direct target of LY294002 (**9**). Two additional targets were also reproducibly enriched, cytidine deaminase-like protein (A0A3Q8IAT7) and serine/threonine protein kinase RIO2 (EBLN5). Notably, the human orthologue of EBLN5, RIO2 kinase, was previously identified as a secondary target of LY294002 in human cells.^42^

To generate more nuanced information, we repeated pulldowns with probe **9b** in the presence of dose-dependent competition from the parent at concentrations ranging from 0.01–100 μM (Figure 5A). By plotting the abundance of protein peptides as a function of competitor concentration, this variant of chemical pulldown can provide invaluable information regarding the relative binding affinities of individual targets. This can effectively denoise hits identified from single-dose affinity pulldown experiments by eliminating any putative targets that do not display dose-responsive behavior and can also allow ranking of enriched target proteins for target validation based on approximated affinity. All four putative targets proteins previously identified from our preliminary pulldown experiment displayed a dose-responsive behavior (Figure 5B–E), with similar affinity. Interestingly, for the PI3K and associated targets, data imputation was required to observe the protein only at the highest concentration of competitor, supporting our theory that in the presence of high inhibitor competition, target binding can be depleted below the limits of mass spectrometry detection. This affinity matrix has previously been used as a component of the lipid kinobead technology in mammalian cells, where it can enrich four class one PI3K subunits, and the PI3K-related kinase mTOR,^42^ which represents over a quarter of human PI3Ks.^41^ Additional PI3K inhibitor-affinity matrices would need to be explored to enable effective lipid-kinase profiling in *L. donovani*

**Figure 5:**
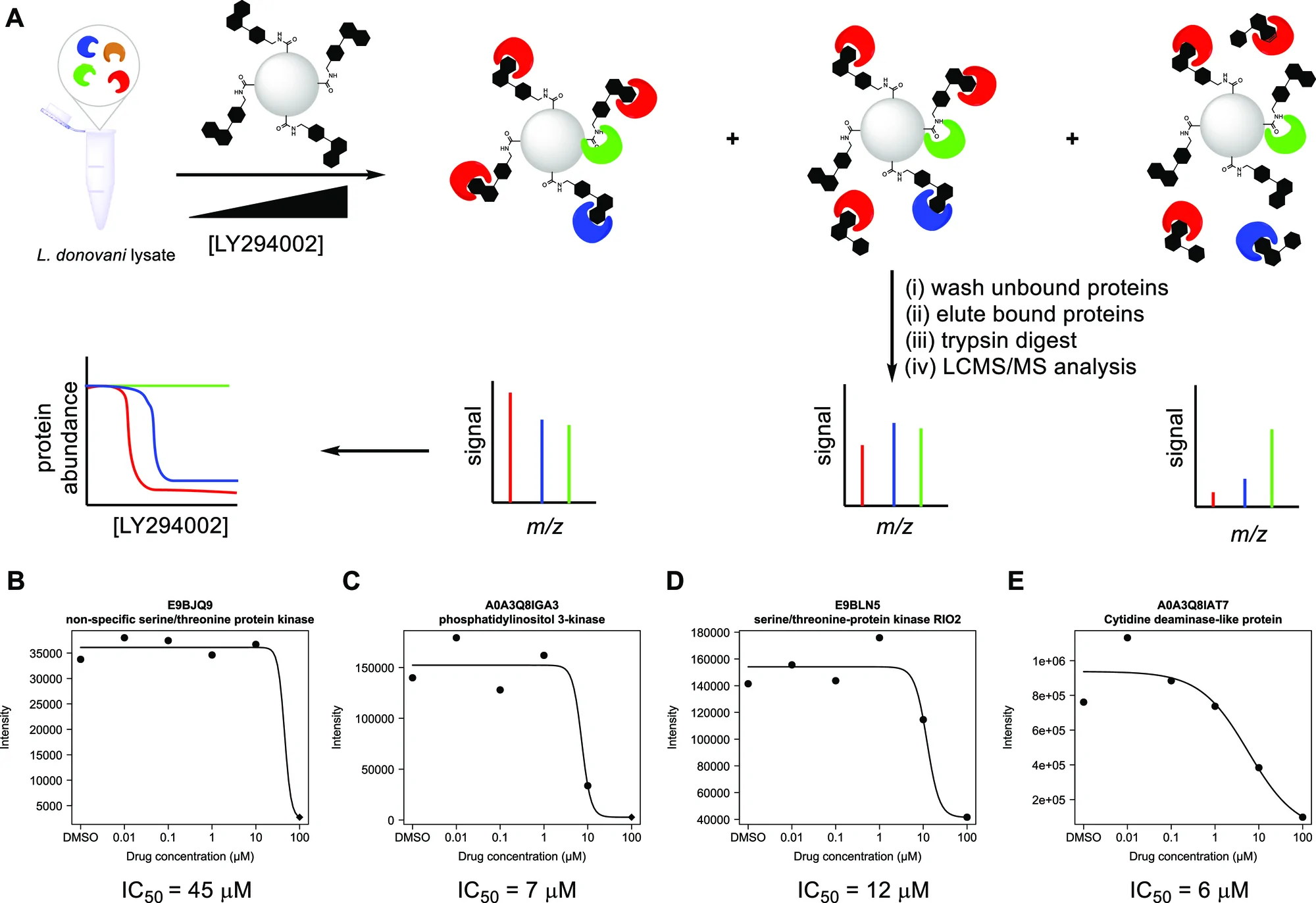
**LY294002 (9)** dose–response pulldown. (A) Schematic representation of the dose–response pulldown strategy. (B–E) dose–response curves and IC_50_ values for hits identified through chemical pulldown.

## Conclusions

A proof-of-concept workflow was developed using HTE to assess phenotypic hit compounds for tractability toward the thianthrenation LSF reaction. This work identified five suitable substrates from a 24 compound set that were suitable for thianthrenation. Affinity pulldown probes were successfully prepared and compound **9b** was advanced to an affinity pulldown experiment. Four high-confidence protein targets were consistently identified, including the expected PI3K-related targets. These results highlight the utility of the thianthrenation reaction to expedite the synthesis of functionalized tool compounds for downstream applications. The resulting functionalized tool compounds can facilitate multiple approaches including affinity pulldown, as described here, the synthesis of biotinylated probes, PROTACs, and photocatalyst conjugates used in microenvironment mapping. The development of a broad toolkit of LSF reactions with wide substrate scope and orthogonal chemo- and regioselectivity would enable further efficiencies in the synthesis of functionalized tool compounds.

## Experimental Section

### General

All solvents were purchased from commercial suppliers at reagent grade or higher and used without further purification. Dry solvents were purchased fitted with AcroSeal lids and used as supplied. Air and moisture sensitive reactions were performed under an inert nitrogen atmosphere using oven-dried glassware.

Normal phase flash column chromatography was performed on a Combiflash nextgen 300+ (Teledyne ISCO) using redisep silver prepacked cartridges. Reverse-phase flash chromatography was performed on a Combiflash *R*_f_+ (Teledyne ISCO) using redisep gold prepacked C18 cartridges. Preparative HPLC was performed on a Waters mass-directed preparative HPLC (acidic method: X-select CSH prep 5 μm OBD 19 × 100 mm column, 5:95–95:5 water/acetonitrile (0.1% formic acid), 25 mL min^–1^, 10 min gradient; basic method: X-bridge prep C18 5 μm OBD 19 × 100 mm column, 5:95–95:5 water/acetonitrile (0.1% NH_3_), 25 mL min^–1^, 10 min gradient), or Gilson preparative HPLC system (X-select CSH prep 5 μm OBD 19 × 100 mm column, 5:95–95:5 water/acetonitrile (0.1% formic acid), 25 mL min^–1^, 10 min gradient).

^1^H NMR spectra were recorded on a Bruker Avance DPX 500 spectrometer (^1^H at 500.1 MHz, ^13^C at 125 MHz, ^19^F at 470.5 MHz). Chemical shifts (δ) are expressed in ppm recorded using the residual solvent as the internal reference in all cases. Signal splitting patterns are described as singlet (s), doublet (d), triplet (t), quartet (q), multiplet (m), broad (br), or a combination thereof. Coupling constants (J) are quoted to the nearest 0.1 Hz. Low resolution electrospray ionization (ESI) mass spectra were recorded on either an Advion Expression Compact Mass Spectrometer connected to a ThermoDionex Ultimate 3000 UPLC or a Shimadzu LCMS 2020 system. High resolution ESI-MS measurements were performed on either an Orbitrap Exploris 120 equipped with a Thermo scientific Hypersil GOLD 50 mm × 2.1 mm column 1.9 μM particle size or a Waters Xevo QTOF.

Compounds have been named using the ChemDraw Ultra 22.0 naming application which is commercially available from Revvity Signals.

All stock solutions used in the synthesis of drug beads were prepared in high grade DMSO (Acros, 99.8%, for molecular biology). Drug beads were stored in molecular biology grade 2-propanol (iPrOH) (Sigma-Aldrich, BioReagent, for molecular biology, >99.5%). Drug bead reactions were mixed using a Miltenyi Biotec MACSmix Tube Rotator.

Photochemical reactions were carried out using either a 32 W 440 nm LED Kessil Lamp or using an Asynt Lightsyn Illumin8 parallel photoreactor with 10 W 450 nm LED array. Reaction yields are generally comparable between these two light sources. Commercially sourced catalysts (Ir­(ppy)_3_, [Ir­(dF­(CF_3_)­ppy)_2_(dtbbpy)]­PF_6_, Pd_2_(dba_3_), RuPhos, Pd­(dppf)­Cl_2_) were immobilized 1% on chembeads following the reported literature procedure for preparation with a vortex mixer.^44^

All compounds are >95% pure by HPLC analysis.

### General Procedures

#### General Procedure A. Thianthrenation Reaction

Procedure adapted from Berger et al. (2019).^18^ To an arene substrate (1 equiv) in dry MeCN (0.1–0.2 M) was added sequentially HBF_4_·Et_2_O (1–2 equiv), TfOH (1–2 equiv) or BF_3_·Et_2_O (1–2 equiv) and TFAA (3 equiv). The reaction was cooled in an ice bath followed by addition of thianthrene*-S-*oxide (1–1.2 equiv) and HBF_4_·Et_2_O (1–2 equiv) or BF_3_·Et_2_O (1–2 equiv). Initiation of the reaction was indicated by a color change to a deep purple/blue solution. The reaction was stirred for 1 h then removed from the ice bath and stirred until the reaction was complete (as indicated by LCMS), typically 2 h. After this time methanol (5 mL) was added to quench the reaction and the volatiles removed *in vacuo*. The residue was partitioned between DCM (20 mL) and saturated sodium bicarbonate solution (aq, 20 mL). The aqueous phase was extracted with DCM (20 mL) and the combined organic layers washed with saturated sodium bicarbonate solution (aq, 20 mL), NaBF_4_ solution (5% w/w, 2 × 20 mL), water (2 × 20 mL). The DCM layer was dried over solid Na_2_SO_4_, filtered and reduced *in vacuo*. The residue was purified by column chromatography or dissolved in 3:1 MeCN:water, filtered through a 0.4 μM syringe filter and purified by preparative HPLC to provide the product.

#### General Procedure B. Etherification Reaction with Thianthrene Salt

Procedure is adapted from Sang et al. (2019).^22^ To a 5 mL oven-dried glass vial or to an Asynt Octomini reaction vial (if Asynt photoreactor is used) was added copper thiophene-2-carboxylate (CuTC) (2 equiv), 3 Å molecular sieves (approximately 200 mg) and Na_2_CO_3_ (1 equiv). The vial was sealed and placed under a nitrogen atmosphere (vacuum cycling × 3). A solution of *tert*-butyl (2-(2-(2-hydroxyethoxy)­ethoxy)­ethyl)­carbamate (5 equiv) in dry MeCN (0.2 M) was added and the reaction stirred for 2 h. After this time a solution of thianthrene salt (1 equiv) and [Ir­(dF­(CF_3_)­ppy)_2_(dtbbpy)]­PF_6_ (1 mol %, if <2 mg was required this reagent was immobilized 1% on chembeads) in dry MeCN (0.2 M) was added. If the thianthrene salt was insoluble in MeCN, a minimal volume of DMF was added to solubilize this reagent. The reaction was then irradiated with either a 32 W Kessil lamp using a USB fan for cooling to maintain the reaction at approximately 30 °C (an orange UV shield was in place over the reaction for the duration), or in an Asynt Lightsyn Illumin8 photoreactor with fan cooling directed at the reactions, temperature maintained at approximately 36–40 °C for 24 h. After this time the reaction was reduced *in vacuo* and the residue dissolved in 3:1 MeCN:water, filtered through a 0.4 μM syringe filter, and purified by preparative HPLC to provide the product.

#### General Procedure C. Buchwald-Hartwig Reaction with Thianthrene Salt—Primary Amines

Procedure is adapted from Engl et al. (2019).^23^ To a 5 mL oven-dried glass vial or to an Asynt Octomini reaction vial (if asynt photoreactor is used) was added thianthrene salt (1 equiv), [Cu­(MeCN_4_)]­BF_4_ (1 equiv), K_2_CO_3_ (2 equiv) and Ir­(ppy)_3_ (2 mol %, if <2 mg was required this reagent was immobilized 1% on chembeads). The vial was sealed and placed under a nitrogen atmosphere via vacuum cycling (×3). A solution of *tert*-butyl (2-(2-(2-aminoethoxy)­ethoxy)­ethyl)­carbamate (1.5 equiv) in dry MeCN (0.2 M) was added and the reaction irradiated with either a 32 W Kessil lamp using a USB fan for cooling to maintain the reaction at approximately 30 °C (an orange UV shield was in place over the reaction for the duration), or in an Asynt Lightsyn Illumin8 photoreactor with fan cooling directed at the reactions, temperature maintained at approximately 36–40 °C for 24 h. After this time the reaction was diluted with methanol (1 mL) and filtered through Celite (2.5 g prepacked cartridge), using methanol (10 mL) to flush the filter cartridge. The volatiles were removed *in vacuo*, the residue dissolved in 3:1 MeCN:water, filtered through a 0.4 μM syringe filter and purified by preparative HPLC to provide the product.

#### General Procedure D. Buchwald-Hartwig Reaction with Thianthrene Salt—Secondary Amines

Procedure is adapted from Engl et al. (2019).^23^ To a 5 mL oven-dried glass vial was added thianthrene salt (1 equiv), Pd_2_(dba)_3_ (2 mol %, if <2 mg was required this reagent was immobilized 1% on chembeads), Ruphos (4 mol %, if <2 mg was required this reagent was immobilized 1% on chembeads) and Cs_2_CO_3_ (2 equiv). The vial was sealed and placed under a nitrogen atmosphere via vacuum cycling (×3). A solution of *tert*-butyl (2-(2-(methylamino)­ethoxy)­ethyl)­carbamate (3 equiv) in dry DMF (0.2 M) was added and the vial heated at 90 °C for 20 h. After this time, solvent was removed on a spiral evaporator and the residue purified by column chromatography (silica, EtOAc/heptane) or dissolved in 3:1 MeCN:water, filtered through a 0.4 μM syringe filter and purified by preparative HPLC to provide the product.

#### General Procedure E. Sonogashira Reaction with Thianthrene Salt

Procedure is adapted from Berger et al. (2019).^18^ To a 5 mL oven-dried glass vial was added thianthrenium salt (1 equiv), Pd­(dppf)­Cl_2_ (5 mol %, if <2 mg was required this reagent was immobilized 1% on chembeads) and CuI (20 mol %). The vial was sealed and placed under a nitrogen atmosphere via vacuum cycling (×3). A solution of *tert*-butyl (2-(2-(2-(prop-2-yn-1-yloxy)­ethoxy)­ethoxy)­ethyl)­carbamate (2 equiv) and *N*-methylmorpholine (2 equiv) in dry 1,4-dioxane (0.2 M) was added and sparged with nitrogen gas for 5 min. The reaction was heated to 40 °C for 48–72 h until consumption of thianthrene salt as indicated by LCMS analysis. After this time the reaction was filtered through Celite (2.5 g prepacked cartridge) using DCM (10 mL) to wash the filter cartridge and the solvent reduced *in vacuo*. The residue was purified by column chromatography (silica, 0–100% EtOAc/heptane) or dissolved in 3:1 MeCN:water, filtered through a 0.4 μM syringe filter and purified by preparative HPLC to provide the product.

##### 2,3,7,8-Tetrafluoro-5-(4-(4-(2-(pyridin-2-yloxy)­propoxy)­phenoxy)­phenyl)-5*H*-thianthren-5-ium Tetrafluoroborate (**1**)

Following **General procedure A**, pyriproxyphen (496 mg, 1.54 mmol) was reacted with tetraflorothianthrene-*S*-oxide (470 mg, 1.54 mmol), HBF_4_·Et_2_O (210 μL, 1.54 mmol), TFAA (644 μL, 4.63 mmol) and MeCN (6.2 mL). Thianthrene (15 mg, 0.077 mmol) was added and was necessary to activate the tetrafluorothianthrene-*S*-oxide. Purification by column chromatography (24 g silica, 0:100 → 10:90 MeOH:DCM) provided the title compound as a white solid (420 mg, 0.574 mmol, 37%).

^1^H­{^19^F} NMR (500 MHz, CDCl_3_, 303 K) δ 8.54 (s, 2H, 2 × ArH), 8.14 (dt, *J* = 5.5, 1.4 Hz, 1H, ArH), 7.65 (s, 2H, 2 × ArH), 7.57 (ddd, *J* = 8.3, 7.1, 2.0 Hz, 1H, ArH), 7.31–7.27 (m, 2H, 2 × ArH), 6.99–6.90 (m, 6H, 6 × ArH), 6.86 (ddd, *J* = 7.1, 5.0, 1.0 Hz, 1H, ArH), 6.75–6.71 (m, 1H, ArH), 5.58 (dtd, *J* = 11.5, 6.3, 5.0 Hz, 1H, CH), 4.20 (dd, *J* = 9.9, 5.3 Hz, 1H, C*H*H), 4.07 (dd, *J* = 9.9, 4.9 Hz, 1H, C*H*H), 1.47 (d, *J* = 6.4 Hz, 3H, CH_3_).

^19^F­{^1^H} NMR (471 MHz, CDCl_3_, 303 K) δ −121.78 (d, *J* = 20.8 Hz, 2 × CF), −128.38 (d, *J* = 20.9 Hz, 2 × CF), −149.55 (br s, BF_4_).

MS (ES+): *m*/*z* (%) 513 (100) [M–C_5_H_6_NO]^+^, 608.0 (30) [M]^+^.

Analytical data are consistent with that reported in Berger et al. (2019).^18^

##### 5-(4-(4-(2-(Pyridin-2-yloxy)­propoxy)­phenoxy)­phenyl)-5*H*-thianthren-5-ium tetrafluoroborate (**2**)

Following **General procedure A**, pyriproxyphen (500 mg, 1.56 mmol) was reacted with thianthrene-*S*-oxide (361 mg, 1.56 mmol), HBF_4_·Et_2_O (212 μL, 1.56 mmol), TFAA (650 μL, 4.67 mmol) and MeCN (6.2 mL). Purification by column chromatography (24 g silica, 0:100 → 10:90 MeOH:DCM) provided the title compound, which was dried under vacuum to form a white solid (400 mg, 0.61 mmol, 39%).

^1^H NMR (500 MHz, CDCl_3_, 303 K) δ 8.61–8.52 (m, 2H, 2 × ArH), 8.14 (ddd, *J* = 5.0, 2.0, 0.8 Hz, 1H, ArH), 7.83–7.71 (m, 6H, 6 × ArH), 7.56 (ddd, *J* = 8.4, 7.1, 2.0 Hz, 1H, ArH), 7.24–7.18 (m, 2H, 2 × ArH), 6.98–6.87 (m, 6H, 6 × ArH), 6.86 (ddd, *J* = 7.2, 5.1, 1.0 Hz, 1H, ArH), 6.73 (dt, *J* = 8.4, 0.9 Hz, 1H, ArH), 5.57 (dtd, *J* = 11.5, 6.4, 5.0 Hz, 1H, CH), 4.18 (dd, *J* = 9.9, 5.3 Hz, 1H, C*H*H), 4.06 (dd, *J* = 9.9, 4.9 Hz, 1H, C*H*H), 1.47 (d, *J* = 6.4 Hz, 3H, CH_3_).

MS (ES+): *m*/*z* (%) 137.0 (40) [M-C_24_H_17_O_2_S_2_]^+^ 441 (100) [M-C_5_H_6_NO]^+^, 536.1 (80) [M]^+^.

Analytical data are consistent with that reported in Berger et al. (2019).^18^

##### *tert*-Butyl *N*-[2-[2-(2-prop-2-ynoxyethoxy)­ethoxy]­ethyl]­carbamate (**3d**)

To a round-bottom flask was added NaH (184 mg, 4.61 mmol) and THF (10 mL), which was cooled in an ice bath, followed by slow addition of a solution of *tert*-butyl (2-(2-(2-hydroxyethoxy)­ethoxy)­ethyl)­carbamate (**3a**) (1044 mg, 4.19 mmol) in THF (5 mL). After stirring for 30 min 3-bromo-1-propyne (0.677 mL, 6.28 mmol) was added dropwise and the reaction heated to reflux and monitored by TLC. After 18 h the reaction was diluted with EtOAc (10 mL) and washed with water (20 mL) and brine (20 mL). The organic phase was then dried over magnesium sulfate, filtered and reduced *in vacuo*. Purification by column chromatography (24 g silica, 0:100 → 100:0 EtOAc:Heptane) collecting all fractions as the product is not UV active. Product containing fractions were identified by TLC (1:1 EtOAc:heptane staining with potassium permanganate, *R*_f_ ∼ 0.5) to provide the title compound as a colorless oil (608 mg, 2.01 mmol, 48%).

^1^H NMR (400 MHz, CDCl_3_, 303 K) δ 5.00 (s, 1H, NH), 4.21 (d, 2H, *J* = 2.4 Hz, CH_2_), 3.73–3.67 (m, 4H, 2 × CH_2_), 3.66–3.59 (m, 4H, 2 × CH_2_), 3.54 (t, 2H, *J* = 5.1 Hz, CH_2_), 3.35–3.27 (m, 2H, CH_2_), 2.42 (t, 1H, *J* = 2.4 Hz, CH), 1.45 (s, 9H, 3 × CH_3_).

^13^C NMR (101 MHz, CDCl_3_, 303 K) δ 156.2 (C=O), 79.8 (C/CH), 79.3 (C), 74.7 (C/CH), 70.7 (CH_2_), 70.6 (CH_2_), 70.4 (CH_2_), 69.3 (CH_2_), 58.6 (CH_2_), 40.6 (CH_2_), 28.6 (CH_3_).

11/12 expected ^13^C resonances observed, the missing resonance is due to overlap of CH_2_ signals at 70.3 ppm. The protonated alkyne carbon could not be identified, as it did not show a HSQC correlation to the corresponding proton resonance, although it is the ^13^C peak at either 79.8 or 74.7 ppm.

MS (ES+): *m*/*z* (%) 188 (100) [M–C_5_H_9_O_2_]^+^, 310 (50) [M + Na]^+^.

##### *tert*-Butyl *N*-[2-[2-(2-allyloxyethoxy)­ethoxy]­ethyl]­carbamate (**3e**)

To a round-bottom flask was added NaH (187 mg, 4.70 mmol) and THF (10 mL), which was cooled in an ice bath, followed by slow addition of a solution of *tert*-butyl (2-(2-(2-hydroxyethoxy)­ethoxy)­ethyl)­carbamate (**3a**) (1065 mg, 4.27 mmol) in THF (5 mL). After stirring for 30 min, 3-bromoprop-1-ene (0.555 mL, 6.41 mmol) was added dropwise and the reaction heated to reflux and monitored by TLC. After 18 h the reaction was diluted with EtOAc (10 mL), washed with water (20 mL) and brine (20 mL). The organic phase was then dried over magnesium sulfate, filtered and reduced *in vacuo*. Purification by column chromatography (24 g silica, 0:100 → 100:0 EtOAc:Heptane) collecting all fractions as the product is not UV active. Product containing fractions were identified by TLC (1:1 EtOAc:heptane staining with potassium permanganate, *R*_f_ ∼ 0.5) to provide the title compound as a colorless oil (696 mg, 2.29 mmol, 53.5%).

^1^H NMR (400 MHz, CDCl_3_, 303 K) δ 5.91 (ddt, *J* = 17.2, 10.4, 5.6 Hz, CH), 5.27 (qd, 1H, *J* = 1.6, 17.2 Hz, alkene–C*H*H), 5.18 (qd, 1H, *J* = 1.4, 10.4 Hz, alkene–C*H*H), 5.00 (s, 1H, NH), 4.03 (td, 2H, *J* = 1.4, 5.6 Hz, CH_2_), 3.68–3.59 (m, 8H, 4 × CH_2_), 3.54 (t, 2H, J = 5.2 Hz, CH_2_), 3.35–3.27 (m, 2H, CH_2_), 1.44 (s, 9H, 3 × CH_3_).

^13^C NMR (101 MHz, CDCl_3_, 303 K) δ 156.2 (C=O), 134.9 (CH), 117.2 (CH_2_), 79.3 (C), 72.4 (CH_2_), 70.83 (CH_2_), 70.76 (CH_2_), 70.50 (CH_2_), 70.4 (CH_2_), 69.6 (CH_2_), 40.6 (CH_2_), 28.6 (CH_3_).

MS (ES+): *m*/*z* (%) 190 (100) [M–C_5_H_9_O_2_]^+^, 312 (75) [M + Na]^+^.

##### *tert*-Butyl (2-(2-(2-(4-(4-(2-(pyridin-2-yloxy)­propoxy)­phenoxy)­phenoxy)­ethoxy)­ethoxy) ethyl) carbamate (**4**)

Following **1** (50 mg, 0.072 mmol) was reacted with CuTC (27 mg, 0.144 mmol), Na_2_CO_3_ (8 mg, 0.072 mmol), *tert*-butyl (2-(2-(2-hydroxyethoxy)­ethoxy)­ethyl)­carbamate (**3a**) (90 mg, 0.36 mmol) and [Ir­(dF­(CF_3_)­ppy)_2_(dtbbpy)]­PF_6_ (0.81 mg, 0.0007 mmol) in MeCN (0.8 mL) using a 440 nm Kessil lamp for irradiation. Purification by column chromatography (4 g silica, 0:100 → 100:0 EtOAc:Heptane) followed by preparative HPLC (5:95 → 95:5 MeCN:water (0.1% formic acid)) provided the title compound as a colorless oil (13.7 mg, 0.023 mmol, 32%).

^1^H NMR (500 MHz, CDCl_3_, 303 K) δ 8.14 (ddd, 1H, *J* = 0.7, 1.9, 5.0 Hz, ArH), 7.56 (ddd, 1H, *J* = 2.0, 7.0, 8.4 Hz, ArH), 6.90–6.83 (m, 9H, 9 × ArH), 6.73 (td, 1H, *J* = 0.8, 8.4 Hz, ArH), 5.61–5.54 (m, 1H, CH), 4.98 (s, 1H, NH), 4.17 (dd, 1H, *J* = 5.3, 9.8 Hz, C*H*H), 4.12–4.09 (m, 2H, CH_2_), 4.05 (dd, 1H, *J* = 4.9, 9.9 Hz, C*H*H), 3.86–3.83 (m, 2H, CH_2_), 3.73–3.70 (m, 2H, CH_2_), 3.66–3.63 (m, 2H, CH_2_), 3.55 (t, 2H, *J* = 5.2 Hz, CH_2_), 3.35–3.27 (m, 2H, CH_2_), 1.47 (d, 3H, *J* = 6.4 Hz, CH_3_), 1.43 (s, 9H, 3 × CH_3_).

^13^C NMR (126 MHz, CDCl_3_, 303 K) δ 163.4 (C=O), 154.9 (C), 154.7 (C), 152.0 (C), 151.9 (C), 147.0 (CH), 138.8 (CH), 119.7 (CH), 119.6 (CH), 116.9 (CH), 116.0 (CH), 115.9 (CH), 111.8 (CH), 71.4 (CH_2_), 70.9 (CH_2_), 70.5 (CH_2_), 70.0 (CH_2_), 69.5 (CH), 68.2 (CH_2_), 40.6 (CH_2_), 28.6 (CH_3_), 17.2 (CH_3_).

Twenty two ^13^C resonances are observed, accounting for 28 of31 carbon atoms.· One CH_2_ missing due to coincidental overlap at 70.5 ppm. The Boc quaternary carbon is missing due to low intensity, one quaternary aromatic carbon resonance is also missing, either due to coincidental overlap or low intensity.

HRMS (ES+): calcd. for C_31_H_41_N_2_O_8_ [M + H]^+^ 569.2863; found 569.2885 (3.9 ppm).

##### *tert*-Butyl (2-(2-(2-((4-(4-(2-(pyridin-2-yloxy)­propoxy)­phenoxy)­phenyl)­amino)­ethoxy) ethoxy)­ethyl) carbamate (**5**)

Following **General procedure C**, **1** (50 mg, 0.072 mmol) was reacted with *tert*-butyl (2-(2-(2-aminoethoxy)­ethoxy)­ethyl)­carbamate (**3b**) (27 mg, 0.108 mmol), K_2_CO_3_ (20 mg, 0.0144 mmol), [Cu­(MeCN)_4_]­BF_4_ (23 mg, 0.072 mmol), Ir­(ppy)_3_ (0.95 mg, 0.0014 mmol) and MeCN (0.4 mL) using a 440 nm Kessil lamp for irradiation. Purification by column chromatography (4 g silica, 0:100 → 100:0 EtOAc:Heptane) followed by preparative HPLC (5:95 → 95:5 MeCN:water (0.1% formic acid)) provided the title compound as a yellow oil (23 mg, 0.0397 mmol, 55%).

^1^H NMR (500 MHz, CDCl_3_, 303 K) δ 8.17–8.11 (m, 1H, ArH), 7.59–7.52 (m, 1H, ArH), 6.91–6.81 (m, 7H, 7 × ArH), 6.74 (d, 1H, *J* = 8.3 Hz, ArH), 6.65–6.56 (m, 2H, 2 × ArH), 5.61–5.53 (m, 1H, CH), 5.01 (s, 1H, NH), 4.17 (dd, 1H, *J* = 5.3, 9.7 Hz, CH_2_), 4.05 (dd, 1H, *J* = 4.9, 9.9 Hz, C*H*H), 3.72 (dd, 2H, *J* = 5.1, 5.1 Hz, C*H*H), 3.68–3.60 (m, 4H, 2 × CH_2_), 3.56 (dd, 2H, *J* = 5.2, 5.2 Hz, CH_2_), 3.37–3.24 (m, 4H, 2 × CH_2_), 1.47 (d, 3H, *J* = 6.6 Hz, CH_3_), 1.44 (s, 9H, 3 × CH_3_).

Only one NH signal was observed in the ^1^H NMR spectrum.

^13^C NMR (125 MHz, CDCl_3_, 303 K) δ 163.4 (C=O), 156.1 (C), 154.5 (C), 152.6 (C), 149.5 (C), 146.9 (CH), 144.4 (C), 138.8 (CH), 120.3 (CH), 119.0 (CH), 116.9 (CH), 115.9 (CH), 114.3 (CH), 111.8 (CH), 71.4 (CH_2_), 70.4 (CH_2_), 69.9 (CH_2_), 69.5 (CH), 44.4 (CH_2_), 40.6 (CH_2_), 28.6 (CH_3_), 17.2 (CH_3_)

Twenty two ^13^C resonances are observed, this accounts for 28/31 carbon atoms. There is one missing quaternary C resonance of the Boc (low intensity, not observed) and two CH_2_ resonance due to coincidental overlap at 70.4 ppm HRMS (ES+): calcd. for C_31_H_42_N_3_O_7_ [M + H]^+^ 568.3023; found 568.3021 (−0.4 ppm).

##### *tert*-Butyl­(2-(2-(methyl­(4-(4-(2-(pyridin-2-yloxy)­propoxy)­phenoxy)­phenyl)­amino)­ethoxy)­ethyl) carbamate (**6**)

Following **General procedure D**, **2** (50 mg, 0.08 mmol) was reacted with *tert*-butyl *N*-[2-[2-(methylamino)­ethoxy]­ethyl]­carbamate (**3c**) (52 mg, 0.241 mmol), Cs_2_CO_3_ (53 mg, 0.16 mmol), RuPhos (1.5 mg, 0.003 mmol), Pd_2_(dba)_3_ (1.5 mg, 0.002 mmol) and DMF (0.4 mL). Purification by column chromatography (4 g silica, 0:100 → 50:50 EtOAc:Heptane) provided the title compound as a pale yellow oil (11 mg, 0.019 mmol, 24%).

^1^H NMR (500 MHz, CDCl_3_, 303 K) δ 8.14 (ddd, 1H, *J* = 1.4, 2.4, 3.9 Hz, ArH), 7.55 (ddd, 1H, *J* = 2.0, 7.1, 8.4 Hz, ArH), 6.91–6.83 (m, 7H, 7 × ArH), 6.75–6.72 (m, 1H, ArH), 6.72–6.68 (m, 2H, 2 × ArH), 5.60–5.53 (m, 1H, CH), 4.79 (s, 1H, NH), 4.16 (dd, 1H, *J* = 5.3, 9.9 Hz, C*H*H), 4.04 (dd, 1H, *J* = 4.9, 9.9 Hz, C*H*H), 3.62 (t, 2H, *J* = 5.8 Hz, CH_2_), 3.51–3.45 (m, 4H, 2 × CH_2_), 3.31–3.25 (m, 2H, CH_2_), 2.94 (s, 3H, CH_3_), 1.47 (d, 3H, *J* = 6.4 Hz, CH_3_), 1.44 (s, 9H, 3 × CH_3_).

^13^C NMR (126 MHz, CDCl_3_, 303 K) δ 163.4 (C=O), 156.1 (C), 154.5 (C), 152.6 (C), 148.9 (C), 147.0 (CH), 146.1 (C), 138.8 (CH), 120.1 (CH), 119.1 (CH), 116.9 (CH), 115.9 (CH), 113.9 (CH), 111.8 (CH), 79.4 (C), 71.4 (CH_2_), 70.4 (CH_2_), 69.5 (CH), 68.8 (CH_2_), 53.4 (CH_2_), 40.6 (CH_2_), 39.5 (CH_3_), 28.6 (CH_3_), 17.2 (CH_3_) HRMS (ES+): calcd. for C_30_H_40_N_3_O_6_ [M + H]^+^ 538.2917; found 538.2929 (2.2 ppm)

##### *tert*-Butyl (2-(2-(2-((3-(4-(4-(2-(pyridin-2-yloxy)­propoxy)­phenoxy)­phenyl)­prop-2-yn-1-yl)­oxy)­ethoxy)­ethoxy)­ethyl) carbamate (**7**)

Following **General procedure E**, **2** (50 mg, 0.08 mmol) was reacted with *tert*-butyl *N*-[2-[2-(2-prop-2-ynoxyethoxy)­ethoxy]­ethyl]­carbamate (**3d**) (46 mg, 0.16 mmol), CuI (3 mg, 0.016 mmol), Pd­(dppf)­Cl_2_ (2 mg, 0.03 mmol), *N*-methylmorpholine (17.6 μL, 0.16 mmol) and 1,4-dioxane (0.32 mL). Purification by column chromatography (4 g silica, 0:100 → 100:0 EtOAc:Heptane) provided the title compound as a yellow oil (20 mg, 0.033 mmol, 41%).

^1^H NMR (500 MHz, CDCl_3_, 303 K) δ 8.15 (ddd, 1H, *J* = 0.6, 2.0, 5.0 Hz, ArH), 7.56 (ddd, 1H, *J* = 2.0, 7.1, 8.4 Hz, ArH), 7.38–7.33 (m, 2H, 2 × ArH), 6.97–6.91 (m, 4H, 4 × ArH), 6.87–6.82 (m, 3H, 3 × ArH), 6.75–6.72 (m, 1H, ArH), 5.62–5.55 (m, 1H, CH), 5.01 (s, 1H, NH), 4.41 (s, 2H, CH_2_), 4.19 (dd, 1H, *J* = 5.3, 9.9 Hz, C*H*H), 4.07 (dd, 1H, *J* = 4.9, 9.9 Hz, C*H*H), 3.78–3.74 (m, 2H, CH_2_), 3.73–3.69 (m, 2H, CH_2_), 3.68–3.64 (m, 2H, CH_2_), 3.64–3.61 (m, 2H, CH_2_), 3.54 (t, 2H, *J* = 5.2 Hz, CH_2_), 3.34–3.27 (m, 2H, CH_2_), 1.48 (d, 3H, *J* = 6.4 Hz, CH_3_), 1.44 (s, 9H, 3 × CH_3_).

^13^C NMR (126 MHz, CDCl_3_, 303 K) δ 163.3 (C), 159.1 (C), 156.2 (C), 155.8 (C), 149.6 (C), 146.9 (CH), 138.8 (CH), 133.5 (CH), 121.3 (CH), 117.3 (CH), 116.9 (CH), 116.6 (C), 116.1 (CH), 111.8 (CH), 86.2 (C), 84.3 (C), 79.3 (C), 71.1 (CH_2_), 70.74 (CH_2_), 70.67 (CH_2_), 70.4 (CH_2_), 69.4 (CH), 69.3 (CH_2_), 59.4 (CH_2_), 40.6 (CH_2_), 28.6 (CH_3_), 17.2 (CH_3_).

One missing ^13^C resonance, accounted for due to overlap of two CH_2_ resonance at 70.4 ppm.

HRMS (ES+): calcd. for C_34_H_43_N_2_O_8_ [M + H]^+^ 607.3019; found 607.3032 (2.1 ppm).

##### *tert*-Butyl (2-(2-(2-((3-(4-(4-(2-(pyridin-2-yloxy)­propoxy)­phenoxy)­phenyl)­allyl)­oxy) ethoxy)­ethoxy)­ethyl) carbamate (**8**)

Procedure adapted from Berger et al. (2019).^18^ A 5 mL oven-dried glass tube, equipped with a Teflon coated magnetic stir bar, was charged with, **2** (50 mg, 0.08 mmol), Pd­(OAc)_2_ (1.62 mg, 0.0072 mmol), and PPh_3_ (3.16 mg,0.012 mmol). After adding DMF (0.401 mL), the vial was capped and purged by vacuum cycling (×3) with nitrogen gas, followed by the addition of NEt_3_ (33.5 μL, 0.241 mmol) and a solution of *tert*-butyl *N*-[2-[2-(2-allyloxyethoxy)­ethoxy]­ethyl]­carbamate (**3e**) (23.2 mg, 0.0802 mmol) in DMF (0.2 mL). The mixture was stirred at 100 °C for 24 h. The reaction mixture was concentrated under reduced pressure and the residue was purified by column chromatography (4 g silica, 0:100 → 100:0 EtOAc:Heptane) to provide a mixed fraction which was dissolved in 3:1 MeCN:water and further purified by preparative HPLC (10:90 → 100:0 MeCN:water (0.1% NH_3_)) to provide the trans-isomer of the title product as a pale yellow oil (6.42 mg, 0.01 mmol, 12.5%). Additional impure fractions containing the *trans-* (2.9 mg) and the *cis-* product (∼5 mg) were also collected, however were not sufficiently pure for characterization.

^1^H NMR (500 MHz, CDCl_3_, 303 K) δ 8.15 (dd, 1H, *J* = 1.8, 5.0 Hz, ArH), 7.56 (ddd, 1H, *J* = 1.9, 7.0, 8.4 Hz, ArH), 7.33–7.29 (m, 2H, 2 × ArH), 6.97–6.91 (m, 4H, 4 × ArH), 6.89–6.84 (m, 3H, 3 × ArH), 6.75–6.73 (m, 1H, ArH), 6.56 (d, 1H, *J* = 15.9 Hz, CH), 6.18 (td, 1H, *J* = 6.2, 15.9 Hz, CH), 5.62–5.55 (m, 1H, CH), 5.00 (s, 1H, NH), 4.21–4.16 (m, 3H,CH_2_, C*H*H), 4.07 (dd, 1H, *J* = 4.9, 9.9 Hz, C*H*H), 3.70–3.61 (m, 8H, 4 × CH_2_), 3.54 (dd, 2H, *J* = 5.2, 5.2 Hz, CH_2_), 3.34–3.28 (m, 2H, CH_2_), 1.48 (d, 3H, *J* = 6.4 Hz, CH_3_), 1.44 (s, 9H, 3 × CH_3_).

Coupling constant of 15.9 Hz for the alkene CH at 6.18 ppm is consistent with the *trans*-alkene

^13^C NMR (126 MHz, CDCl_3_, 303 K) δ 163.4 (C), 158.4 (C), 155.5 (C), 150.3 (C), 147.0 (CH), 138.8 (CH), 132.1 (CH, alkene), 131. Four (C), 127.9 (CH), 124.9 (CH, alkene), 120.9 (CH), 117.8 (CH), 116.9 (CH), 116.0 (CH), 111.8 (CH), 72.1 (CH_2_), 71.3 (CH_2_), 70.9 (CH_2_), 70.8 (CH_2_), 70.5 (CH_2_), 70.4 (CH_2_), 69.6 (CH), 69.5 (CH_2_), 40.6 (CH_2_), 28.6 (CH_3_), 17.2 (CH_3_).

Two ^13^C resonances missing, one is the Boc quaternary carbon resonance (low intensity), and the other a quaternary aromatic carbon resonance, either low intensity or coincidental overlap.

MS (ES+): *m*/*z* (%) 136 (70) [C_8_H_10_NO]^+^, 265 (60) [unknown fragment], 360.2 (100) [M-C_11_H_22_NO_5_]^+^, 631.3 (25) [M + Na]^+^.

HRMS (ES+): calcd. for C_34_H_45_N_2_O_8_ [M + H]^+^ 609.3176; found 609.3188 (2.0 ppm).

##### 5-(4-(2-Morpholino-4-oxo-4*H*-chromen-8-yl)­phenyl)-5*H*-thianthren-5-ium trifluoromethanesulfonate (**33**)

Following **General procedure A**, LY294002 (**9**) (325 mg, 1.06 mmol) was reacted with thianthrene-*S*-oxide (614 mg, 2.64 mmol), TfOH (187 μL, 2.11 mmol), TFAA (894 μL, 6.34 mmol) in MeCN (4 mL) overnight (approximately 18 h). Purification by column chromatography (C18 silica, 10:90–100:0 MeCN:water (0.1% formic acid)) provided the title compound (271 mg, 0.468 mmol, 45%) which was freeze-dried to afford a pale yellow solid.

^1^H NMR (400 MHz, MeOD, 303 K) δ 8.55 (d, *J* = 7.8 Hz, 2H, 2 × ArH), 8.13–8.04 (m, 3H, 3 × ArH), 7.97 (t, *J* = 7.6 Hz, 2H, 2 × ArH), 7.90 (t, *J* = 7.6 Hz, 2H, 2 × ArH), 7.73 (d, *J* = 8.4 Hz, 2H, 2 × ArH), 7.63 (dd, *J* = 7.6, 1.7 Hz, 1H, ArH), 7.47 (t, *J* = 7.7 Hz, 1H, ArH), 7.31 (d, *J* = 8.4 Hz, 2H, 2 × ArH), 5.59 (s, 1H, CH), 3.68 (t, *J* = 4.9 Hz, 4H, 2 × CH_2_), 3.35 (t, *J* = 5.0 Hz, 4H, 2 × CH_2_).

^19^F NMR (377 MHz, MeOD, 298 K) δ −80.12 (s) ^13^C NMR (101 MHz, MeOD, 303 K) δ 177.3 (C=O), 162.9 (C), 150.3 (C), 140.9 (C), 136.6 (C), 135.1 (CH), 135.0 (CH), 133.4 (CH), 131.3 (CH), 130.4 (CH), 129.8 (CH), 128.1 (C), 127.9 (CH), 125.4 (CH), 124.9 (CH), 124.2 (C), 122.7 (C), 119.3 (C), 85.8 (CH), 65.4 (CH_2_), 44.6 (CH_2_).

MS (ES+): *m*/*z* (%) 522 (100) [M]^+^, 261 (80) [M + H]^2+^.

HRMS (ES+): calcd. for C_31_H_24_O_3_NS_2_ [M + H]^+^ 522.1192; found 522.12032 (2.13 ppm)

Partial structural assignment to determine regioselectivity of the thianthrenation reaction can be found in the SI, Figure S2.

##### *tert*-Butyl (4-(2-morpholino-4-oxo-4*H*-chromen-8-yl)­benzyl)­carbamate (**9a**)

To a 5 mL glass vial equipped with a stir bar was added **33** (33 mg, 0.058 mmol), K_2_CO_3_ (24 mg, 0.174 mmol), potassium [[(*tert*-butoxycarbonyl)­amino]­methyl]­trifluoroborate (**34**) (16.5 mg, 0.0698 mmol) and S-phos Pd G4 (2.31 mg, 0.0029 mmol, 5 mol %). The reaction vessel was then sealed and placed under a nitrogen atmosphere (vacuum cycling × 3). 1,4-Dioxane (1 mL) and water (0.5 mL) were subsequently added, and the vessel sparged with nitrogen for 10 min. After this time the vial was heated to 80 °C overnight. The solution was cooled to room temperature, diluted with water (5 mL) and EtOAc (5 mL). The layers were separated and the aqueous phase extracted with EtOAc (5 mL). The combined organic phase was washed with brine (10 mL), dried over magnesium sulfate, filtered and reduced *in vacuo* onto silica gel. Purification by column chromatography (4 g silica, 0–20% EtOH in EtOAc) provided an oil that was dissolved in MeCN/water and freeze-dried to provide the title compound (19 mg, 0.0427 mmol, 73%) as a white solid.

^1^H NMR (400 MHz, CDCl_3_, 303 K) δ 8.21–8.15 (m, 1H, ArH), 7.55 (d, *J* = 7.4 Hz, 1H, ArH), 7.49 (d, *J* = 7.9 Hz, 2H, 2 × ArH), 7.43–7.35 (m, 3H, 3 × ArH), 5.51 (s, 1H, CH), 4.91 (s, 1H, NH), 4.40 (d, *J* = 6.1 Hz, 2H, CH_2_), 3.74 (t, *J* = 4.9 Hz, 4H, 2 × CH_2_), 3.35 (t, *J* = 4.9 Hz, 4H, 2 × CH_2_), 1.49 (s, 9H, 3 × CH_3_).

MS (ES+): *m*/*z* (%) 337 (100) [M+H–C_5_H_8_O_2_]^+^, 381 (40) [M+H–C_4_H_8_]^+^, 459 (10) [M + Na]^+^.

Analytical data are consistent with that reported in Bergamini et al. (2012).^42^

##### 8-(4-(Aminomethyl)­phenyl)-2-morpholino-4*H*-chromen-4-one dihydrochloride (CZC15098, **9b**)

To a round-bottom flask equipped with a stir bar was added **9a** (10 mg, 0.0229 mmol), DCM (0.5 mL) and 4 M HCl in dioxane (0.5 mL, 2 mmol). The reaction was stirred for 2 h at room temperature then reduced *in vacuo*. The residue was dissolved in MeCN/water and freeze-dried to provide the title compound (9.67 mg, 0.0227 mmol, 100%) as a white solid which was used without further purification.

^1^H NMR (400 MHz, DMSO-*d*_6_, 303 K) δ 8.47 (s, 2H, NH_2_), 7.98 (dd, *J* = 7.8, 1.7 Hz, 1H, ArH), 7.73–7.66 (m, 3H, 3 × ArH), 7.62 (d, *J* = 8.2 Hz, 2H, 2 × ArH), 7.49 (t, *J* = 7.6 Hz, 1H, ArH), 5.65 (s, 1H, CH), 4.10 (q, *J* = 5.9 Hz, 2H, CH_2_), 3.67 (t, *J* = 4.8 Hz, 4H, 2 × CH_2_), 3.38 (t, *J* = 4.9 Hz, 4H, 2 × CH_2_).

MS (ES+): *m*/*z* (%) 160 (100) [(M+H)-NH_3_]^2+^, 180 (20) [M+H+Na]^2+^, 320 (80) [M-NH_3_]^+^, 337 (90) [M + H]^+^, 359 (25) [M + Na]^+^, 695 (50) [2M+Na]^+^.

Analytical data are consistent with that reported in Bergamini et al. (2012).^42^

##### 5-(4-Methoxy-3-(6-(((1r,4r)-4-((2-methylpropyl)­sulfonamido)­cyclohexyl)­amino)-1*H*-pyrazolo­[3,4-*d*]­pyrimidin-3-yl)­phenyl)-5*H*-thianthren-5-ium formate (**S4**)

Following **General procedure A**, *N*-((1r,4r)-4-((3-(2-methoxyphenyl)-1H-pyrazolo­[3,4-*d*]­pyrimidin-6-yl)­amino)­cyclohexyl)-2-methylpropane-1-sulfonamide (**24**) (186 mg, 0.406 mmol) was reacted with thianthrene-*S*-oxide (104 mg, 0.446 mmol), HBF_4_·Et_2_O (110 μL, 0.811 mmol), TFAA (172 μL, 1.22 mol) and MeCN (6 mL). Purification by column chromatography (50 g C18, 5:95 → 95:5 MeCN:water (0.1% formic acid)) provided a yellow oil, which was suspended in MeCN (5 mL) and reduced *in vacuo* to provide the title compound as a white solid (190 mg, 0.25 mmol, 61.5%).

^1^H NMR (500 MHz, MeOD, 303 K) δ 8.69 (s, 1H, ArH), 8.54 (s, 1H, formic acid), 8.43 (dd, *J* = 8.0, 1.4 Hz, 2H, 2 × ArH), 8.01 (dd, *J* = 7.9, 1.3 Hz, 2H, 2 × ArH), 7.91 (td, *J* = 7.7, 1.4 Hz, 2H, 2 × ArH), 7.83 (td, *J* = 7.7, 1.4 Hz, 2H, 2 × ArH), 7.57 (dd, *J* = 2.0, 1.0 Hz, 1H, ArH), 7.33–7.30 (m, 2H, 2 × ArH), 3.95 (s, 3H, CH_3_), 3.82–3.75 (m, 1H, CH), 3.25–3.18 (m, 1H, CH), 2.94 (d, *J* = 6.4 Hz, 2H, CH_2_), 2.19 (septet, *J* = 6.7 Hz, 1H, CH), 2.14–2.02 (m, 4H, 2 × CH_2_), 1.50–1.36 (m, 4H, 2 × CH_2_), 1.09 (d, *J* = 6.8 Hz, 6H, 2 × CH_3_).

^13^C NMR (126 MHz, MeOD, 303 K) δ 170.0 (C), 161.8 (C), 161.7 (C), 158.7 (C), 156.7 (CH), 141.7 (C), 137.7 (C), 136.1 (CH), 135.6 (CH), 131.7 (CH), 131.6 (CH), 131.1 (CH), 130.7 (CH), 125.6 (C), 120.9 (C), 115.9 (C), 115.0 (CH), 107.3 (C), 62.1 (CH_2_), 56.9 (CH_3_), 53.3 (CH), 50.5 (CH), 34.3 (CH_2_), 32.3 (CH_2_), 26.2 (CH), 22.9 (CH_3_).

MS (ES+): *m*/*z* (%) 337 (100) [M + H]^2+^, 673 (100) [M]^+^.

HRMS (ES+): calcd. for C_34_H_37_N_6_O_3_S_3_ [M]^+^ 673.2089; found 673.2122 (4.9 ppm).

Partial structural assignment to determine regioselectivity of the thianthrenation reaction can be found in the SI, Table S7.

##### (*R*)-5-(1-(2-(4-Chlorophenoxy)­acetyl)-2-(methylcarbamoyl)­indolin-5-yl)-5*H*-thianthren-5-ium tetrafluoroborate (**S1**)

Following the **General procedure A**, (*R*)-1-(2-(4-chlorophenoxy)­acetyl)-*N*-methylindoline-2-carboxamide (**26**) (750 mg, 2.18 mmol) was reacted with thianthrene-*S*-oxide (556 mg, 2.39 mmol), HBF_4_·Et_2_O (444 μL, 3.26 mmol), TFAA (920 μL, 6.53 mmol) and MeCN (9 mL). Purification by column chromatography (40 g silica, 0:100 → 10:90 MeOH:DCM) provided the title compound as an off-white foam (1251 mg, 1.55 mmol, 71%) when dried under vacuum.

A small portion was further purifiedby preparative HPLC for data reporting (5:95 → 95:5 MeCN:water (0.1% formic acid)). NMR data is for the formate salt. Minor DMSO contamination.

^1^H NMR (500 MHz, CDCl_3_) δ 9.24 (s, 1H, NH), 8.71 (s, 1H, formic acid), 8.42 (t, *J* = 6.9 Hz, 2H, 2 × ArH), 7.98 (d, *J* = 8.3 Hz, 1H, ArH), 7.90–7.74 (m, 4H, 4 × ArH), 7.70 (td, *J* = 7.6, 1.5 Hz, 2H, 2 × ArH), 7.16–7.11 (m, 2H, 2 × ArH), 6.94 (d, *J* = 8.3 Hz, 1H, ArH), 6.92 (s, 1H, ArH), 6.90–6.85 (m, 2H, 2 × ArH), 5.67 (br s, 1H, CH), 5.05 (d, *J* = 15.9 Hz, 1H, C*H*H), 4.64 (d, *J* = 15.9 Hz, 1H, C*H*H), 3.68–3.53 (m, 1H, C*H*H), 3.12 (dd, *J* = 18.1, 3.2 Hz, 1H, C*H*H), 2.79 (d, *J* = 4.1 Hz, 3H, CH_3_).

^13^C NMR (126 MHz, CDCl3, 298 K) δ 171.3 (C=O), 168.4 (C=O), 167.9 (C=O), 156.7 (C–O), 148.2 (C), 136.5 (C), 136.3 (C), 135.0 (CH), 134.9­(CH), 134.6 (CH), 134.0 (C), 130.6 (CH), 130.5 (CH), 130.4 (CH), 130.3 (CH), 129.6 (CH), 129.0 (CH), 126.5 (C), 124.2 (CH), 119.3 (C), 119.2 (C), 118.2 (CH), 116.2 (CH), 116.0 (C), 66.8 (CH_2_), 60.5 (CH), 34.9 (CH_2_), 26.5 (CH_3_).

28/31 resonances observed in ^13^C NMR spectrum. Missing resonances accounted for by coincidental overlap of aromatic resonances at 116.2, 129.6, and 134.6 ppm. Resonances 136.5/136.3, 135.0/134.9, 130.6/130.5, 130.4, 130.3 and 119.3/119.2 appear to be possible doublets, these have been assigned as distinct peaks since splitting is likely caused by desymmetrization due to the stereo center.

MS (ES+): *m*/*z* (%) 559 (100) [M + H]^+^.

HRMS (ES+): calcd. for C_30_H_24_N_2_O_3_S_2_Cl [M]^+^ 559.0917; found 559.0922 (0.9 ppm).

Partial structural assignment to determine regioselectivity of the thianthrenation reaction can be found in the SI, Table S8.

##### *tert*-Butyl (*R*)-(2-(2-(2-((3-(1-(2-(4-chlorophenoxy)­acetyl)-2-(methylcarbamoyl)­indolin-5-yl)­prop-2-yn-1-yl)­oxy)­ethoxy)­ethoxy)­ethyl)­carbamate (**26a**)

Following **General procedure E**, (*R*)-5-(1-(2-(4-chlorophenoxy)­acetyl)-2-(methylcarbamoyl)­indolin-5-yl)-5*H*-thianthren-5-ium tetrafluoroborate (**S1**) (50 mg, 0.077 mmol) was reacted with *tert*-butyl *N*-[2-[2-(2-prop-2-ynoxyethoxy)­ethoxy]­ethyl]­carbamate (**3d**) (44 mg, 0.155 mmol), CuI (3 mg, 0.016 mmol), Pd­(dppf)­Cl_2_ (2 mg, 0.03 mmol), *N*-methylmorpholine (34 μL, 0.309 mmol) and 1,4-dioxane (0.32 mL). Purification by preparative HPLC (5:95 → 95:5 MeCN:water (0.1% formic acid)) provided the title compound as a yellow oil (26 mg, 0.039 mmol, 50%).

^1^H NMR (500 MHz, MeOD, 298 K) δ 8.18–8.03 (m, 1H, ArH), 7.29 (s, 2H, 2 × ArH), 7.29–7.24 (m, 2H, 2 × ArH), 6.98–6.91 (m, 2H, 2 × ArH), 5.12 (dd, *J* = 10.8, 2.9 Hz, 1H, CH), 4.92–4.85 (m, 1H, C*H*H partially overlapped with water peak), 4.61 (d, *J* = 15.3 Hz, 1H, C*H*H), 4.41 (s, 2H, CH_2_), 3.76–3.59 (m, 9H, 4 × CH_2_ & C*H*H), 3.50 (t, *J* = 5.6 Hz, 2H, CH_2_), 3.21 (t, *J* = 5.5 Hz, 2H, CH_2_), 3.18–3.12 (m, 1H, C*H*H), 2.74 (s, 3H, CH_3_), 1.43 (s, 9H, 3 × CH_3_).

^13^C NMR (126 MHz, MeOD, 298 K) δ 173.7 (C=O), 168.8 (C=O), 158.2 (C–O), 144.2 (C), 132.5 (CH), 130.4 (CH), 128.8 (CH), 127.5 (C), 120.1 (C), 118.3 (CH), 117.3 (CH), 87.0 (C), 85.6 (C), 80.1 (C), 71.6 (CH_2_), 71.4 (CH_2_), 71.2 (CH_2_), 71.1 (CH_2_), 70.2 (CH_2_), 68.3 (CH_2_), 62.1 (CH), 59.8 (CH_2_), 41.3 (CH_2_), 35.7 (CH_2_), 28.8 (CH_3_), 26.6 (CH_3_).

Twenty six ^13^C resonances are observed, 4 resonances are missing due to overlap. The two ^13^C resonances unaccounted for; a quaternary aromatic carbon, and Boc carbonyl, are missing either due to coincidental overlap or low intensity.

HRMS (ES+): calcd. for C_27_H_33_N_3_O_6_Cl [M + H]^+^ 530.2058; found 530.2063 (0.9 ppm).

##### *tert*-Butyl (*R*)-(2-(2-(2-((1-(2-(4-chlorophenoxy)­acetyl)-2-(methylcarbamoyl) indolin-5-yl)­amino)­ethoxy)­ethoxy)­ethyl)­carbamate (**26b**)

Following **General procedure C**, (*R*)-5-(1-(2-(4-chlorophenoxy)­acetyl)-2-(methylcarbamoyl)­indolin-5-yl)-5*H*-thianthren-5-ium tetrafluoroborate (**S1**) (200 mg, 0.309 mmol) was reacted with *tert*-butyl (2-(2-(2-aminoethoxy)­ethoxy)­ethyl)­carbamate (**3b**) (115 mg, 0.464 mmol), K_2_CO_3_ (85 mg, 0.618 mmol), [Cu­(MeCN)_4_]­BF_4_ (97 mg, 0.309 mmol), Ir­(ppy)_3_ (4 mg, 0.006 mmol) and MeCN (1.5 mL) using an Asynt Illumin8 photoreactor. Purification by preparative HPLC (5:95 → 95:5 MeCN:water (0.1% NH_3_)) provided the title compound as a white solid (109 mg, 0.184 mmol, 59%).

^1^H NMR (500 MHz, DMSO-*d*_6_, 298 K) δ 8.19–8.15 (m, 1H, NH), 7.75 (d, *J* = 8.6 Hz, 1H, ArH), 7.33–7.30 (m, 2H, 2 × ArH), 6.96–6.92 (m, 2H, 2 × ArH), 6.76–6.71 (m, 1H, NH), 6.51–6.47 (m, 1H, ArH), 6.42–6.36 (m, 1H, ArH), 5.36 (t, *J* = 5.9 Hz, 1H, NH), 4.99 (dd, *J* = 10.6, 2.6 Hz, 1H, CH), 4.90 (d, *J* = 15.3 Hz, 1H, CH_2_), 4.43 (d, *J* = 15.1 Hz, 1H, CH_2_), 3.55–3.49 (m, 7H, 4 × CH_2_), 3.38 (t, *J* = 6.2 Hz, 2H, CH_2_), 3.15 (q, *J* = 5.9 Hz, 2H, CH_2_), 3.08–3.04 (m, 2H, CH_2_), 3.01–2.95 (m, 1H, CH_2_), 2.62 (d, *J* = 4.5 Hz, 3H, CH_3_), 1.37 (s, 9H, 3 × CH_3_).

^13^C NMR (126 MHz, DMSO, 298 K) δ 171.2 (C=O), 164.3 (C=O), 157.0 (C), 155.6 (C=O), 145.9 (C), 133.0 (C), 130.6 (C), 129.0 (CH), 124.5 (C), 117.1 (CH), 116.3 (CH), 110.2 (CH), 108.1 (CH), 77.6 (C), 69.6 (CH_2_), 69.5 (CH_2_), 69.2 (CH_2_), 69.0 (CH_2_), 66.0 (CH_2_), 59.7 (CH), 43.1 (CH_2_), 39.44 (CH_2_, overlapped with DMSO-*d*_6_ residual solvent peak, identified by HSQC), 34.7 (CH_2_), 28.2 (CH_3_), 25.9 (CH_3_).

MS (ES+): *m*/*z* (%) 491 (70) [M+H–C_5_H_9_O_2_]^+^, 591 (100) [M + H]^+^, 613 (30) [M + Na]^+^.

HRMS (ES+): calcd. for C_29_H_40_N_4_O_7_Cl [M + H]^+^ 591.2586; found 591.2610 (4.1 ppm).

##### 4-(4-((5-Amino-4-((3-methoxy-4-(5*H*-thianthren-5-ium-5-yl)­phenyl)­carbamoyl)-1*H*-1,2,3-triazol-1-yl)­methyl)­benzyl)­morpholin-4-ium diformate (**S2**)

Following the **General procedure A** 5-amino-*N*-(3-methoxyphenyl)-1-(4-(morpholinomethyl)­benzyl)-1*H*-1,2,3-triazole-4-carboxamide (**27**) (200 mg, 0.473 mmol) was reacted with thianthrene-*S*-oxide (121 mg, 0.521 mmol), BF_3_·Et_2_O (117 μL, 0.947 mmol), TFAA (200 μL, 1.42 mmol) and MeCN (4.4 mL). Workup was omitted, the material was quenched with MeOH and reduced *in vacuo* and the residue dissolved in EtOH (4 mL) followed by the addition of HCl (12 N, aq, 1 mL). The reaction was then heated to reflux overnight. After this time the reaction was neutralized with saturated sodium bicarbonate (aq, 50 mL) and diluted with DCM (50 mL). The DCM layer was washed with water (50 mL), dried over sodium sulfate, filtered and reduced *in vacuo*. Purification by mass-directed preparative HPLC (5:95 → 95:5 MeCN:water (0.1% formic acid)) provided the title compound as a white solid (164 mg, 0.215 mmol, 45%).

^1^H NMR (500 MHz, MeOD, 303 K) δ 8.50 (s, 2H, formic acid), 8.25 (dd, *J* = 8.0, 1.4 Hz, 2H, 2 × ArH), 7.93 (dd, *J* = 7.9, 1.3 Hz, 2H, 2 × ArH), 7.86–7.80 (m, 3H, 3 × ArH), 7.76 (td, *J* = 7.7, 1.4 Hz, 2H, 2 × ArH), 7.37–7.30 (m, 3H, 3 × ArH), 7.27 (d, *J* = 7.8 Hz, 2H, 2 × ArH), 6.62 (d, *J* = 9.0 Hz, 1H, ArH), 5.42 (s, 2H, CH_2_), 3.91 (s, 3H, CH_3_),3.67–3.61 (m, 6H, 3 × CH_2_), 2.58–2.53 (m, 4H, 2 × CH_2_).

^13^C NMR (126 MHz, MeOD, 303 K) δ 169.4 (C), 162.6 (C), 159.9 (C), 147.4 (C), 147.3 (C), 138.1 (C), 136.2 (C), 136.1 (C), 135.7 (CH), 135.5 (CH), 131.6 (CH), 131.5 (CH), 131.1 (CH), 130.8 (CH), 128.8 (CH), 123.1 (C), 119.3 (C), 113.4 (CH), 105.3 (CH), 102.4 (C), 66.8 (CH_2_), 62.9 (CH_2_), 57.3 (CH_3_), 53.9 (CH_2_), 50.0 (CH_2_).

MS (ES+): *m*/*z* (%) 319 (100) [M + H]^2+^, 637 (50) [M]^+^.

HRMS (ES+): calcd. for C_34_H_33_N_6_O_3_S_2_ [M]^+^ 637.2056; found 637.2104 (7.5 ppm).

Partial structural assignment to determine regioselectivity of the thianthrenation reaction can be found in the SI, Figure S3.

##### *tert*-Butyl (2-(2-(2-((3-(4-(5-amino-1-(4-(morpholinomethyl)­benzyl)-1*H*-1,2,3-triazole-4-carboxamido)-2-methoxyphenyl)­prop-2-yn-1-yl)­oxy)­ethoxy)­ethoxy) ethyl)­carbamate (**27a**)

Following the **General procedure E**, 4-(4-((5-amino-4-((3-methoxy-4-(5*H*-thianthren-5-ium-5-yl)­phenyl)­carbamoyl)-1*H*-1,2,3-triazol-1-yl)­methyl)­benzyl)­morpholin-4-ium diformate (**S2**) (150 mg, 0.207 mmol) was reacted with *tert*-butyl *N*-[2-[2-(2-prop-2-ynoxyethoxy)­ethoxy]­ethyl]­carbamate (**3d**) (119 mg, 0.414 mmol), Pd­(dppf)­Cl_2_ (5 mg, 0.006 mmol), CuI (8 mg, 0.041 mmol), *N*-methylmorpholine (91 μL, 0.823 mmol) and 1,4-dioxane (1 mL). Purification by mass-directed preparative HPLC (5:95 → 95:5 MeCN:water (0.1% NH_3_)) provided the title compound as a colorless oil (68 mg, 0.091 mmol, 44%).

^1^H NMR (400 MHz, MeOD, 303 K) δ 7.57–7.52 (m, 1H, ArH), 7.40–7.34 (m, 2H, 2 × ArH), 7.32–7.19 (m, 4H, 4 × ArH), 5.42 (s, 2H, CH_2_), 4.43 (s, 2H, CH_2_), 3.89–3.83 (m, 3H, CH_3_), 3.79–3.74 (m, 2H, CH_2_), 3.72–3.64 (m, 8H, 4 × CH_2_), 3.62–3.59 (m, 4H, 2 × CH_2_), 3.50 (t, *J* = 5.5 Hz, 2H, CH_2_), 3.21 (t, *J* = 5.5 Hz, 2H, CH_2_), 2.60–2.48 (m, 4H, 2 × CH_2_), 1.42 (s, 9H, 3 × CH_3_).

^13^C NMR (101 MHz, MeOD, 303 K) δ 161.2 (C), 160.7 (C), 157.0 (C), 145.5 (C), 140.1 (C), 136.5 (C), 134.3 (C), 133.3 (CH), 129.9 (CH), 127.2 (CH), 122.2 (C), 111.4 (CH), 106.8 (C), 102.6 (CH), 87.9 (C), 82.4 (C), 78.7 (C), 70.1 (CH_2_), 70.0 (CH_2_), 69.9 (CH_2_), 69.7 (CH_2_), 68.6 (CH_2_), 66.1 (CH_2_), 62.3 (CH_2_), 58.6 (CH_2_), 54.9 (CH_3_), 53.1 (CH_2_), 48.7 (CH_2_), 39.9 (CH_2_), 27.4 (CH_3_).

MS (ES+): *m*/*z* (%) 477 (80) [M+H–C_11_H_22_NO_4_]^+^, 708 (100) [M + H]^+^, 731 (40) [M + Na]^+^.

HRMS (ES+): calcd. for C_36_H_50_O_8_N_7_ [M + H]^+^ 708.37629; found 708.37316 (2.29 ppm).

##### 4-(4-((5-Amino-4-(3-(2-methoxy-5-(5*H*-thianthren-5-ium-5-yl)­phenyl)-1,2,4-oxadiazol-5-yl)-1*H*-1,2,3-triazol-1-yl)­methyl)­benzyl)­morpholin-4-ium diformate (**S3**)

Following the **General procedure A**, 4-(3-(2-methoxyphenyl)-1,2,4-oxadiazol-5-yl)-1-(4-(morpholinomethyl)­benzyl)-1*H*-1,2,3-triazol-5-amine (**29**) (392 mg, 0.876 mmol) was reacted with thianthrene-*S*-oxide (224 mg, 0.964 mmol), HBF_4_·Et_2_O (238 μL, 1.75 mmol), TFAA (370 μL, 2.63 mmol) and MeCN (5 mL). Workup was omitted, the material was quenched with MeOH and reduced *in vacuo*, as washing with 5% NaHBF_4_ will protonate the product and it will be drawn into the aqueous phase. The residue was purified by reverse phase chromatography (43 g C18, 5:95 → 95:5 MeCN:water (0.1% formic acid)) to provide the title compound and a mixed fraction containing the title compound and a trifluoroacetylated byproduct (300 mg). This byproduct-containing fraction was dissolved in EtOH (5 mL), HCl (12 N, 0.5 mL) was added and the reaction heated to reflux for 2 h. After this time LCMS indicated complete consumption of the trifluoroacetylated byproduct and formation of the desired product. Purification by reverse-phase chromatography (24 g C18, 10:90 → 100:0 MeCN:water (0.1% formic acid)) provided the title compound as a white solid, which was combined with the material previously isolated (440 mg, 0.558 mmol, 64%).

^1^H NMR (500 MHz, Acetone-*d*_6_, 303 K) δ 8.68 (dd, *J* = 8.0, 1.4 Hz, 2H, 2 × ArH), 8.57 (s, 2H, formic acid), 8.10–8.06 (m, 2H, 2 × ArH), 8.03–7.99 (m, 3H, 3 × ArH), 7.92 (td, *J* = 7.7, 1.4 Hz, 2H, 2 × ArH), 7.53 (dd, *J* = 9.1, 2.7 Hz, 1H, ArH), 7.44–7.40 (m, 2H, 2 × ArH), 7.34 (d, *J* = 9.1 Hz, 1H, ArH), 7.31 (m, 2H, 2 × ArH), 5.66 (s, 2H, CH_2_), 3.98 (s, 3H, CH_3_), 3.57 (t, *J* = 4.7 Hz, 4H, 2 × CH_2_), 3.43 (s, 2H, CH_2_), 2.36–2.32 (m, 4H, 2 × CH_2_).

No NH peaks were visible in the 1H NMR spectrum.

^13^C NMR (126 MHz, Acetone-*d*_6_, 303 K) δ 169.5 (C), 166.7 (C), 166.6 (C), 164.9 (C), 162.5 (C), 146.2 (C), 139.0 (C), 136.9 (C), 136.2 (CH), 136.0 (CH), 135.3 (C), 133.4 (CH), 132.1 (CH), 131.3 (CH), 131.0 (CH), 130.1 (CH), 128.9 (CH), 120.1 (C), 118.7 (C), 115.8 (C), 115.6 (C), 115.4 (CH), 67.4 (CH_2_), 63.4 (CH_2_), 57.3 (CH_3_), 54.4 (CH_2_), 49.9 (CH_2_).

MS (ES+): *m*/*z* (%) 332 (100) [M + H]^2+^, 662 (20) [M]^+^.

HRMS (ES+): calcd. for C_35_H_32_N_7_O_3_S_2_ [M]^+^ 662.2008; found 662.2040 (4.8 ppm).

Partial structural assignment to determine regioselectivity of the thianthrenation reaction can be found in the SI, Figure S4.

##### 4-(4-((5-Amino-4-(3-(5-(2,2-dimethyl-4-oxo-3,8,11,14-tetraoxa-5-azaheptadec-16-yn-17-yl)-2-methoxyphenyl)-1,2,4-oxadiazol-5-yl)-1*H*-1,2,3-triazol-1-yl)­methyl)­benzyl) morpholin-4-ium formate (**29a**)

Following the **General Procedure E**, 5-(3-(5-(5-amino-1-(4-(morpholinomethyl)­benzyl)-1*H*-1,2,3-triazol-4-yl)-1,2,4-oxadiazol-3-yl)-4-methoxyphenyl)-5*H*-thianthren-5-ium tetrafluoroborate (**S3**) (60 mg, 0.080 mmol) was reacted with *tert*-butyl *N*-[2-[2-(2-prop-2-ynoxyethoxy)­ethoxy]­ethyl]­carbamate (**3d**) (46 mg, 0.160 mmol), Pd­(dppf)­Cl_2_ (2 mg, 0.002 mmol), CuI (3 mg, 0.016 mmol), *N*-methylmorpholine (35 μL, 0.321 mmol) and 1,4-dioxane (0.32 mL). Purification by preparative HPLC (5:95 → 95:5 MeCN:water (0.1% formic acid)) provided the title compound as a yellow oil (7 mg, 0.009 mmol, 10%).

^1^H NMR (500 MHz, CDCl_3_) δ 8.12 (d, *J* = 2.2 Hz, 1H, ArH), 7.54 (dd, *J* = 8.6, 2.2 Hz, 1H, ArH), 7.38 (d, *J* = 8.0 Hz, 2H, 2 × ArH), 7.26 (d, *J* = 2.3 Hz, 2H, overlapped with CDCl_3_ residual peak, 2 × ArH), 7.00 (d, *J* = 8.7 Hz, 1H, ArH), 5.49 (s, 2H, CH_2_), 5.12 (s, 2H, NH_2_), 5.05 (s, 1H, NH), 4.42 (s, 2H, CH_2_), 3.99 (s, 3H, CH_3_), 3.78–3.75 (m, 2H, CH_2_), 3.73–3.69 (m, 6H, 3 × CH_2_), 3.67–3.60 (m, 4H, 2 × CH_2_), 3.54 (t, *J* = 5.1 Hz, 2H, CH_2_), 3.50 (s, 2H, CH_2_), 3.30 (q, *J* = 5.4 Hz, 2H, CH_2_), 2.43 (d, *J* = 4.8 Hz, 4H, 2 × CH_2_), 1.43 (s, 9H, 3 × CH_3_).

^13^C NMR (126 MHz, CDCl_3_) δ 168.2 (C=O), 165.4 (C), 158.3 (C), 143.6 (C), 139.0 (C, observed via HMBC), 135.7 (CH), 134.7 (CH), 132.0 (C), 130.2 (CH), 127.5 (CH), 117.6 (C), 116.0 (C), 115.0 (C), 111.8 (CH), 85.4 (C), 84.5 (C), 79.2 ppm (C, observed via HMBC) 70.6 (CH_2_), 70.5 (CH_2_), 70.3 (CH_2_), 70.2 (CH_2_), 69.2 (CH_2_), 66.9 (CH_2_), 62.8 (CH_2_), 59.3 (CH_2_), 56.2 (CH_3_), 53.6 (CH_2_), 50.6 (CH_2_), 40.4 (CH_2_), 28.4 (CH_3_).

Several quaternary ^13^C signals were missing from the ^13^C spectrum due to low signal-to-noise but were observed via HMBC spectrum.

MS (ES+): *m*/*z* (%) 733.1 (100) [M + H]^+^.

HRMS (ES+): calcd. for C_37_H_49_N_8_O_8_ [M + H]^+^ 733.3673; found 733.371 (5.0 ppm).

## Supplementary Material





## Notes

Molecular strings file (CSV)
Supporting tables: Pyriproxyphen cross coupling reactions, high-throughput chemistry optimization, functionalized probe cross coupling reactions, *L. donovani* PI3/4K kinases; supporting proteomics figures: enrichment plots for additional replicates; partial NMR assignment of thianthrene salts **33**, **S1–4**,; additional experimental protocols: high-throughput chemistry, drug sensitivity assays, affinity pulldown, quantitative proteomics sample preparation, data acquisition and data analysis; ^1^H, ^13^C, ^19^F NMR and LCMS spectra (PDF)

## Abbreviations Used

CuTC: copper­(I) thiphene-2-carboxylate
dba: dibenzylideneacetone
dF­(CF_3_)­ppy: 2-(2,4-difluorophenyl)-5-(trifluoromethyl)­pyridine
dppf: bis­(diphenylphosphino)­ferrocene
dtbbpy: 4,4′-ditertbutyl-2,2′-bipyridine
HTE: high-throughput experimentation
LCMS/MS: liquid chromatography-tandem mass spectrometry
*Ld*CRK12: *Leishmania donovani* Cdc-2-related kinase
LED: light emitting diode
LSF: late stage functionalization
MeCN: acetonitrile
mTOR: mechanistic target of rapamycin
NMM: *N*-methylmorpholine
ppy: 2-phenylpyridyl
PROTAC: proteolysis targeting chimera
Q-TOF: quadropole time-of-flight
RIO: right open reading frame
SD: standard deviation
Tf_2_O: trifluoromethanesulfonic anhydride
TfOH: trifluoromethanesulfonic acid
TTO: thianthrene-*S*-oxide.
