# Supplementary material for: Late-Stage Functionalization via Thianthrenation: A Reaction Enabling Rapid Preparation of Drug Beads for Chemical Pulldown
Source: J Med Chem. 2026 Jul 21;69(15):19230–47. doi: 10.1021/acs.jmedchem.6c01571 (PMC13492373; doi:10.1021/acs.jmedchem.6c01571)
Supplement: Supplementary file 1 [file jm6c01571_si_002.pdf]

Supporting Information

Late-Stage Functionalization via Thianthrenation: A Reaction Enabling Rapid Preparation of Drug Beads for Chemical Pulldown

Robert J. Smith, Laurine Brouck, Chloe Pelletier, Sandra Carvalho, Gustavo Bravo Ruiz, Victoriano Corpas-Lopez, Gourav Dey, Stephen Patterson* and Susan Wyllie*

Drug Discovery Unit, Division of Drug Discovery, Faculty of Life Sciences, University of Dundee, Dow Street, Dundee DD1 5EH, United Kingdom

*Corresponding authors: Susan Wyllie (s.wyllie@dundee.ac.uk), Stephen Patterson (s.z.patterson@dundee.ac.uk)

Contents

Table S1. Screen of conditions to miniaturize the thianthrenation reaction. S3

Table S2. Thianthrenation substrate test plate. S4

Table S3. Preparation of functionalized probes based on 26. S5

Table S4. Preparation of functionalized probes based on 27. S5

Table S5. Preparation of functionalized probes based on 29. S6

Table S6. PI3/4/5K and PIKK proteins in *L. donovani* identified by InterPro domain and GO term searches S6

Figure S1. LY294002 affinity pulldown (replicates 2 and 3). S8

NMR assignment of 33 S8

NMR assignment of S4 S9

NMR assignment of S1 S10

NMR Assignment of S2 S11

NMR Assignment of S3 S12

Additional experimental procedures S14

  High-throughput experimentation (HTE) procedures S14

  Drug bead synthesis S16

Biological, chemical pulldown and proteomics protocols S17

  Drug sensitivity assays in *L. donovani* S17

  Affinity pulldown protocol S17

  Sample preparation and analysis by LC-MS/MS S17

  Data processing and enrichment analysis S18

NMR and LCMS spectra S19

References S60

Table S1. Screen of conditions to miniaturize the thianthrenation reaction. Reactions conducted in MeCN. after 3 h reactions quenched with 1:1 MeCN/water and analysed by LCMS, conversion estimated based on peak area. ND: product ion not detected.

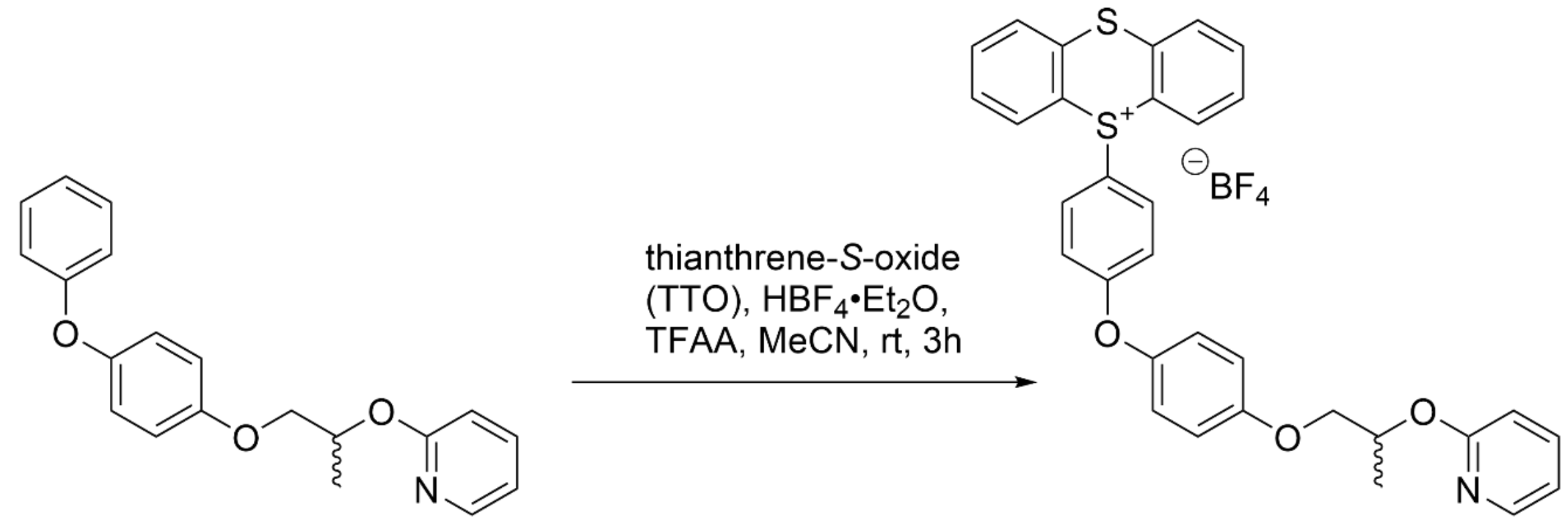

Entry	[Pyriproxyphen]/mM	[TTO]/mM	[HBF_4•Et_2O]/mM	Eq. HBF_4•Et_2O	[TFAA]/mM	Eq. TFAA	Conversion
**1**	10	10	10	1	30	1	ND
**2**	10	10	20	2	30	1	ND
**3**	10	10	50	5	120	12	>95%
**4**	5	5	25	5	60	12	ND
**5**	5	5	50	10	60	12	<5%
**6**	5	5	100	20	60	12	<20%
**7**	5	5	100	20	120	24	>95%
**8**	1.25	1.25	100	80	120	96	>95%
**9**	1.25	1.25	50	40	120	96	<10%

Table S2. Thianthrenation substrate test plate. 2.5 mM Ar-H, 2.5 mM TTO, 200 mM acid, 240 mM TFAA, MeCN, 3 h.Y = yes, N = no. [a]Incomplete conversion, as indicated by residual TTO in LCMS [b] multiple unidentified by-products in addition to desired product *m/z*

**Entry**	**Structure**	**Product *m/z* detected (Y/N)**		
		**HBF_4•Et_2O**	**TfOH**	**BF_3•Et_2O**
**1**	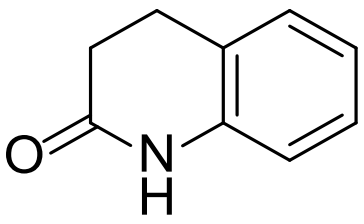	Y	Y[a]	Y
**2**	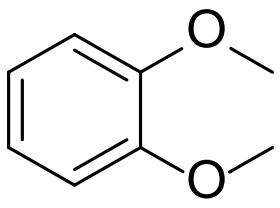	Y	Y[a]	Y
**3**	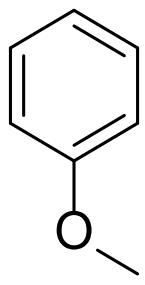	Y	Y[a]	Y
**4**	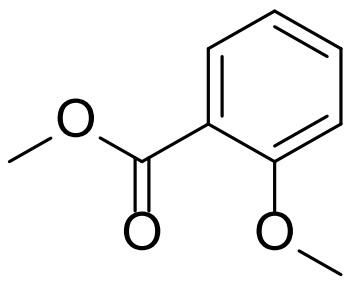	Y	Y	Y
**5**	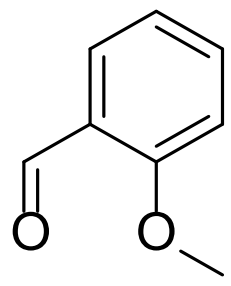	Y	Y[b]	Y
**6**	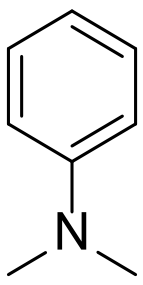	trace	trace	Y[a]
**7**	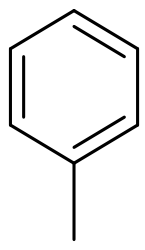	N	Y[a]	Y[a]
**8**	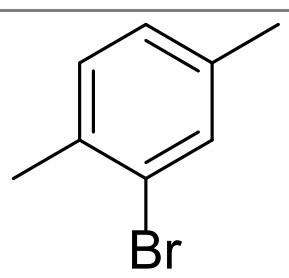	N	N	N
**9**	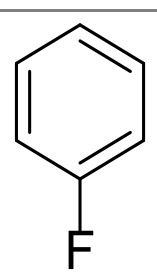	N	N	N
**10**	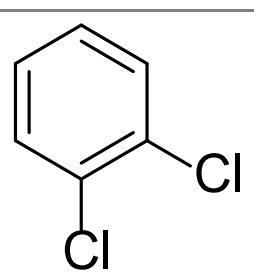	N	N	N
**11**	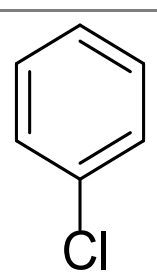	N	N	N

Table S3. Preparation of functionalized probes based on 26.
[a][Ir(dF(CF_3)ppy)$_2$(dtbbpy)]PF_6, CuTC, Na_2CO_3, MeCN, molecular sieves, 440nm LED, rt, 16hr, ; [b]Pd_2(dba)$_3$, RuPhos, Cs_2CO_3, DMF, 90 °C, 18h;; [c]Pd(dppf)Cl_2, CuI, NMM, dioxane, 40 °C, 48h [d]Ir(ppy)$_3$, [Cu(MeCN)$_4$]BF_4, K_2CO_3, MeCN, 440 nm LED, rt, 18h

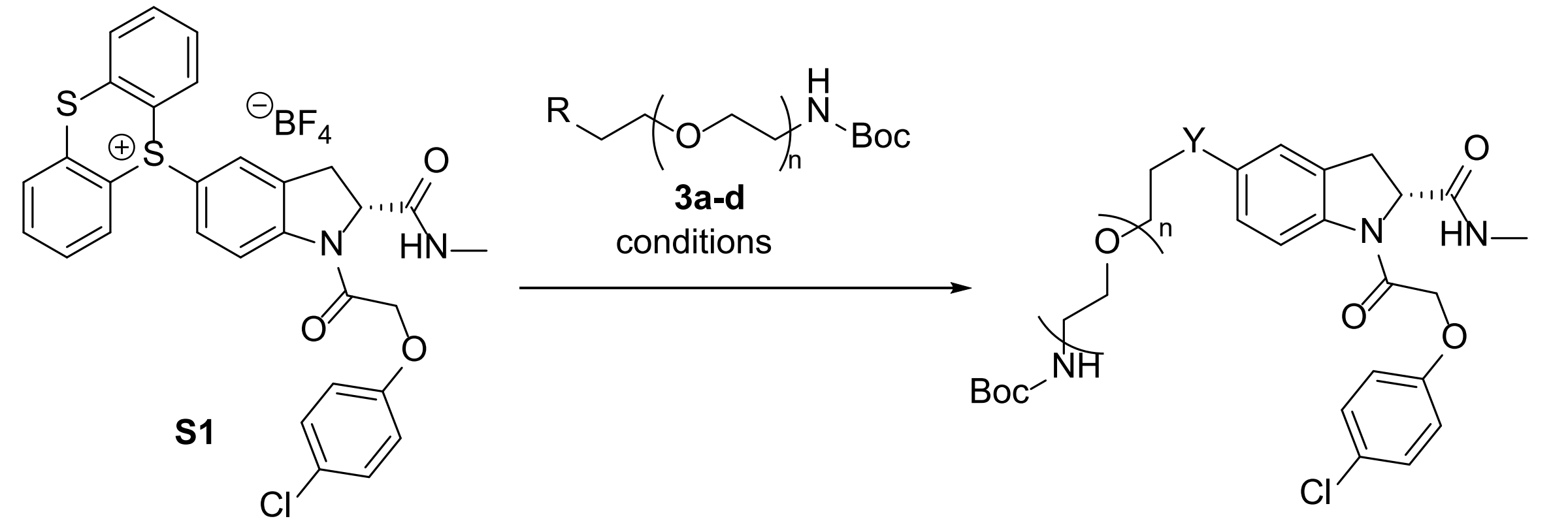

Product	X=	R=	n=	Y=	Yield (%)
-	F	HO-§-	2	O	0[a]
-	H	HN(Me)-§-	1	NMe	0[b]
26a	H	HC≡C–CH$_2$–O-§-	2	Ar-§-C≡C–CH$_2$–O-§-	53[c]
26b	F	H_2N-§-	2	NH	7[d]

Table S4. Preparation of functionalized probes based on 27.
[a][Ir(dF(CF_3)ppy)$_2$(dtbbpy)]PF_6, CuTC, Na_2CO_3, MeCN, molecular sieves, 440nm LED, rt, 16hr, ; [b]Pd_2(dba)$_3$, RuPhos, Cs_2CO_3, DMF, 90 °C, 18h; [c]Ir(ppy)$_3$, [Cu(MeCN)$_4$]BF_4, K_2CO_3, MeCN, 440 nm LED, rt, 18h; [d]Pd(dppf)Cl_2, CuI, NMM, dioxane, 40 °C, 48h

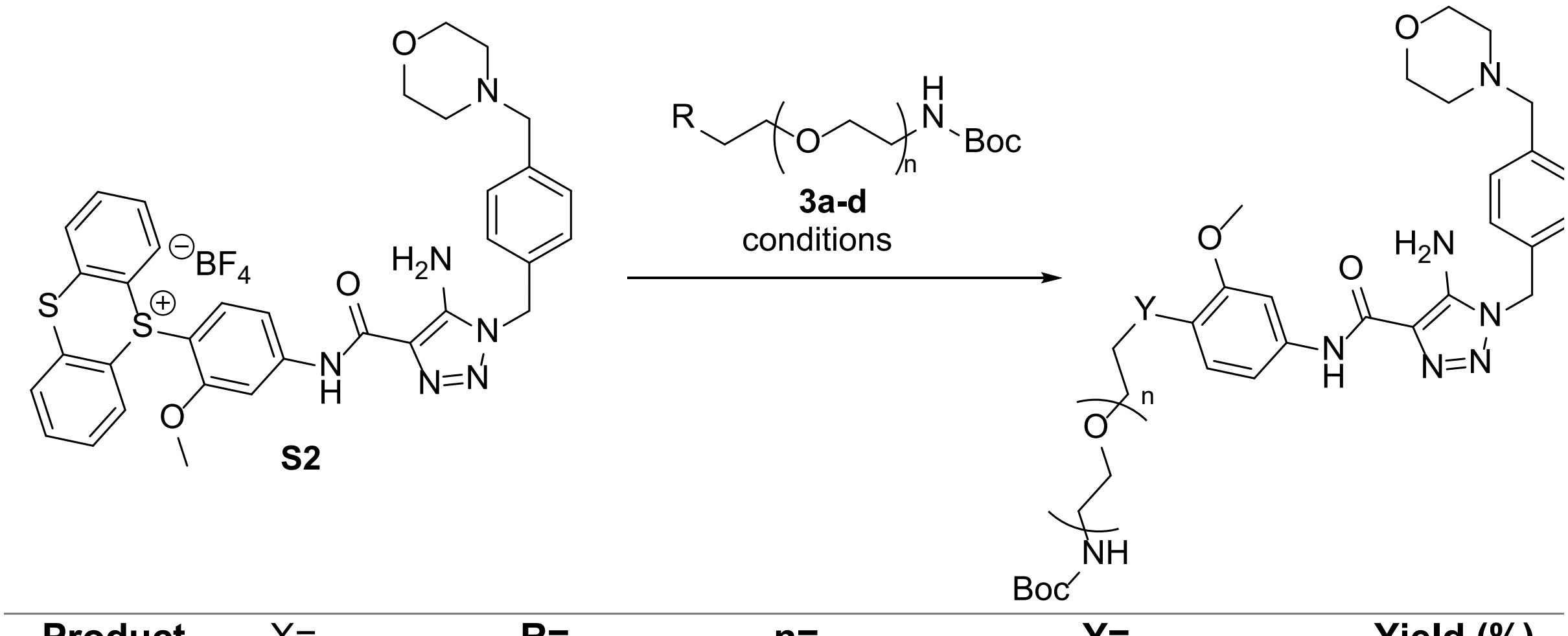

Product	X=	R=	n=	Y=	Yield (%)
-	F	HO-§-	2	O	0[a]
-	H	HN(Me)-§-	1	NMe	0[b]
-	F	H_2N-§-	2	NH	0[c]

**27a**	H	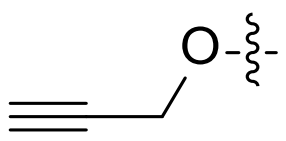	2	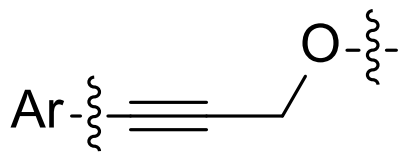	44[d]

Table S5. Preparation of functionalized probes based on 29.
[a][Ir(dF(CF$_3$)ppy)$_2$(dtbbpy)]PF$_6$, CuTC, Na$_2$CO$_3$, MeCN, molecular sieves, 440nm LED, rt, 16hr, ; [b]Pd$_2$(dba)$_3$, RuPhos, Cs$_2$CO$_3$, DMF, 90 °C, 18h; [c]Ir(ppy)$_3$, [Cu(MeCN)$_4$]BF$_4$, K$_2$CO$_3$, MeCN, 440 nm LED, rt, 18h; [d]Pd(dppf)Cl$_2$, CuI, NMM, dioxane, 40 °C, 48h

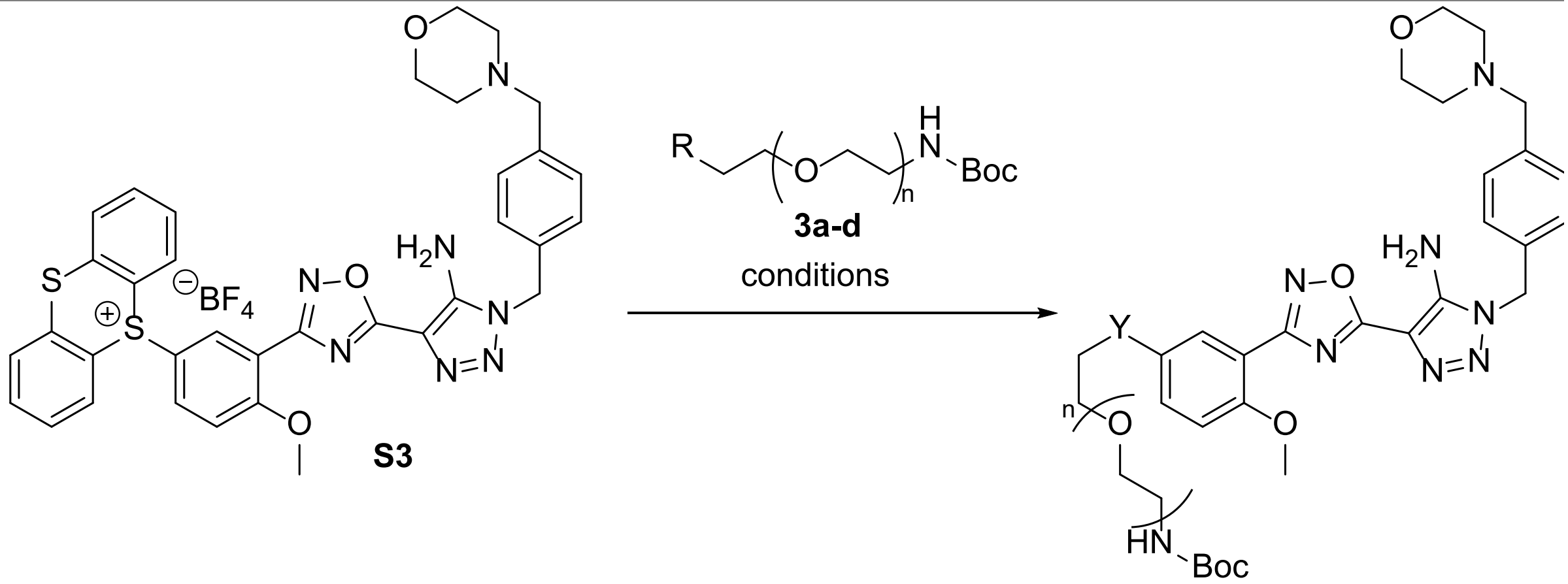

Product	X=	R=	n=	Y=	Yield (%)
-	F	HO-	2	O	0[a]
-	H	HN(Me)-	1	NMe	0[b]
-	F	H$_2$N-	2	NH	0[c]
**29a**	H	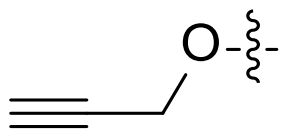	2	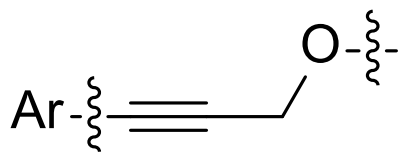	12[d]

Table S6. PI3/4/5K and PIKK proteins in *L. donovani* identified by InterPro domain and GO term searches

Protein name	Protein ID	InterPro domain	GO term	Type
Phosphatidylinositol kinase, putative; FYVE zinc finger family protein	E9BLS8; E9BLS7	IPR000403	GO:0052742	PI3/4K
1-phosphatidylinositol 4-kinase	E9BKV3	IPR000403	GO:0052742	PI3/4K
Phosphatidylinositol 3-kinase	A0A3Q8IGA3	IPR000403	GO:0052742	PI3/4K
Phosphatidylinositol 3-and 4-kinase family protein	A0A3S7X8H7	IPR000403	GO:0052742	PI3/4K
PI3-kinase family, ras-binding domain/ phosphoinositide 3-kinase C2/ phosphoinositide 3-kinase family, accessory	E9BBN7	IPR000403	GO:0052742	PI3/4K

domain /phosphatidylinositol 3- and 4-kinase, putative				
Phosphatidylinositol-4-phosphate 5-kinase family protein	A0A3S7XA92	IPR002498	GO:0052742	PI5K
Phosphatidylinositol 4-phosphate 5-kinase alpha, putative	A0A504WXZ4	IPR002498	GO:0052742	PI5K
Phosphatidylinositol-4-phosphate 5-kinase-like, putative	E9BRT3	IPR002498	GO:0052742	PI5K
Phosphatidylinositol (3,5) kinase, putative;	E9BJ09	IPR002498	GO:0052742	PI5K
PI3K/PI4K catalytic domain-containing protein	E9BJD6	IPR000403		PIKK
non-specific serine/threonine protein kinase	E9BU18	IPR000403		PIKK
non-specific serine/threonine protein kinase	E9BRH3	IPR000403		PIKK
non-specific serine/threonine protein kinase	E9B7F7	IPR000403		PIKK
non-specific serine/threonine protein kinase	E9BER3	IPR000403		PIKK
Serine/threonine-protein kinase ATR	E9BNQ5	IPR000403		PIKK
Serine/threonine-protein kinase TOR	E9BRL5	IPR000403		PIKK
Serine/threonine-protein kinase TOR	E9BV14	IPR000403		PIKK

A

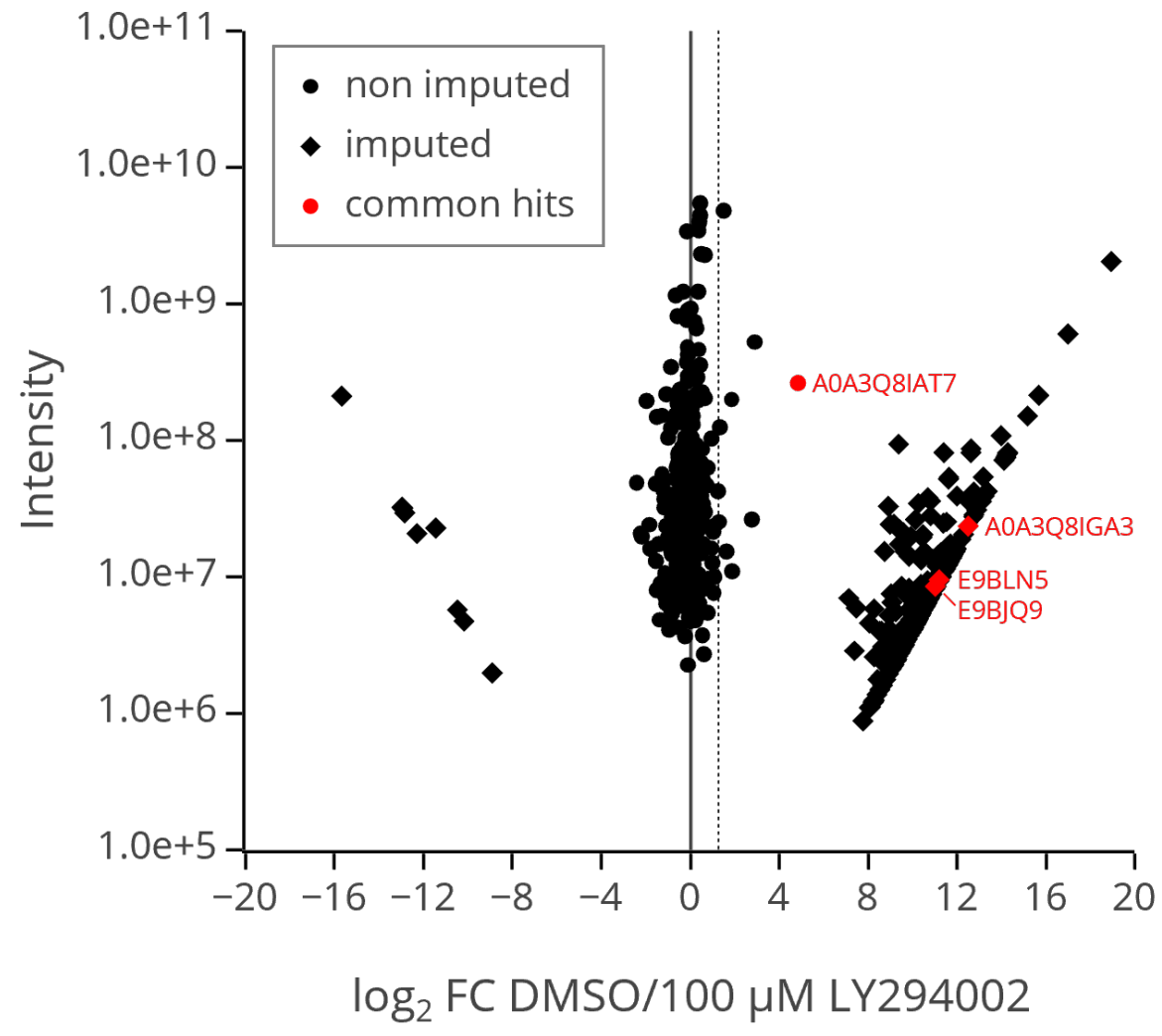

B

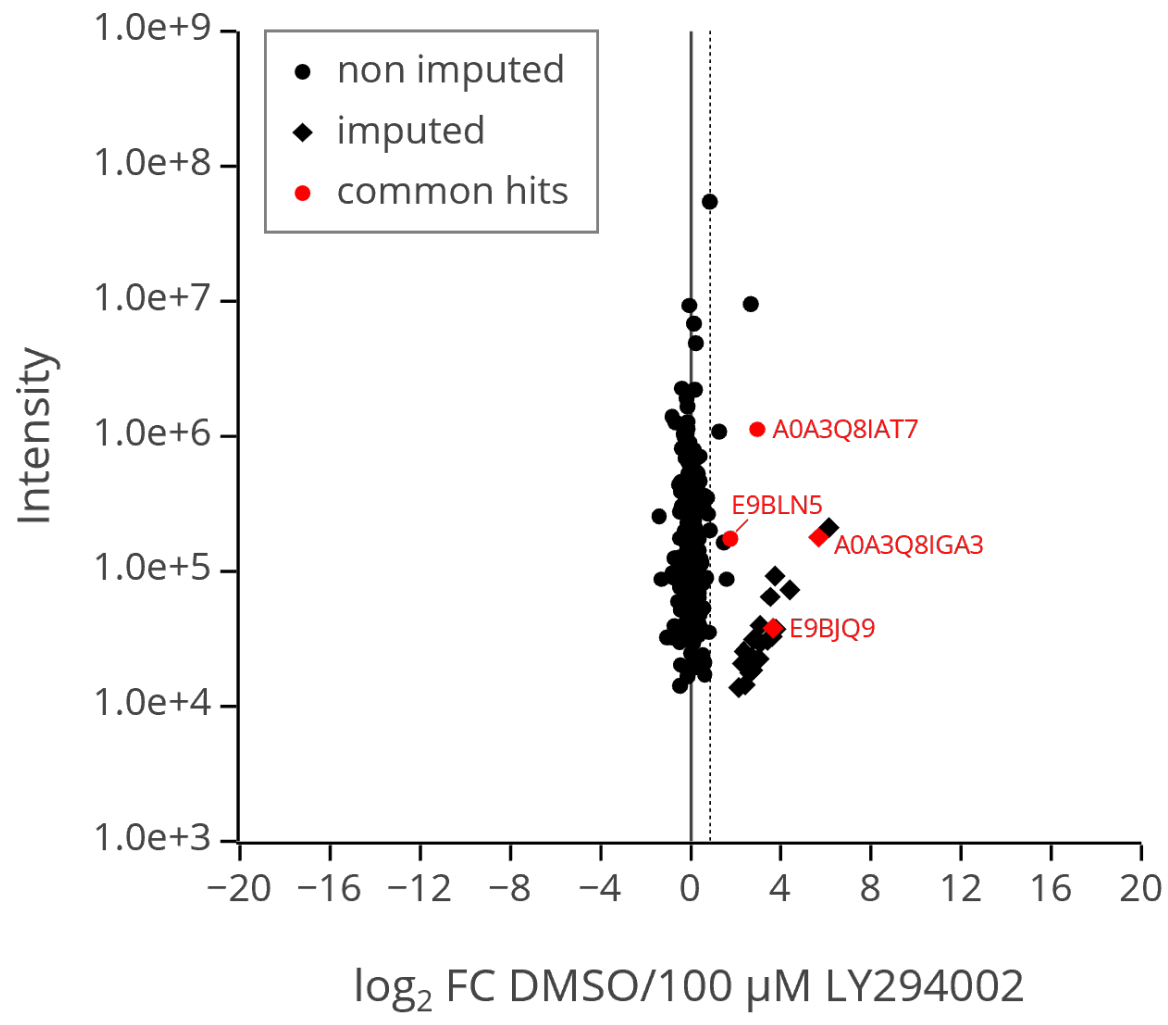

C

Name	Protein ID	Replicate 1		Replicate 2		Replicate 3	
		Pep.	log_2 FC	Pep.	log_2 FC	Pep.	log_2 FC
non-specific serine/threonine protein kinase	E9BJQ9	35	10.72*	64	11.03*	11	3.63*
phosphatidylinositol 3-kinase	A0A3Q8IGA3	36	2.53	46	12.51*	16	5.68*
serine/threonine-protein kinase RIO2	E9BLN5	19	2.53	23	11.19*	14	1.77
Cytidine deaminase-like protein	A0A3Q8IAT7	10	4.78	15	4.84	11	2.95

* log_2 FC calculated from imputed values

Figure S1. LY294002 affinity pulldown (replicates 2 and 3). (A) Enrichment plot of replicate 2. (B) Enrichment plot of replicate 3. (C) Table of commonly enriched hits from all three replicates with peptide numbers and log_2FC.

NMR assignment of 33

Regioselectiviy of thianthrenation was determined by analysis of 1D and 2D NMR spectra, a partial assignment is depicted in Figure S2. COSY correlations split the aromatic region into three well-defined groups. Protons 6-9 are on the same ring system and are consistent with the 8 protons of the thianthrene ring (highlighted blue). The three protons 1-3 are a second ring system, consistent with the three protons of the chromone ring (highlighted red). The remaining two aromatic resonances 4 and 5 (green) show the characteristic AA'XX' coupling of a *para*- substituted aromatic ring. This coupling pattern could only occur if thianthrenation has occurred at the *para*-position on this aromatic ring.

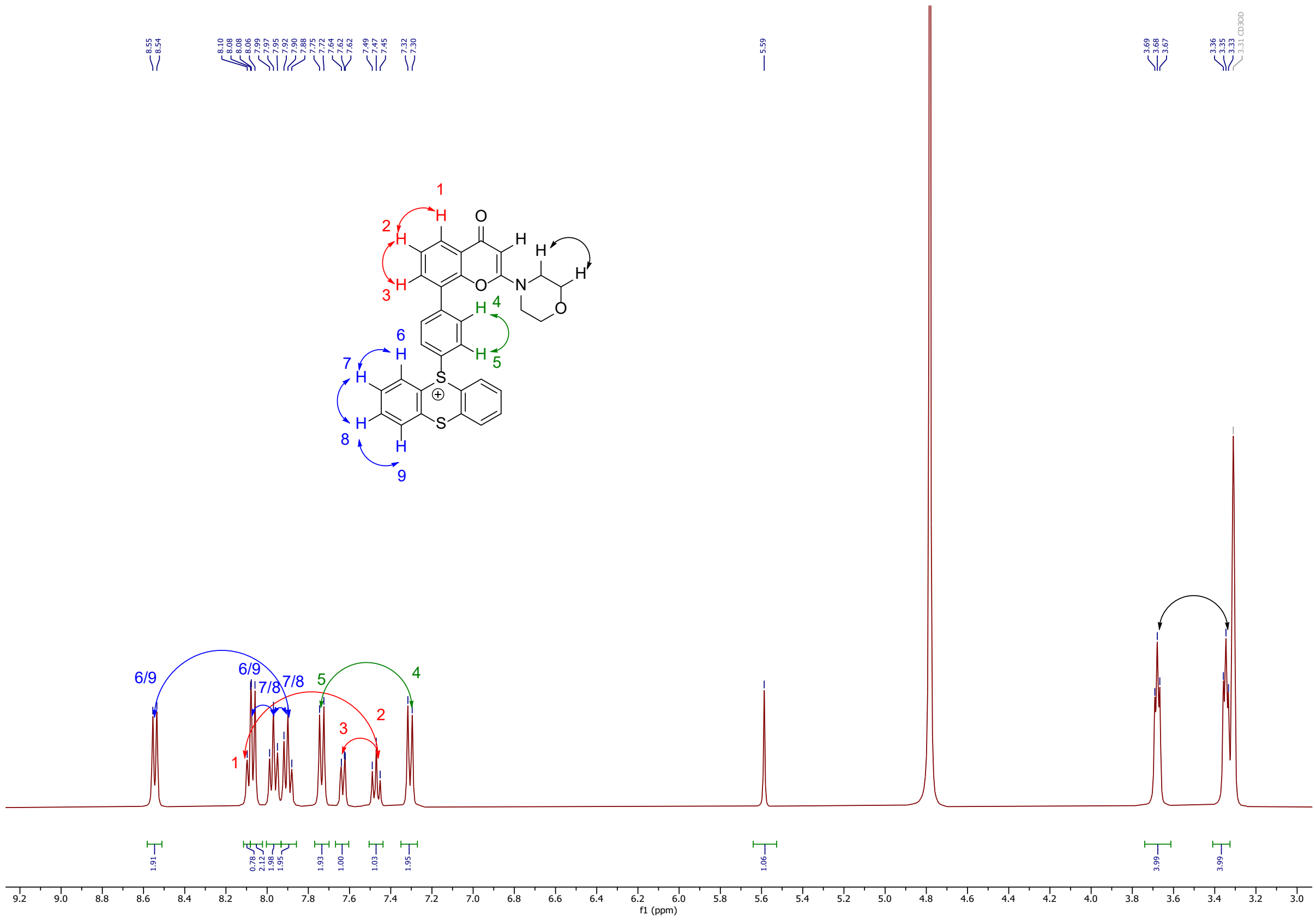

Figure S2. Partial ¹H NMR assignment of 33. Arrows represent COSY correlations and are colour coded to different ring systems; red is the aromatic ring of the chromone bicycle, blue are the thianthrene ring, green are the *para*-substituted aromatic ring, black are the morpholine ring protons.

NMR assignment of S4

Using COSY, HMBC and NOE correlations the different aromatic rings could be differentiated, key interactions are summarised in **Table S7**. The relevant aryl protons were identified as 7.35-7.33 (m, 2H) and 7.57 (dd, *J* = 2, 1 Hz) ppm. The coupling constants of 7.57 (dd, *J* = 2, 1 Hz) ppm require thianthrenation to have occurred at either C3 or C4, as the observed *J*-value for this resonance of 2.0 and 1.0 Hz are consistent with long-range *meta*- and *para*-coupling. Thianthrenation at any other position would require every aromatic proton on this ring to have a vicinal (*ortho*) coupling constant of ~7-10 Hz. This resonance does not display a NOE correlation to the methoxy protons (H7, 3.95 ppm). This is consistent with H2 and thianthrenation at the C3 position. The multiplet 7.35-7.33 (m, 2H) was therefore assigned as H4 and H5.This assignment is consistent with the reported *para*-selectivity of this reaction.[1]

Table S7. Partial assignment of S4.

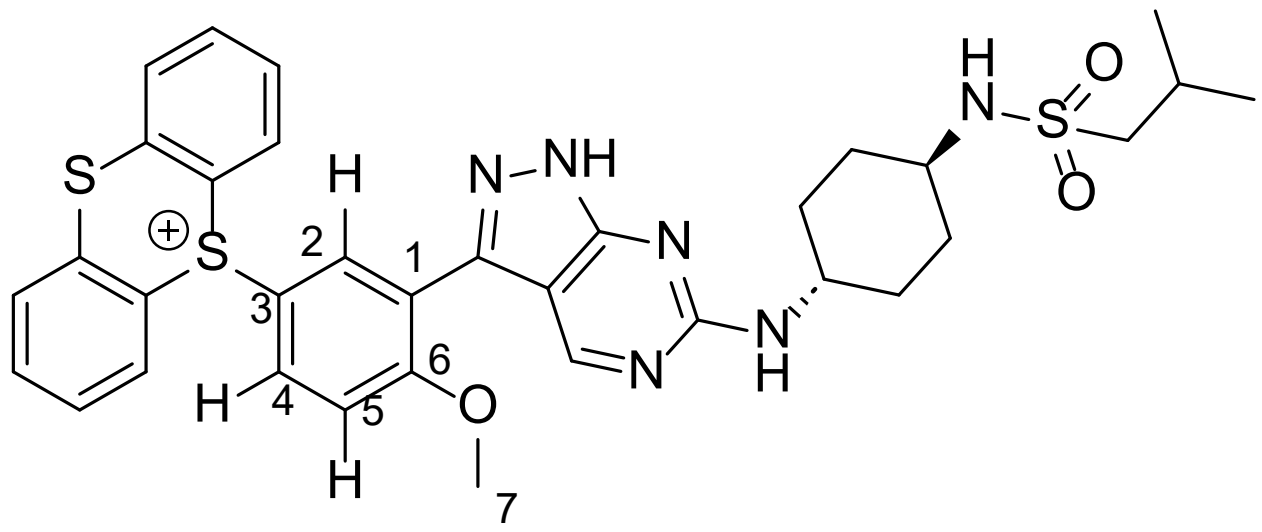

1H δ	13C δ (HSQC)	Key HMBC δ	COSY δ	Key NOE δ	Assignment
8.69 (s, 1H)	156.6		-		Heterocycle C-H
8.54 (s, 1H)	N/A		-		Formic acid
8.43 (dd, 2H)	135.6		7.83		Thianthrene proton
8.01 (dd, 2H)	131.7		7.91		Thianthrene proton
7.91 (td, 2H)	136.1		7.83, 8.01		Thianthrene proton
7.83 (td, 2H)	131.1		7.91, 8.43		Thianthrene proton
7.57 (dd, 1H, *J*=2.0, 1.0 Hz)	130.7	161.7 ,141.7, 130.7, 125.6	7.35-7.32		C2
7.35-7.32 (m, 2H)	115.0	161.7, 115.8, 130.8, 125.6	7.57	3.95	H5/H4
3.95 (s, 3H)	56.9	161.7	-	7.35-7.32	C7

NMR assignment of S1

Using COSY, HMBC and NOE correlations the different aromatic rings could be differentiated, key interactions are summarised in **Table S8**. Based on these data it was possible to identify the three indoline proton resonances as 7.98 (d, *J*=8.3 Hz), 6.94 (d, *J*= 8.3 Hz) and 6.92 (s) ppm (highlighted yellow in Table S11). A Key HMBC correlation between 6.92 (s) ppm and 34.9 ppm (C2) allows this proton resonance to be assigned as H4 which is consistent with the thianthrene group being attached at C5 position. Thianthrenation at C5 is also consistent with the *para*-selectivity of this reaction reported in the literature.

Table S8. Partial assignment of S1.

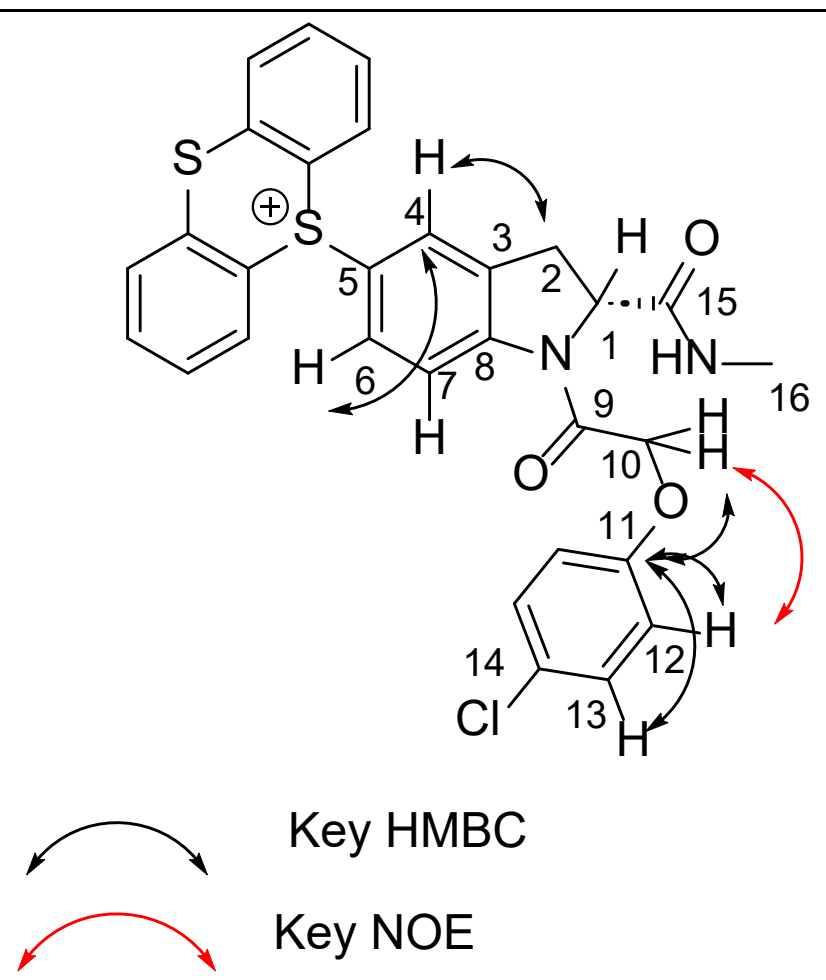

1H δ	13C δ (HSQC)	Key HMBC	COSY	Assignment
8.42 (t, 2H)	134.6		7.70	Thianthrene proton
7.98 (d, 1H)	118.2		6.94	H7
7.90-7.74 (m, 4H)	130.5, 130.6, 134.9, 135.0		7.90-7.74	Thianthrene proton

7.70 (2H)	130.3, 130.4		8.42, 7.90-7.74	Thianthrene proton
7.16-7.11 (m, 2H)	129.6	156.7 (C11)	6.90-6.85	H13
6.94 (d, 1H)	129.0	124.3 (C4)	7.98	H6
6.92 (s, 1H)	124.3	34.9 (C2)	None	H4
6.90-6.85 (2H)	116.2	156.7 (C11)	7.16-7.11	H12
5.67 (br s, 1H)	60.5		3.68-3.53, 3.12	H1
5.05 (d, 1H)	66.9	156.7 (C11)	4.64	H10
4.64 (d, 1H)	66.9	156.7 (C11)	5.05	H10
3.68 – 3.53 (m, 1H)	34.9		5.67, 3.12	H2
3.12 (dd, 1H)	34.9		5.67, 3.68 – 3.53	H2
2.79 (d, 3H)	26.5		26.5	H16

NMR Assignment of S2

Using COSY, HMBC and NOE correlations the different aromatic rings could be differentiated, key interactions are summarised in **Figure S3**. HMBC correlations from the methoxy proton δ3.91 to the C-O aromatic proton, C3 (δ159.9) enabled identification of two aromatic protons on the **A** ring at δ7.84-7.81 (m, 3H) and δ6.62 (d, *J* = 9 Hz, 1H), NOE correlation from δ3.91 to δ7.84-7.81 (m, 3H) confirmed this was *ortho*- to the methoxy group. A further NOE from δ3.91 to δ8.24 indicated the methoxy was also in close proximity to the thianthrene ring, and that thianthrenation had therefore likely occurred at the C2 or C4 positions. The final proton of the aromatic ring was identified via NOE from δ6.62 to δ7.33-7.30 (m, 3H). The lack of an HMBC correlation between δ7.33-7.30 and δ159.9 is consistent with this proton being *para*-to the methoxy at C3, therefore the proton in multiplet δ7.33-7.30 was assigned as H6, and δ6.62 as H5. The splitting pattern of H5 is a doublet with *J* = 9 Hz, consistent with *ortho* coupling to a single proton. Thianthrenation at C2 would be inconsistent with the observed splitting of H5 and 2D correlations, therefore thianthrenation has occurred at the C4 position, and δ7.84-7.81 can be assigned as the H2 proton resonance. This assignment is consistent with the reported *para*- selectivity of this reaction.

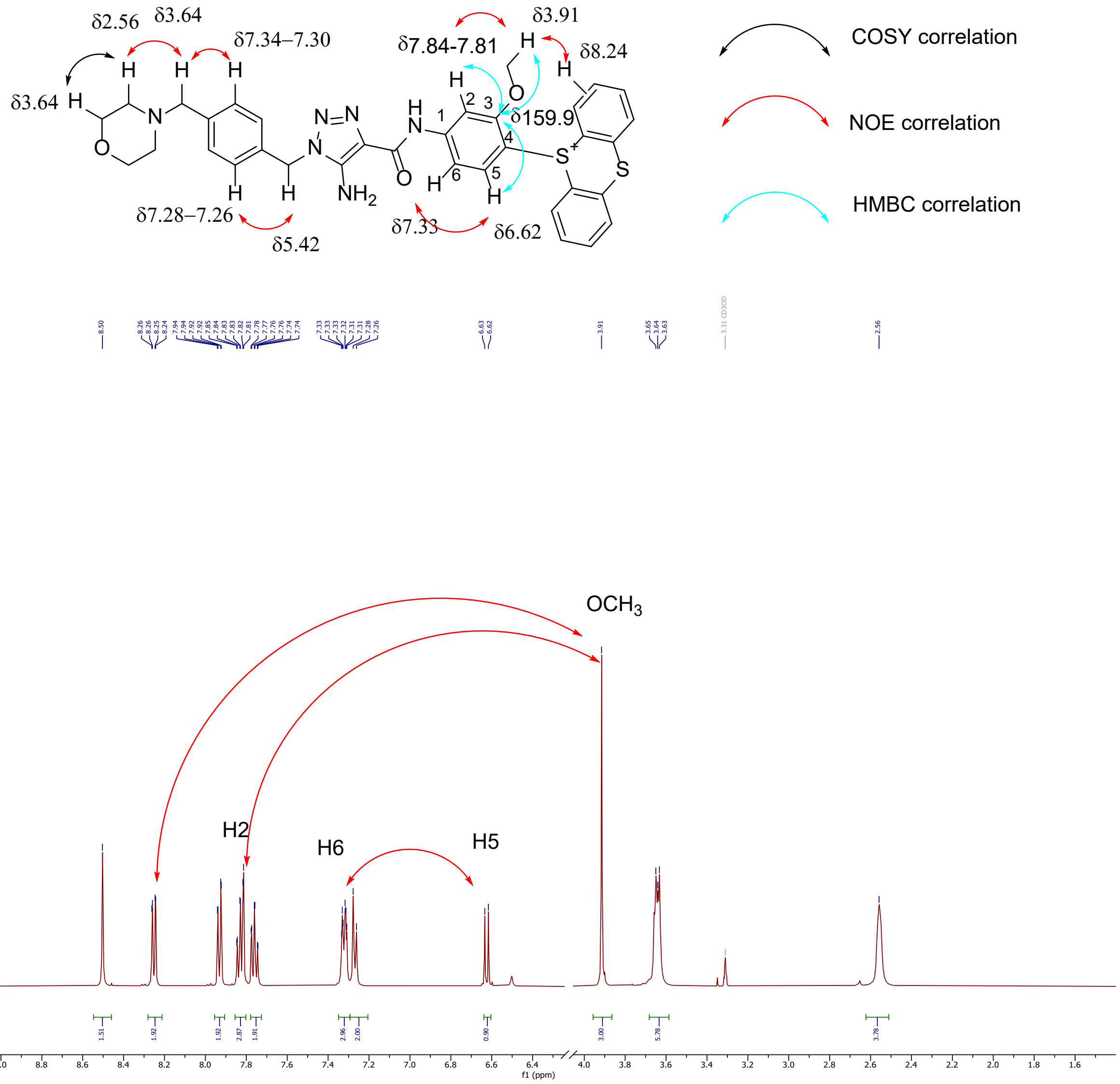

Figure S3. Key NMR interactions of S2.

NMR Assignment of S3

Using COSY, HMBC and NOE correlations the different aromatic rings could be differentiated, key interactions are summarised in **Figure S4**. NOE correlation from the methoxy resonance δ3.98 (s, 3H) enabled identification of the corresponding aromatic *ortho*-proton Ha δ7.34 (d, *J* = 9.1 Hz, 1H). COSY and NOE correlations enabled identification of H_b δ7.53 (dd, *J* = 9.1, 2.7 Hz., 1H). A further (weak) COSY correlation was observed between H_b and multiplet δ8.03-7.98 (m, 3H) which was assigned as H_c. The coupling constants of H_b are consistent with *ortho*-coupling to H_a and long-range *meta*-coupling to H_c. This assignment is consistent with thianthrenation *para*- to the methoxy substituent of ring A, and consistent with the reported *para*-selectivity of the reaction.[1]

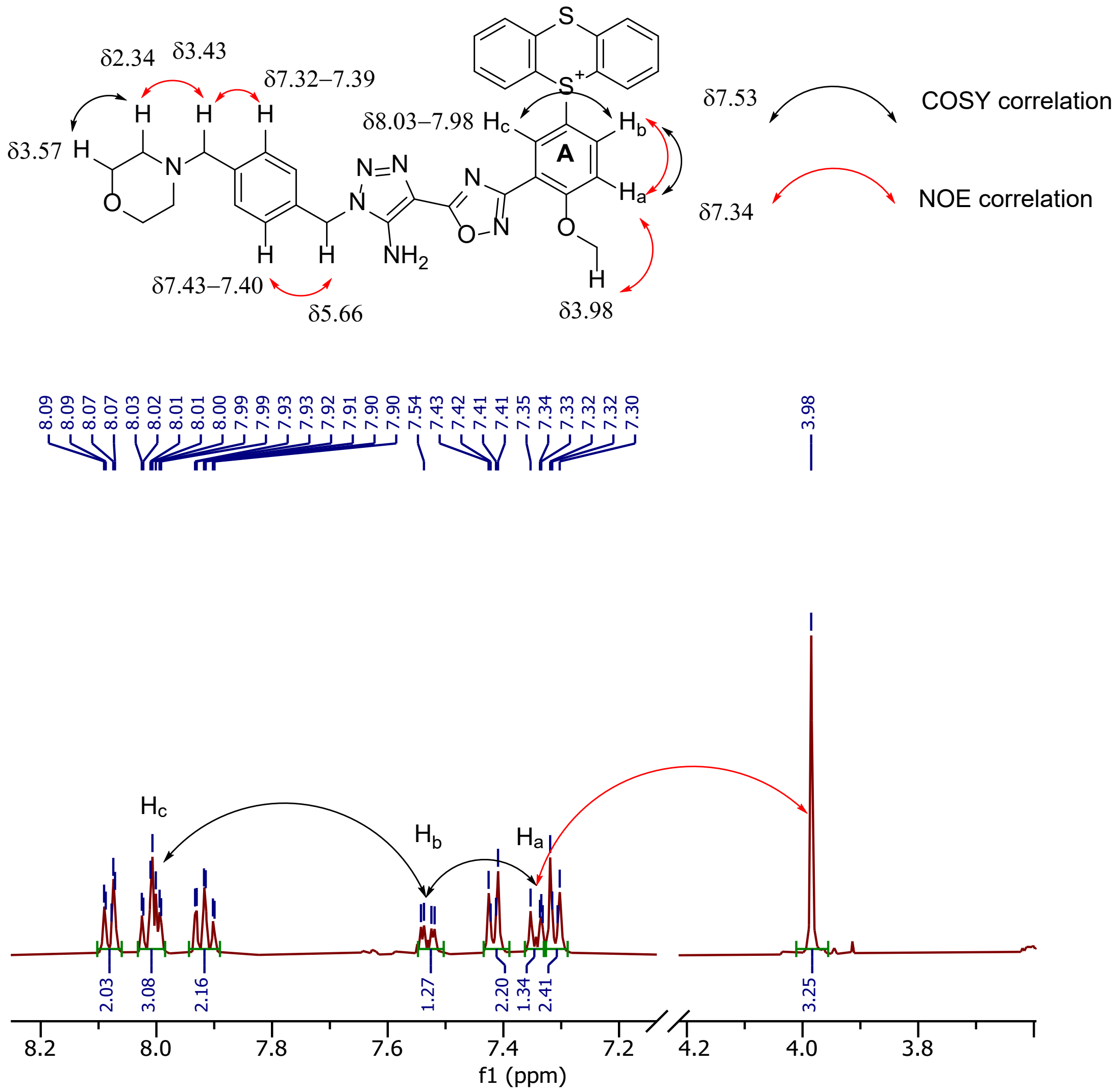

Figure S4. Key NMR interactions of S3.

Additional experimental procedures

High-throughput experimentation (HTE) procedures
Miniaturization of thianthrenation

Stock solutions of pyriproxyphen, HBF4 •Et_2O, TFAA and thianthrene-*S*-oxide were prepared in HPLC grade acetonitrile. During these tests a reduced performance was noted when stocks of thianthrene-*S*-oxide, TFAA and acid were not freshly prepared on the day. Therefore, fresh stock solutions of these reagents should be used wherever possible. The concentration of stock solutions used in this study and the corresponding final reaction concentrations are listed below.

- 80 mM pyriproxyphen (final concentration 10 mM)
- 40 mM pyriproxyphen (final concentration 5 mM)
- 10 mM pyriproxyphen (final concentration 1.25 mM)

- 80 mM HBF_4•Et_2O (final concentration 10 mM)
- 160 mM HBF_4•Et_2O (final concentration 20 mM)
- 200 mM HBF_4•Et_2O (final concentration 25 mM)
- 400 mM HBF_4•Et_2O (final concentration 50 mM)
- 800 mM HBF_4•Et_2O (final concentration 100 mM)

- 120 mM TFAA (final concentration 30 mM)
- 240 mM TFAA (final concentration 60 mM)
- 480 mM TFAA (final concentration 120 mM)

- 20 mM thianthrene-*S*-oxide (final concentration 10 mM)
- 10 mM thianthrene-*S*-oxide (final concentration 5 mM)
- 2.5 mM thianthrene-*S*-oxide (final concentration 1.25 mM)

Initial experiments were performed in low volume (~200 µL) HPLC vials. Sequential addition of 10 µL pyriproxyphen stock, 10 µL HBF_4•Et_2O stock, 20 µL trifluoroacetic anhydride stock and 40 µL thianthrene-*S*-oxide stock were added to the vial, the vial sealed and vortexed for 5 seconds. Upon addition of the thianthrene-*S*-oxide the reaction turned a pink to purple colour. The vials were left to sit for 3 h. After this time the reaction was stopped via addition of 1:1 MeCN:water (120 µL), the vial sealed and vortexed for 5 seconds. Samples were then analysed by LCMS, using the relative peak area (%) of the UV trace as an estimation of reaction conversion. Results of this analysis are presented in **Table S1**.

HTE screen of literature substrates

Stock solutions:

- 10 mM stocks of the following commercially available compounds were prepared: 3,4-dihydroquinolin-2(1*H*)-one, 1,2-dimethoxybenzene, methoxybenzene, methyl-2-methoxybenzoate, 2-methoxybenzaldehyde, *N*,*N*-dimethylaniline, toluene, 2-bromo-4-methyltoluene, fluorobenzene, 1,2-dichlorobenzene and chlorobenzene. Note. pyriproxyphen was also added to the plate as a positive control.

- 5 mM thianthrene-*S*-oxide
- 800 mM HBF_4•Et_2O + 960 mM TFAA
- 800 mM BF_3•Et_2O + 960 mM TFAA
- 800 mM TfOH + 960 mM TFAA

The acid and TFAA solutions were combined into a single stock, when these reagents were added separately, activation of the thianthrene (indicated by the formation of a pink to purple colour) was inconsistent across the plate. Three different acids were tested: HBF_4•Et_2O, BF_3•Et_2O and TfOH.

Reactions were performed in a standard 96-well polypropylene plate.

To a 96 well plate was added 10 µL of each 10 mM substrate stock solution in triplicate, one for each acid condition. To each well was then added 10 µL of one of the acid/TFAA stock solutions and 20 µL of the 5 mM thianthrene-*S*-oxide stock, mixing each well via pipette after the final addition (20 µL injection volume, ×3). The plate was covered with aluminium foil and left for 3 h. After this time 160 µL of 1:1 MeCN:water was added to each well and the plate covered with a plate seal (Phenomenex Sealing Mats, Pre-Slit, 96-Round Well 7mm, Silicone) and then analysed by LCMS, using the relative peak area (%) of the UV trace as an estimation of reaction conversion. Results of this analysis are presented in **Table S2**.

HTE screen of phenotypically active compounds

Stock solutions:

- Compound **9** is commercially available (Combi-Blocks QI-9914), compounds **10-32** had been previously synthesized[2-9]
- 10 mM stocks of these compounds were prepared in MeCN.
    - Where compounds were incompletely soluble the suspension was sonicated for 10 minutes and vortexed immediately prior to pipetting into a plate
    - Suspensions generally solubilised upon addition of acid/TFAA solution
- 800 mM HBF_4•Et_2O + 960 mM TFAA in MeCN
- 800 mM BF_3•Et_2O + 960 mM TFAA in MeCN
- 5 mM thianthrene-*S*-oxide in MeCN

To a 96 well plate was added 10 µL of each 10 mM stock solution in duplicate, one for each acid condition. To each well was then added 10 µL of one acid/TFAA stock solution and then 20 µL of thianthrene-*S*-oxide stock solution, mixing each well via pipette after the final addition (20 µL injection volume, ×3). The plate was covered with aluminium foil and left for 3 h. After this time 160 µL of 1:1 MeCN:water was added to each well and the plate covered with a plate seal (Phenomenex Sealing Mats, Pre-Slit, 96-Round Well 7 mm, Silicone) and then analysed by LCMS, using the relative peak area (%) of the UV trace as an estimation of reaction conversion. Results of this analysis are presented in **Table S3**.

Drug bead synthesis

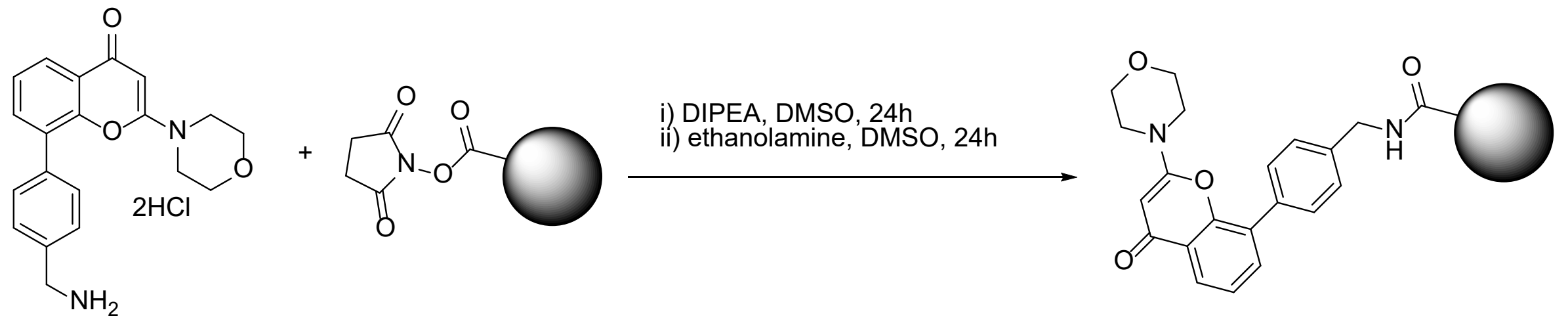

CZC15098 (9b) NHS-activated Sepharose lot 10350106 **CZC15098-matrix** 2 umol/mL settled resin; 9.1%, 20 nmol/20 µL slurry

Procedure is adapted from our previously published protocol in Smith *et. al.* (2023).[10]

Stock solutions

- 200 mM DIPEA in DMSO
- 200mM ethanolamine in DMSO
- 50 mM 8-(4-(aminomethyl)phenyl)-2-morpholino-4*H*-chromen-4-one dihydrochloride (**9b**, CZC15098) in DMSO

Reaction solution: 8-(4-(aminomethyl)phenyl)-2-morpholino-4*H*-chromen-4-one dihydrochloride (12.3 µL, 50 mM) and DIPEA (24.6 µL, 200 mM) were combined and diluted with DMSO (270.6 µL).

Immobilization procedure:

NHS-activated Sepharose fastflow 4 (Cytiva, 300 µL settled resin) was added to amicrocentrifuge tubes and washed with DMSO (1000 µL × 3). The resin was pelleted by centrifugation (1000 rcf, 2 min) and the supernatant discarded. To the tube of resin was added the reaction solution (300 µL). The vials were capped, shaken and pelleted by centrifugation (1000 rcf, 2 min). A 10 µL aliquot was taken from the tube, diluted with 1:1 MeCN/water (190 µL) and analysed via LCMS. The tube was mixed and rotated for 24 h on a Miltenyi Biotec MACSmix Tube Rotator. After this time a 10 µL aliquot was taken from the tube, diluted with 1:1 MeCN:water (190 µL) and analysed via LCMS. The resin was washed with DMSO (1000 µL × 3), then unreacted NHS groups were blocked with 200 mM ethanolamine (200 µL) and the tube was mixed and rotated for 24 h on a Miltenyi Biotec MACSmix Tube Rotator. After this time the resin was washed with DMSO (1000 µL × 3) and then transferred into isopropanol (1000 µL × 3) and stored as a 1:1 slurry of resin:isopropanol.

LCMS analysis of the two aliquots taken at the initiation and end of reaction allowed the percentage of probe remaining in solution to be estimated, it is assumed that all probe removed from solution was attached to the resin. The drug beads had 0.6 µmol probe attached, covering 9.1% of the reactive NHS groups present on the resin.

Biological, chemical pulldown and proteomics protocols

Cell lines and culture conditions

All cell lines investigated in this study were generated from a clonal *Leishmania donovani* cell line LdBOB (derived from MHOM/SD/62/1S-CL2D). Throughout, parasites were grown as promastigotes at 28°C, as previously described.[11]

Drug sensitivity assays in *L. donovani*

Drug sensitivity assays and data analysis were carried out as previously described.[12] Experiments were performed at least in three independent biological replicates with the data presented as the weighted mean ± standard deviation.

Affinity pulldown protocol

*L. donovani Ld*BOB was maintained as promastigotes at 28°C in medium specific for this developmental stage.[11] LdBOB promastigotes in mid-log growth phase were harvested by centrifugation (1,000 g, 10 min, 4°C) and washed 3× with ice-cold PBS. Cell pellets were resuspended in 1 mL of ice-cold lysis buffer (60 mM $MgCl_2$, 0.5% v/v IGEPAL CA-630, 1 mM DTT, 1× Roche EDTA-free cOmplete protease inhibitor cocktail, and 1× Roche PhosSTOP phosphatase inhibitor cocktail in 50 mM Tris-HCl, pH 7.5). Parasites were biologically inactivated by 3 freeze−thaw cycles in a dry ice/ethanol bath and lysed using a cell disruptor (Constant Systems, UK) at 10 kPSI. Clarified lysates were obtained by centrifugation (16,000 g, 15 min, 4°C) and protein concentrations were measured using the Bio-Rad Protein Assay.

Affinity pulldown experiments were performed as previously described,[10] with minor modifications. Briefly, lysates were pre-cleared by end-over-end incubation with blank beads for 30 min at 4°C to remove proteins that bind non-specifically to the resin. Pre-cleared lysates were incubated end-over-end with either DMSO or LY294002 (0.01-100 µM final concentration) for 30 min at 4°C. Drug beads were then added to the lysates and incubated end-over-end for 30 min at 4°C. Beads were recovered by centrifugation (16,000 g, 30 s, RT) and unbound material was discarded. Beads were washed 3 times with wash buffer (5 mM EDTA, 0.5% v/v IGEPAL CA-630, and 1 mg/mL bovine serum albumin in 50 mM Tris-HCl, pH 7.5), followed by 3 washes with 0.1 M triethylammonium bicarbonate (TEAB), pH 8.5.

Sample preparation and analysis by LC-MS/MS

Captured proteins were processed for mass spectrometry using the Suspension-trap method (S-TRAP, ProtiFi) according to the manufacturer's instructions.[13] Briefly, beads were incubated in 5% SDS in 0.1 M TEAB and sonicated in a water bath for 10 min. Proteins were reduced with 5 mM tris(2-carboxyethyl)phosphine (TCEP) for 15 min at 55°C and alkylated with 40 mM iodoacetamide for 30 min at RT in the dark. Following acidification with 2.5% phosphoric acid, proteins were captured on S-Trap columns and digested with 2 µg of trypsin/LysC at 37°C for ~16 h. Peptides were sequentially eluted with 0.1 M TEAB, 1% formic acid, and 50% acetonitrile, then dried by vacuum evaporation.

LC-MS/MS was performed on a Orbitrap Exploris coupled to a Vanquish Neo UHPLC (Thermo Scientific) in DIA mode. Peptides were eluted from the analytical column (Easy-Spray Neo UHPLC column, 50cm length) for 75 min in a gradient from 3-40% buffer B (80% acetonitrile in 0.1% formic acid). A scan cycle comprised of MS1 scan (m/z range from 380-980, with a maximum ion injection time of 25 ms, a resolution of 240,000 and absolute automatic gain control (AGC) value of 500% followed by scan events of 8 m/z non-overlapping windows. Fragmentation was carried out in HCD mode and Normalised collision energy was set to 30%. MS2 scans were carried out at a resolution of 30,000 with an absolute AGC value of 500%. Data was searched using DIA-NN version 2.3 Academic with *in silico* generation of a spectral

library against the *L. donovani* BPK282A1 proteome (Uniprot, accession UP000008980). Trypsin/P was set as a protease with one missed cleavage permitted, N-term M excision, oxidation (M), and acetylation (N-term) were included as variable modifications while carbamidomethylation (C) was included as fixed modification. Unrelated runs and MBR options were enabled, cross-run normalisation was disabled, and all other settings were kept default. All pulldown proteomics data sets have been deposited in the ProteomeXchange Consortium via the PRIDE partner repository under the identifier **PXD075457**.[14]

Enrichment analysis and dose-response modelling

Proteomic datasets were filtered to remove contaminants and proteins detected with ≤ 2 peptides. Missing intensity values were naïvely imputed using the lowest detected intensity value in the dataset.

Enrichment was assessed by calculating the $\log_2$ fold change ($\log_2$ FC) between DMSO and samples treated with 100 µM competitor. Proteins were considered enriched when the $\log_2$ FC exceeded a hit threshold defined as the median $\log_2$ FC plus 4 median absolute deviations (MAD), calculated from non-imputed data.

Dose-response data were analysed in R (version 4.5.1). Drug concentrations were fitted against protein intensities using a four-parameter logistic (4PL) model implemented in the drc package (function drm, model LL.4).

NMR and LCMS spectra

2,3,7,8-tetrafluoro-5-(4-(4-(2-(pyridin-2-yloxy)propoxy)phenoxy)phenyl)-5*H*-thianthren-5-ium tetrafluoroborate (1)

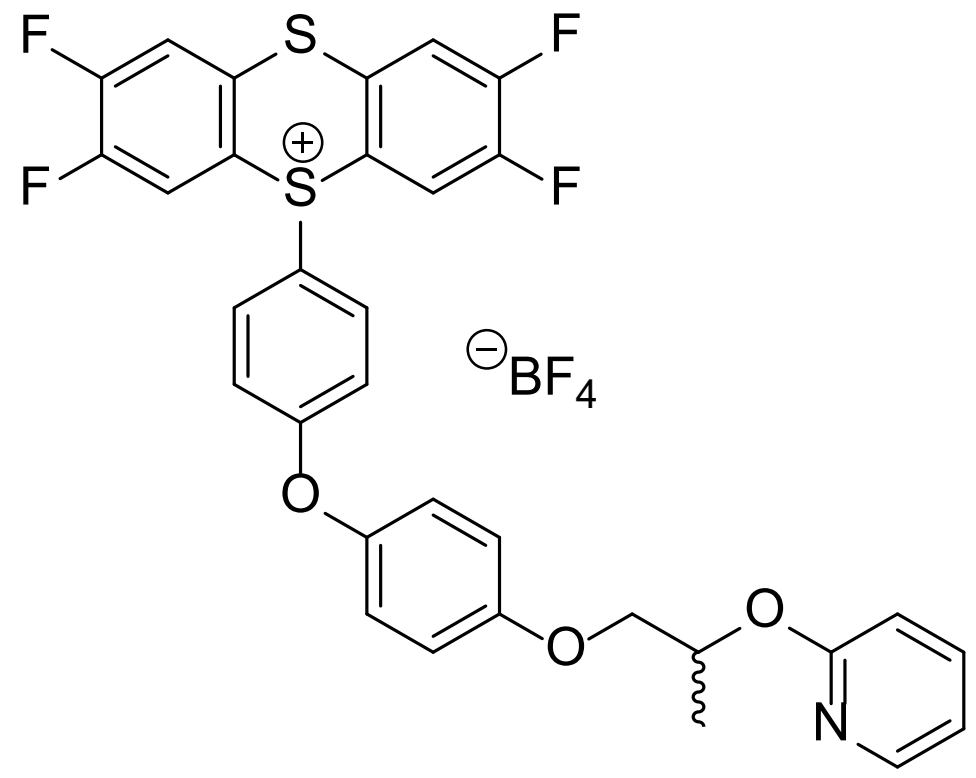

$^1H\{^{19}F\}$ NMR (500 MHz, $CDCl_3$, 303K)

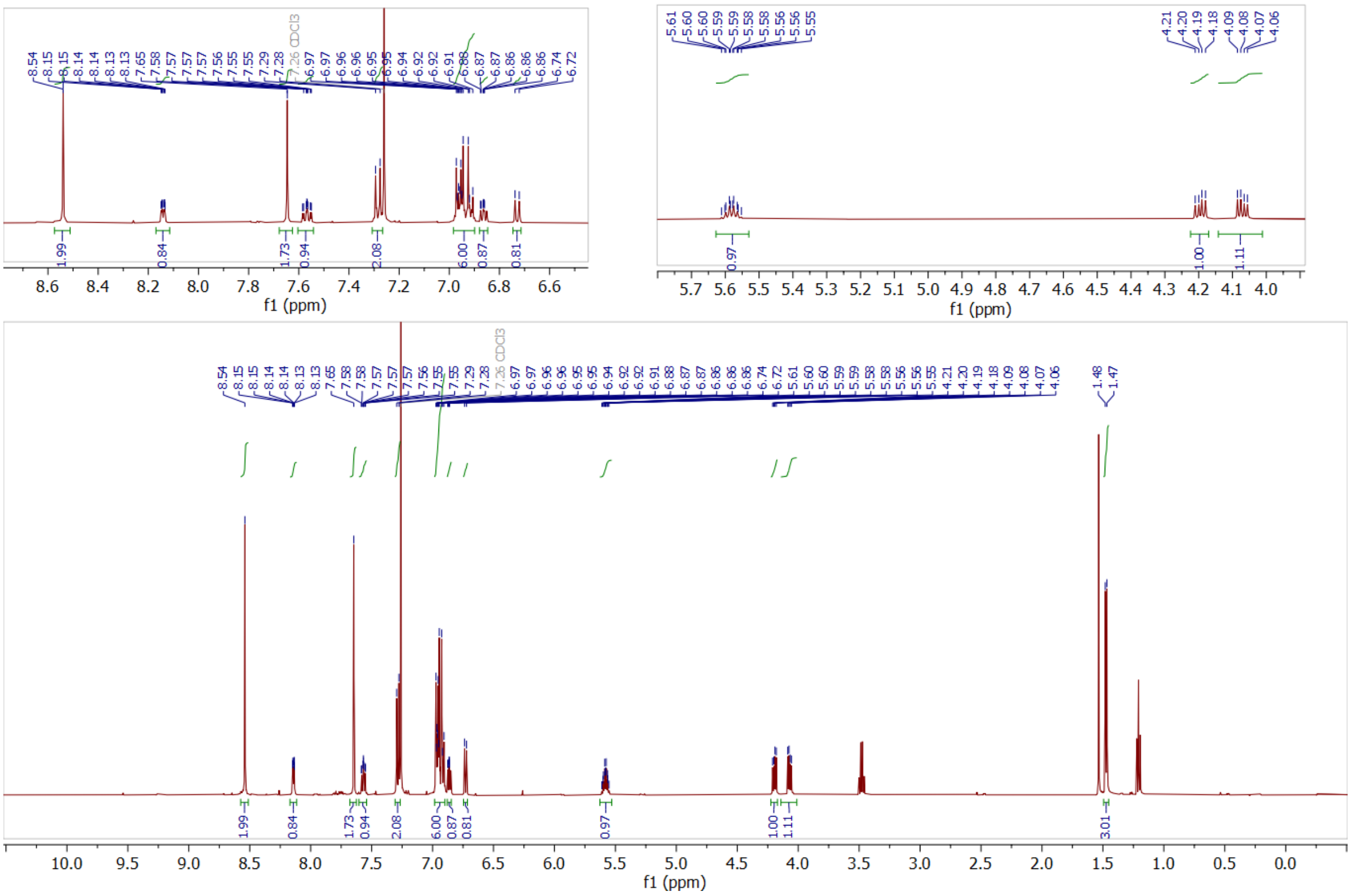

$^{19}F\{^{1}H\}$ NMR (471 MHz, $CDCl_3$, 303K)

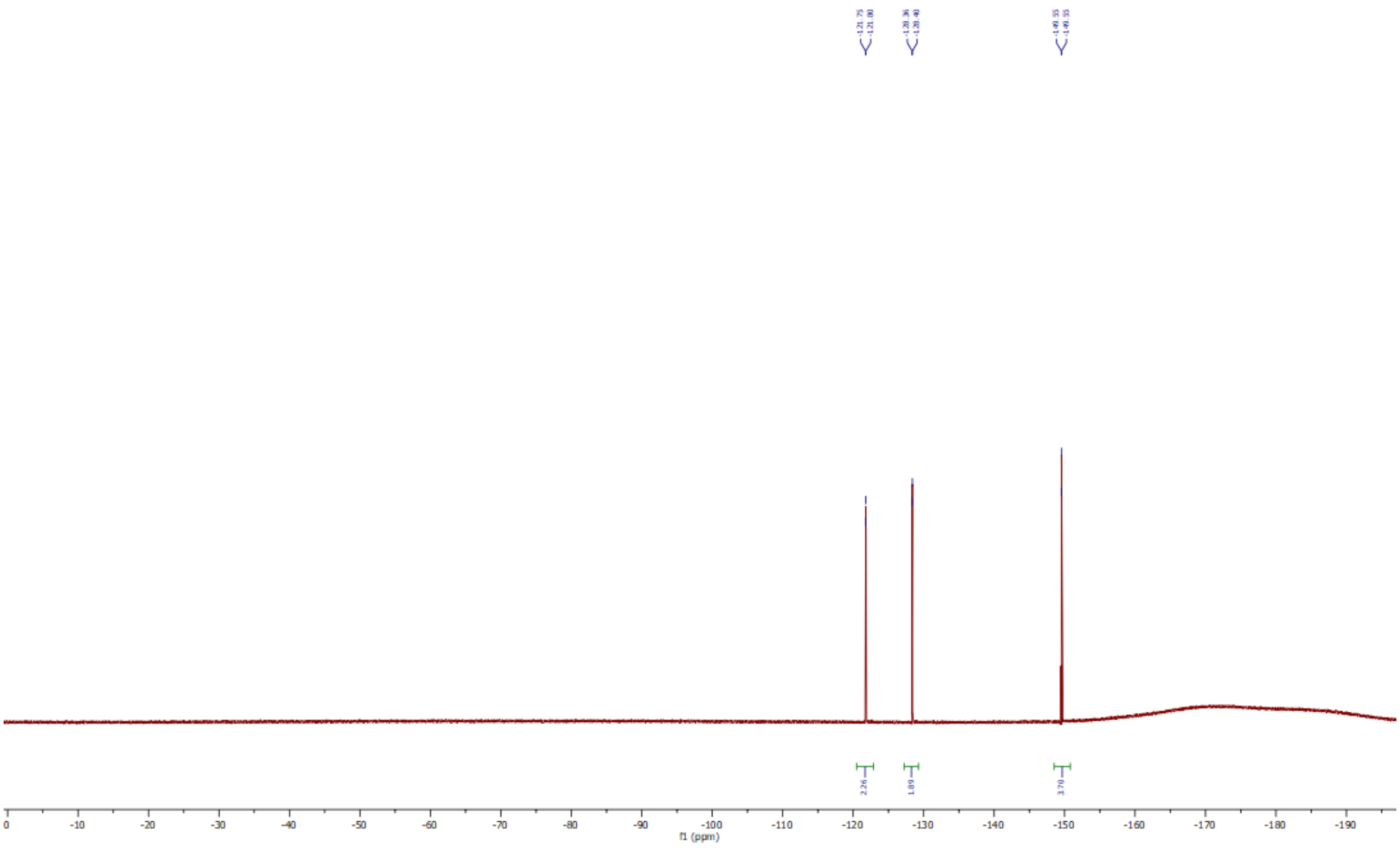

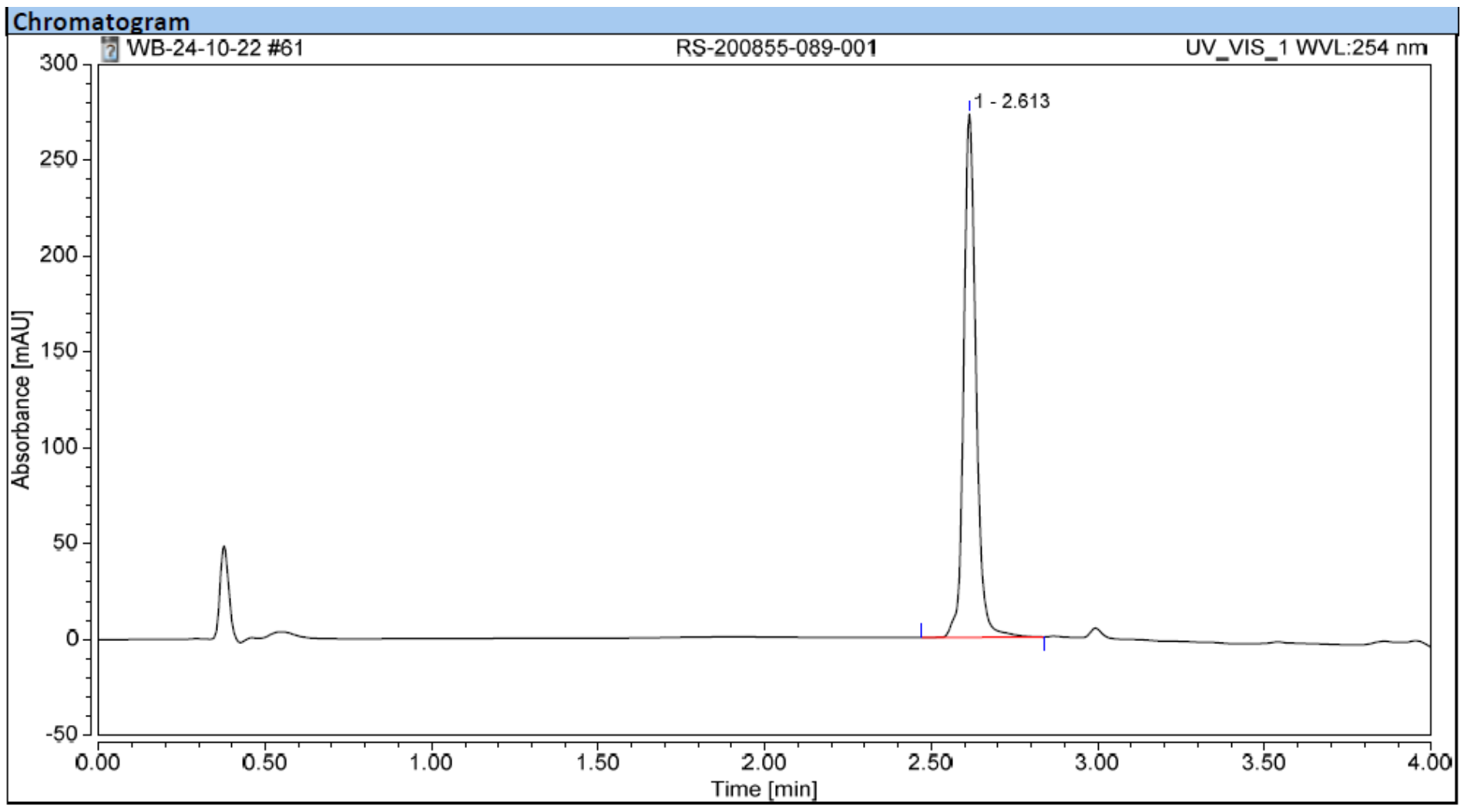

Integration Results

No.	Peak Name	Retention Time min	Area mAU*min	Height mAU	Relative Area %	Relative Height %	Amount n.a.
1		2.613	11.668	272.902	100.00	100.00	n.a.
**Total:**			**11.668**	**272.902**	**100.00**	**100.00**	

Spectrum RT 2:40 (1 scans)
null;
ESI + Max: 1.2E8

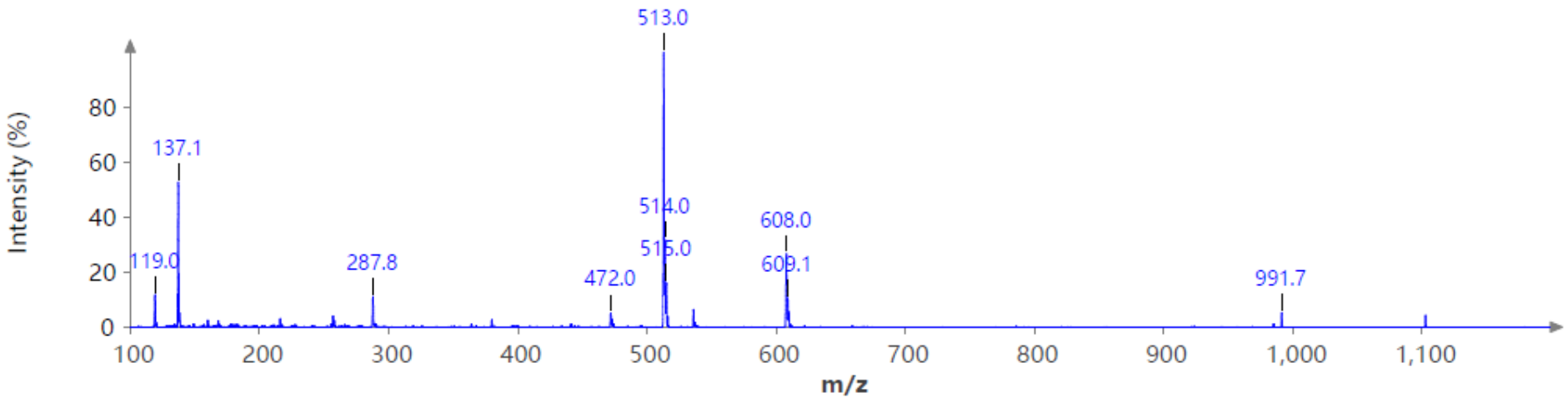

5-(4-(4-(2-(pyridin-2-yloxy)propoxy)phenoxy)phenyl)-5*H*-thianthren-5-ium tetrafluoroborate (2)

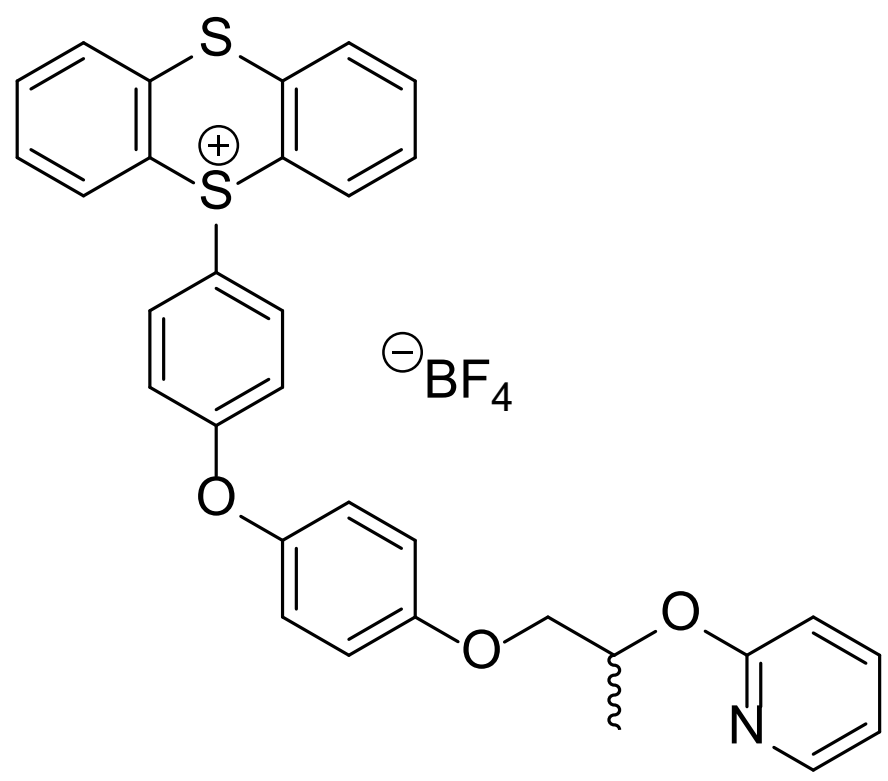

^{1}H NMR (500 MHz, $CDCl_3$, 303K)

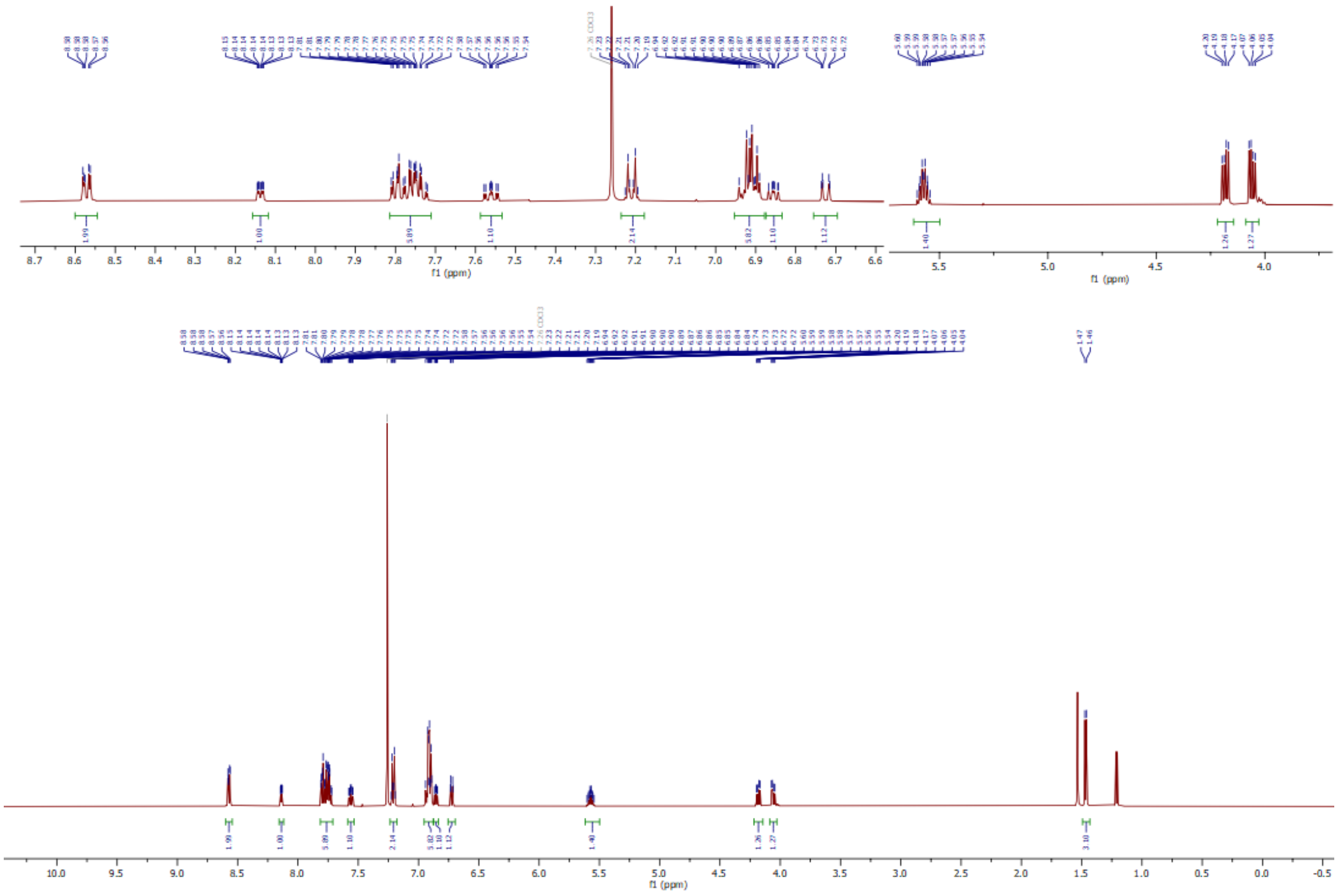

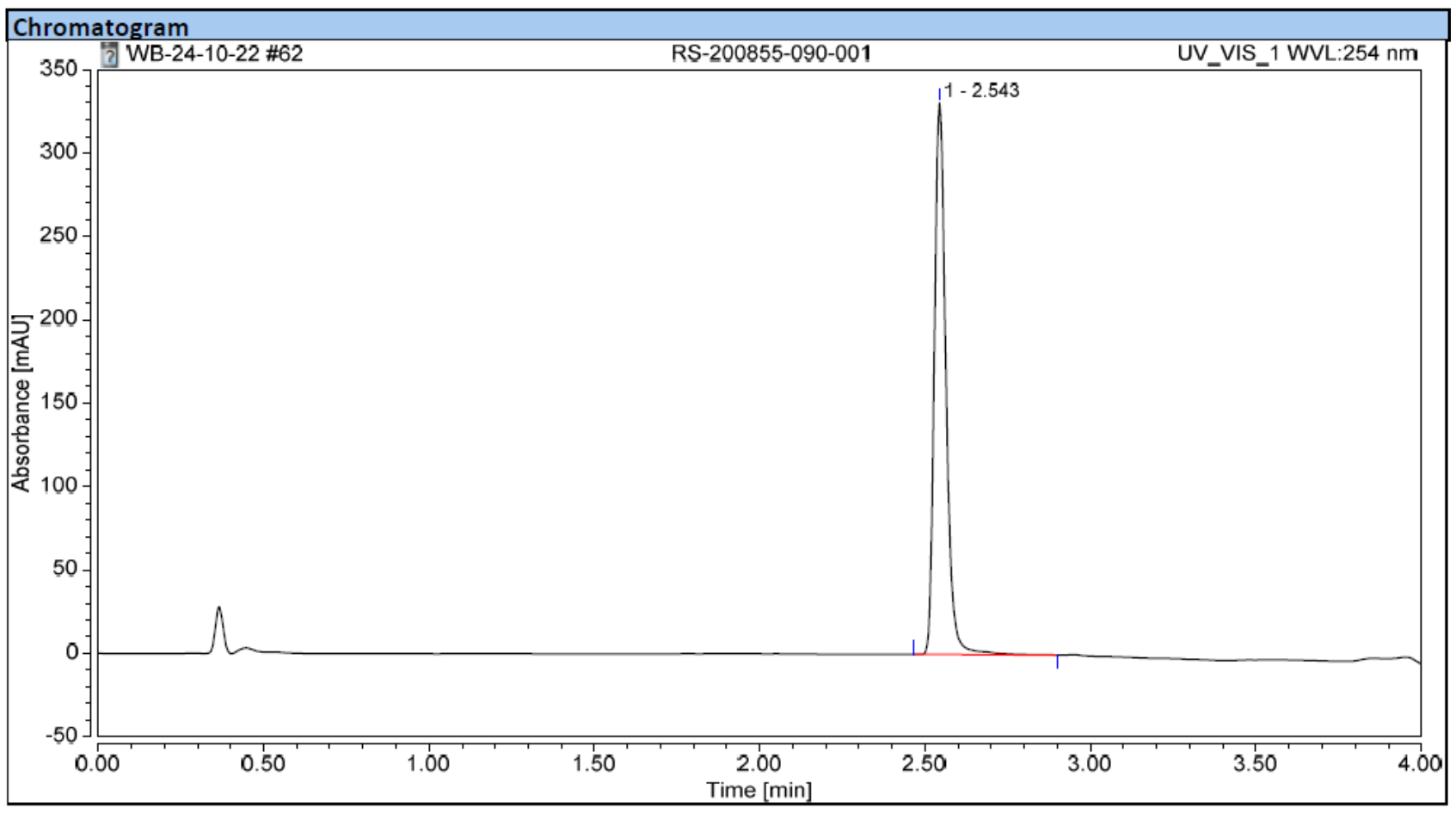

Integration Results

No.	Peak Name	Retention Time min	Area mAU*min	Height mAU	Relative Area %	Relative Height %	Amount n.a.
1		2.543	13.509	330.691	100.00	100.00	n.a.
**Total:**			**13.509**	**330.691**	**100.00**	**100.00**	

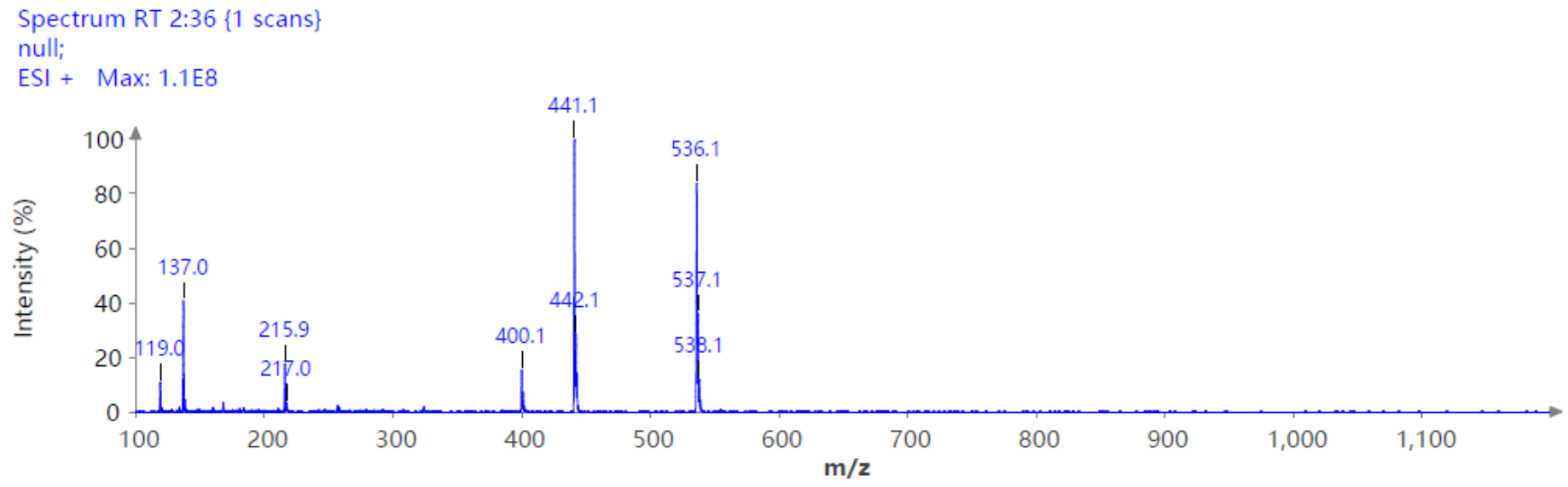

tert-butyl N-[2-[2-(2-prop-2-ynoxyethoxy)ethoxy]ethyl]carbamate (3d)

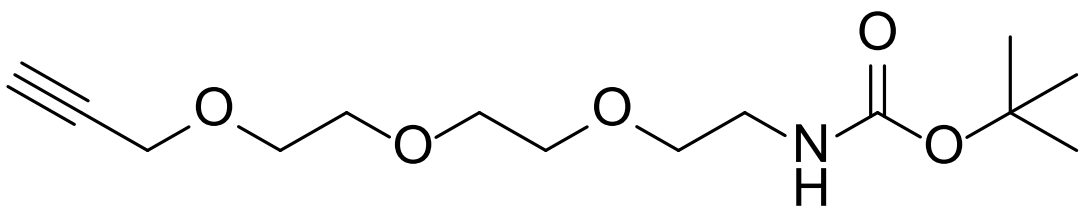

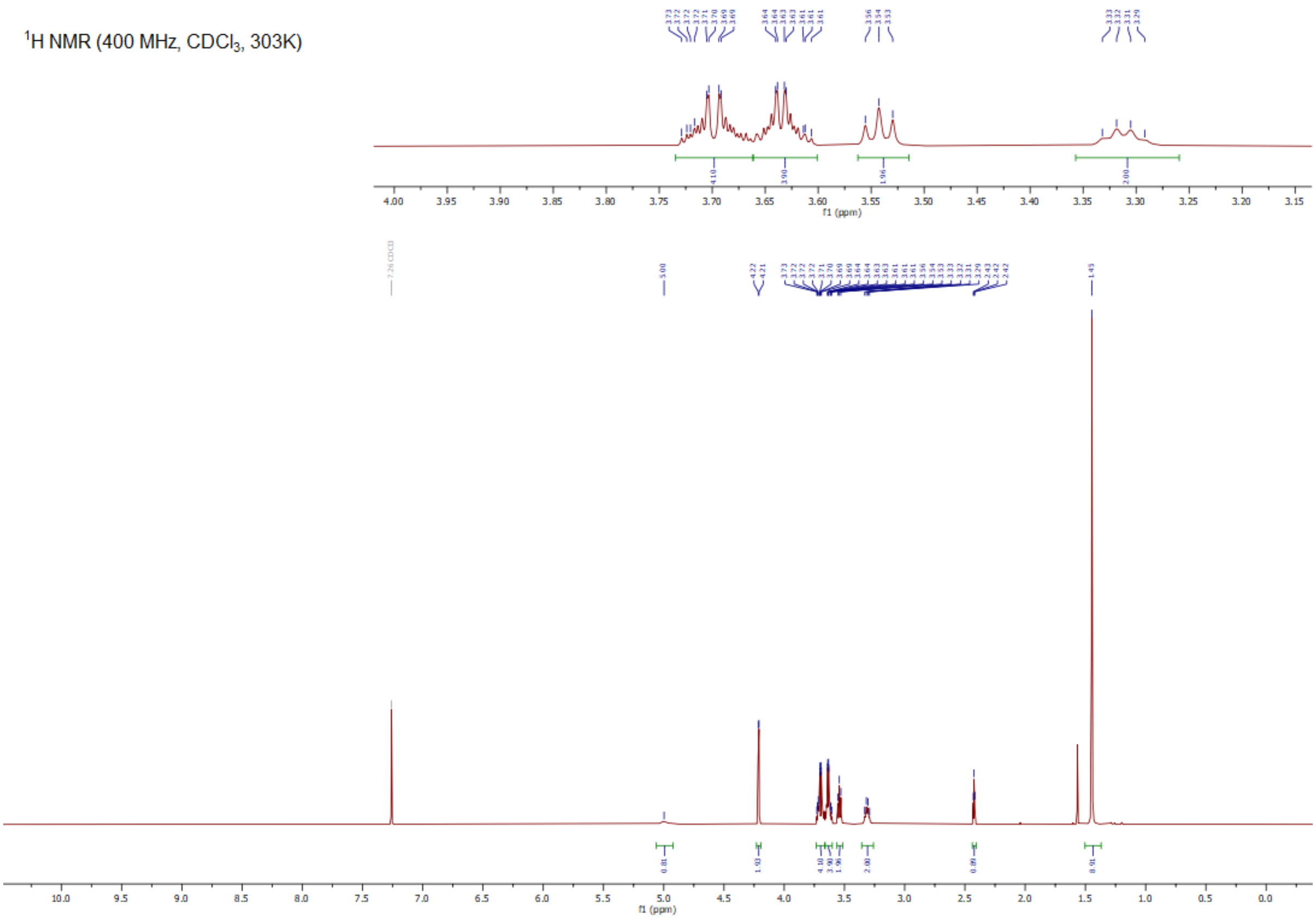

^{13}C NMR (101 MHz, $CDCl_3$, 303K)

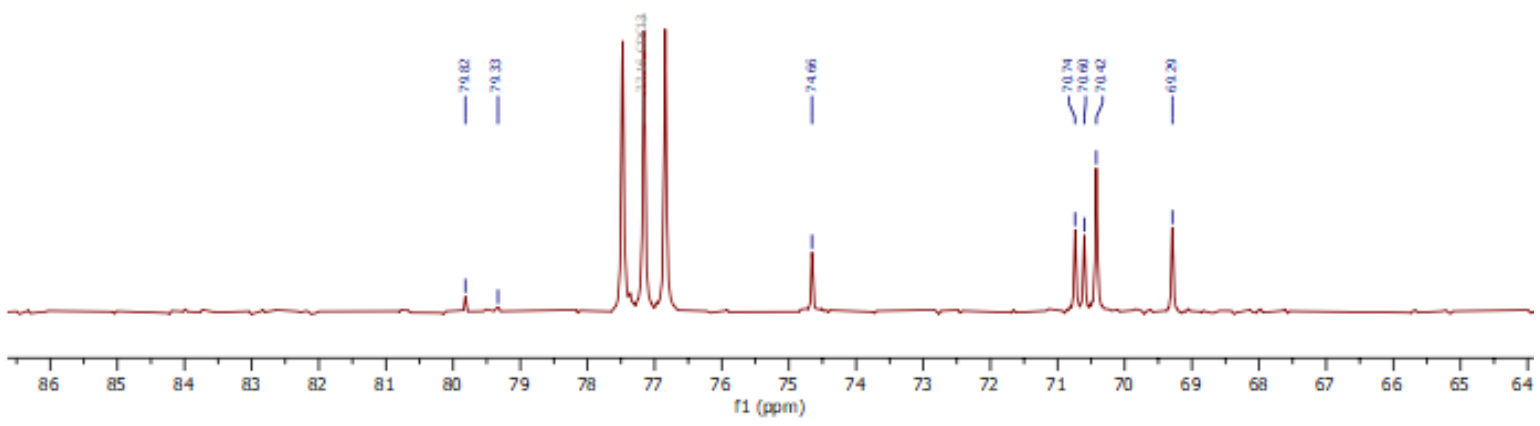

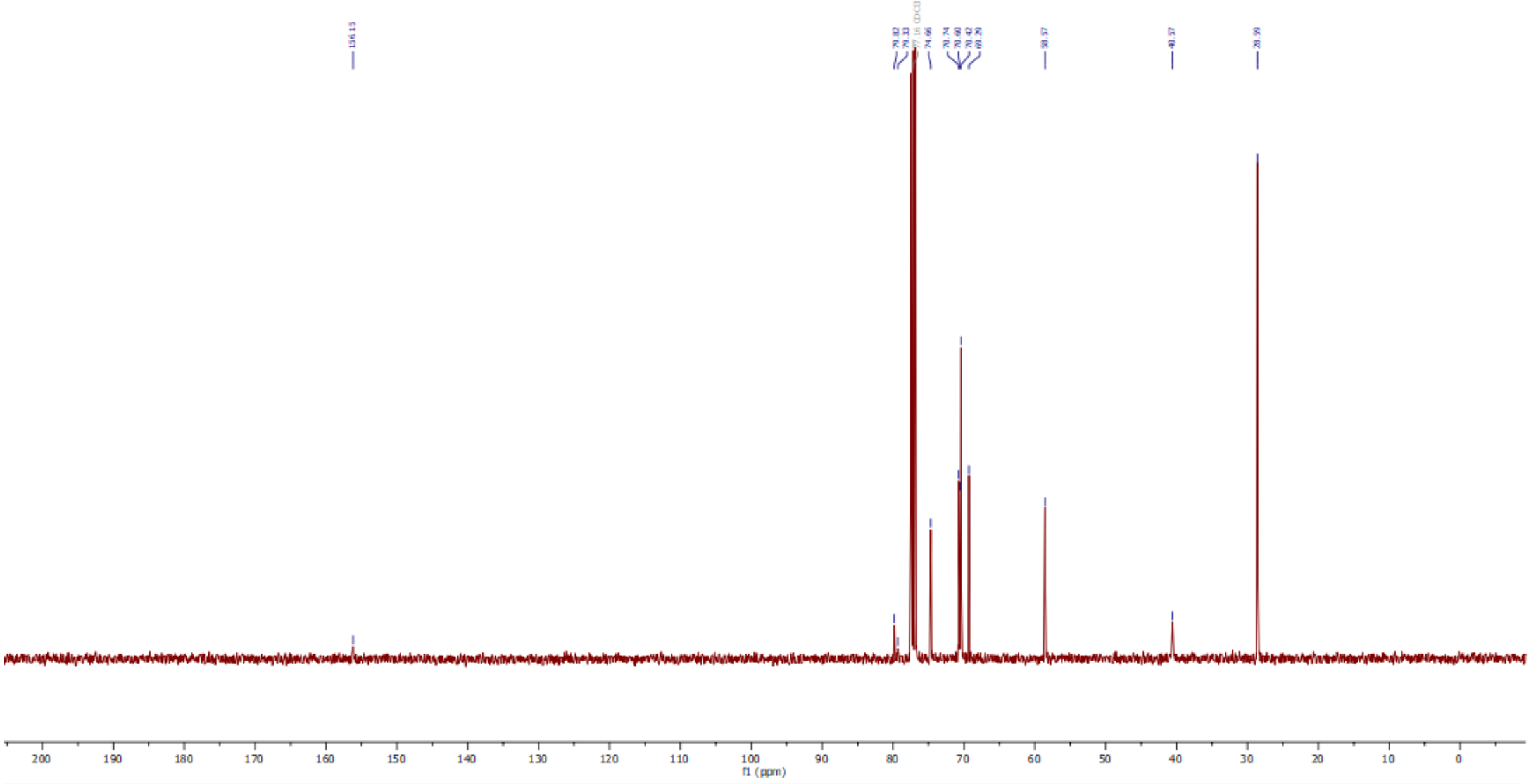

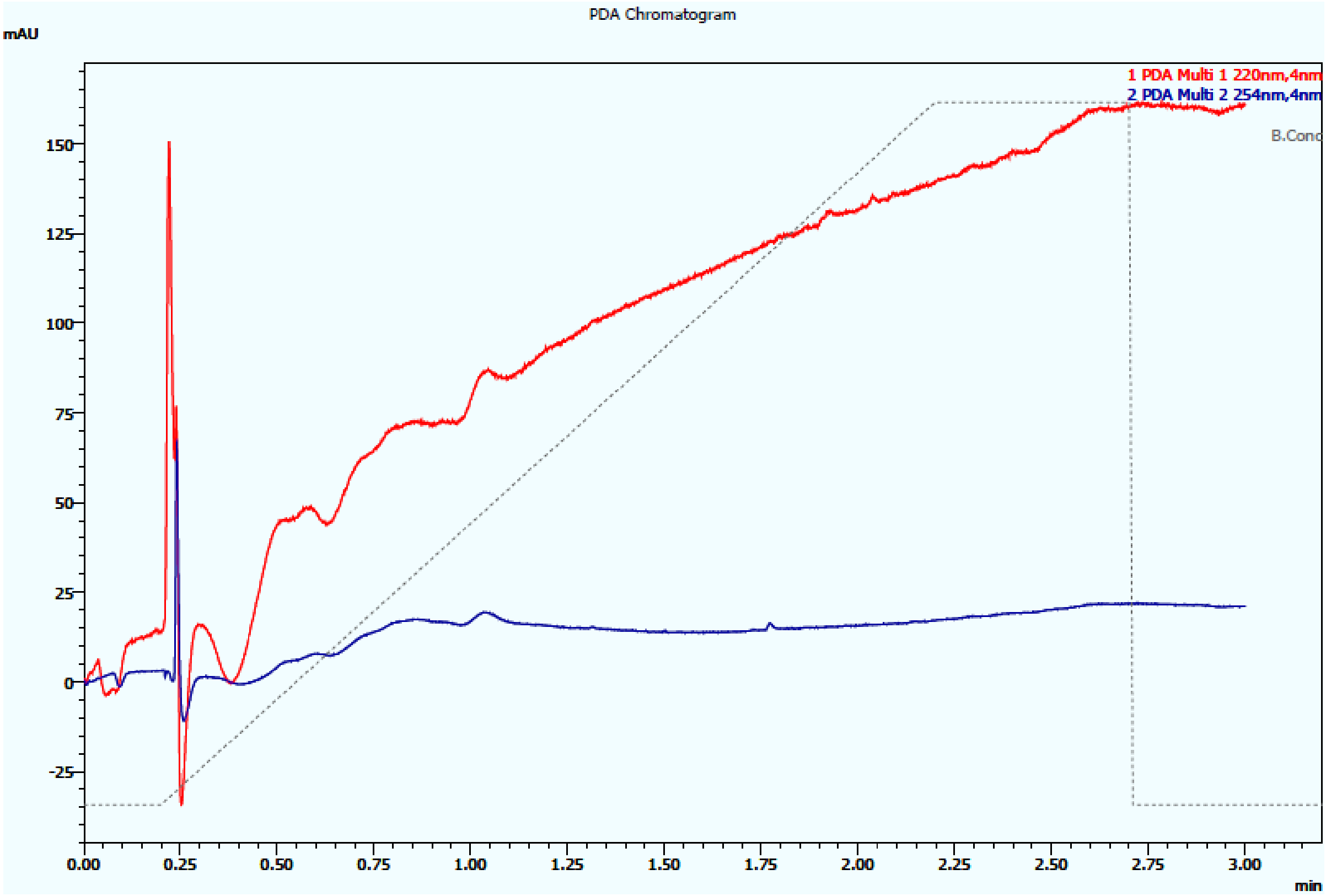

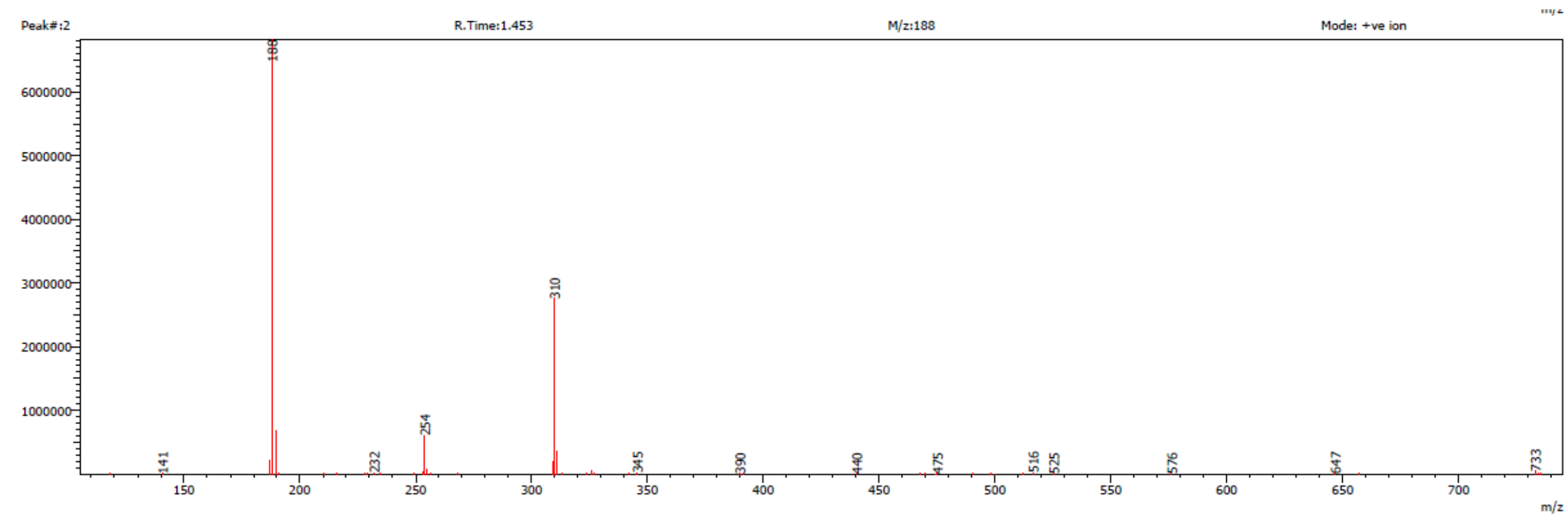

tert-butyl N-[2-[2-(2-allyloxyethoxy)ethoxy]ethyl]carbamate (3e)

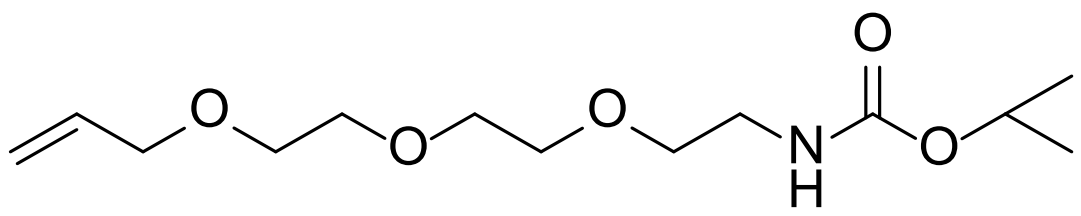

^{1}H NMR (400 MHz, $CDCl_3$, 303K)

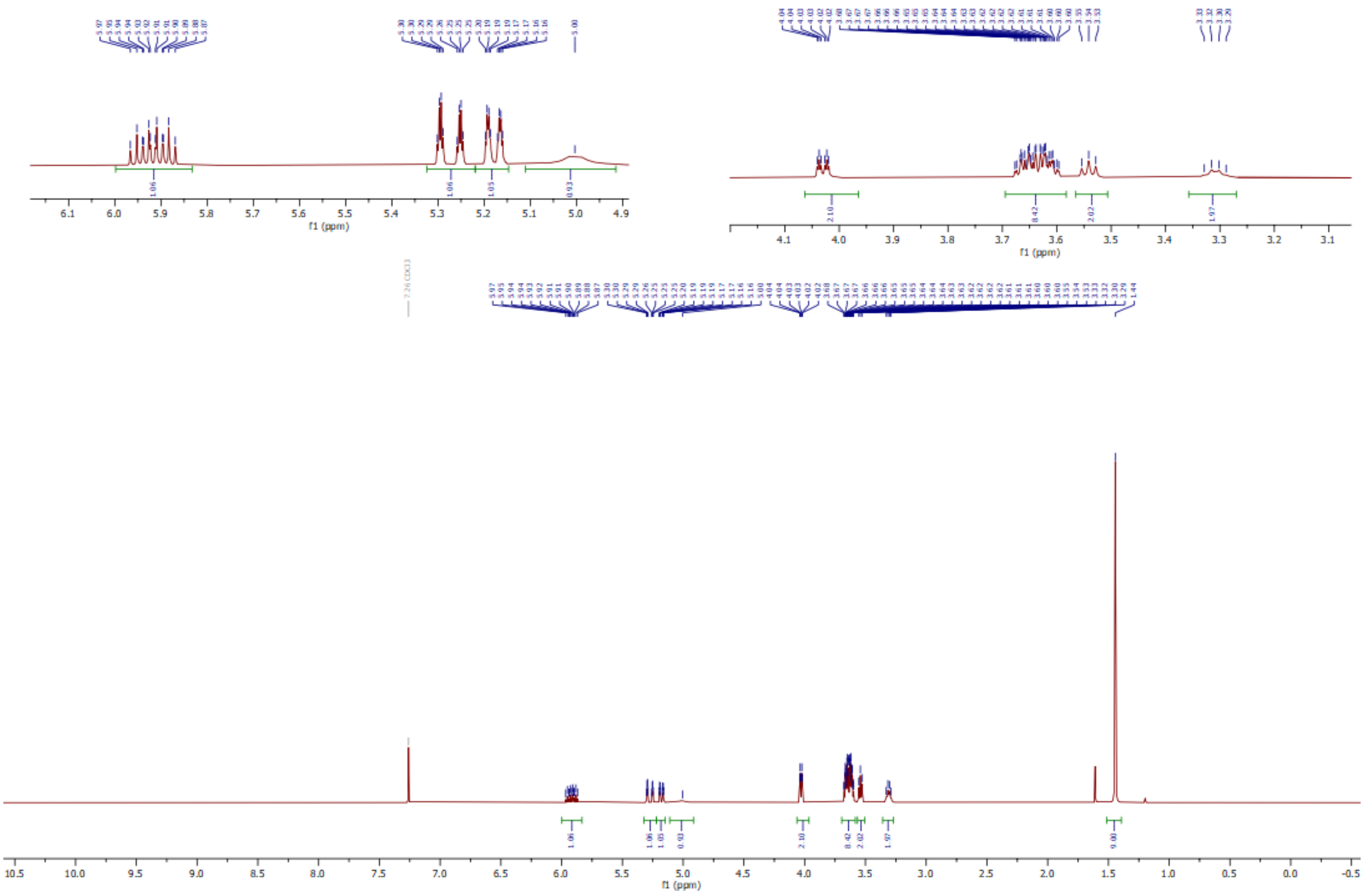

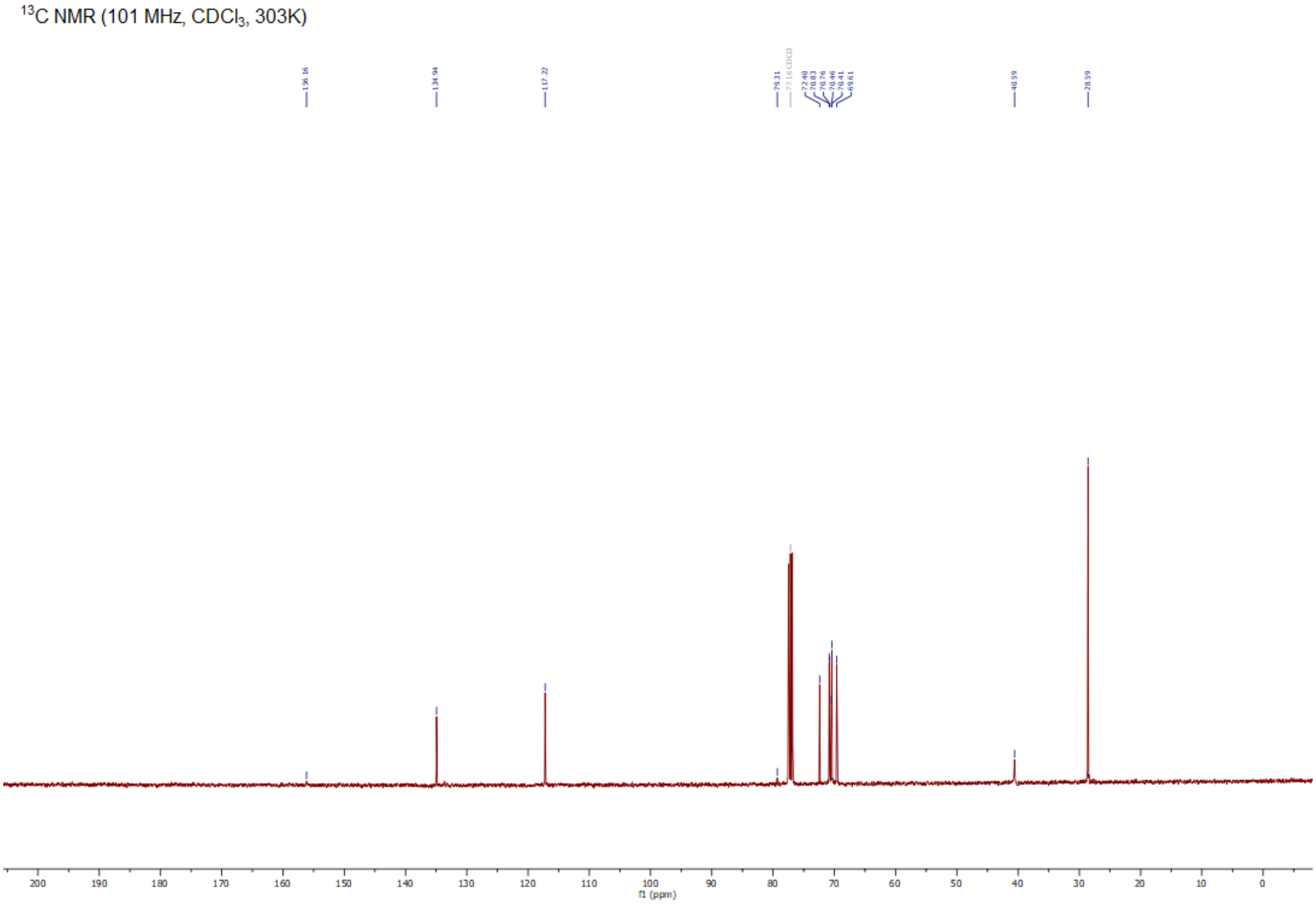

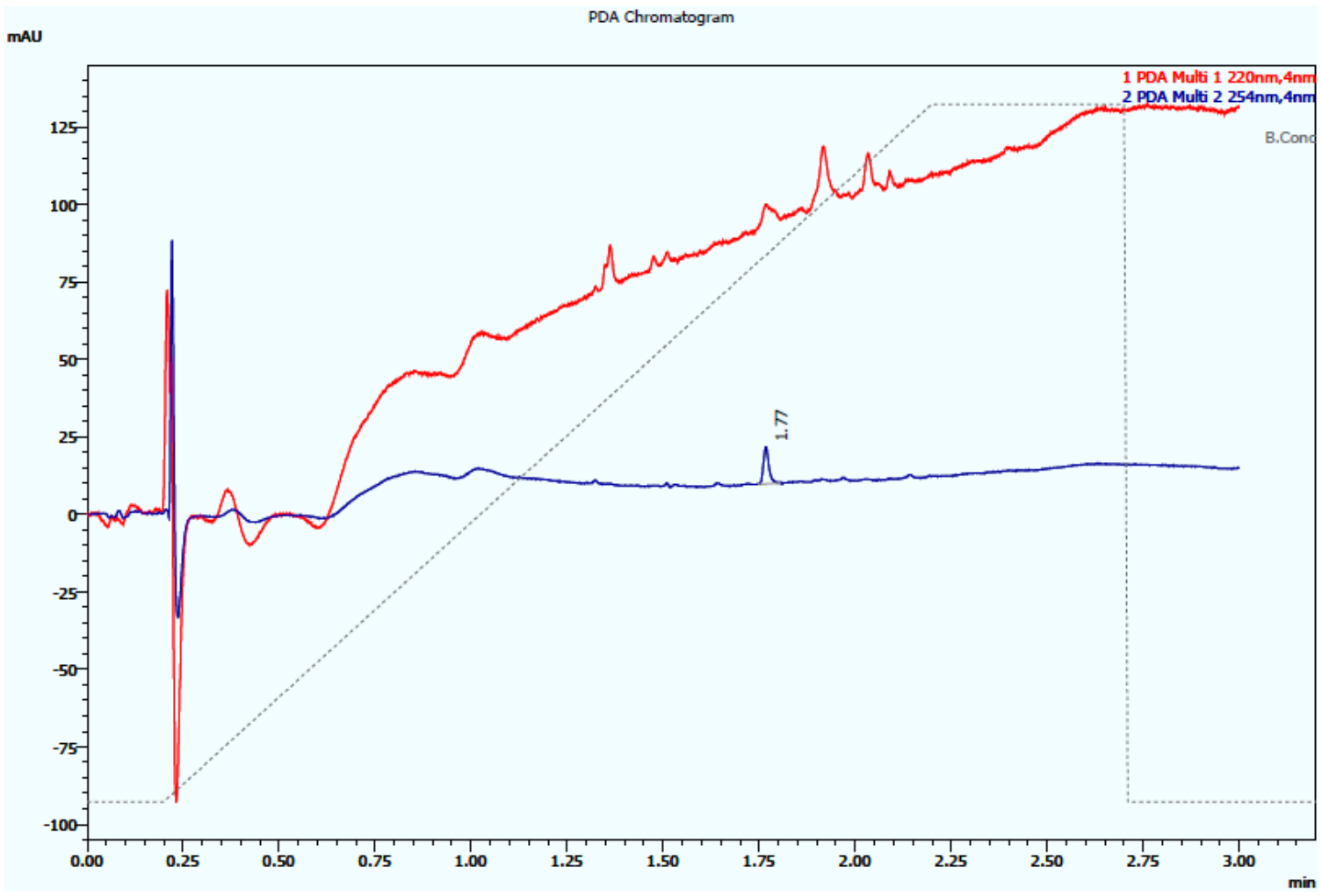

Product is not UV active; UV peaks in above chromatogram due to column contamination.

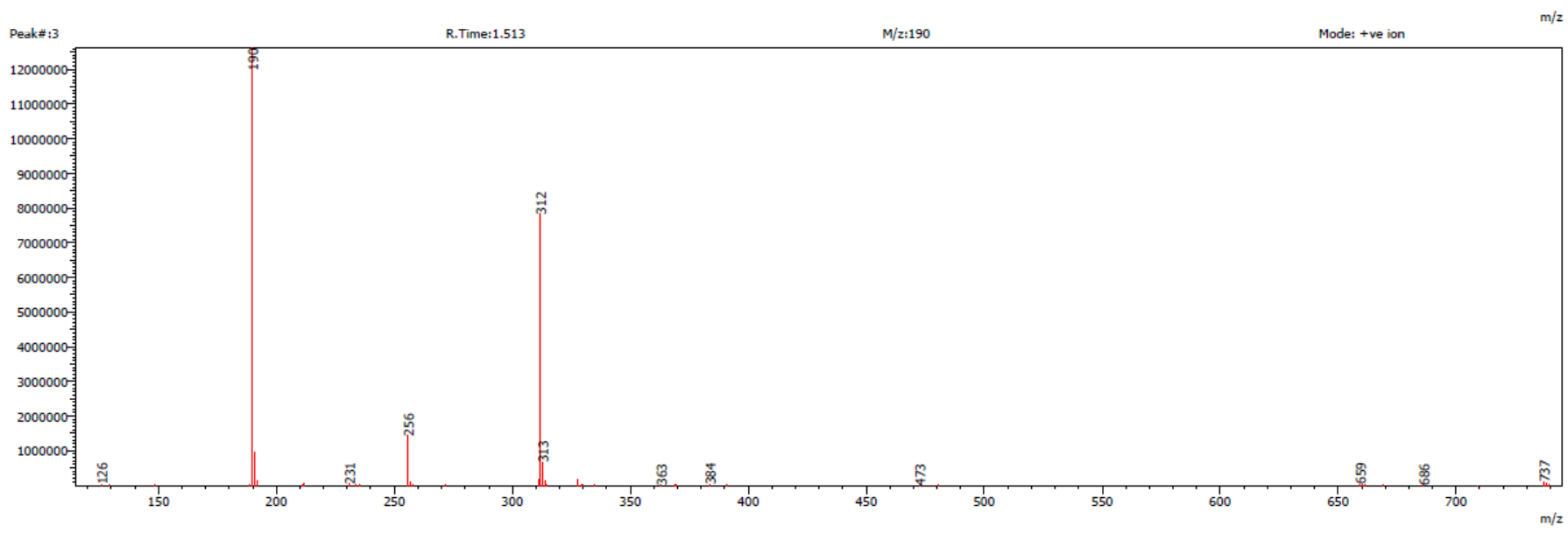

tert-butyl (2-(2-(2-(4-(4-(2-(pyridin-2-yloxy)propoxy)phenoxy)phenoxy)ethoxy)ethoxy)ethyl) carbamate (4)

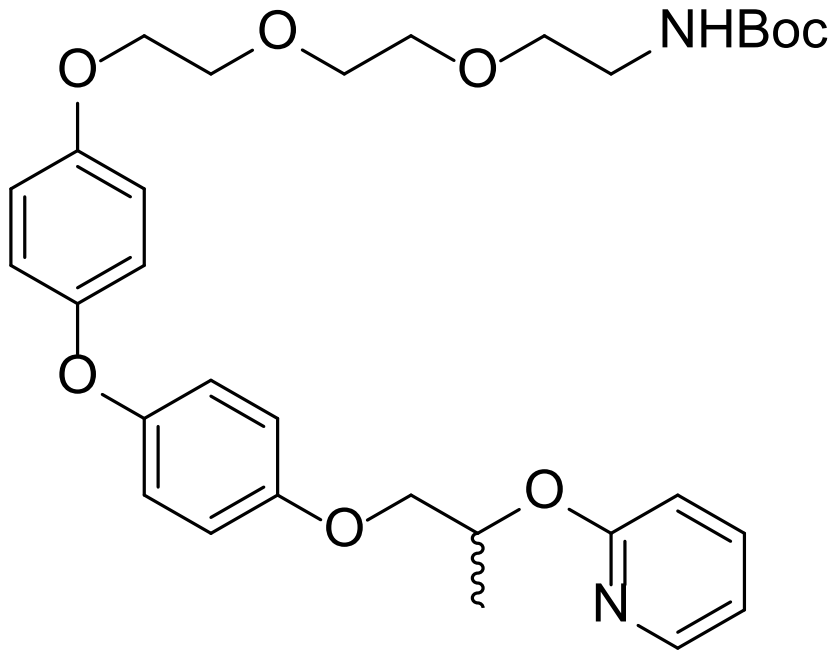

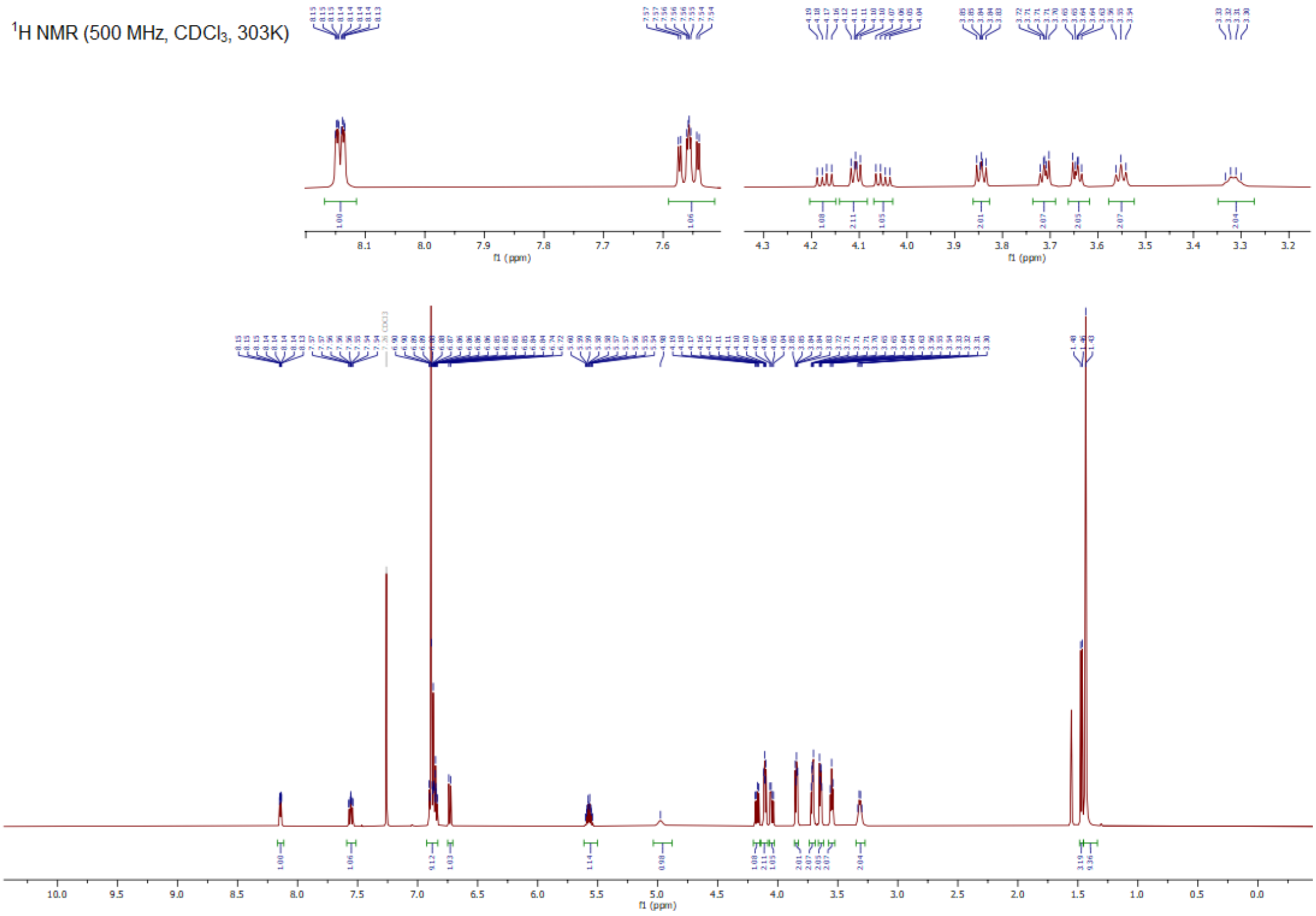

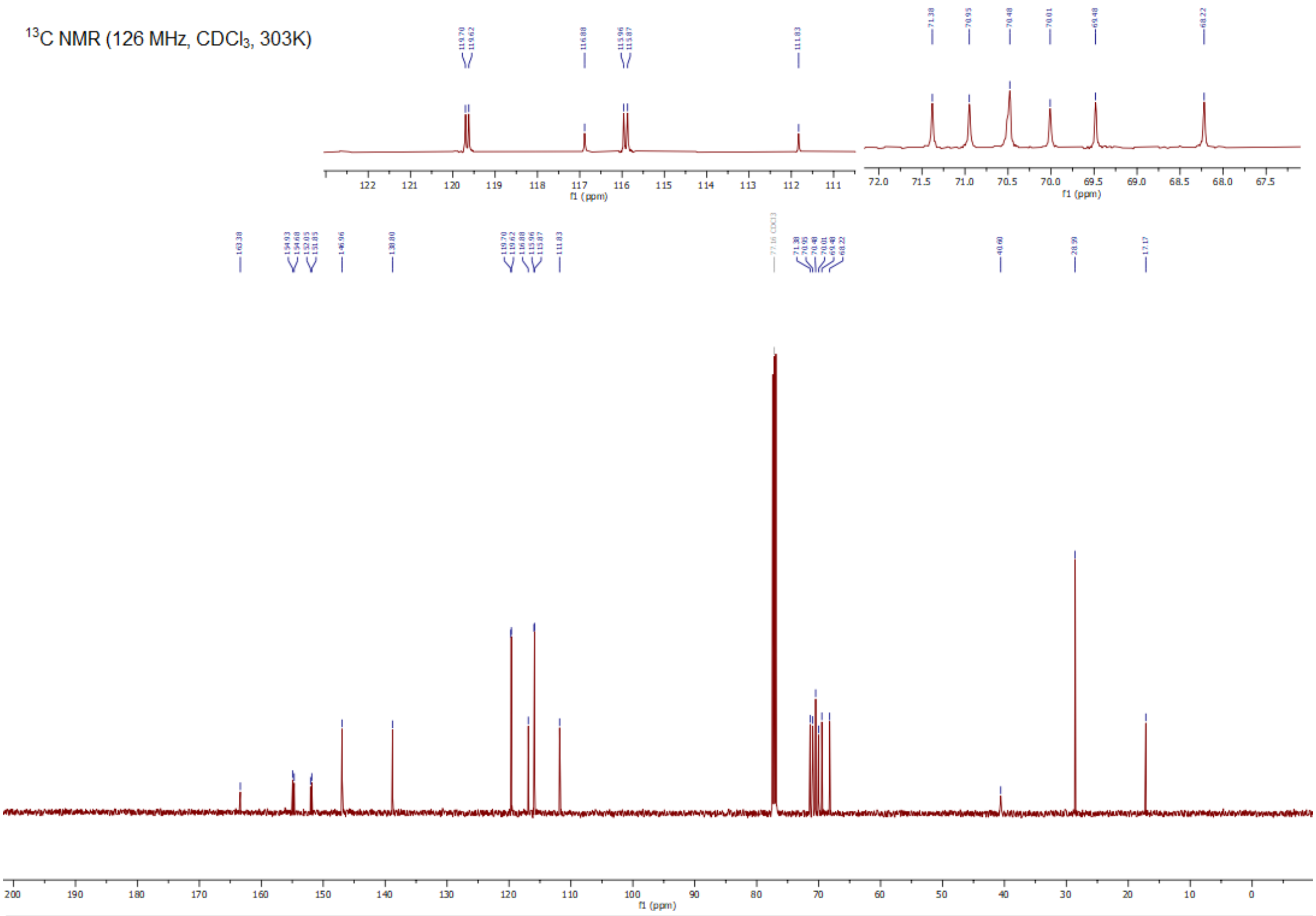

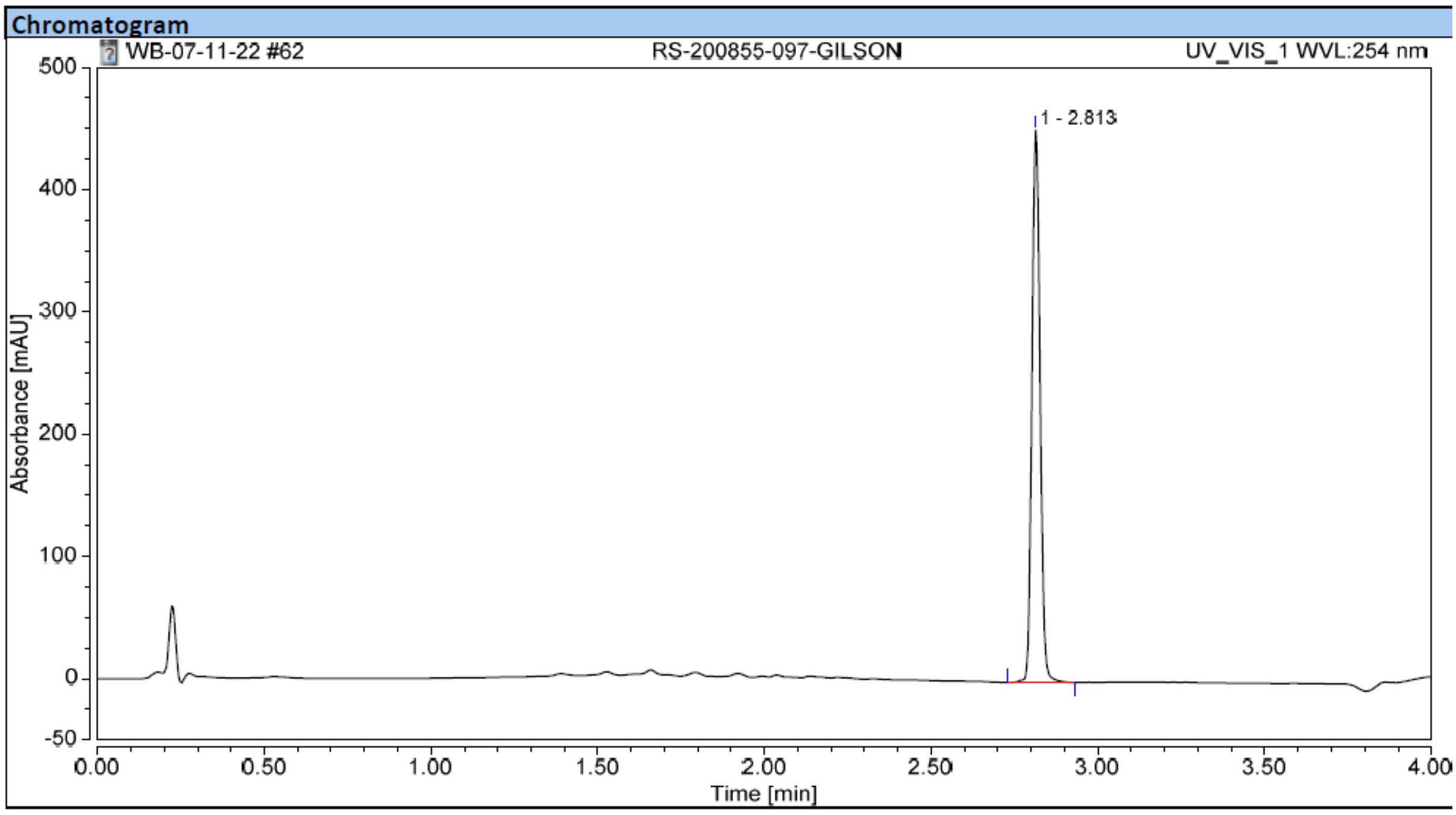

Integration Results

No.	Peak Name	Retention Time min	Area mAU*min	Height mAU	Relative Area %	Relative Height %	Amount n.a.
1		2.813	12.878	451.952	100.00	100.00	n.a.
**Total:**			**12.878**	**451.952**	**100.00**	**100.00**	

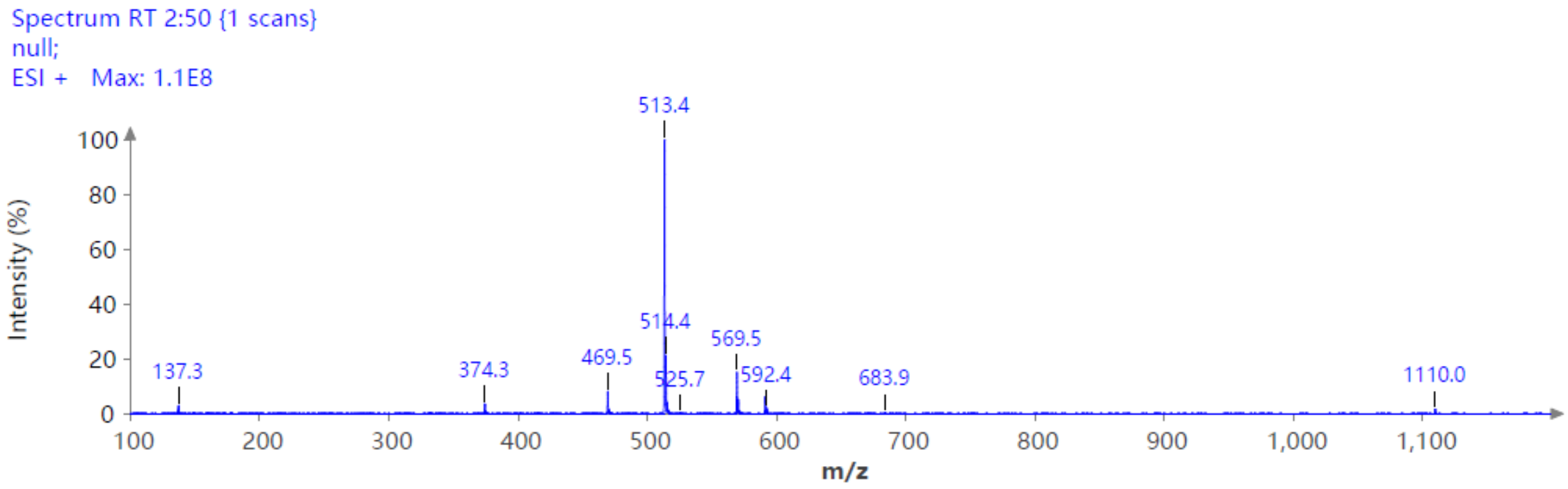

tert-butyl (2-(2-(2-((4-(4-(2-(pyridin-2-yloxy)propoxy)phenoxy)phenyl)amino)ethoxy)ethoxy)ethyl) carbamate (5)

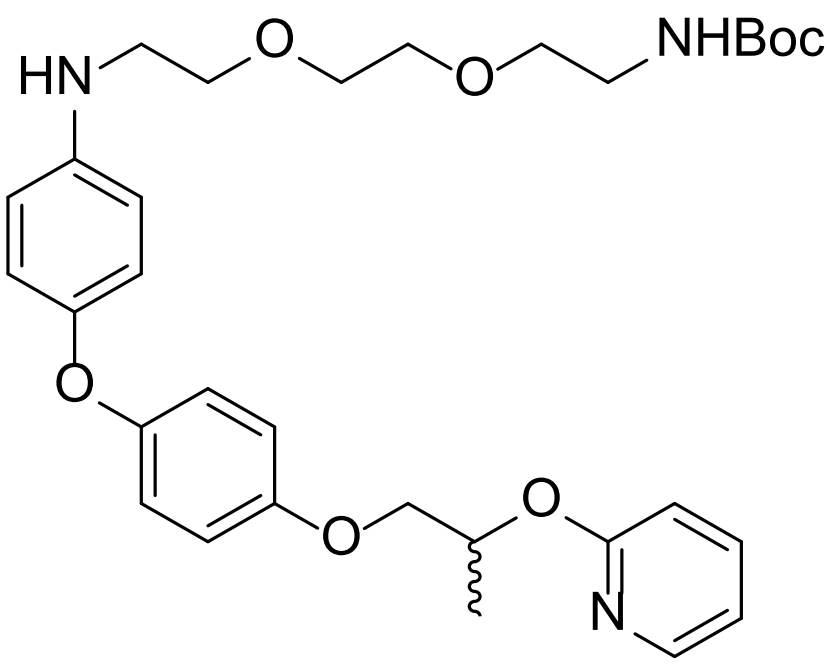

^{1}H NMR (500 MHz, $CDCl_3$, 303K)

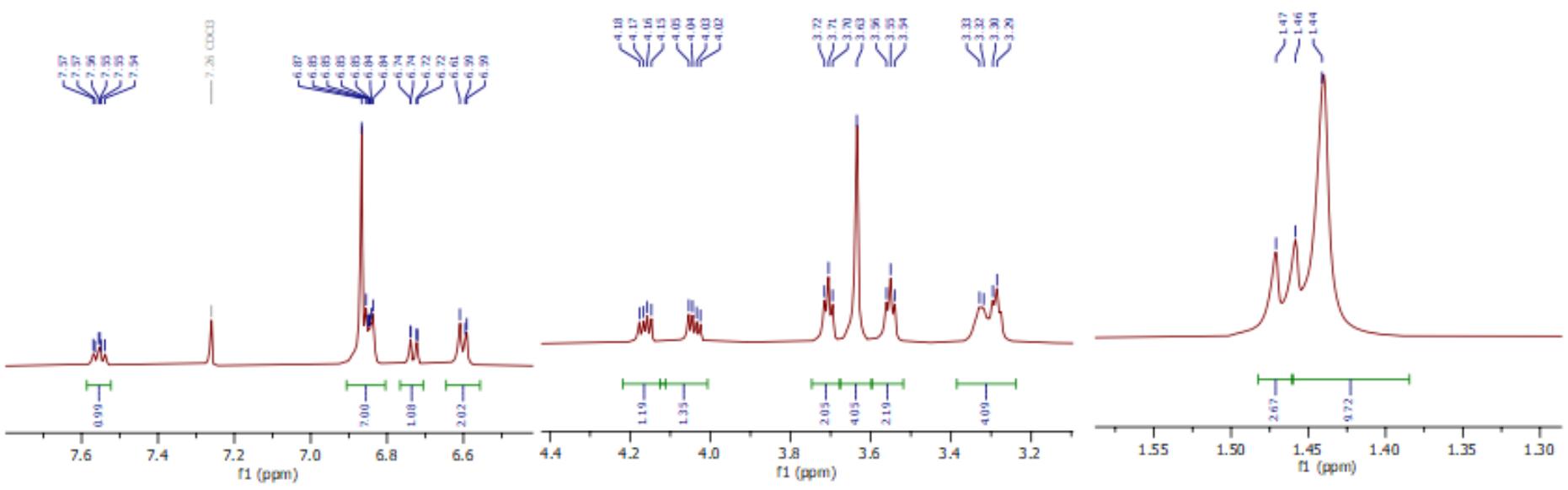

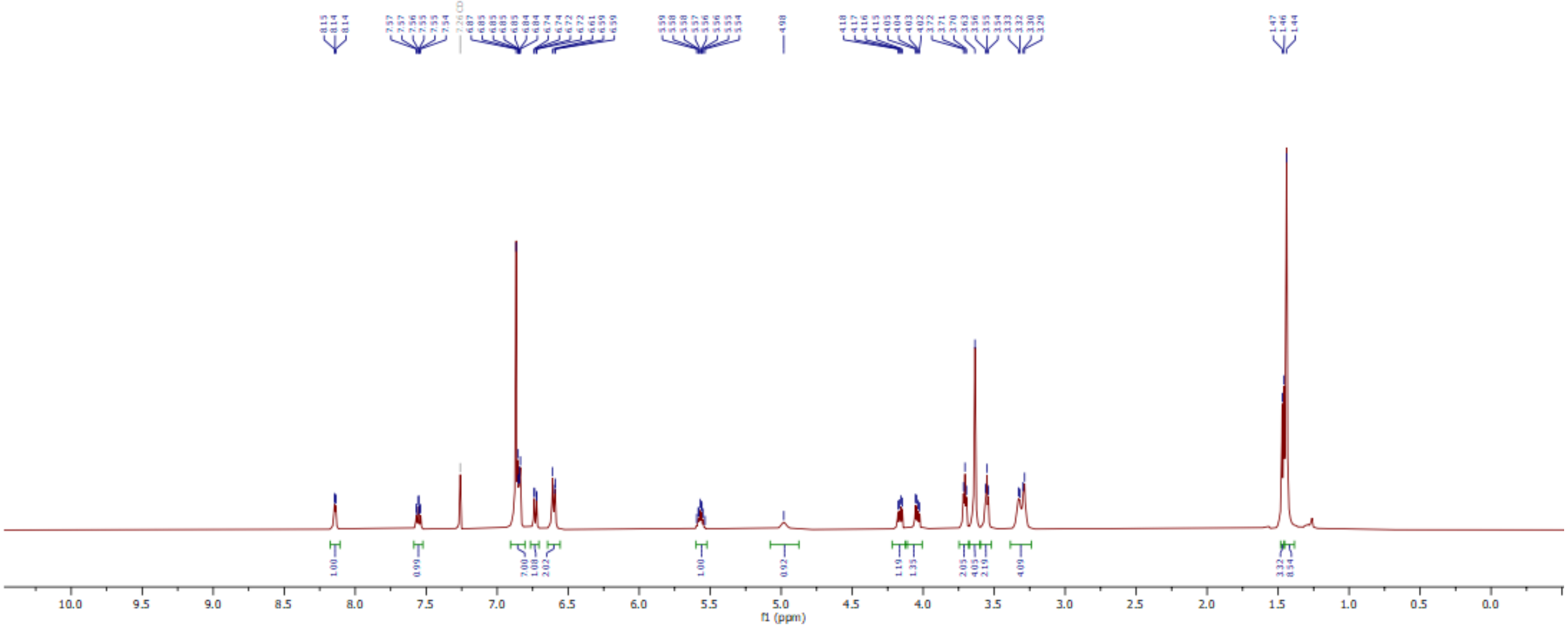

^{13}C NMR (126 MHz, $CDCl_3$, 303K)

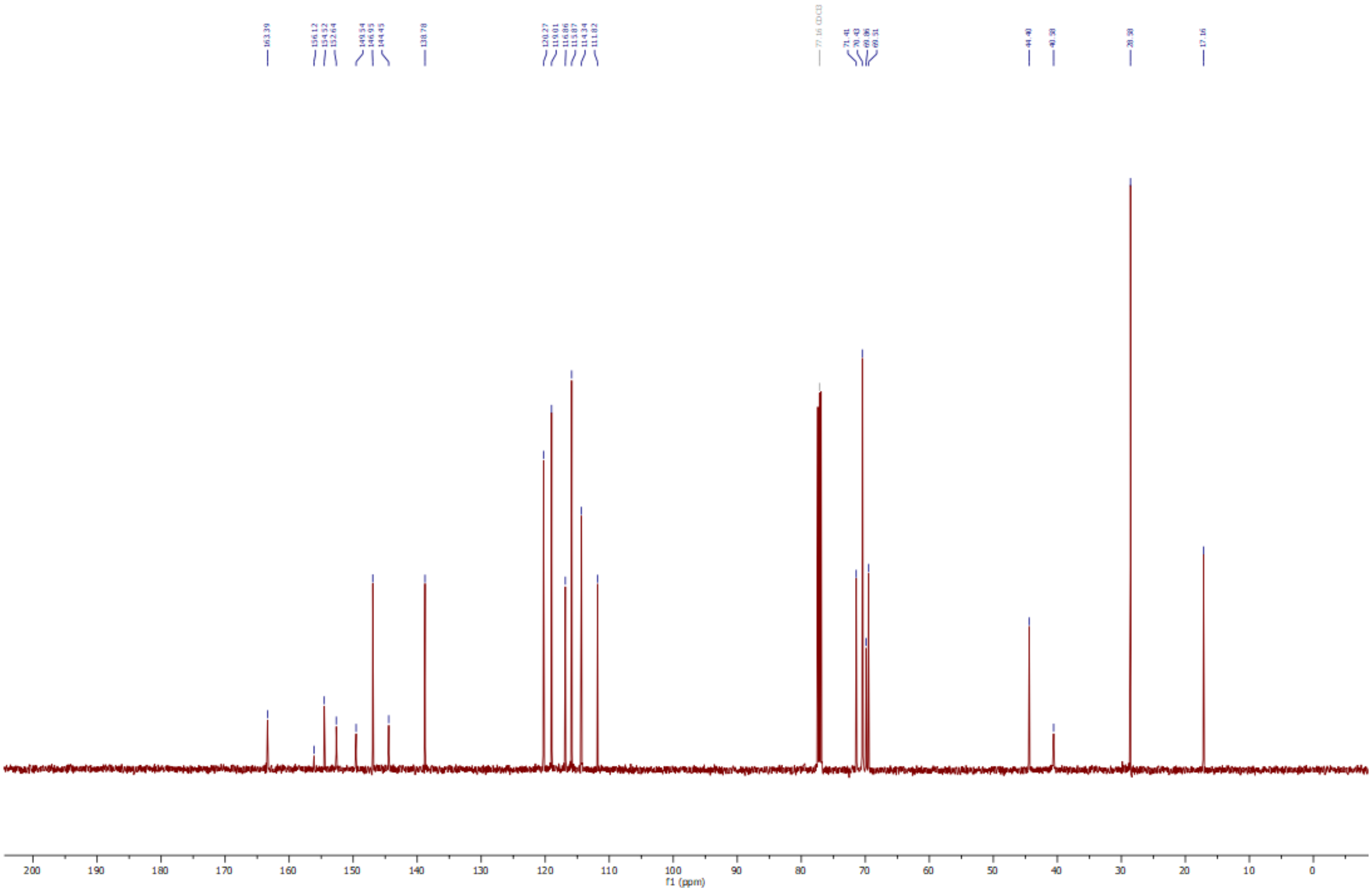

Chromatogram

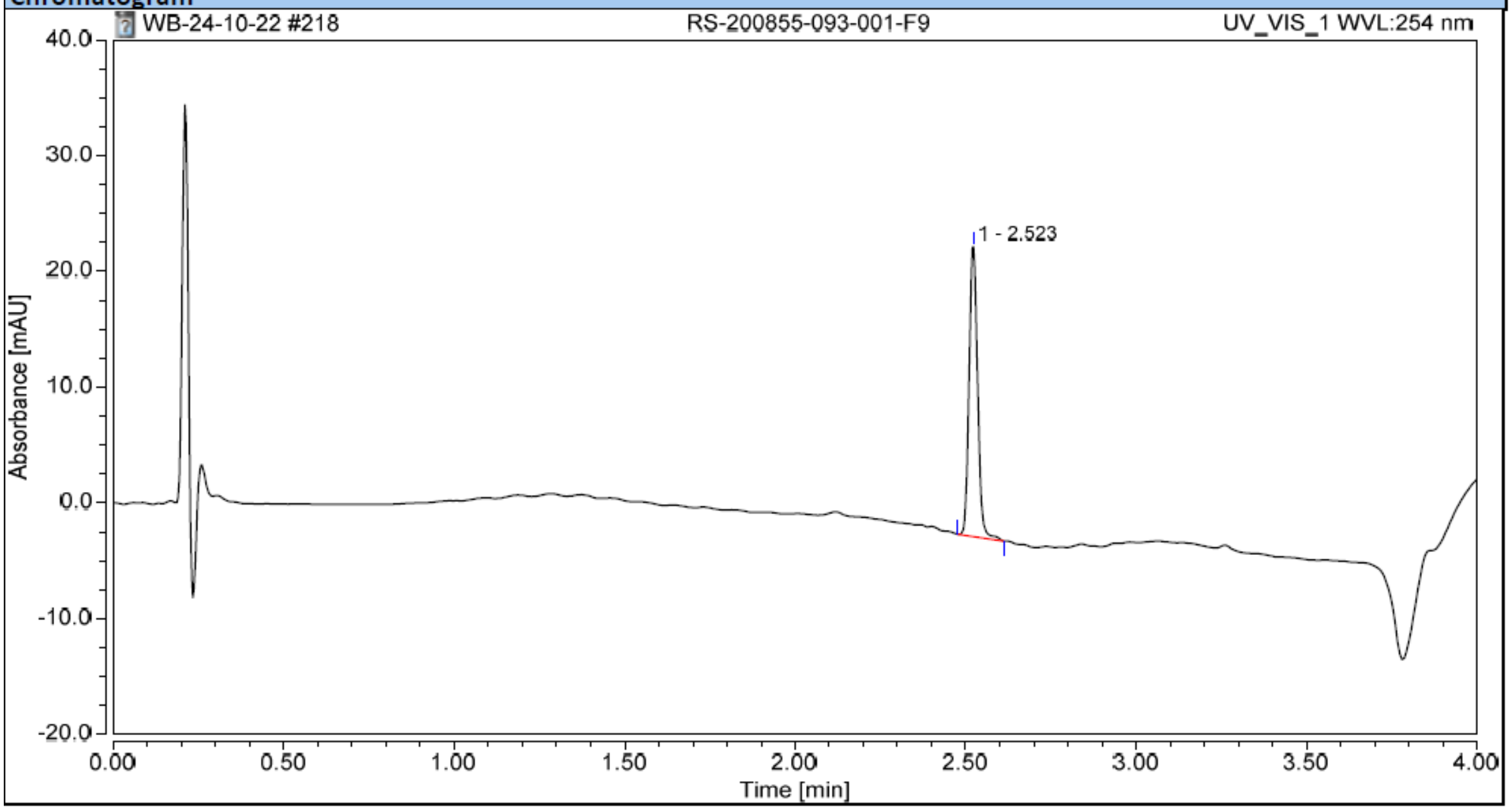

Integration Results

No.	Peak Name	Retention Time<br>min	Area<br>mAU*min	Height<br>mAU	Relative Area<br>%	Relative Height<br>%	Amount<br>n.a.
1		2.523	0.729	25.054	100.00	100.00	n.a.
**Total:**			**0.729**	**25.054**	**100.00**	**100.00**	

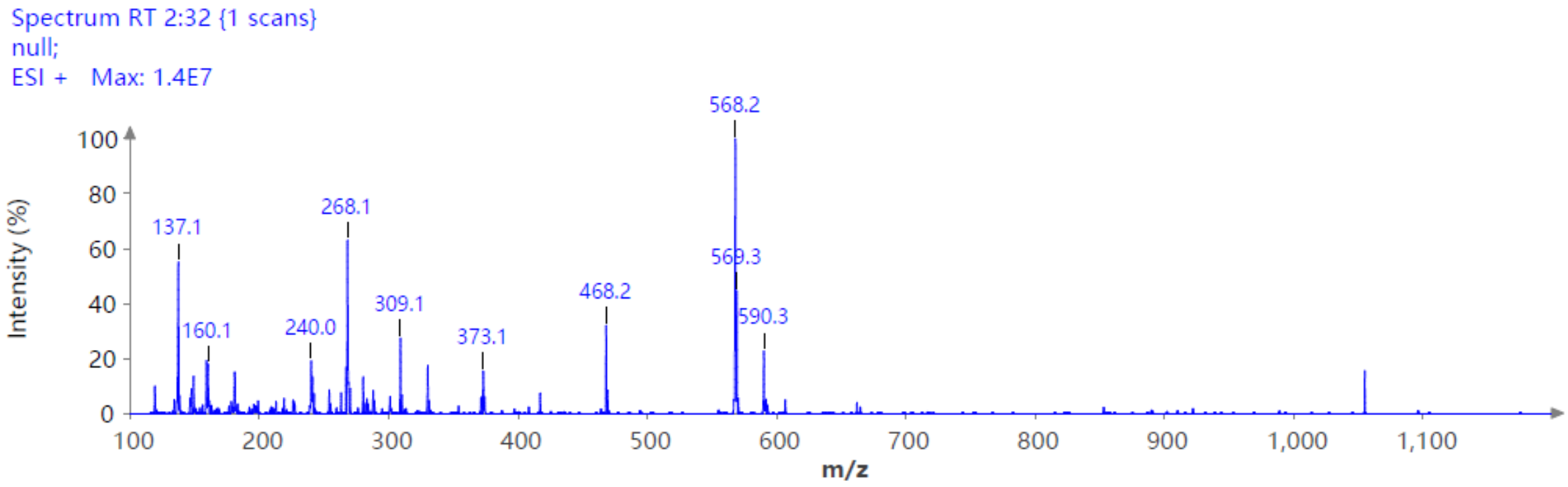

tert-butyl (2-(2-(methyl(4-(4-(2-(pyridin-2-yloxy)propoxy)phenoxy)phenyl)amino)ethoxy)ethyl) carbamate (6)

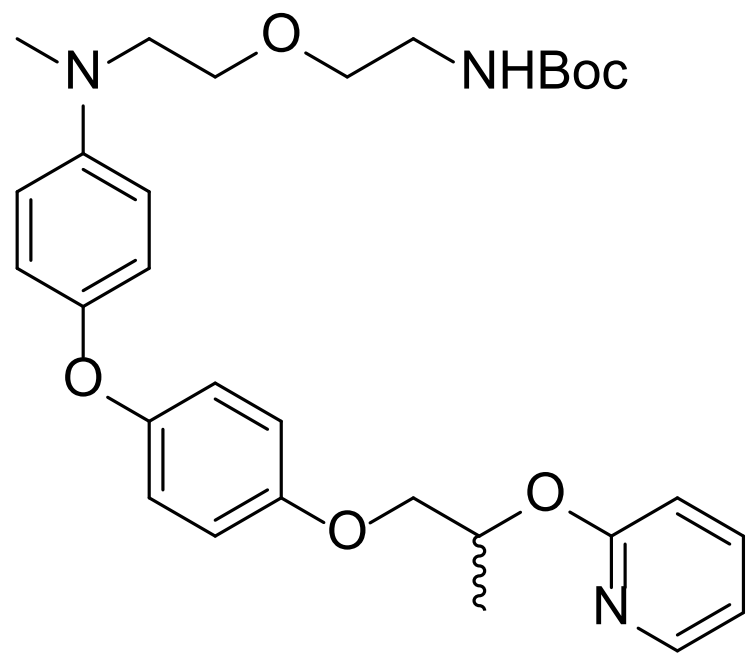

^{1}H NMR (500 MHz, $CDCl_3$, 303K)

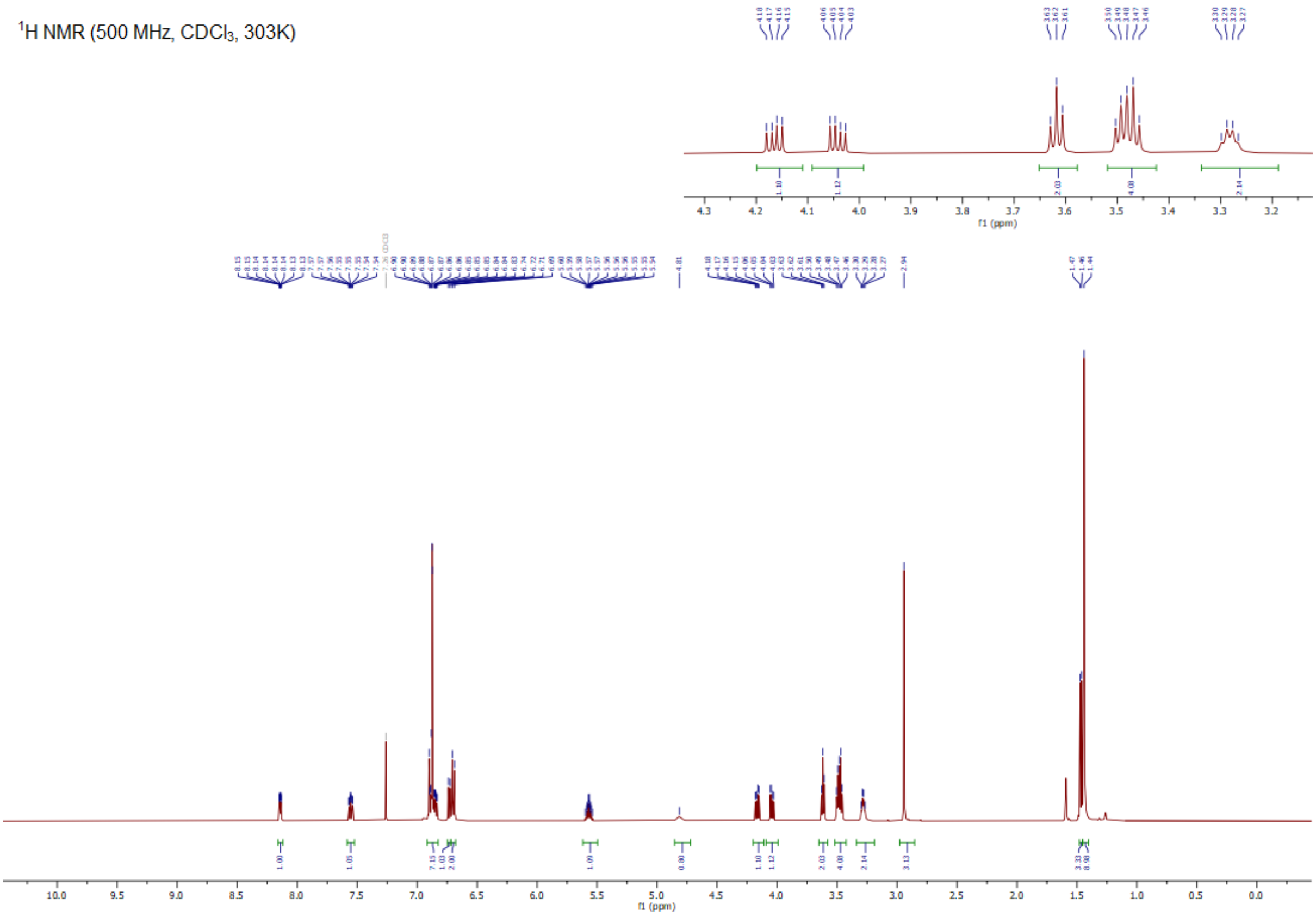

^{13}C NMR (126 MHz, $CDCl_3$, 303K)

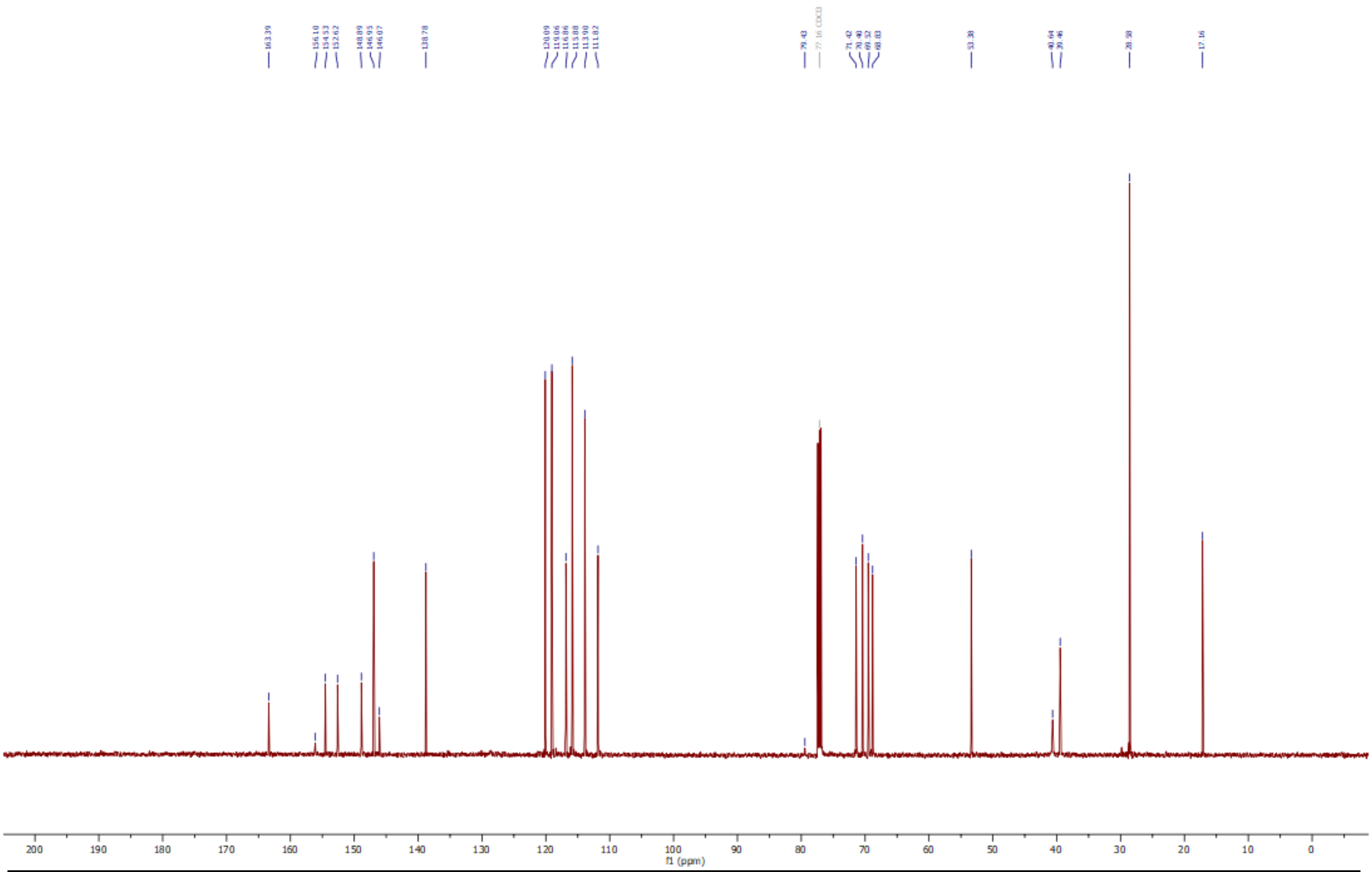

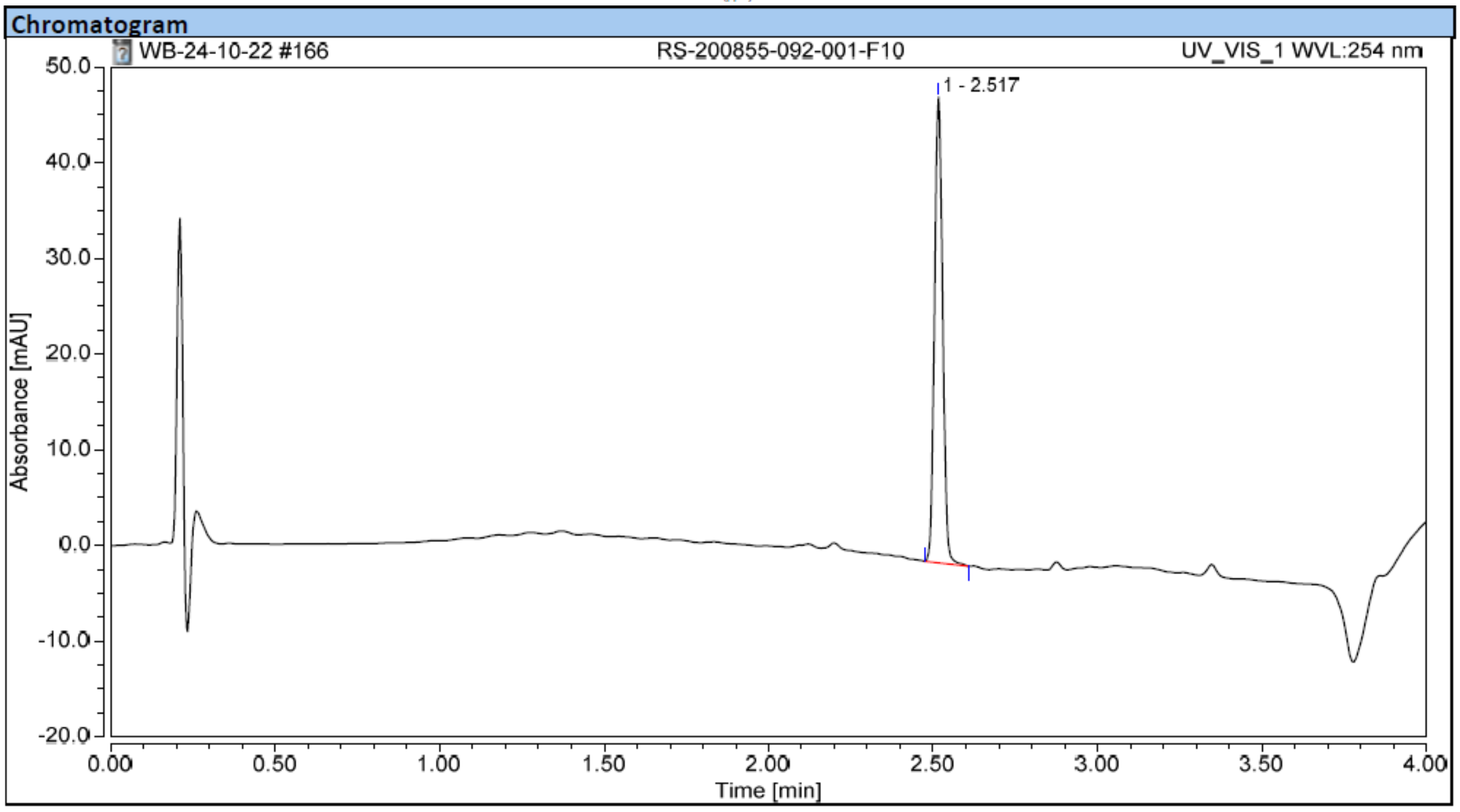

Integration Results

No.	Peak Name	Retention Time min	Area mAU*min	Height mAU	Relative Area %	Relative Height %	Amount n.a.
1		2.517	1.401	48.653	100.00	100.00	n.a.
**Total:**			**1.401**	**48.653**	**100.00**	**100.00**	

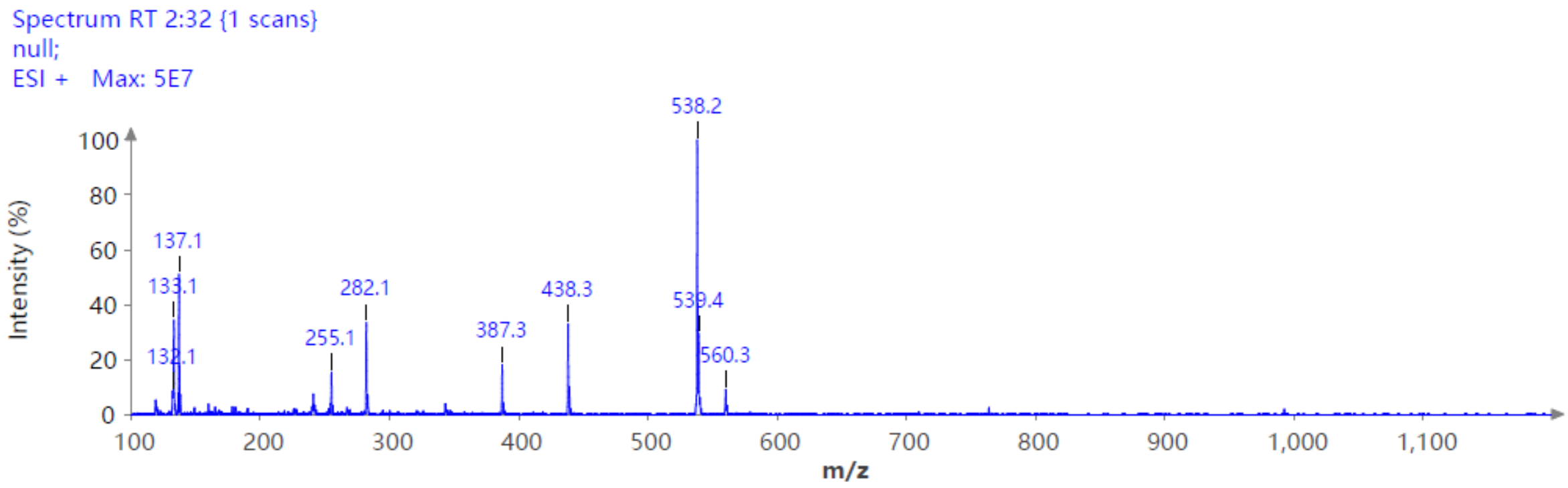

tert-butyl (2-(2-(2-((3-(4-(4-(2-(pyridin-2-yloxy)propoxy)phenoxy)phenyl)prop-2-yn-1-yl)oxy)ethoxy)ethoxy)ethyl) carbamate (7)

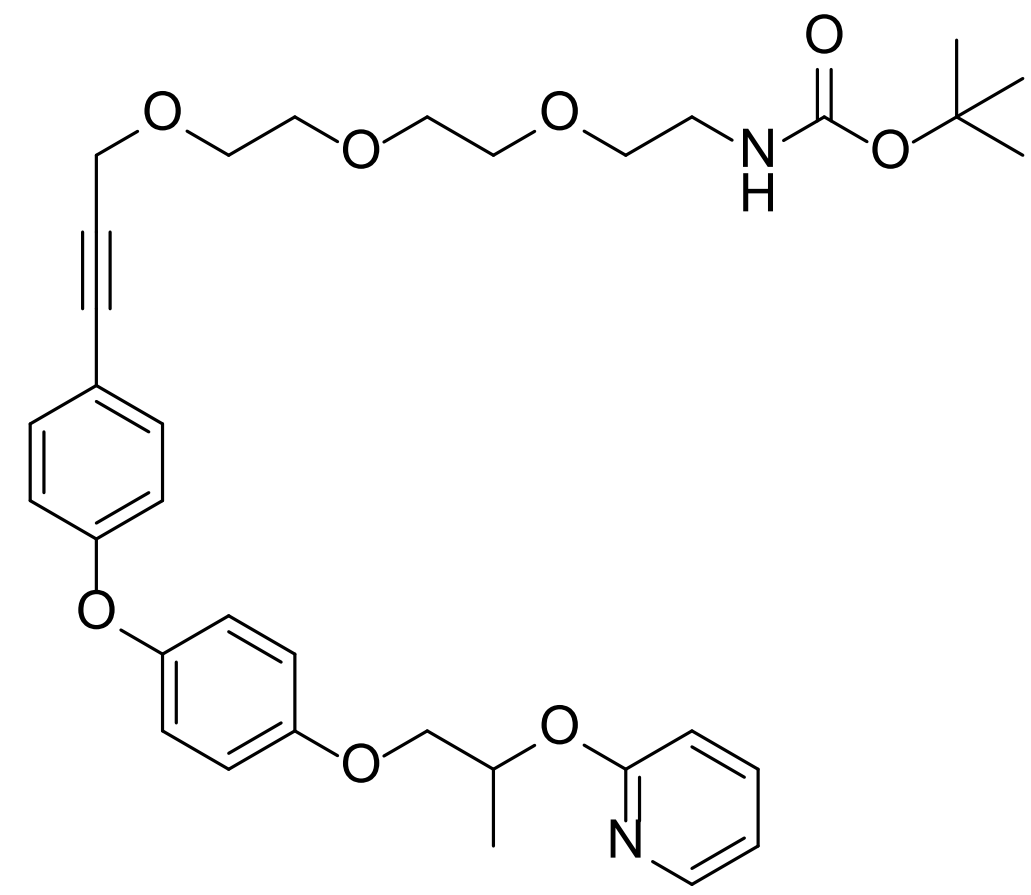

^{1}H NMR (500 MHz, $CDCl_3$, 303K)

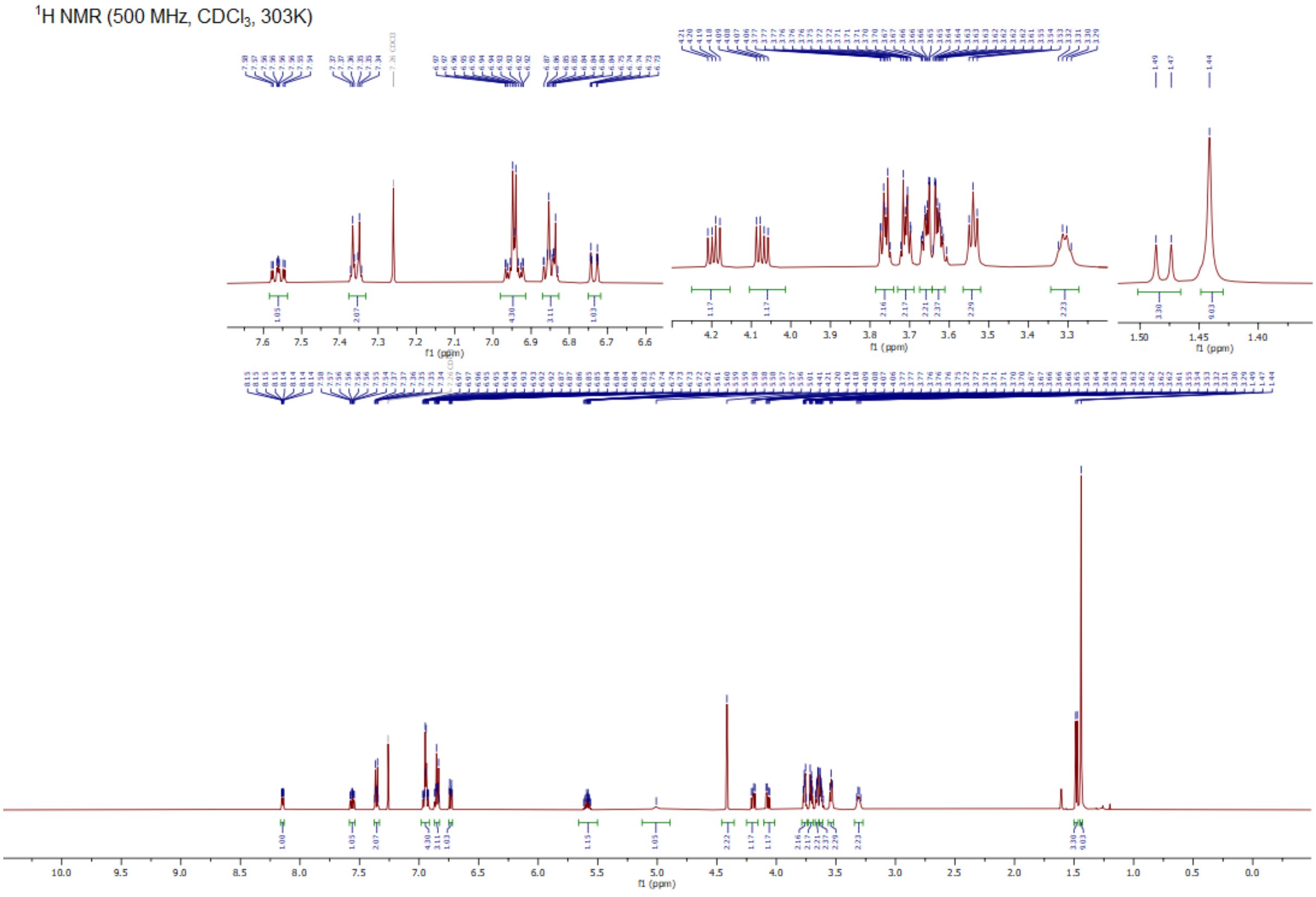

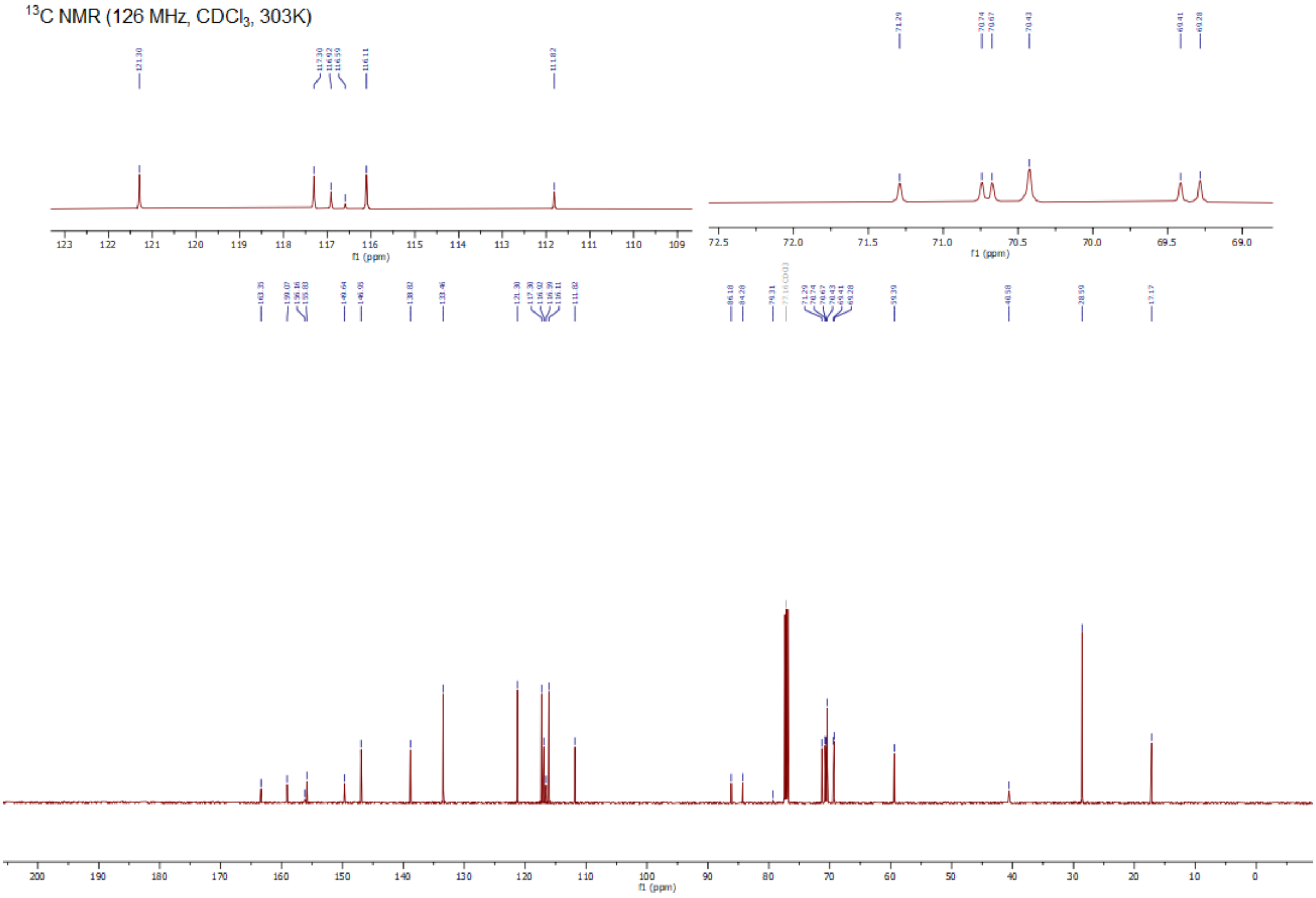

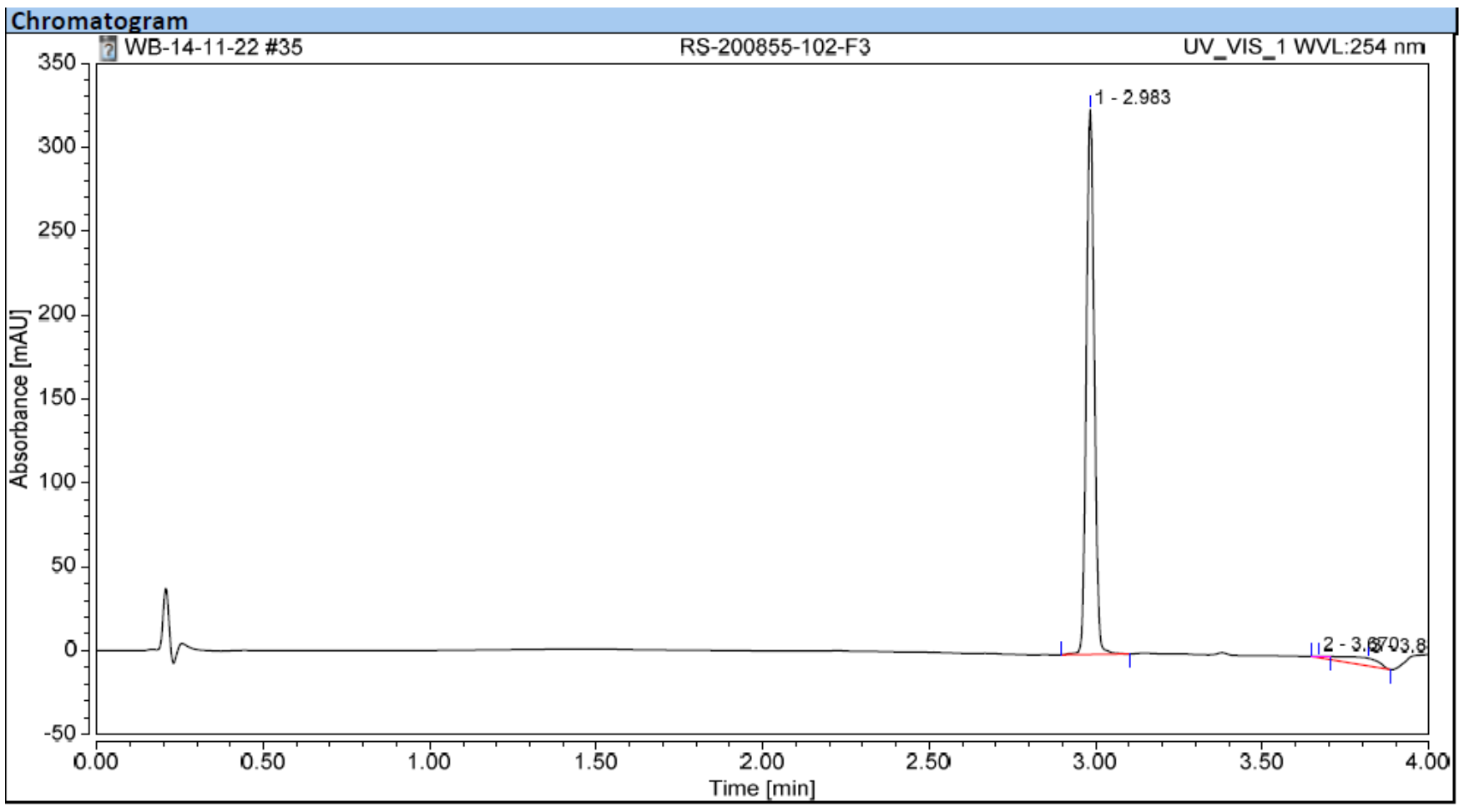

Integration Results

No.	Peak Name	Retention Time min	Area mAU*min	Height mAU	Relative Area %	Relative Height %	Amount n.a.
1		2.983	8.976	324.744	93.63	98.59	n.a.
2		3.670	0.002	0.077	0.02	0.02	n.a.
3		3.817	0.609	4.565	6.35	1.39	n.a.
**Total:**			**9.587**	**329.386**	**100.00**	**100.00**	

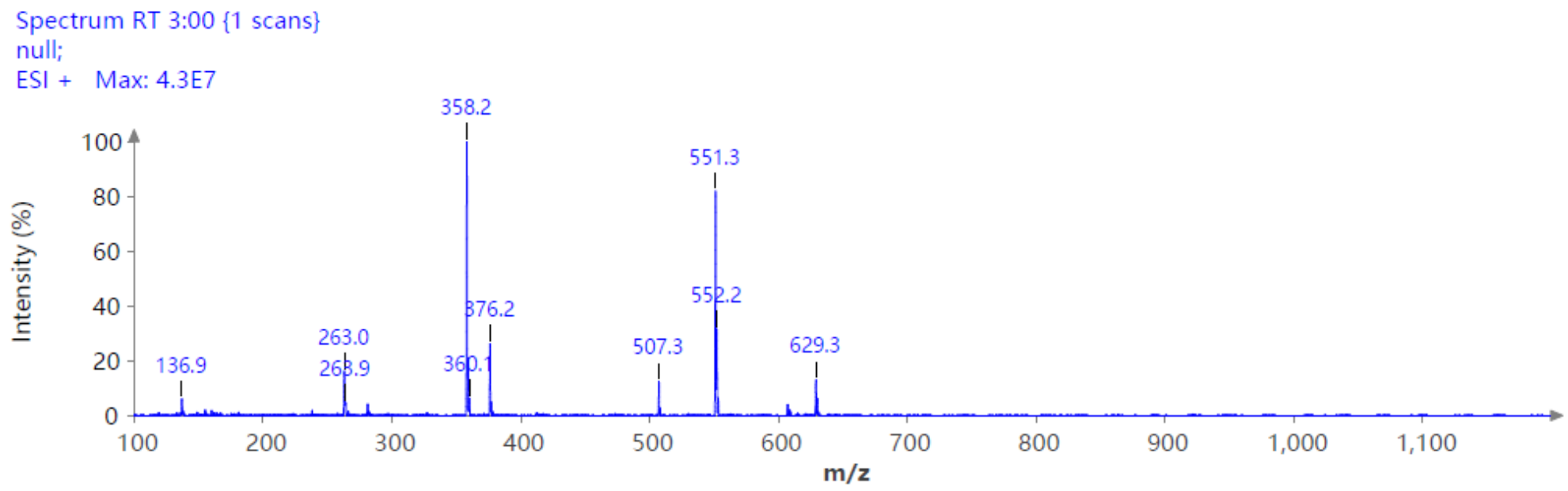

tert-butyl (2-(2-(2-((3-(4-(4-(2-(pyridin-2-yloxy)propoxy)phenoxy)phenyl)allyl)oxy)ethoxy)ethoxy)ethyl) carbamate (8)

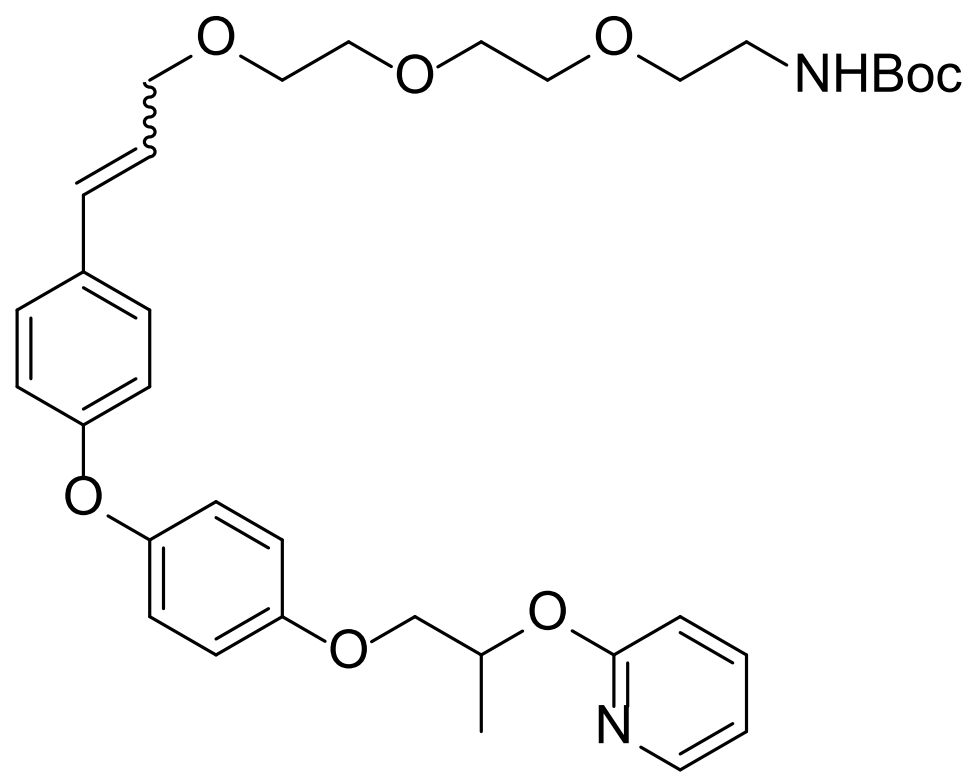

^{1}H NMR (500 MHz, $CDCl_3$, 303K)

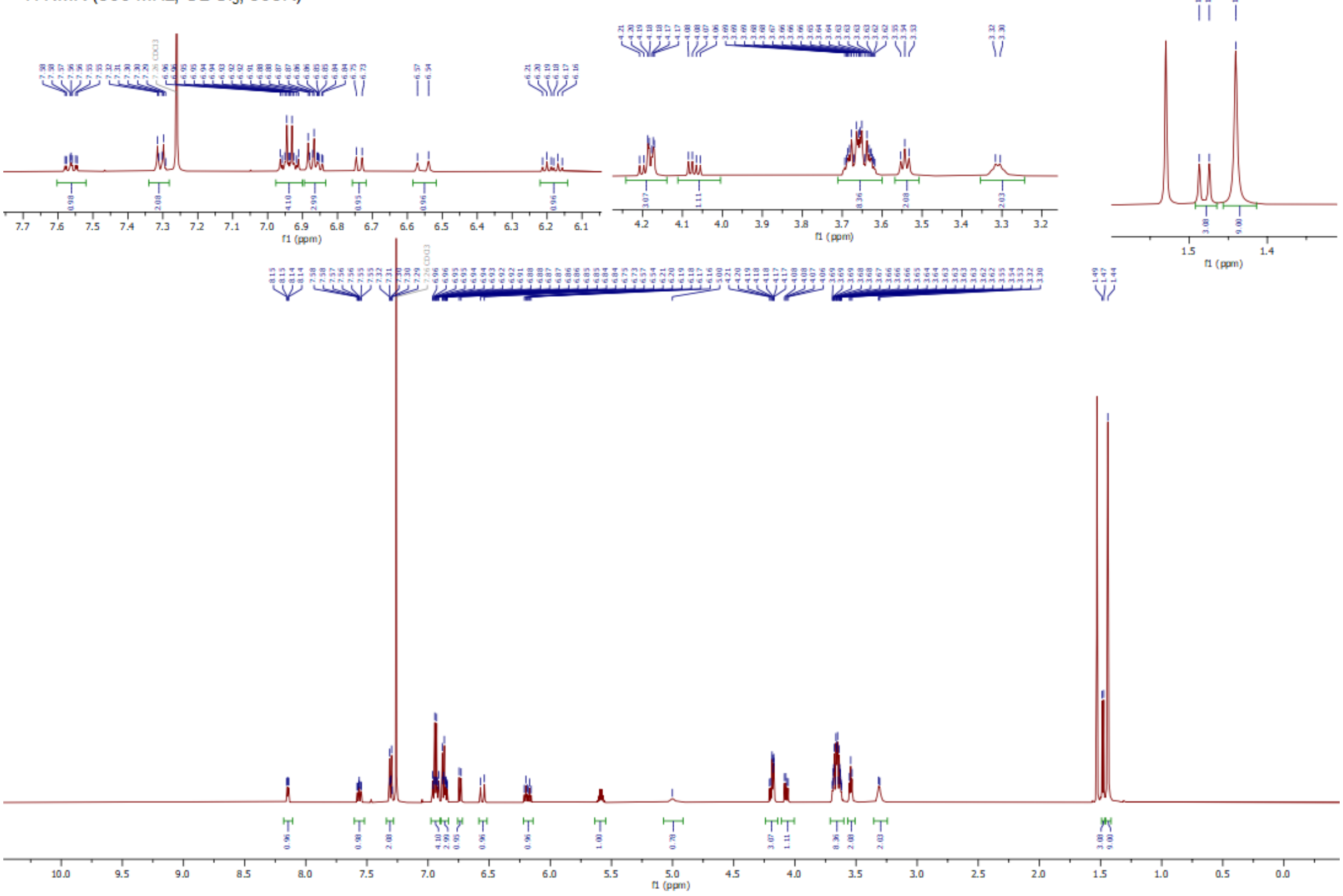

^{13}C NMR (126 MHz, $CDCl_3$, 303K)

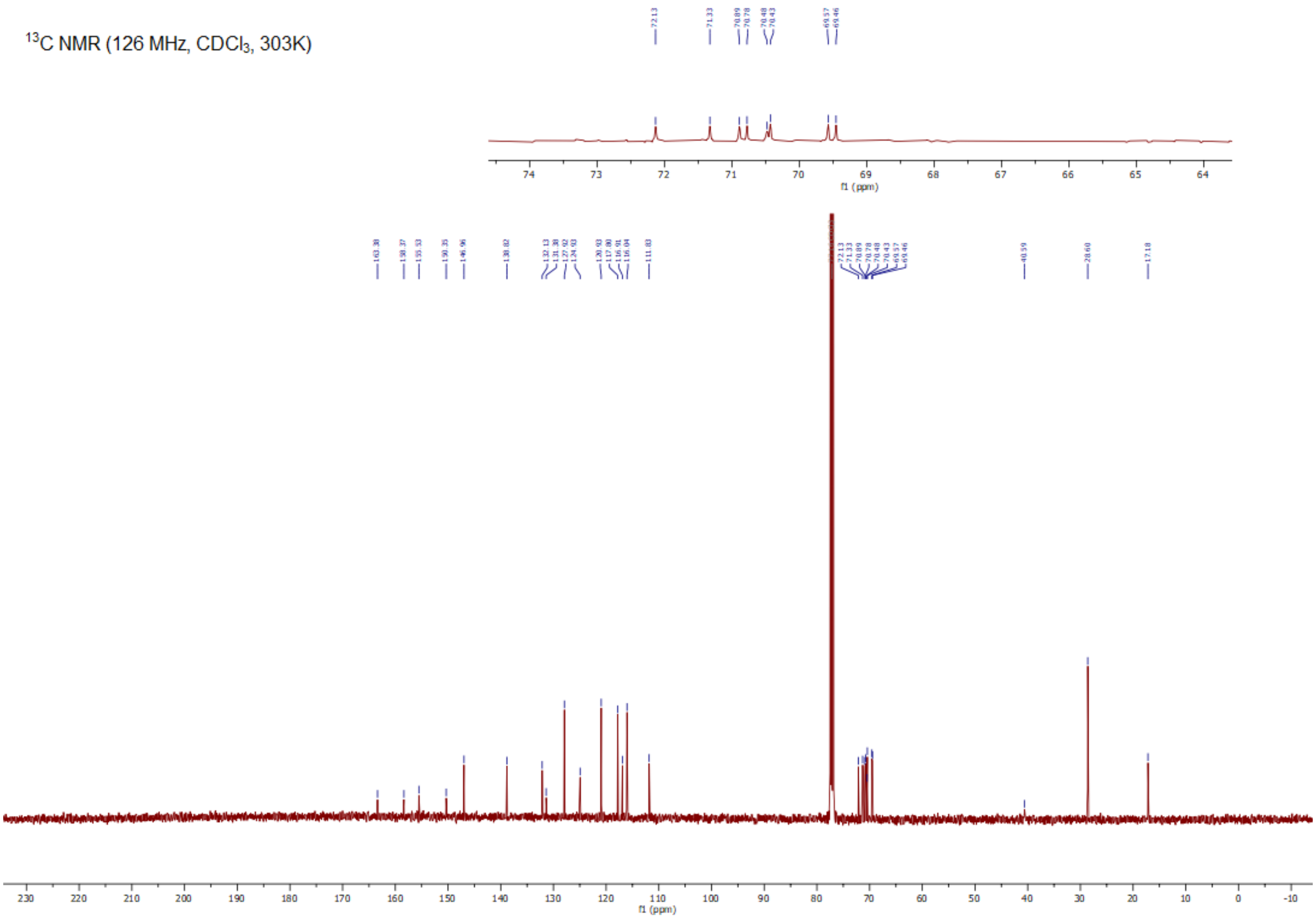

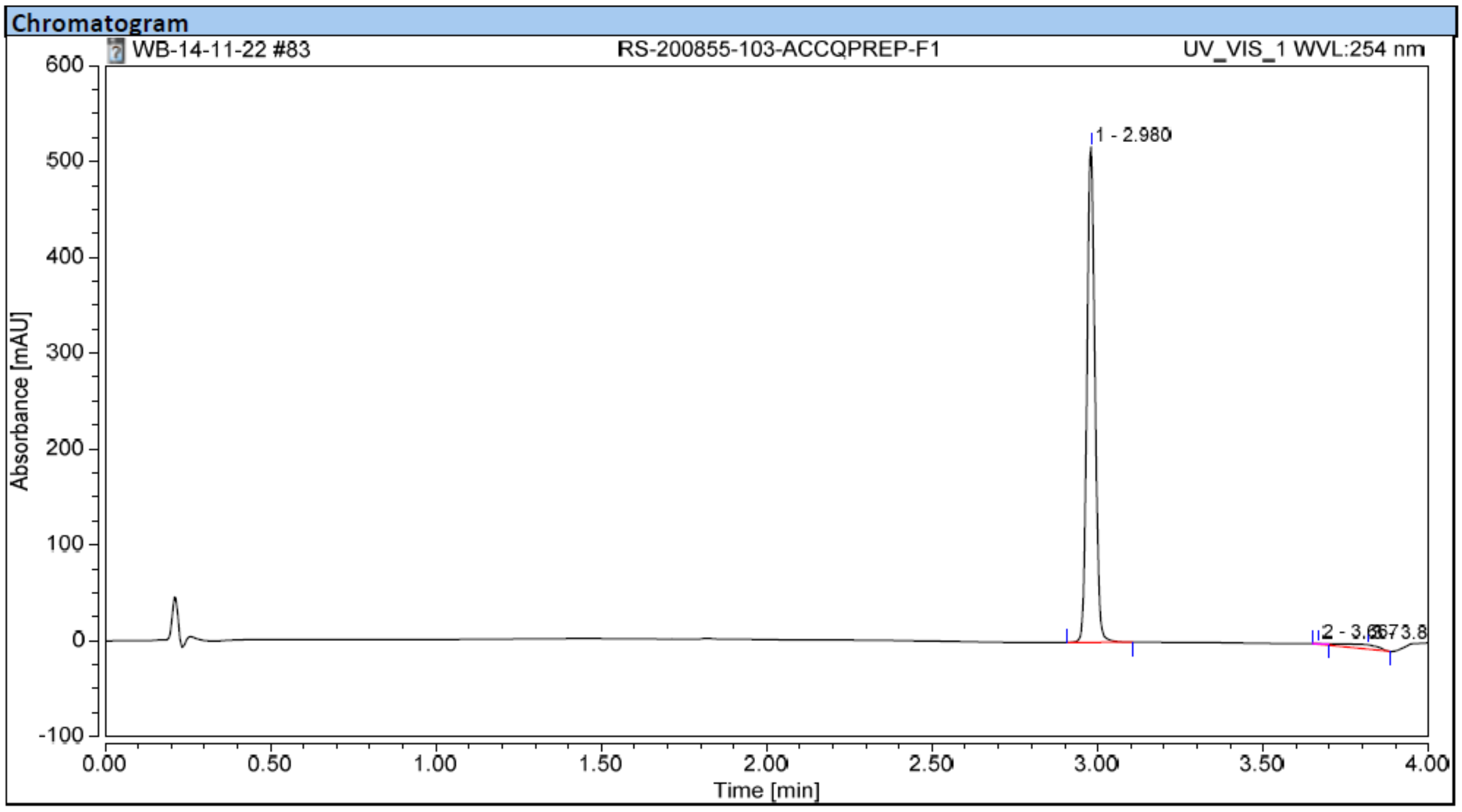

Integration Results

No.	Peak Name	Retention Time min	Area mAU*min	Height mAU	Relative Area %	Relative Height %	Amount n.a.
1		2.980	14.625	516.907	96.00	99.11	n.a.
2		3.667	0.002	0.071	0.01	0.01	n.a.
3		3.817	0.607	4.579	3.99	0.88	n.a.
**Total:**			**15.234**	**521.557**	**100.00**	**100.00**	

5-(4-(2-morpholino-4-oxo-4H-chromen-8-yl)phenyl)-5H-thianthren-5-ium trifluoromethanesulfonate (33)

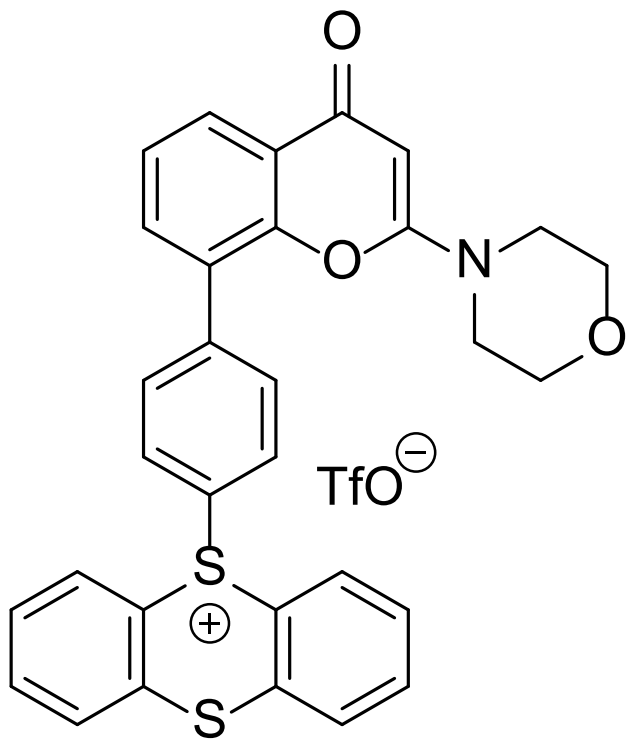

1H NMR (400 MHz, MeOD, 303K)

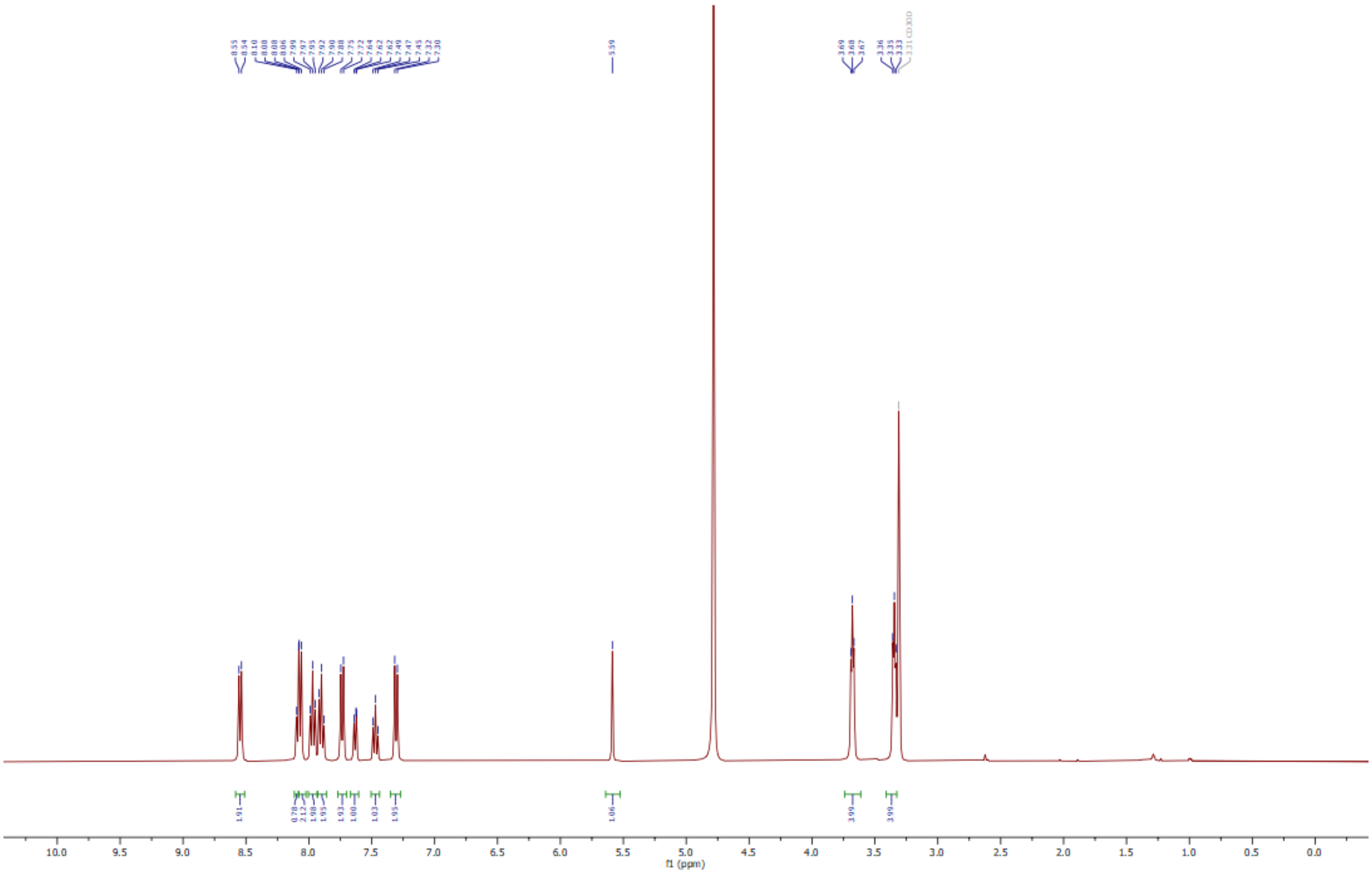

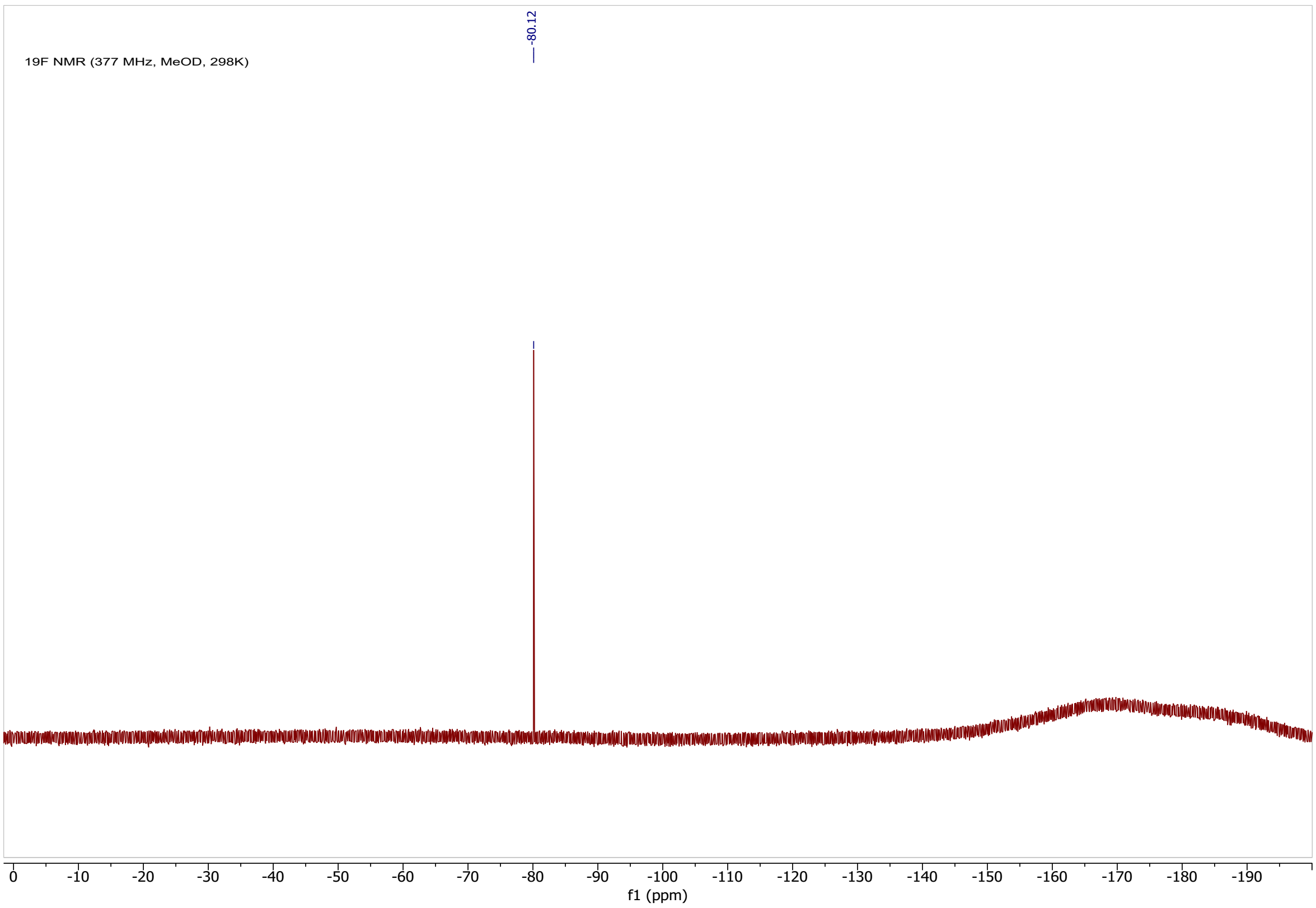

^{13}C NMR (101 MHz, MeOD, 303K)

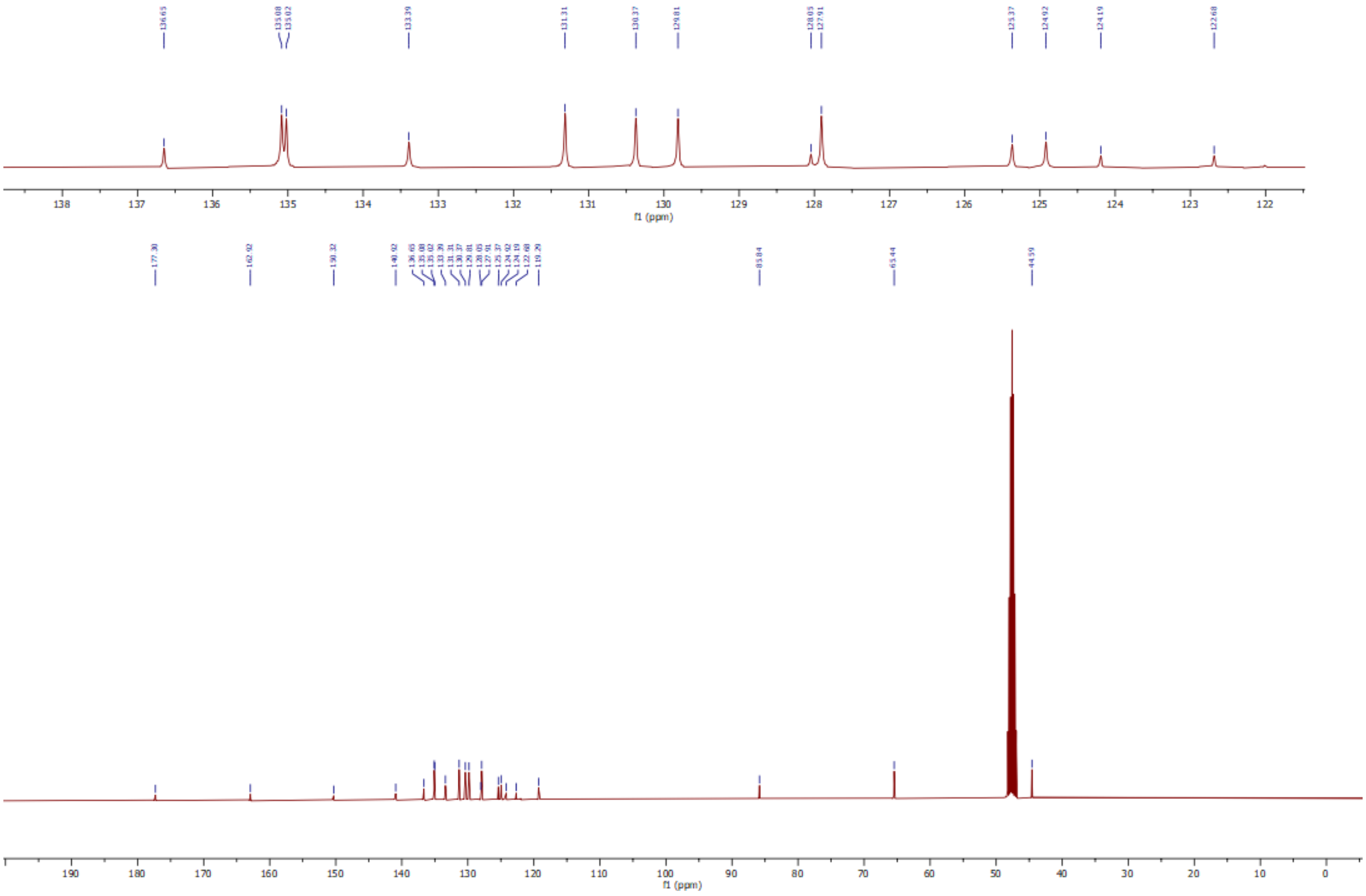

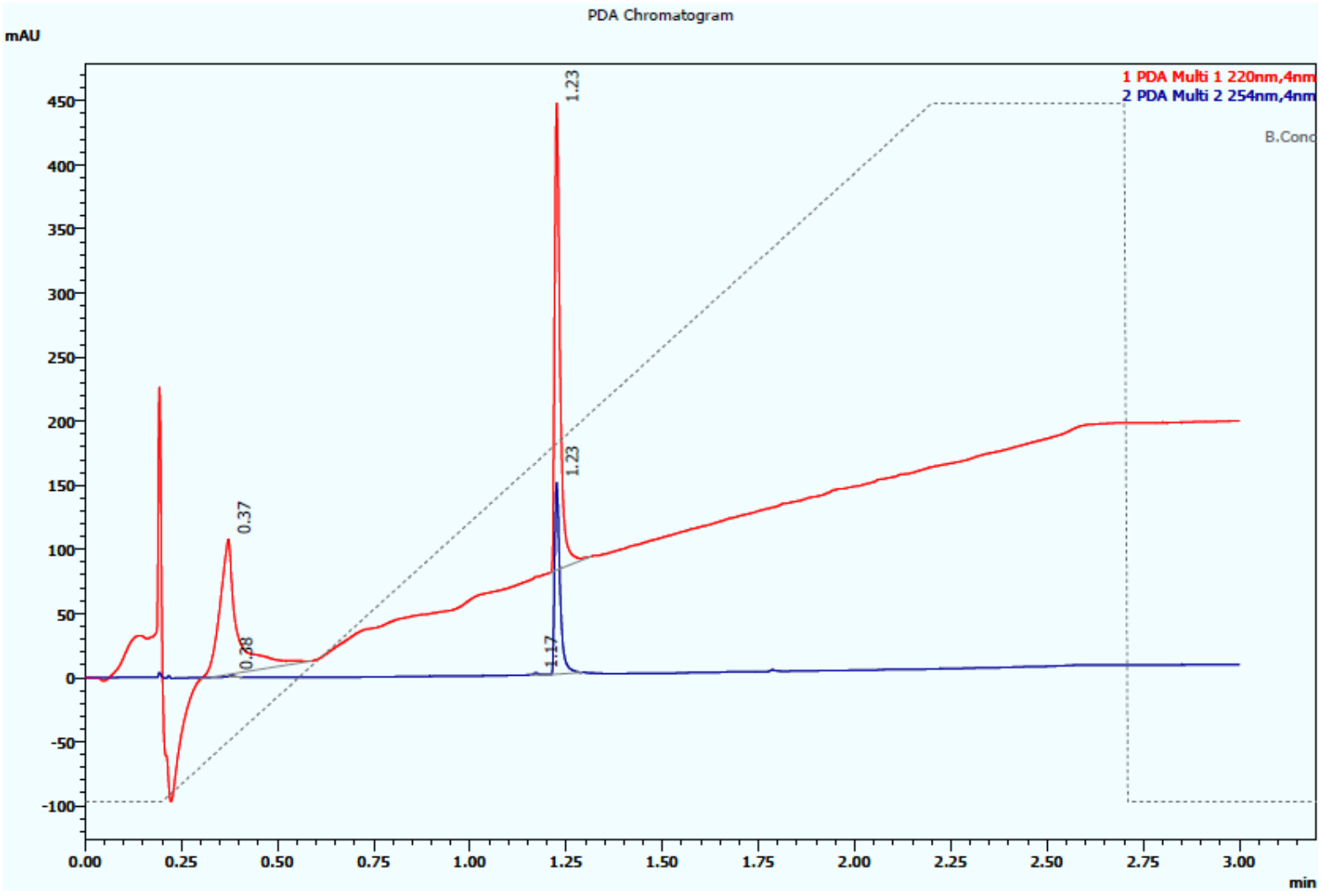

PDA Peak Table

PDA Ch1 220nm

Pk#	R.T.	Area	Height	Area%	UV max	S/N
1	0.37	335513	105439	48.3	206	16.9
3	1.23	359586	363992	51.7	223/301	58.4

PDA Peak Table

PDA Ch2 254nm

Pk#	R.T.	Area	Height	Area%	UV max	S/N
1	0.38	824	1272	0.56	206/345/304/275	1.2
2	1.17	1160	1550	0.79	218	1.4
3	1.23	145636	149259	98.66	223/301	137.2

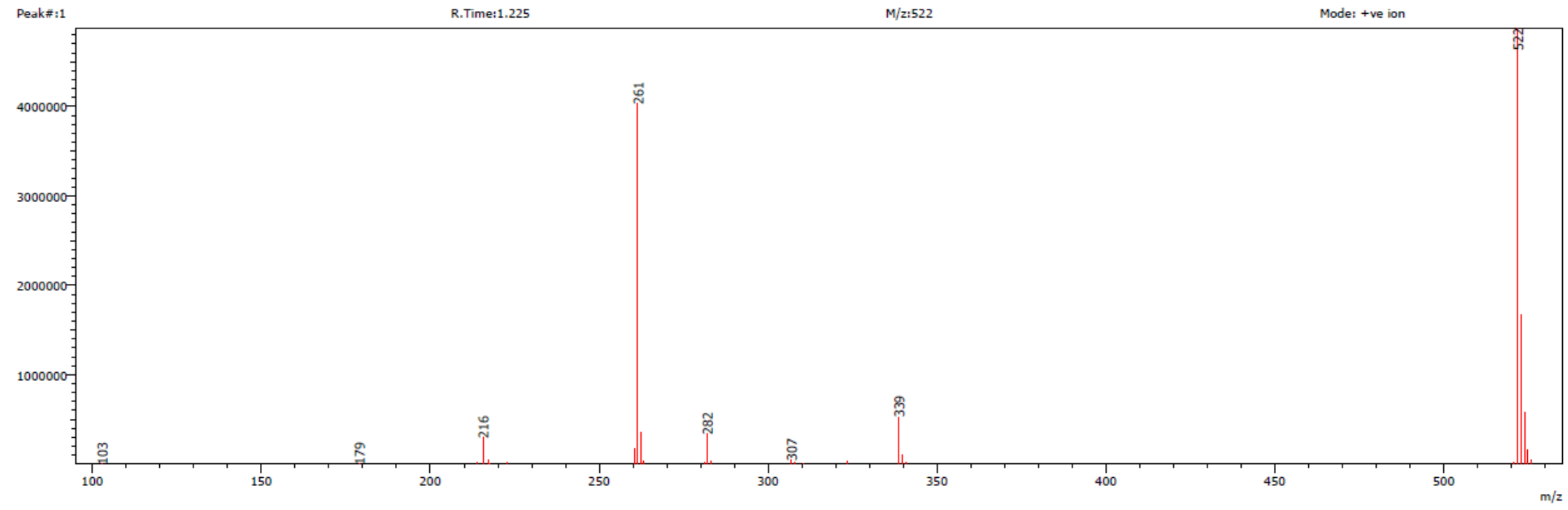

tert-butyl (4-(2-morpholino-4-oxo-4H-chromen-8-yl)benzyl)carbamate (9a)

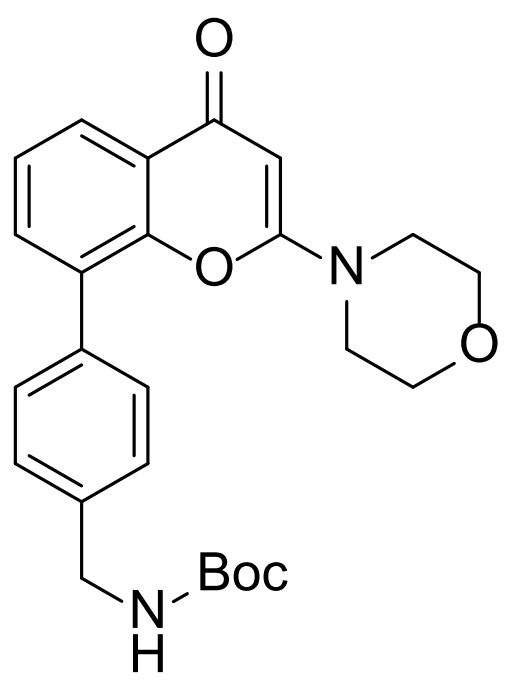

^{1}H NMR (400 MHz, $CDCl_3$, 303K)

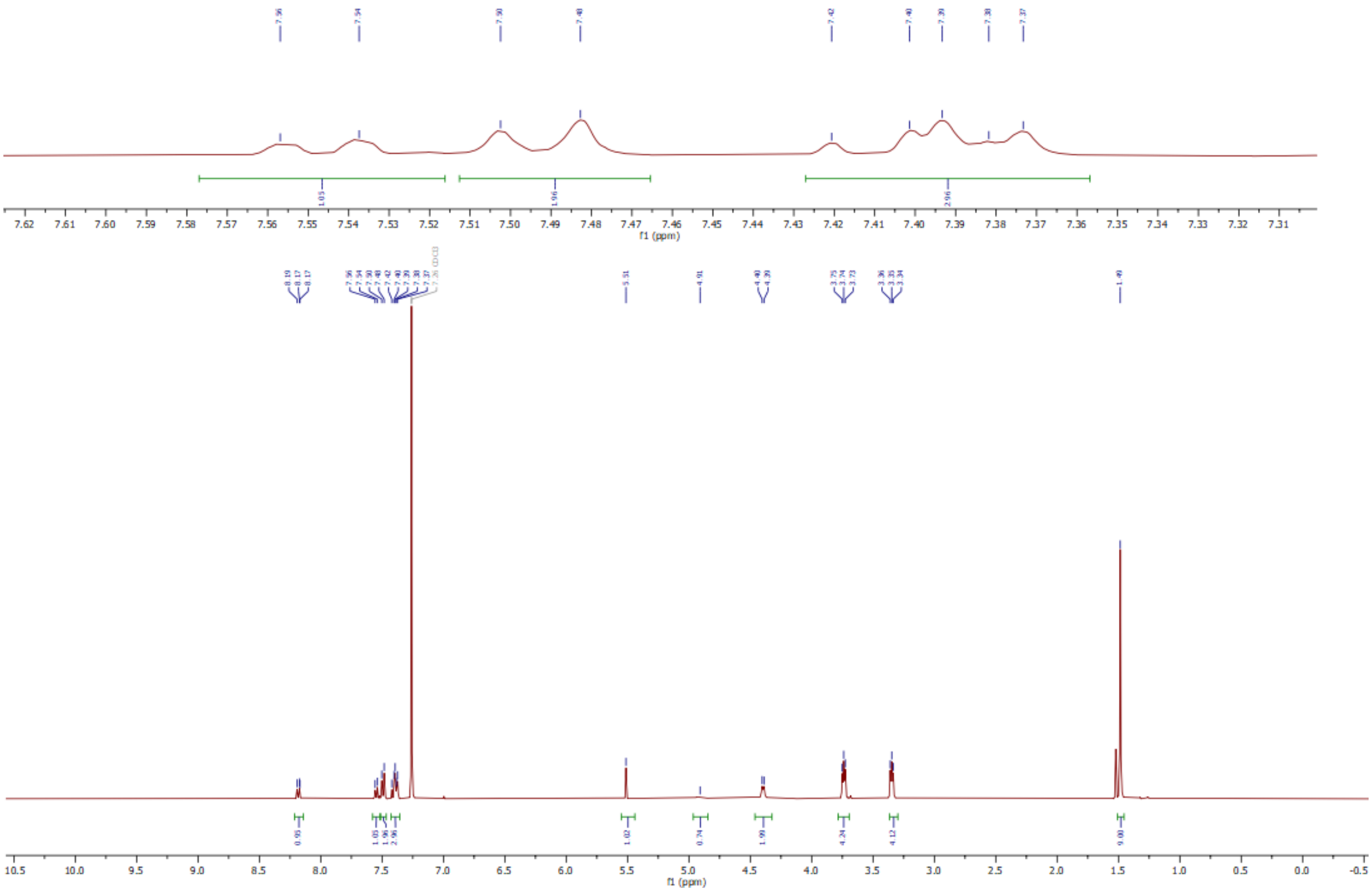

Chromatogram

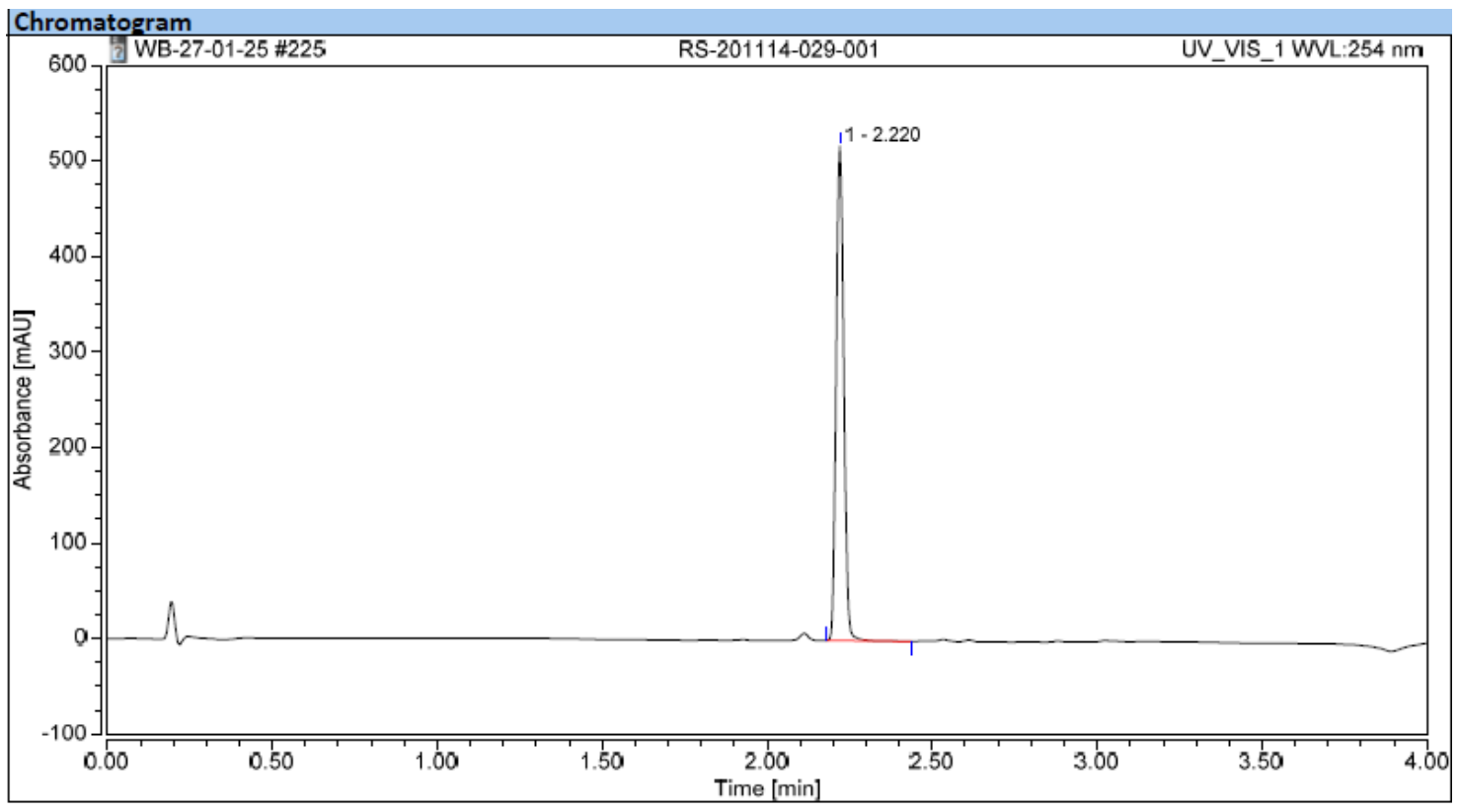

Integration Results

No.	Peak Name	Retention Time	Area	Height	Relative Area	Relative Height	Amount
		min	mAU*min	mAU	%	%	n.a.
1		2.220	13.900	517.288	100.00	100.00	n.a.
**Total:**			**13.900**	**517.288**	**100.00**	**100.00**	

Spectrum RT 2:14 (1 scans)
null;
ESI + Settings for tune mix using source type ESI Positive. Max: 1.8E8

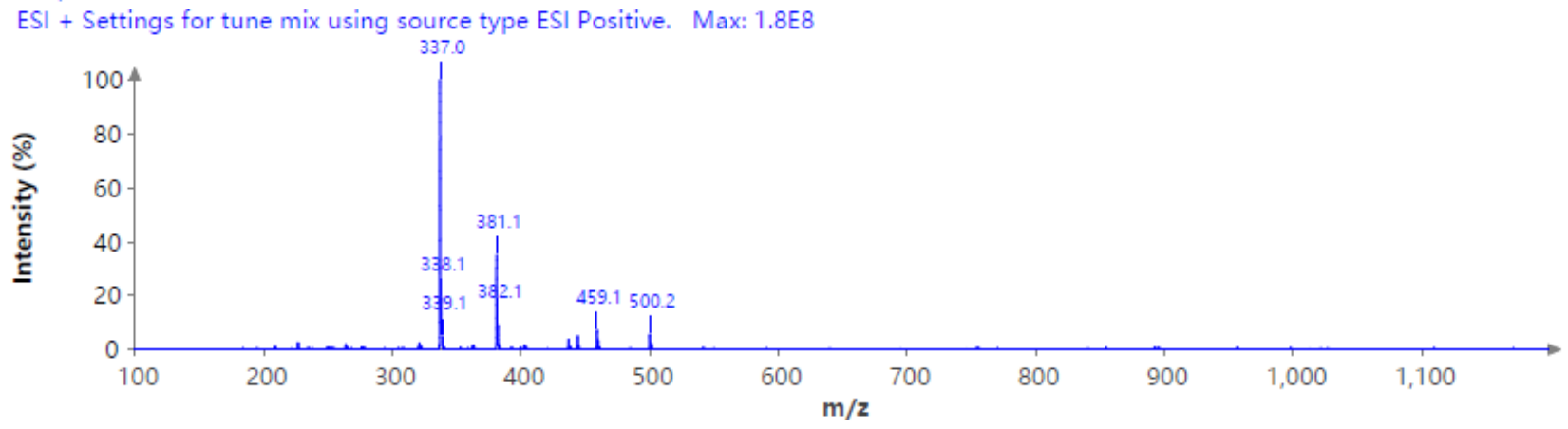

8-(4-(aminomethyl)phenyl)-2-morpholino-4H-chromen-4-one dihydrochloride (CZC15098, 9b)

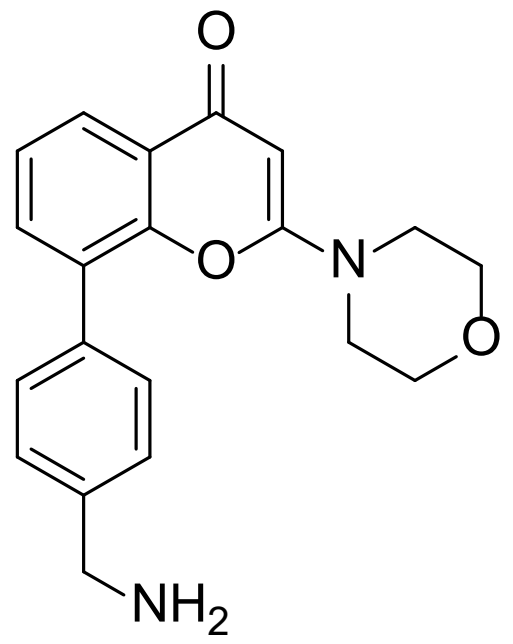

^{1}H NMR (400 MHz, DMSO, 303K)

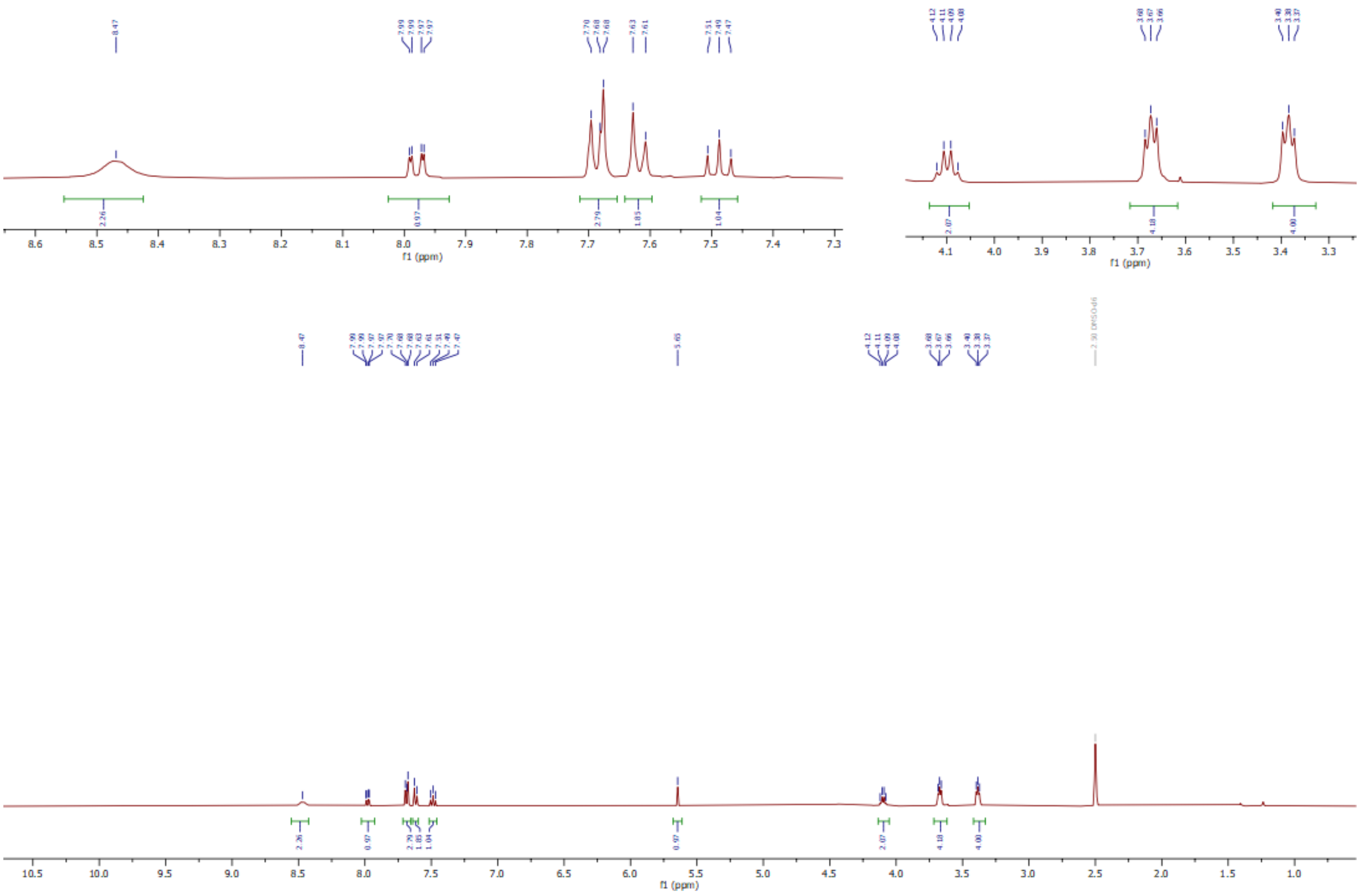

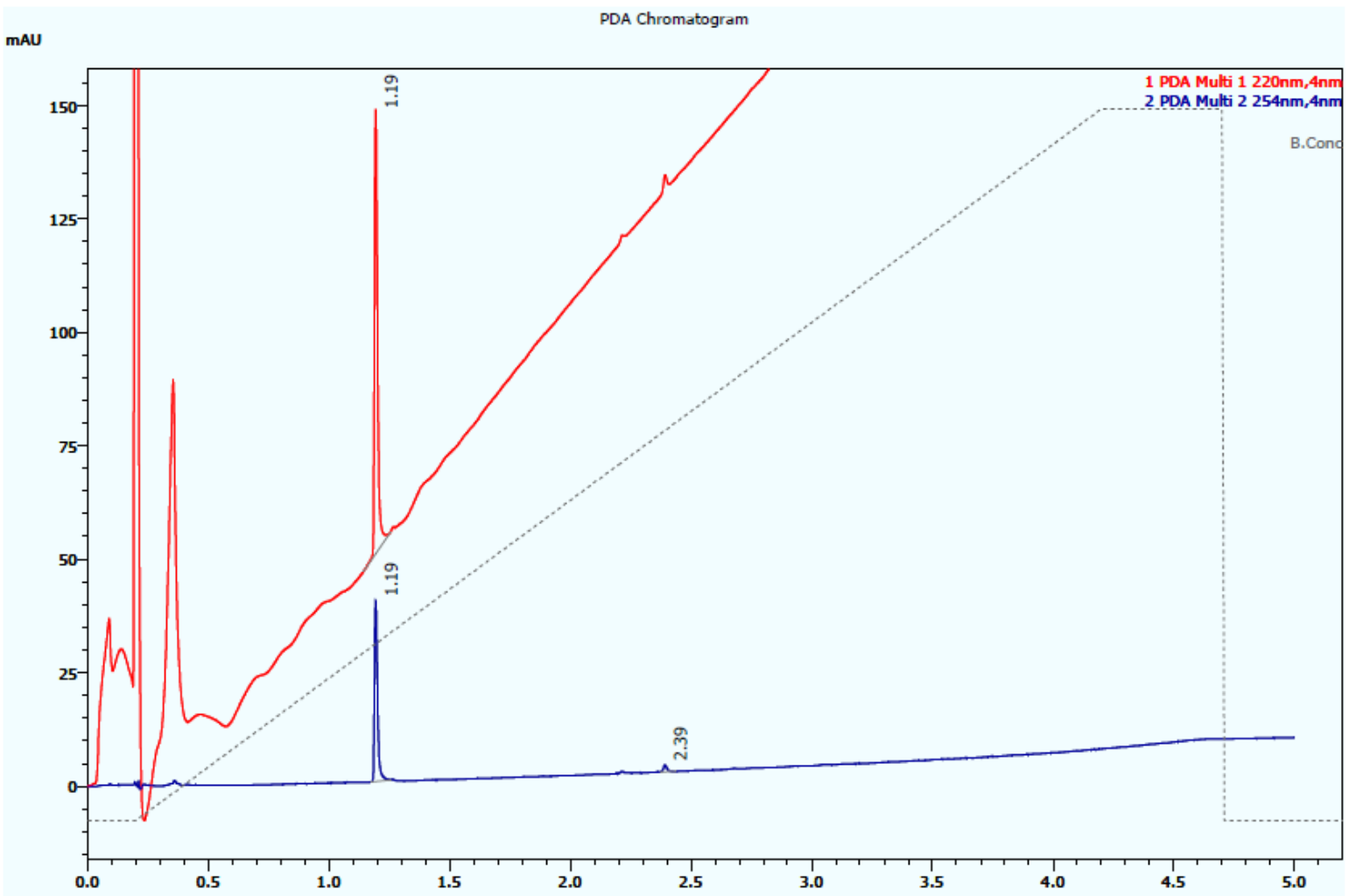

PDA Peak Table

PDA Ch1 220nm

Pk#	R.T.	Area	Height	Area%	UV max	S/N
1	1.19	85537	97972	100.0	204/303	1.1

PDA Peak Table

PDA Ch2 254nm

Pk#	R.T.	Area	Height	Area%	UV max	S/N
1	1.19	34449	39702	95.69	204/303	88.2
2	2.39	1551	1487	4.31	217/304	3.3

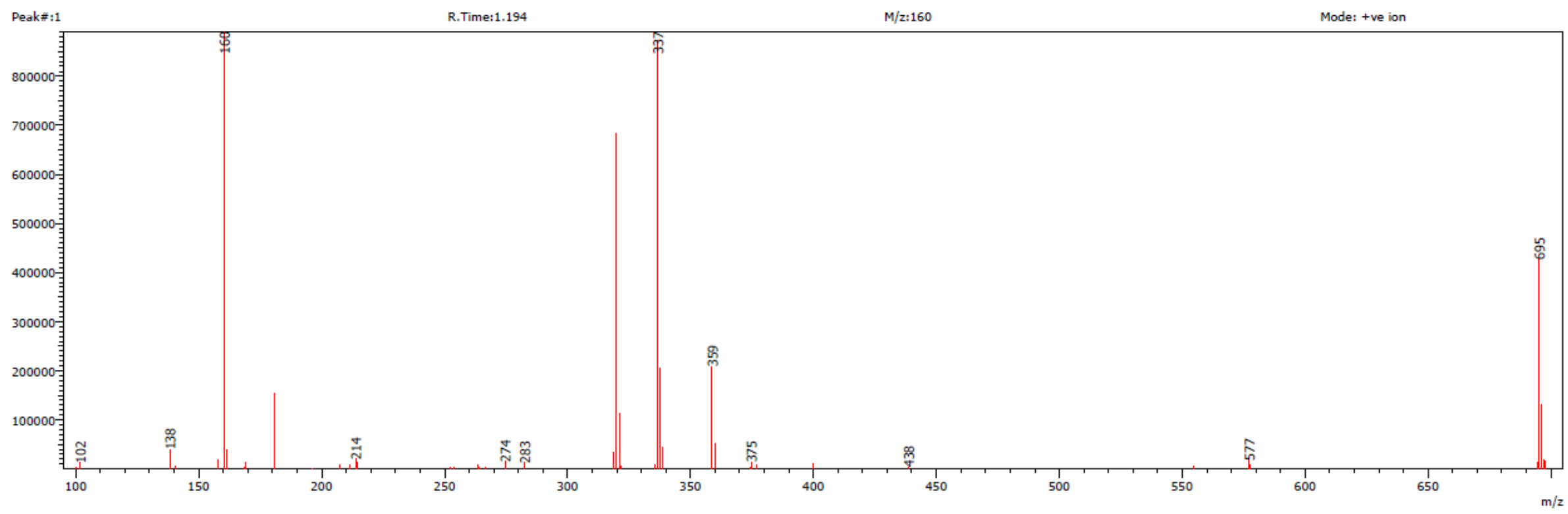

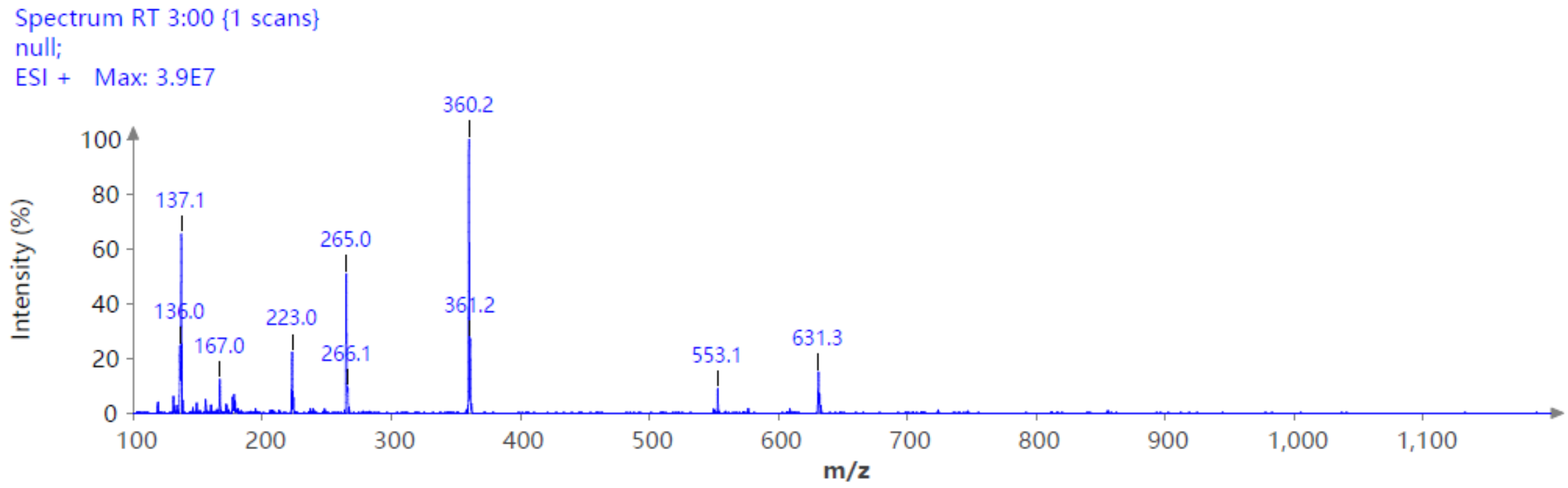

5-(4-methoxy-3-(6-(((1r,4r)-4-((2-methylpropyl)sulfonamido)cyclohexyl)amino)-1*H*-pyrazolo[3,4-d]pyrimidin-3-yl)phenyl)-5H-thianthren-5-ium formate (S4)

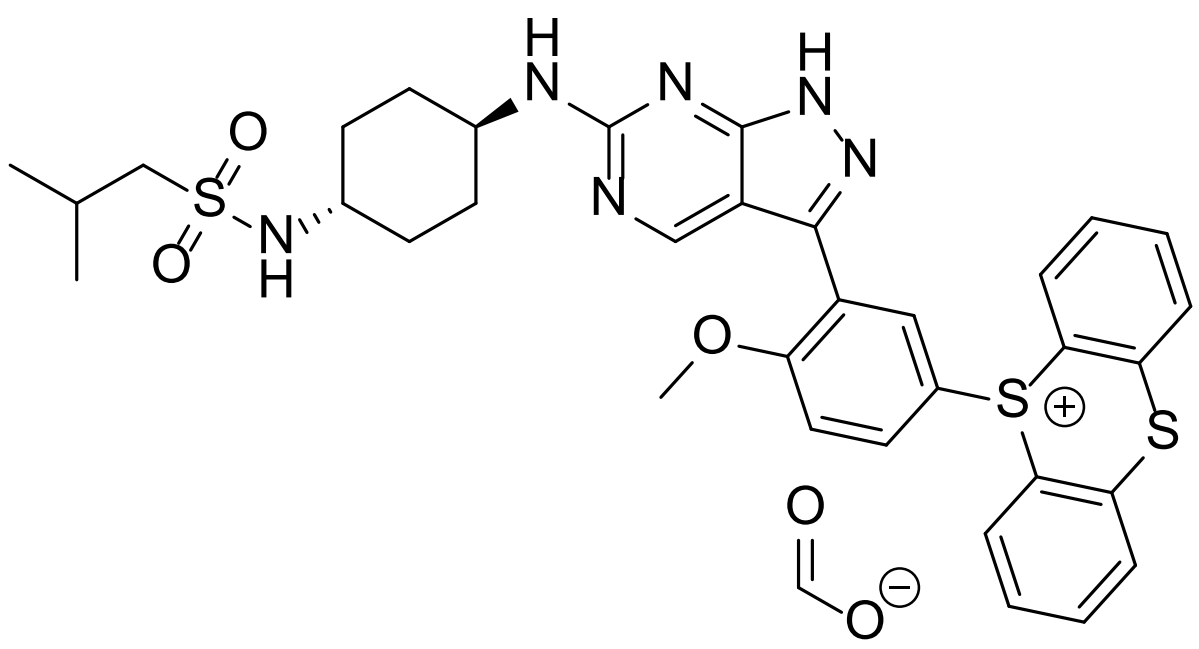

^{1}H NMR (500 MHz, MeOD, 303K)

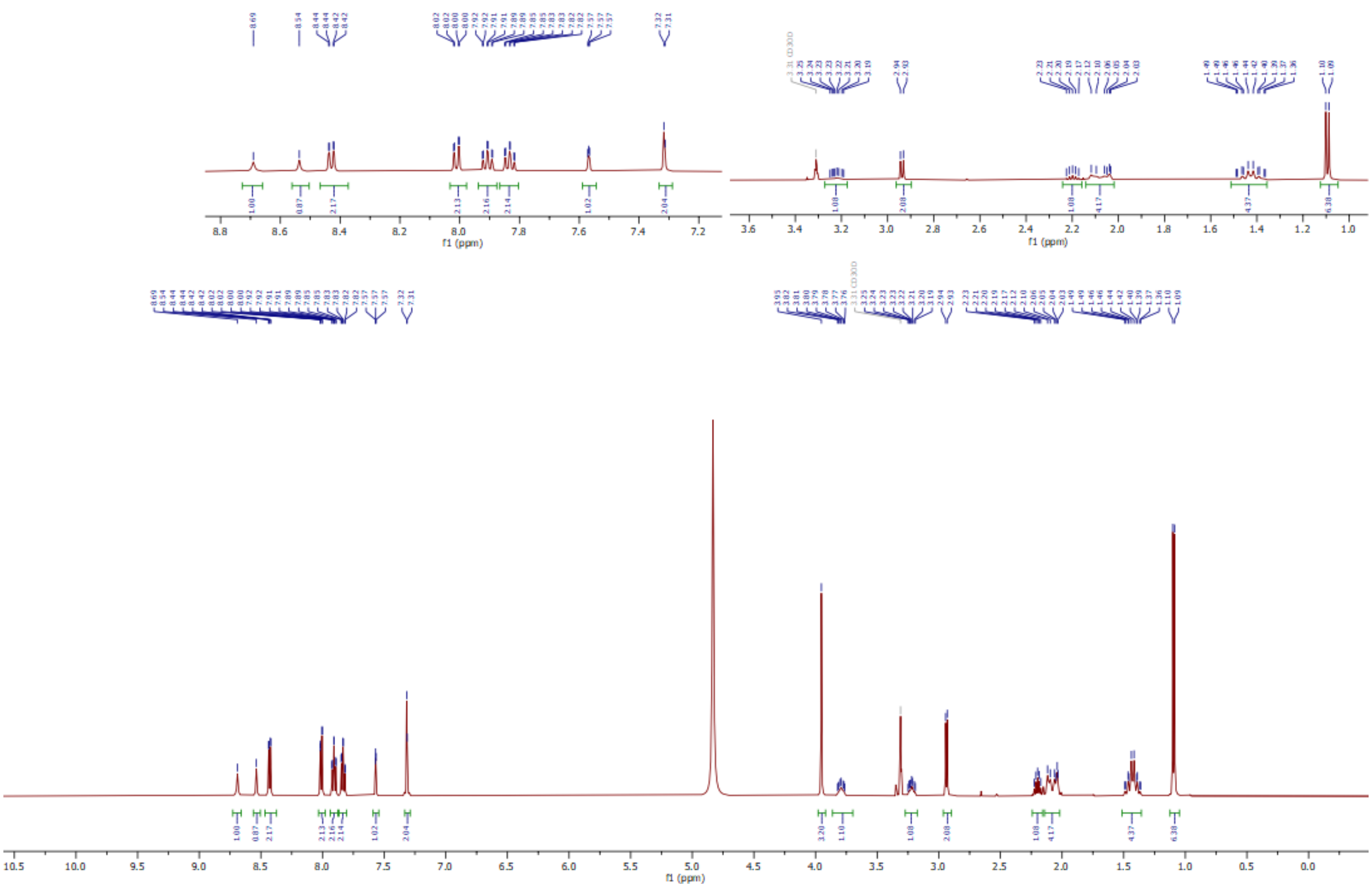

^{13}C NMR (126 MHz, MeOD, 303K)

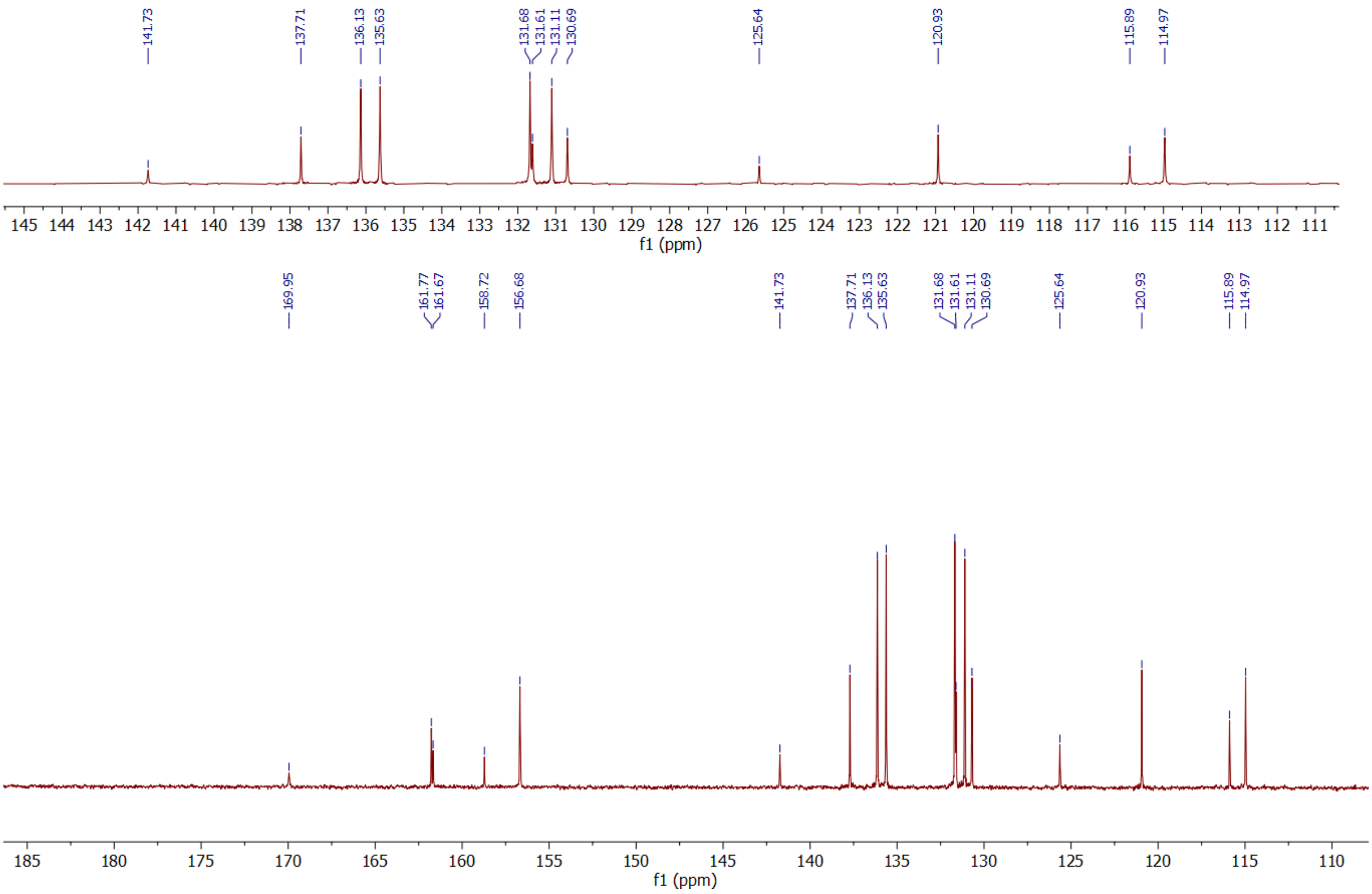

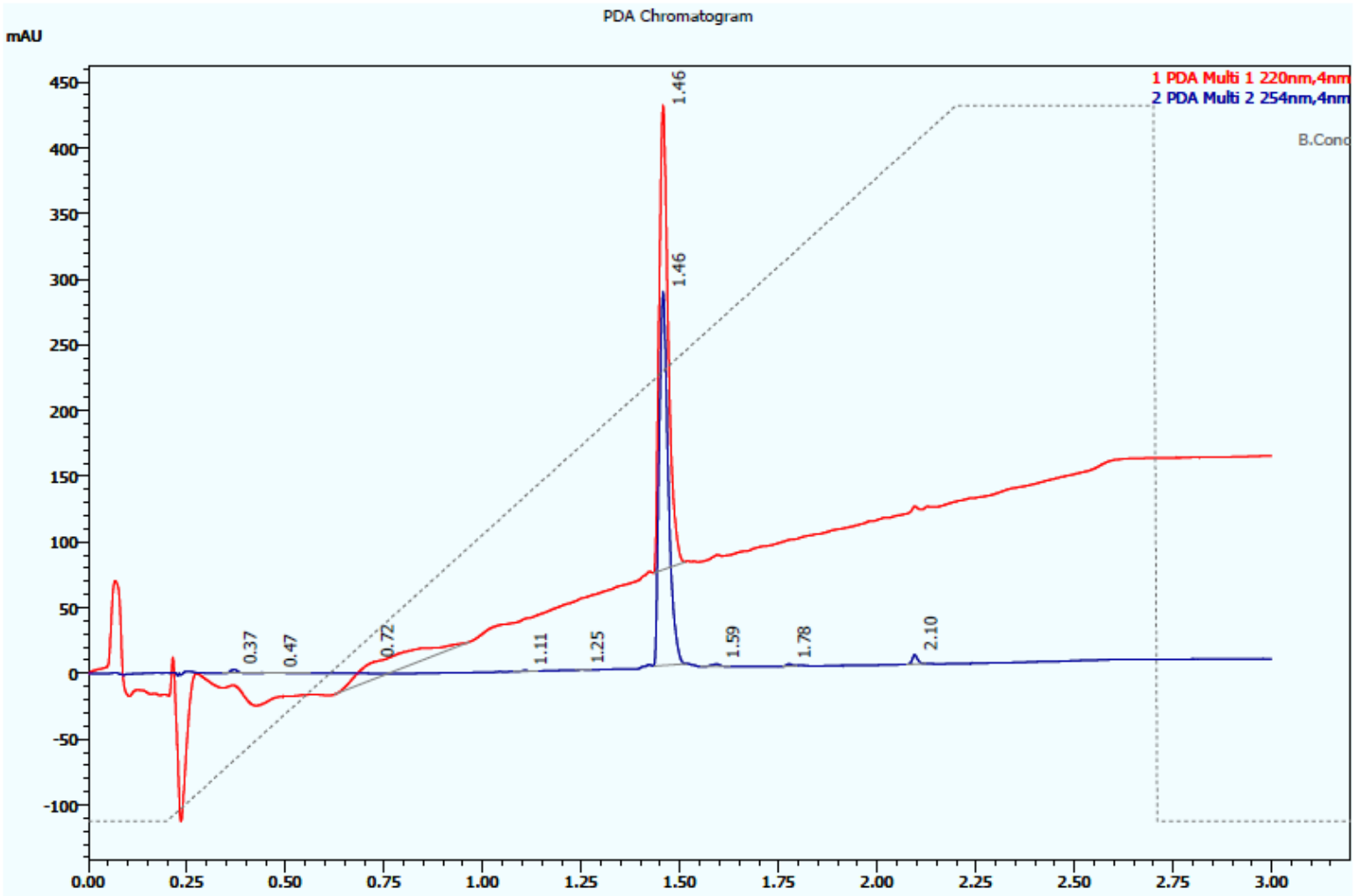

PDA Peak Table

PDA Ch1 220nm

Pk#	R.T.	Area	Height	Area%	UV max	S/N
3	0.72	155099	13113	21.6	220	0.2
6	1.46	562895	353167	78.4	239/306	5.9

PDA Peak Table

PDA Ch2 254nm

Pk#	R.T.	Area	Height	Area%	UV max	S/N
1	0.37	3647	2924	0.77	265	3.4
2	0.47	1264	391	0.27	280	0.5
4	1.11	768	701	0.16	221	0.8
5	1.25	396	409	0.08	222/287/376	0.5
6	1.46	454862	284016	96.38	239/306	334.8
7	1.59	2953	2012	0.63	223	2.4
8	1.78	1444	1514	0.31	223/345	1.8
9	2.10	6622	7131	1.40	223	8.4

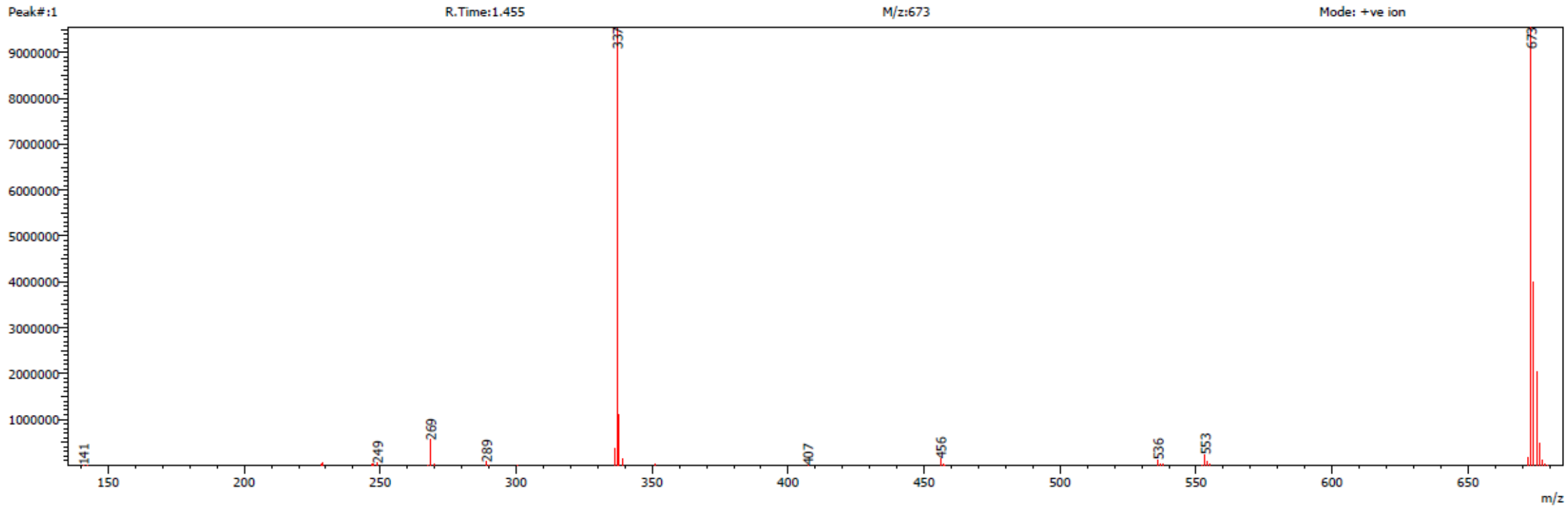

(*R*)-5-(1-(2-(4-chlorophenoxy)acetyl)-2-(methylcarbamoyl)indolin-5-yl)-5H-thianthren-5-ium tetrafluoroborate (S1)

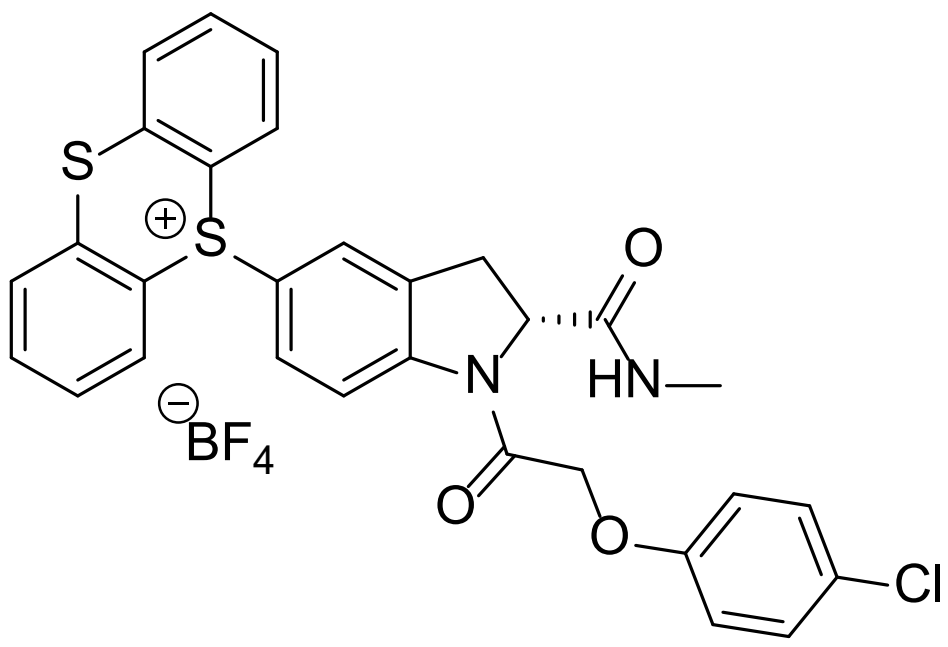

^{1}H NMR (500 MHz, $CDCl_3$, 298K)

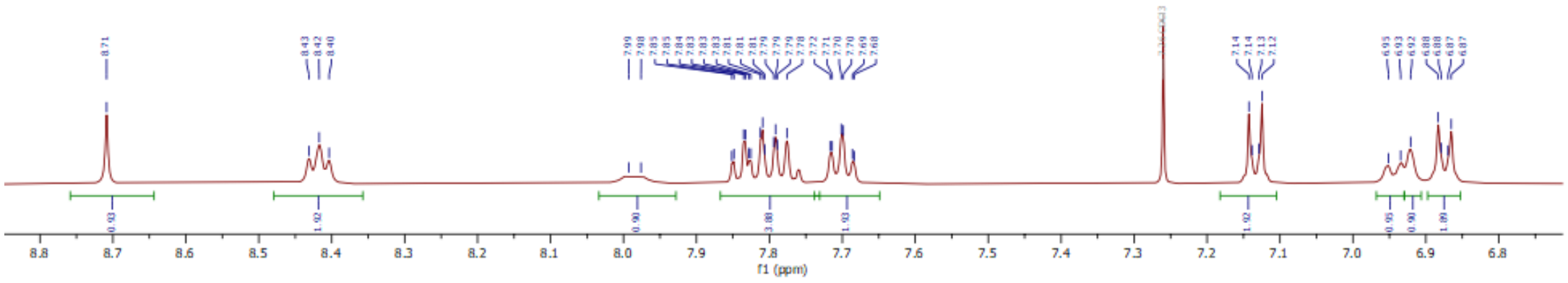

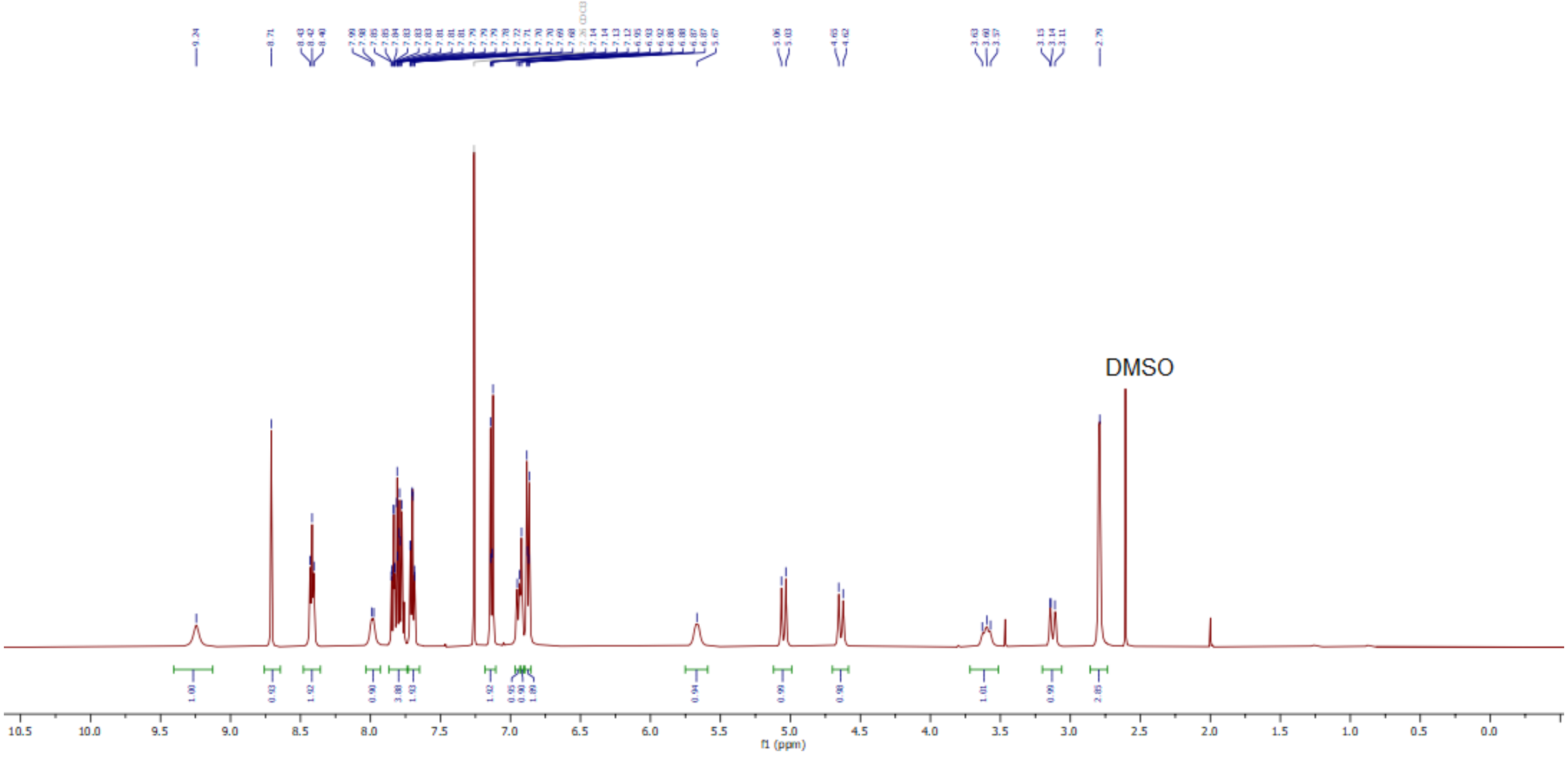

^{13}C NMR (126 MHz, $CDCl_3$, 298K)

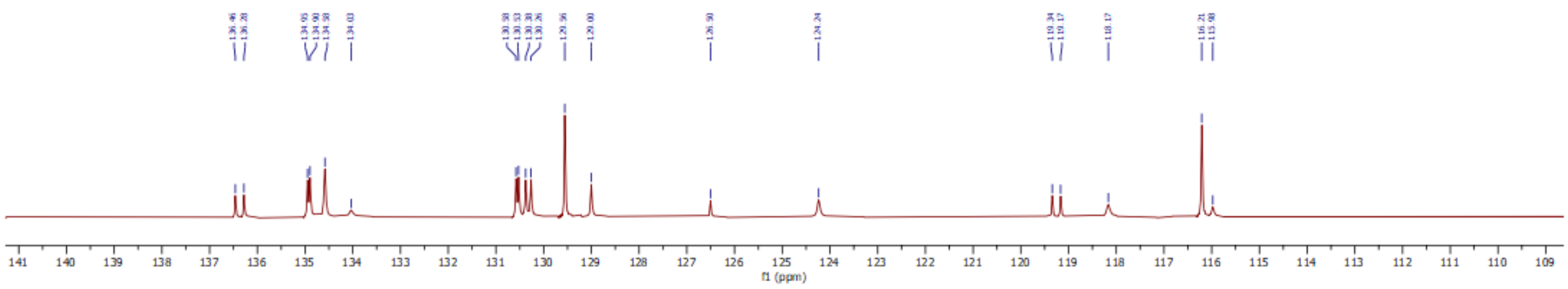

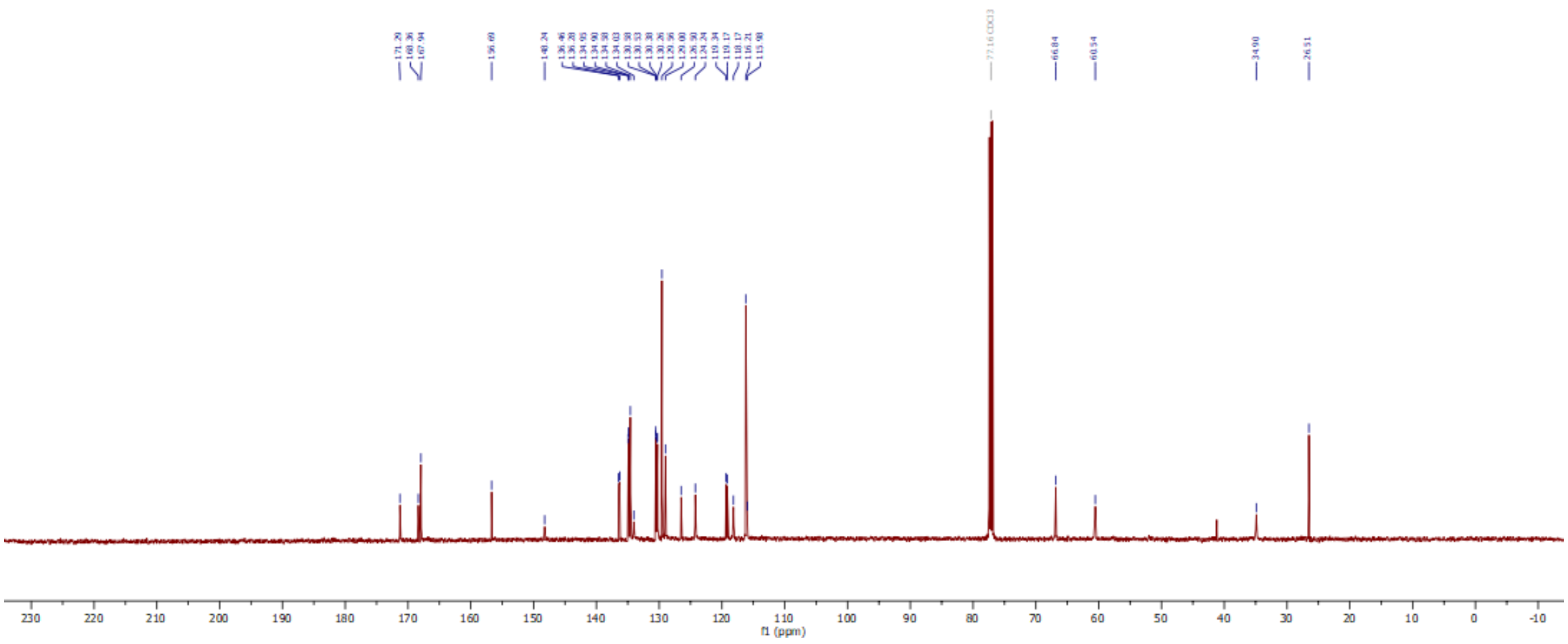

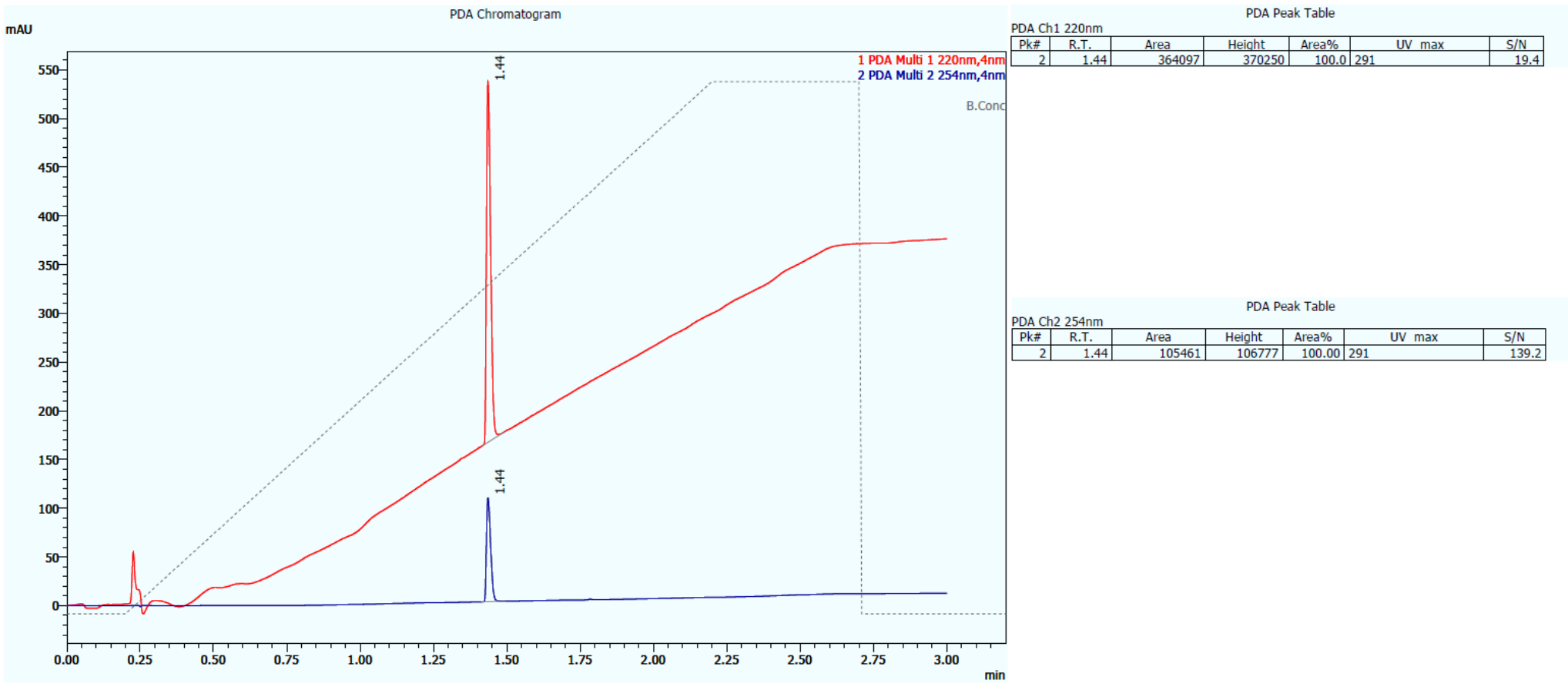

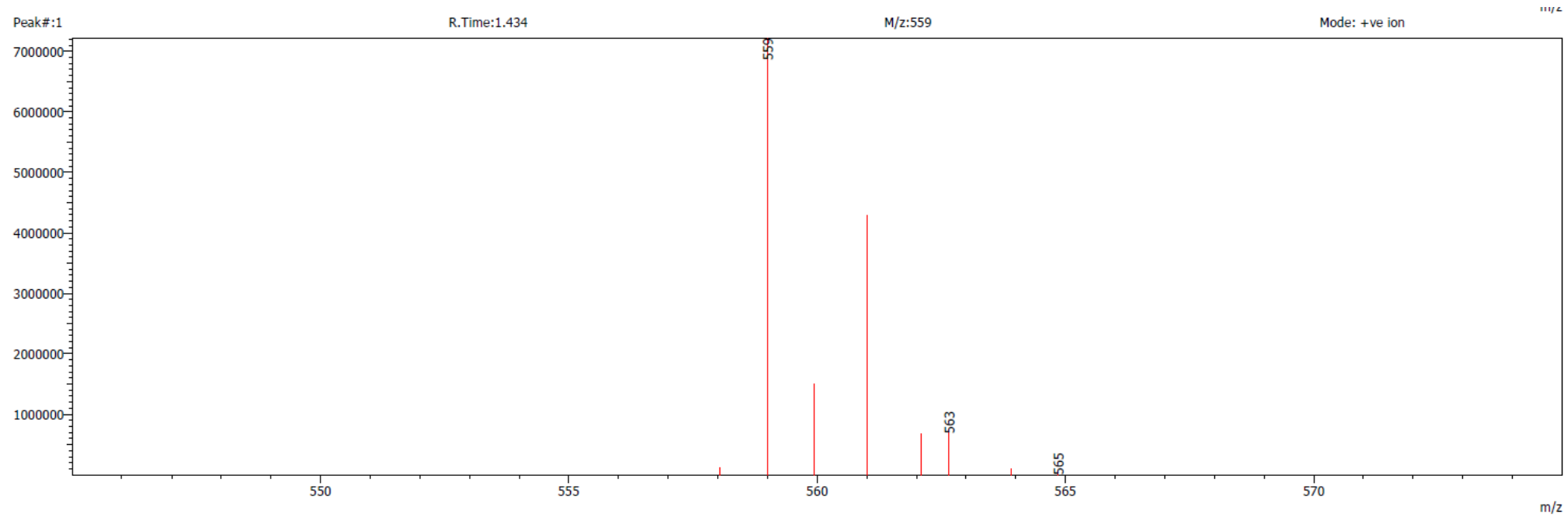

tert-butyl (*R*)-(2-(2-(2-((3-(1-(2-(4-chlorophenoxy)acetyl)-2-(methylcarbamoyl)indolin-5-yl)prop-2-yn-1-yl)oxy)ethoxy)ethoxy)ethyl)carbamate (26a)

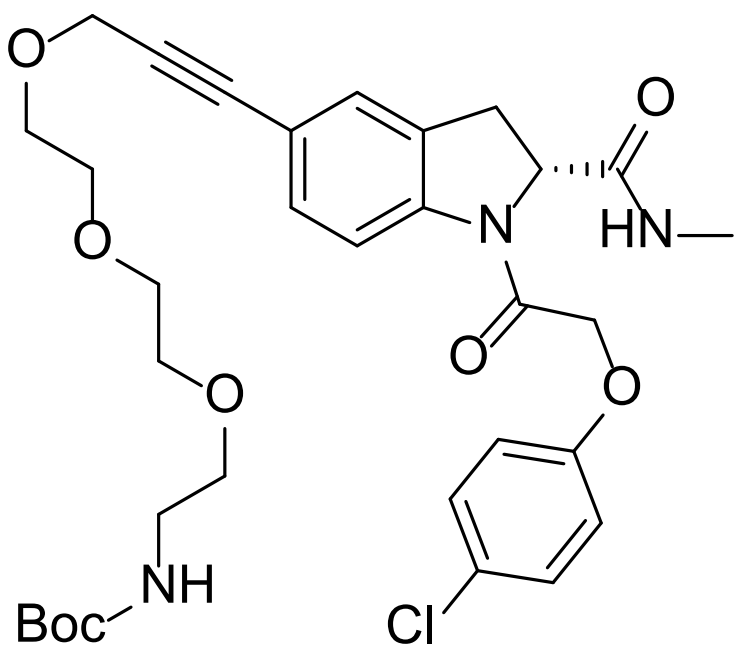

^{1}H NMR (500 MHz, MeOD, 298K)

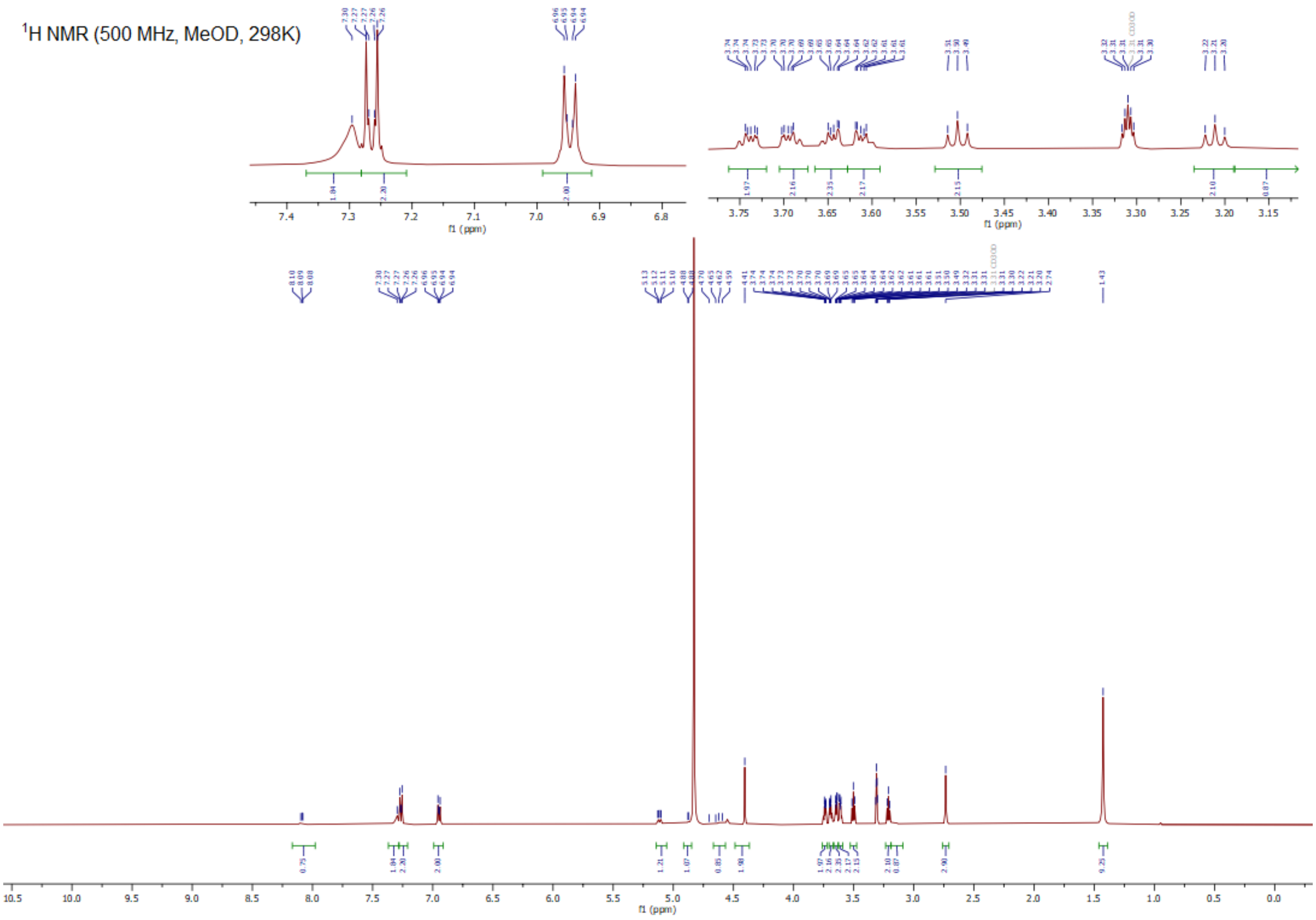

^{13}C NMR (126 MHz, MeOD, 298K)

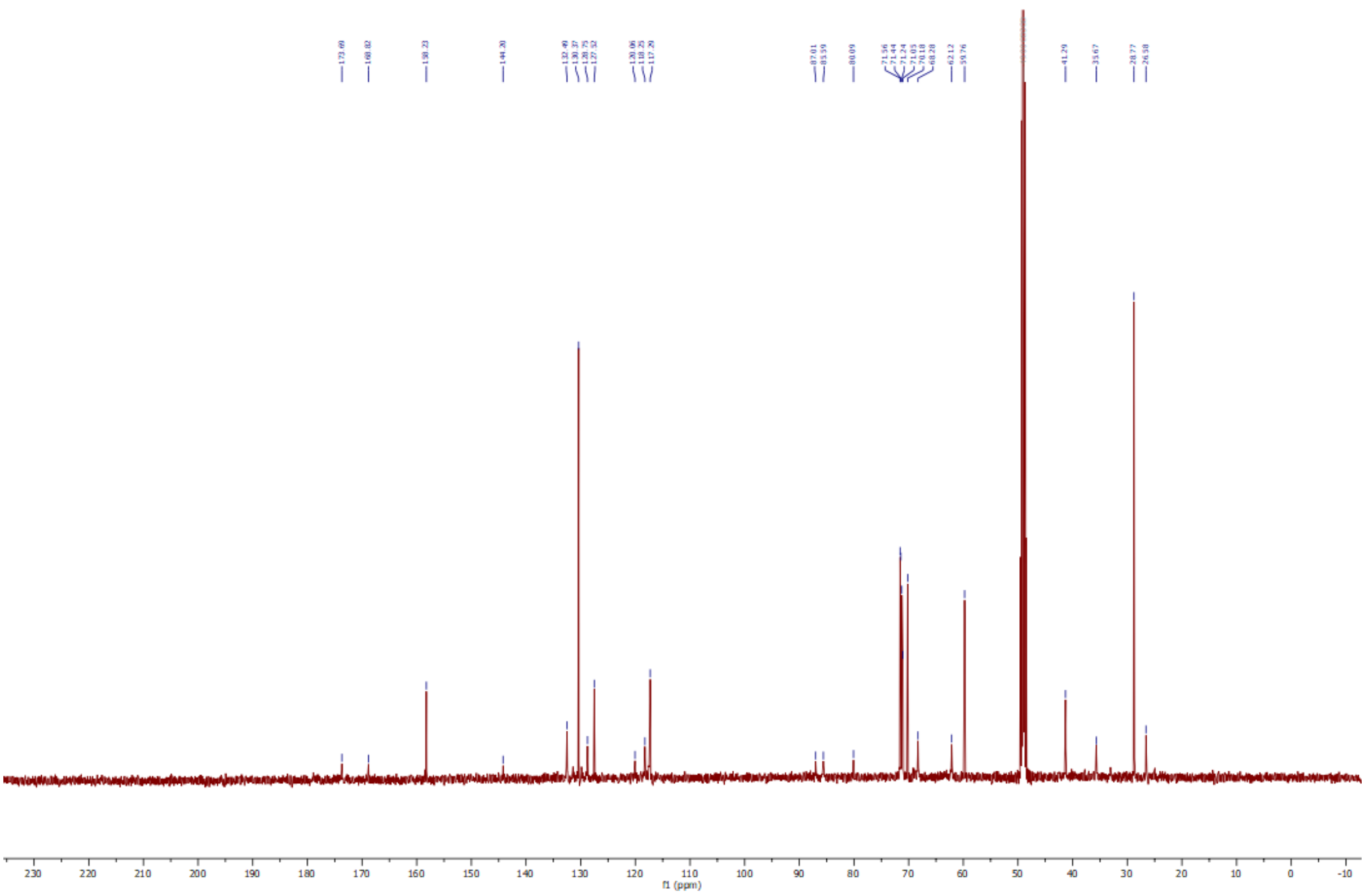

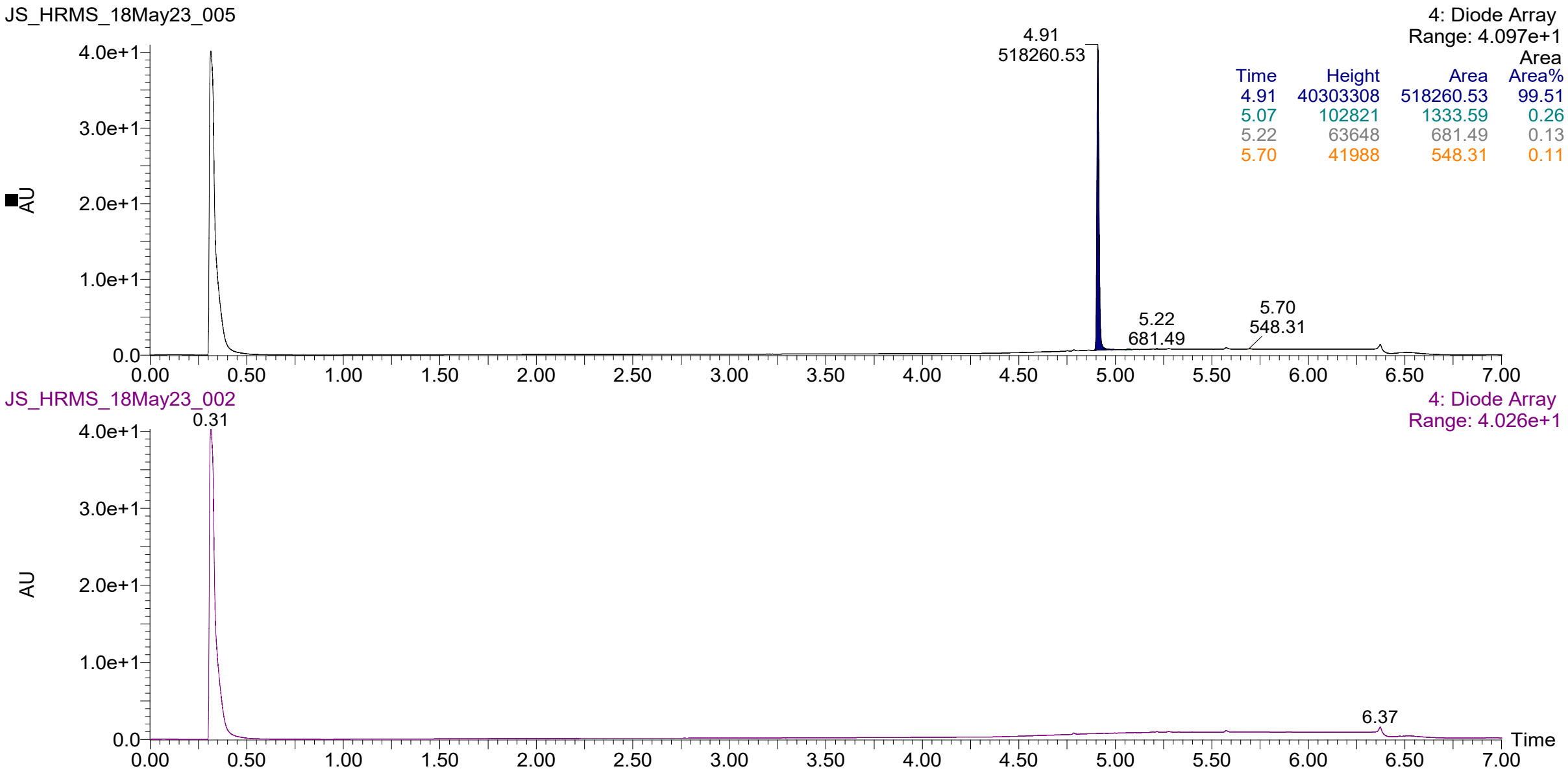

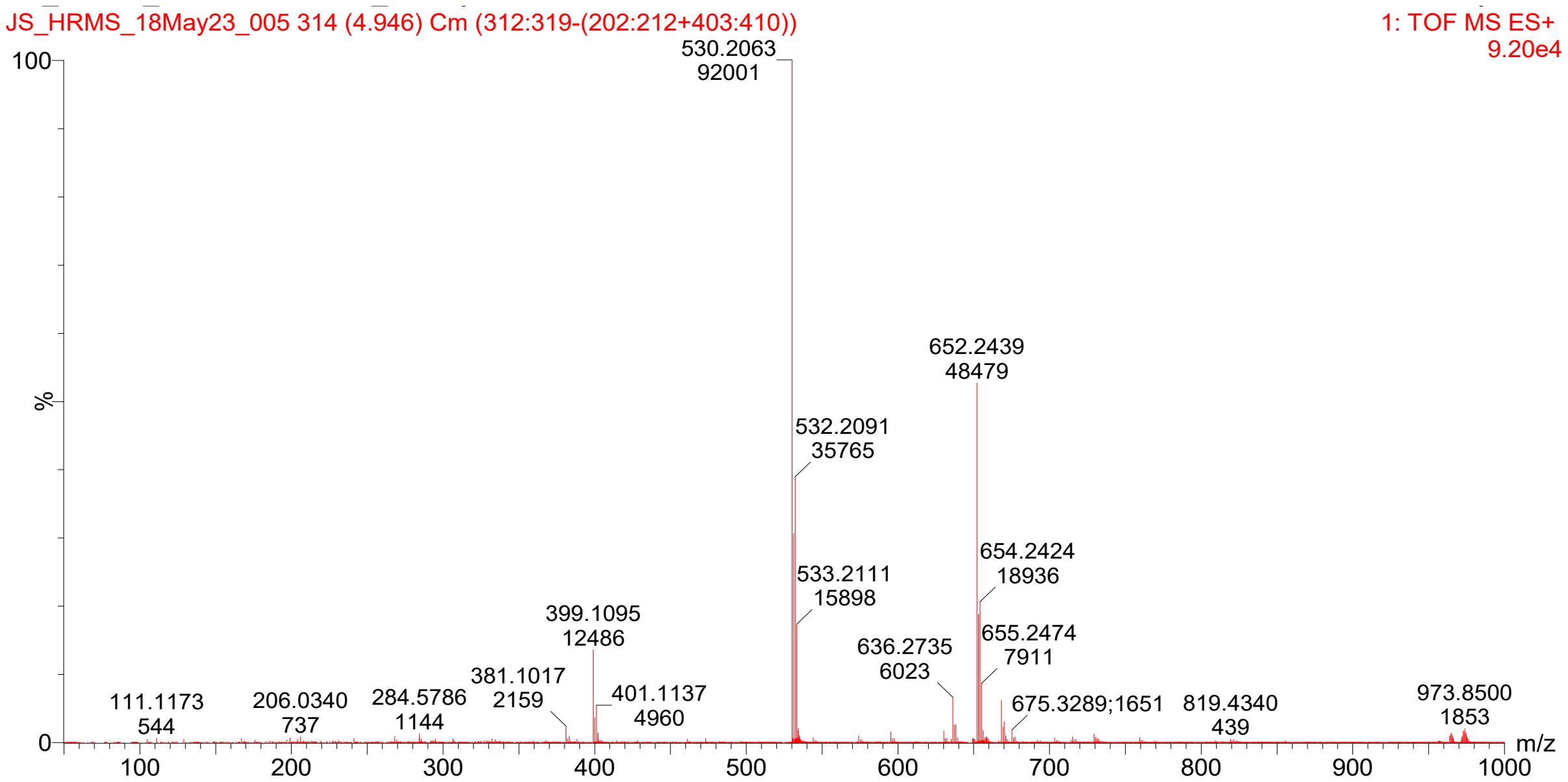

tert-butyl (*R*)-(2-(2-(2-((1-(2-(4-chlorophenoxy)acetyl)-2-(methylcarbamoyl)indolin-5-yl)amino)ethoxy)ethoxy)ethyl)carbamate (26b)

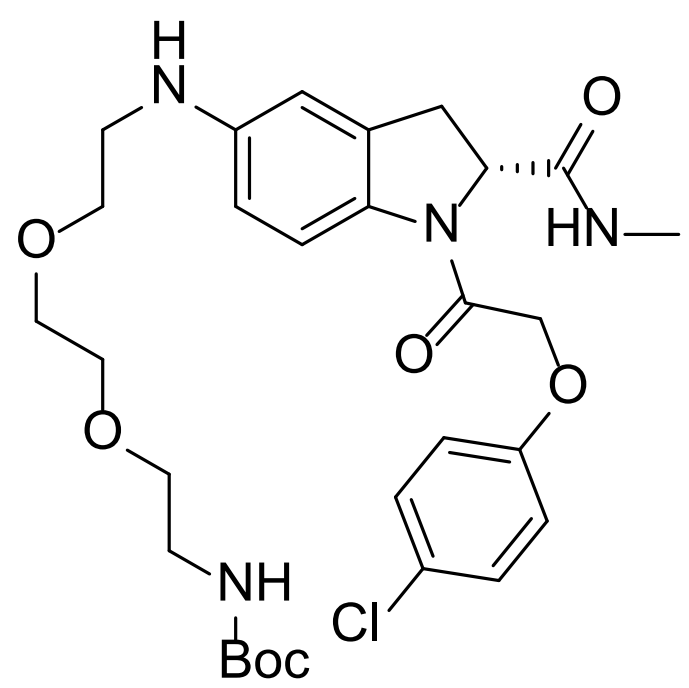

^{1}H NMR (500 MHz, DMSO-d6, 298K)

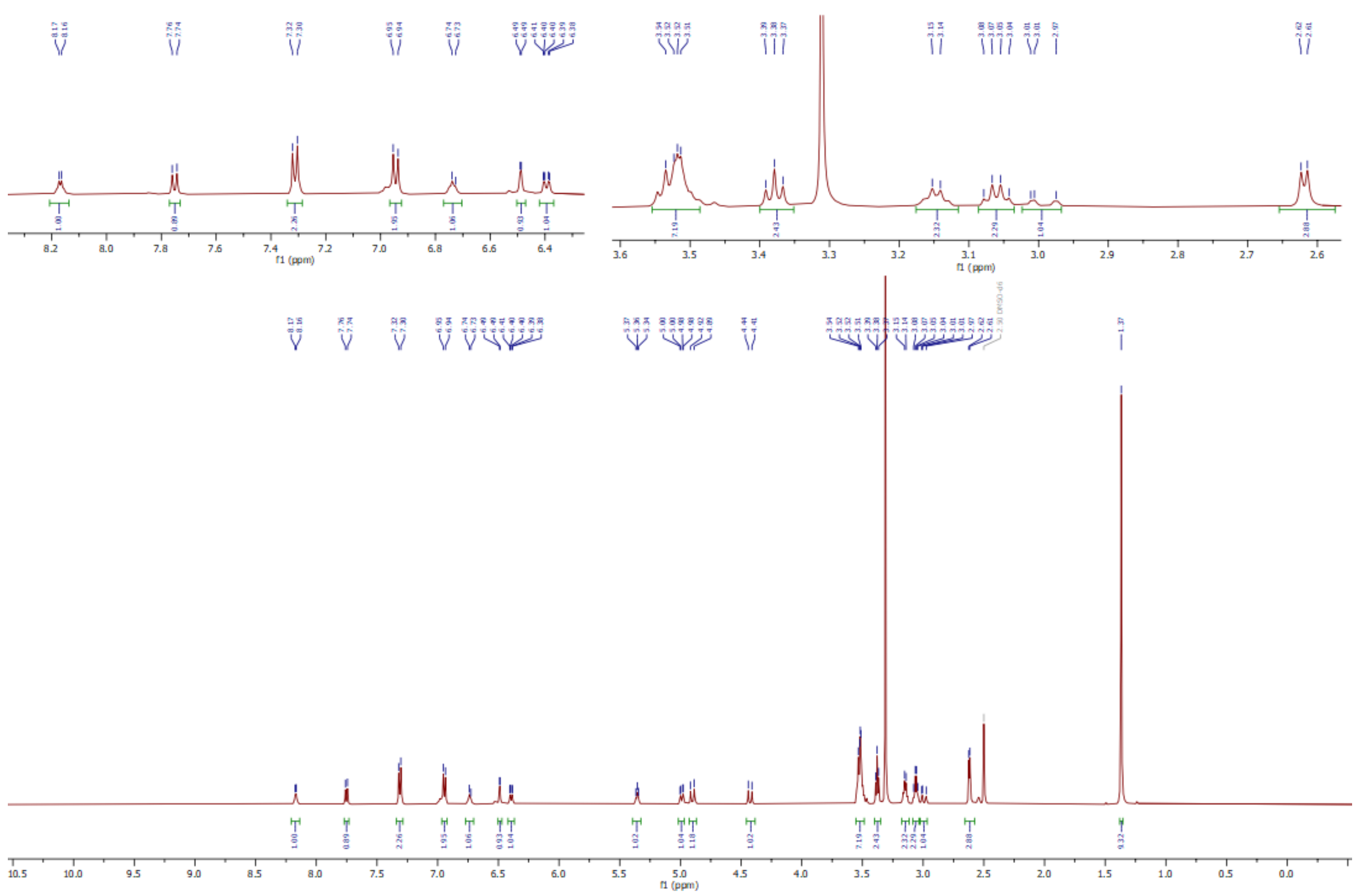

^{13}C NMR (126 MHz, DMSO-d6, 298K)

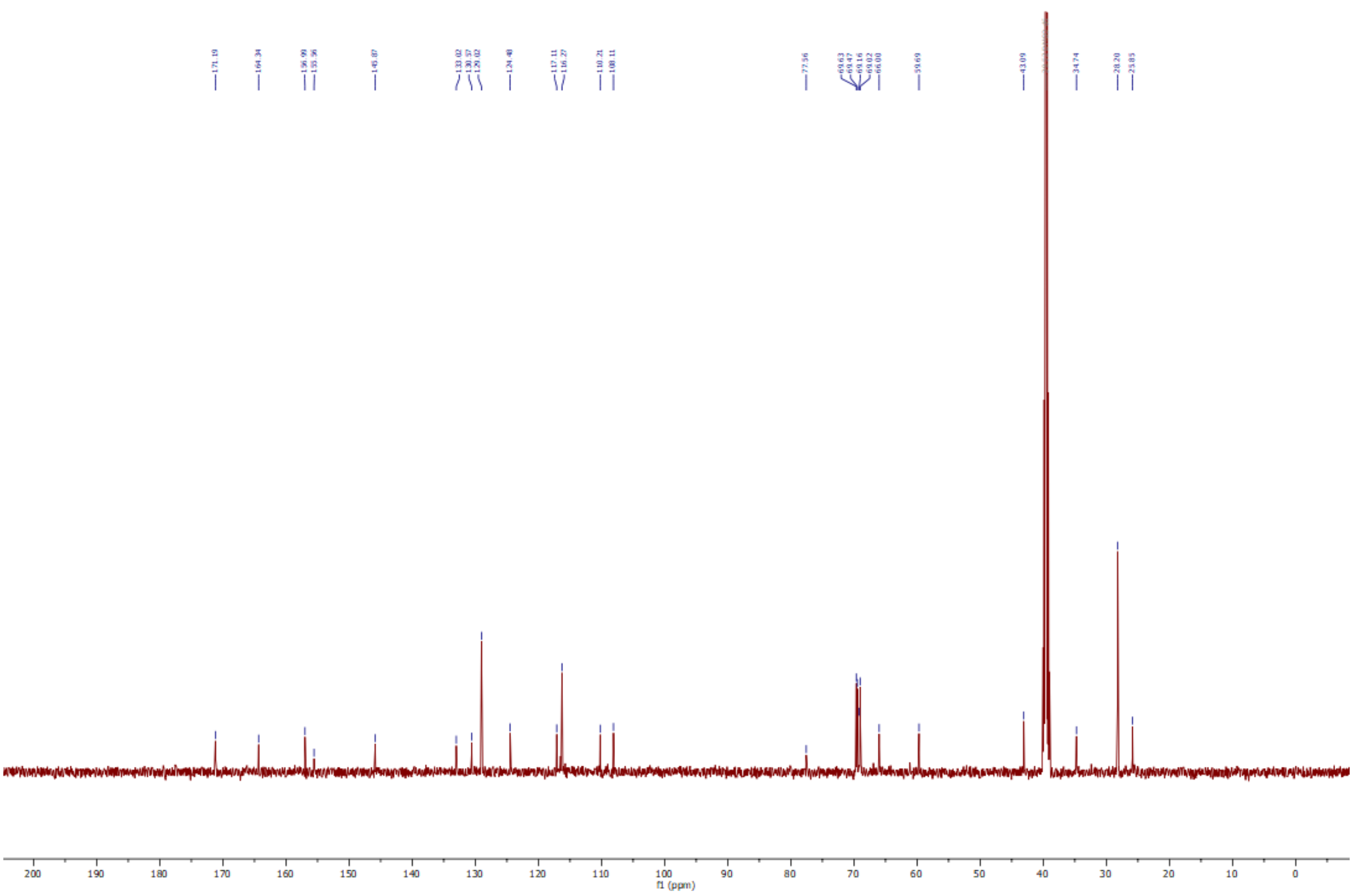

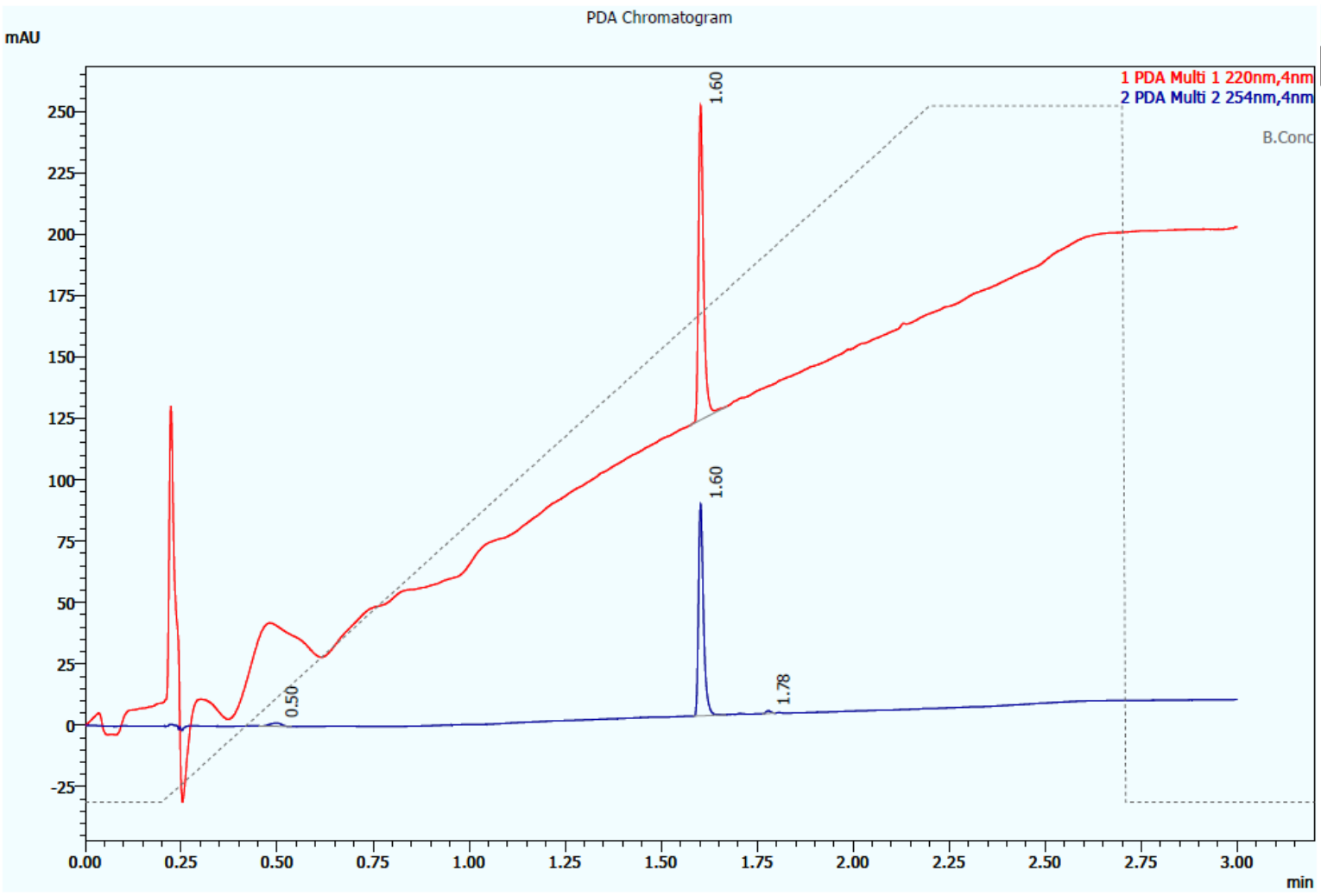

PDA Peak Table

PDA Ch1 220nm

Pk#	R.T.	Area	Height	Area%	UV_max	S/N
2	1.60	116463	127925	100.0	278/265	3.3

PDA Peak Table

PDA Ch2 254nm

Pk#	R.T.	Area	Height	Area%	UV_max	S/N
1	0.50	2668	1310	3.27	206/265/345	2.5
2	1.60	77971	86179	95.65	278/265	162.0
3	1.78	875	1057	1.07	220	2.0

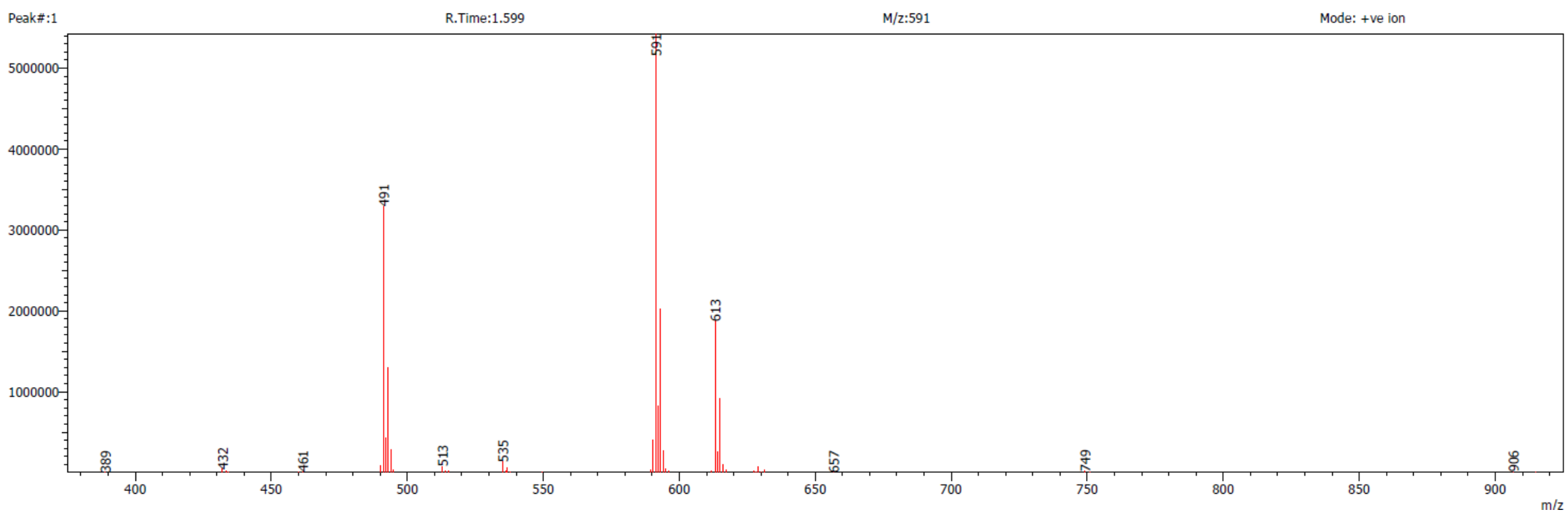

4-(4-((5-amino-4-((3-methoxy-4-(5H-thianthren-5-ium-5-yl)phenyl)carbamoyl)-1H-1,2,3-triazol-1-yl)methyl)benzyl)morpholin-4-ium diformate (S2)

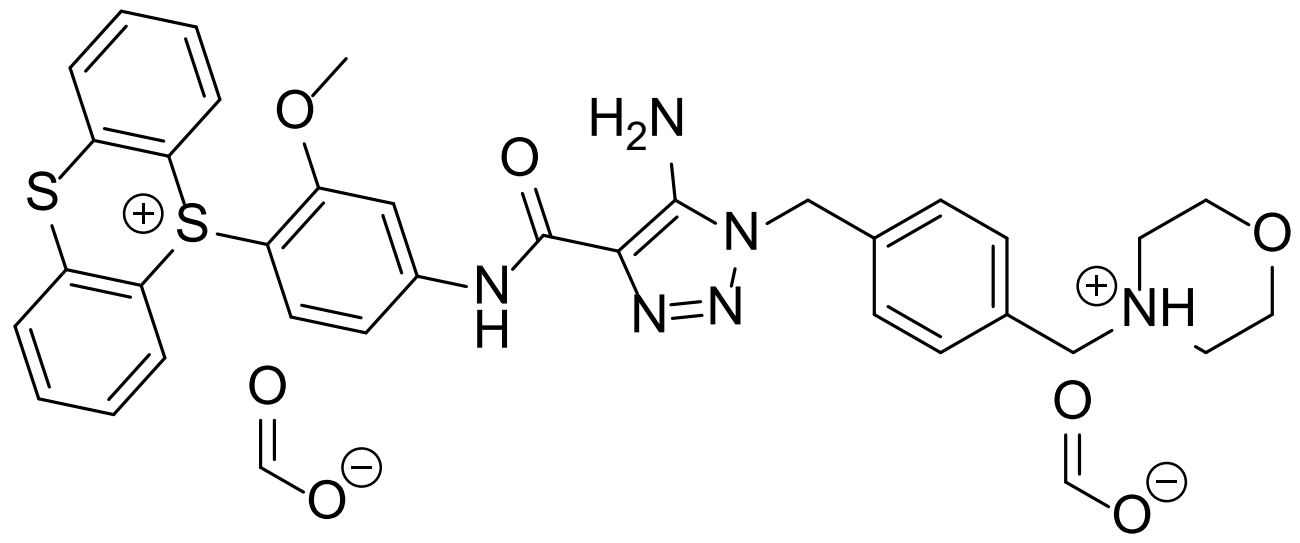

^{1}H NMR (500 MHz, MeOD, 303K)

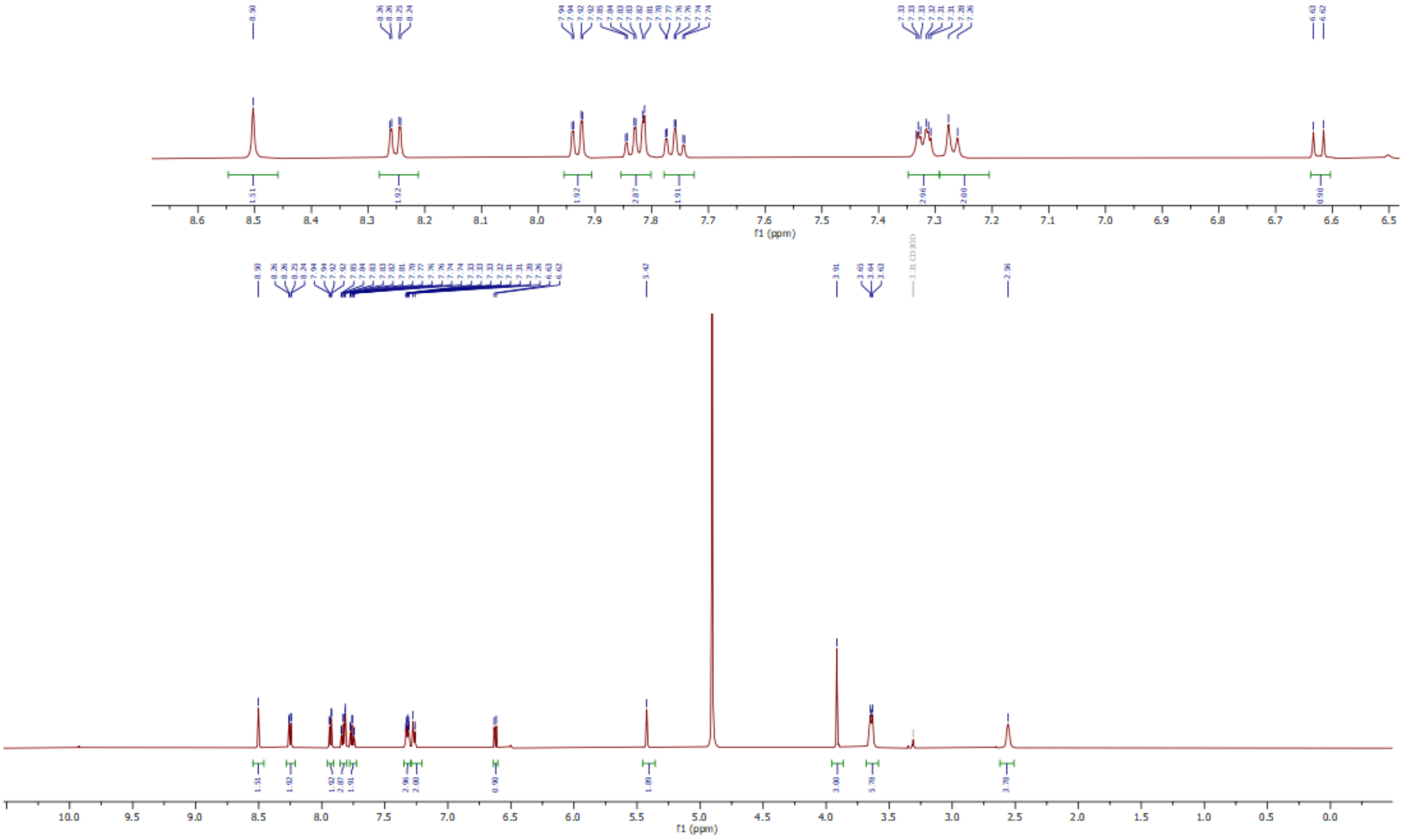

^{13}C NMR (126 MHz, MeOD, 303K)

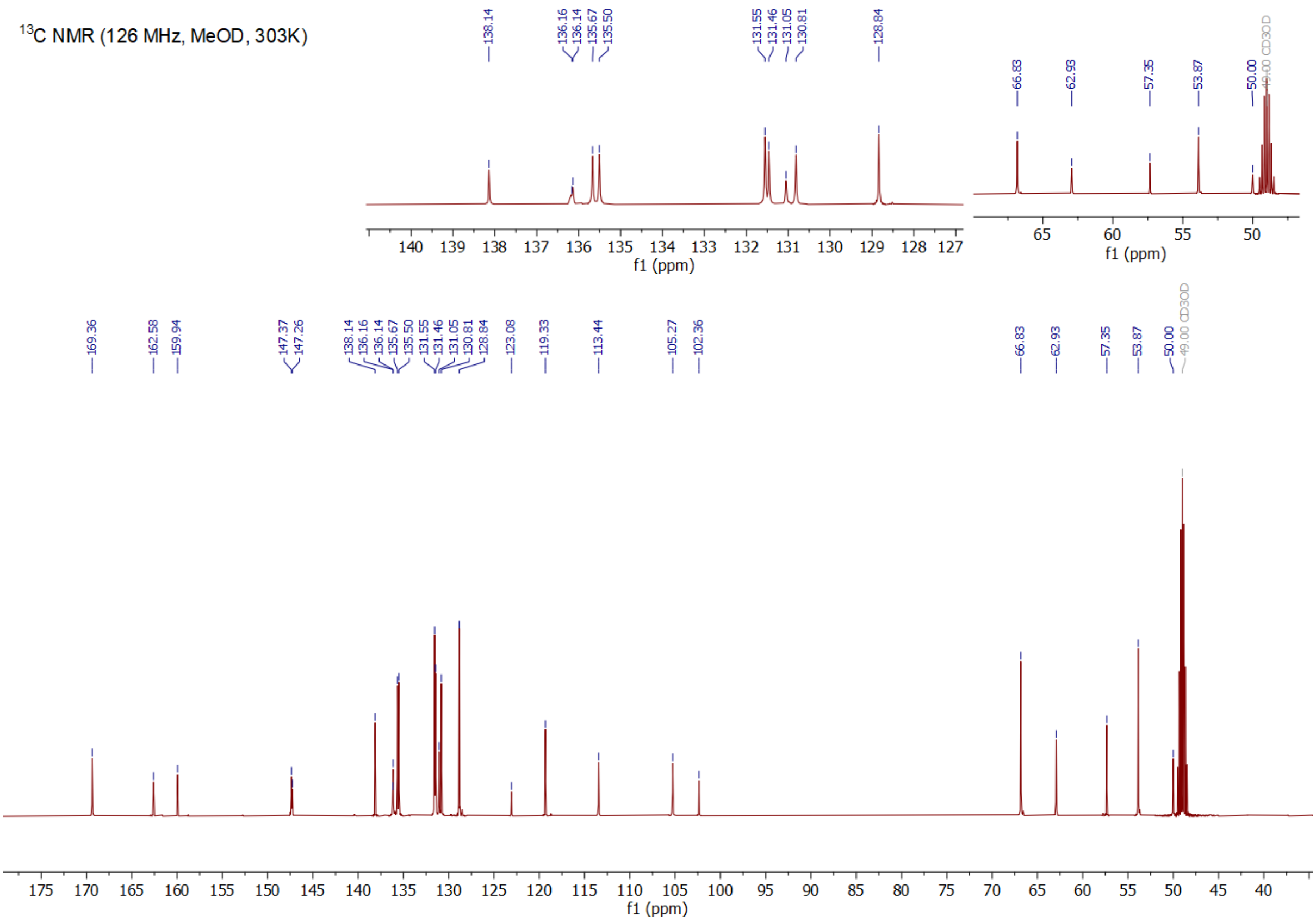

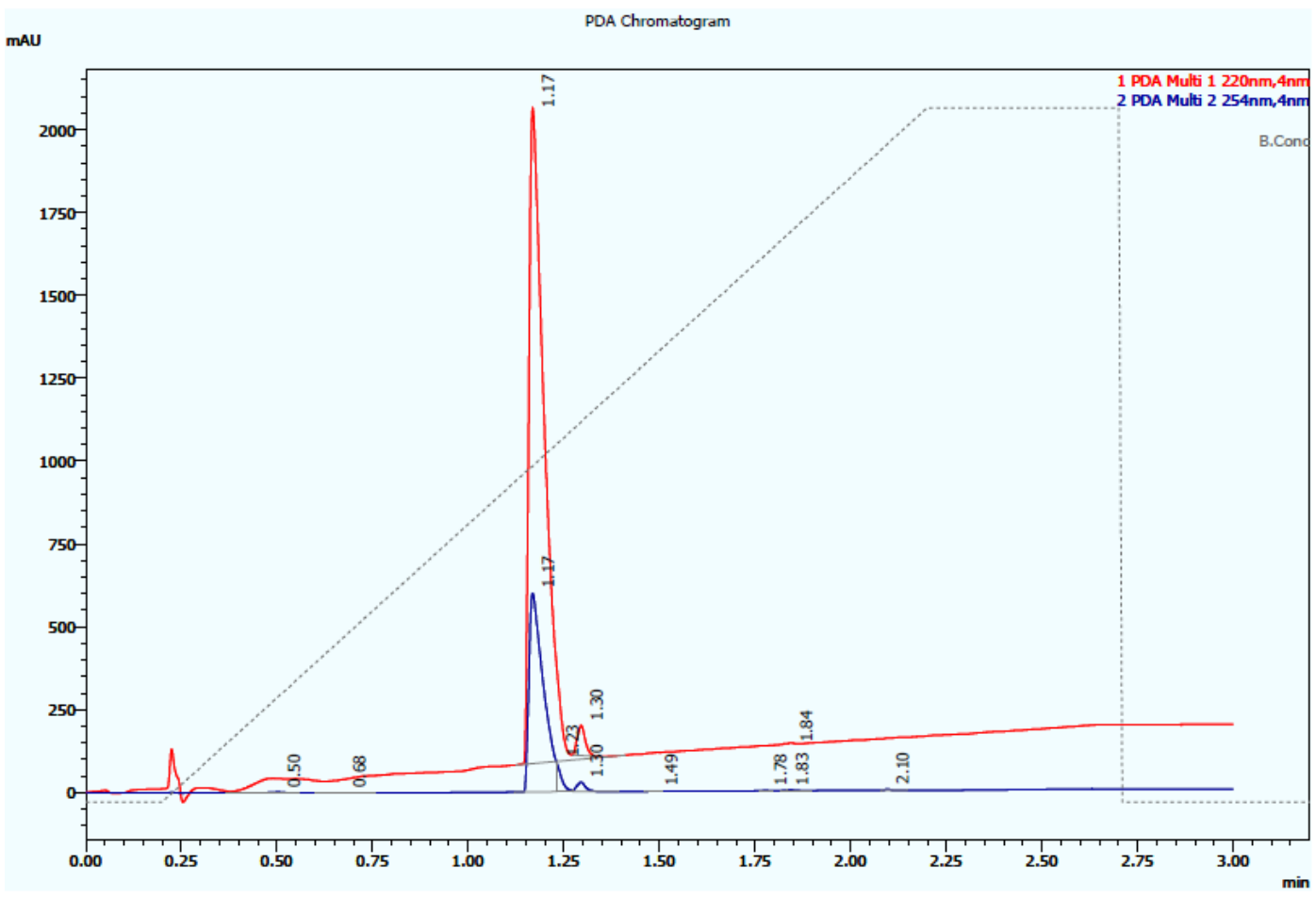

PDA Peak Table

PDA Ch1 220nm

Pk#	R.T.	Area	Height	Area%	UV max	S/N
3	1.17	5575077	1978227	97.7	217/309	50.8
5	1.30	130133	90144	2.3	218/317	2.3

PDA Peak Table

PDA Ch2 254nm

Pk#	R.T.	Area	Height	Area%	UV max	S/N
1	0.50	4679	1628	0.27	205/264/385	4.5
2	0.68	987	292	0.06	208/302	0.8
3	1.17	1586209	600481	92.00	217/309	1644.1
4	1.23	73299	87366	4.25	216/311/261	239.2
5	1.30	48130	28820	2.79	218/317	78.9
6	1.49	1315	934	0.08	219/309	2.6
7	1.78	879	1040	0.05	220	2.8
8	1.83	888	1805	0.05	220/299	4.9
9	1.84	3779	3234	0.22	220/301/378	8.9
10	2.10	4037	4309	0.23	221	11.8

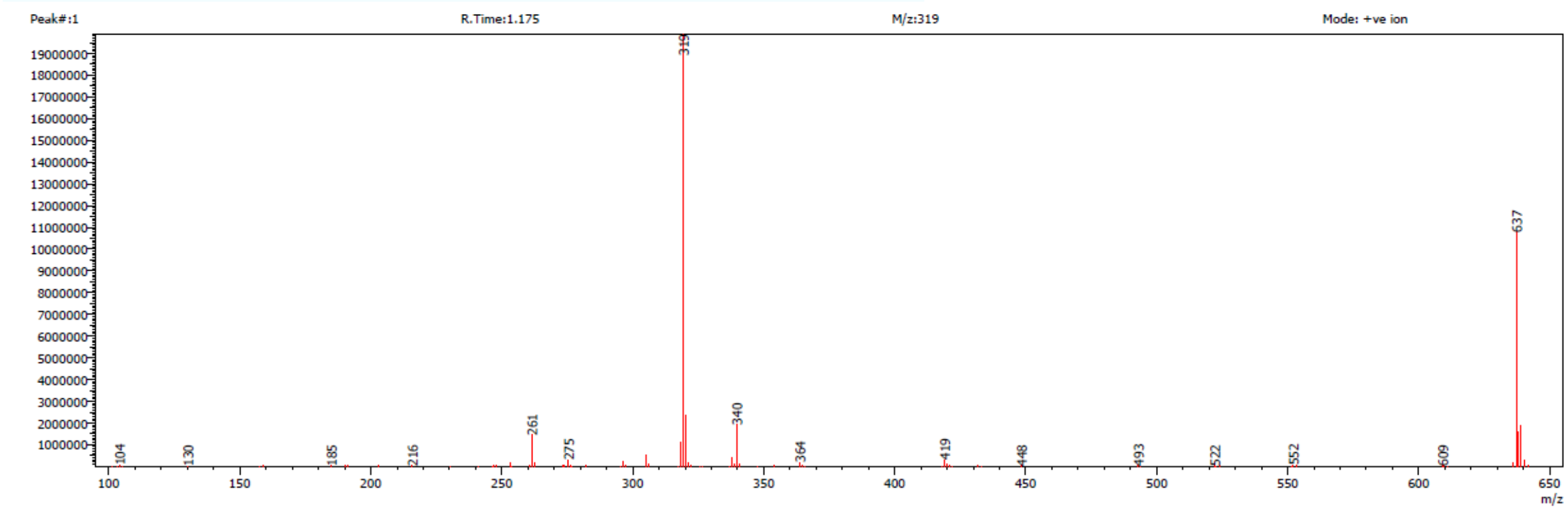

tert-butyl (2-(2-(2-((3-(4-(5-amino-1-(4-(morpholinomethyl)benzyl)-1H-1,2,3-triazole-4-carboxamido)-2-methoxyphenyl)prop-2-yn-1-yl)oxy)ethoxy)ethoxy)ethyl)carbamate (27a)

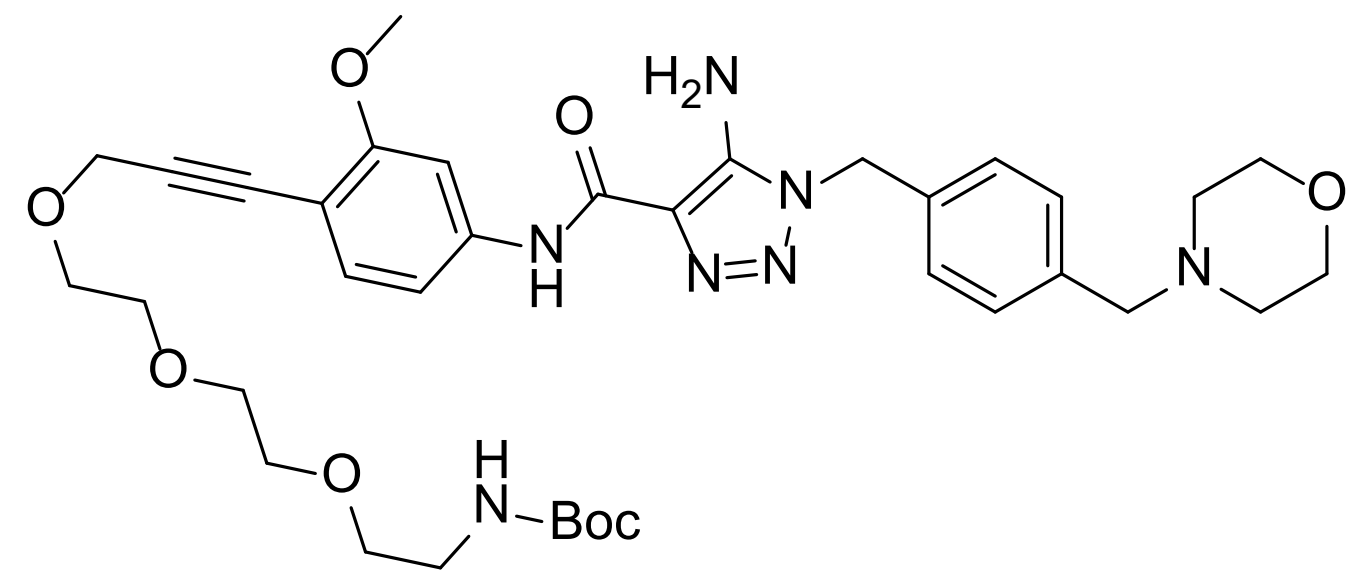

^{1}H NMR (400 MHz, MeOD, 303K)

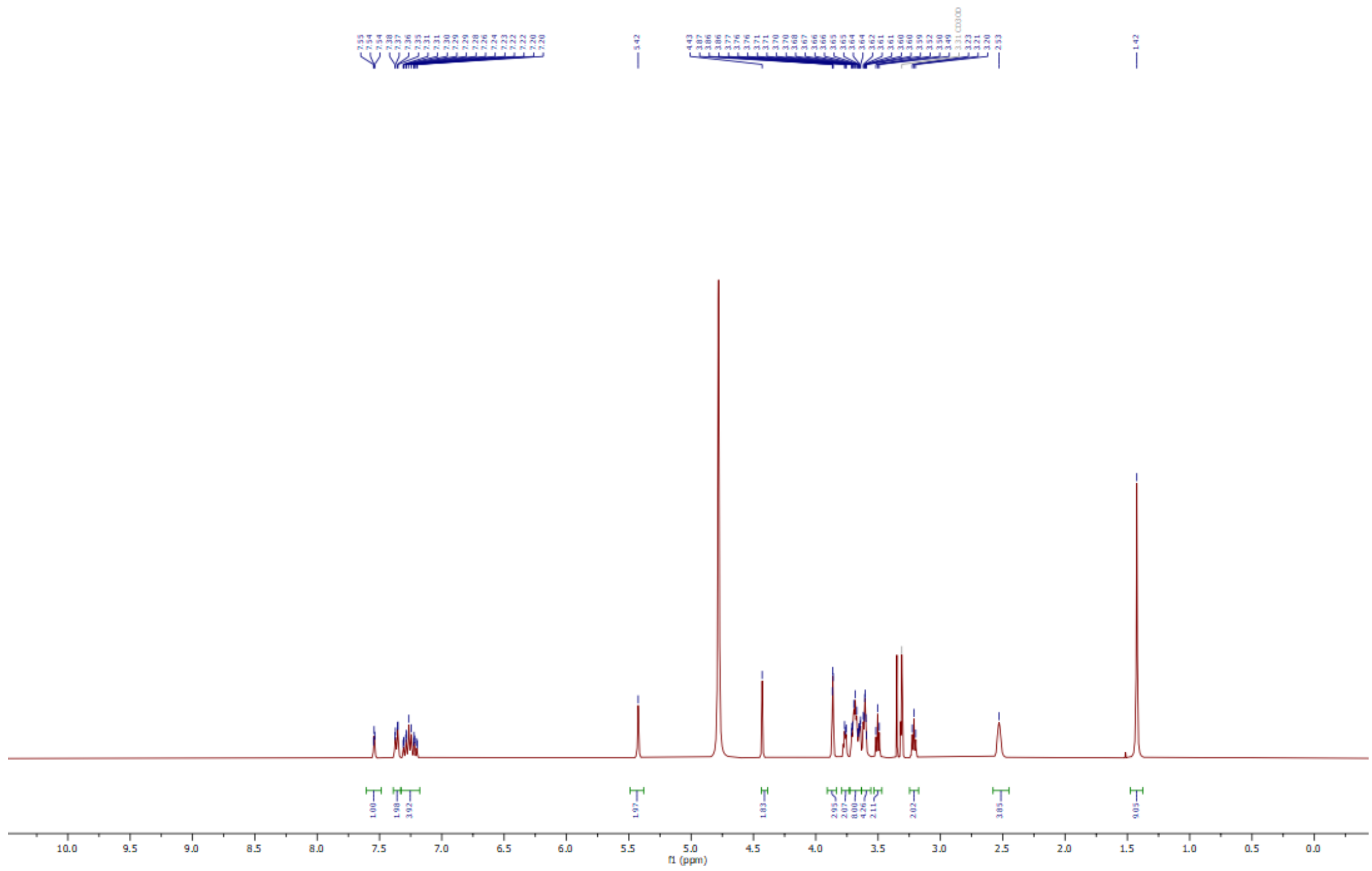

^{13}C NMR (101 MHz, MeOD, 303K)

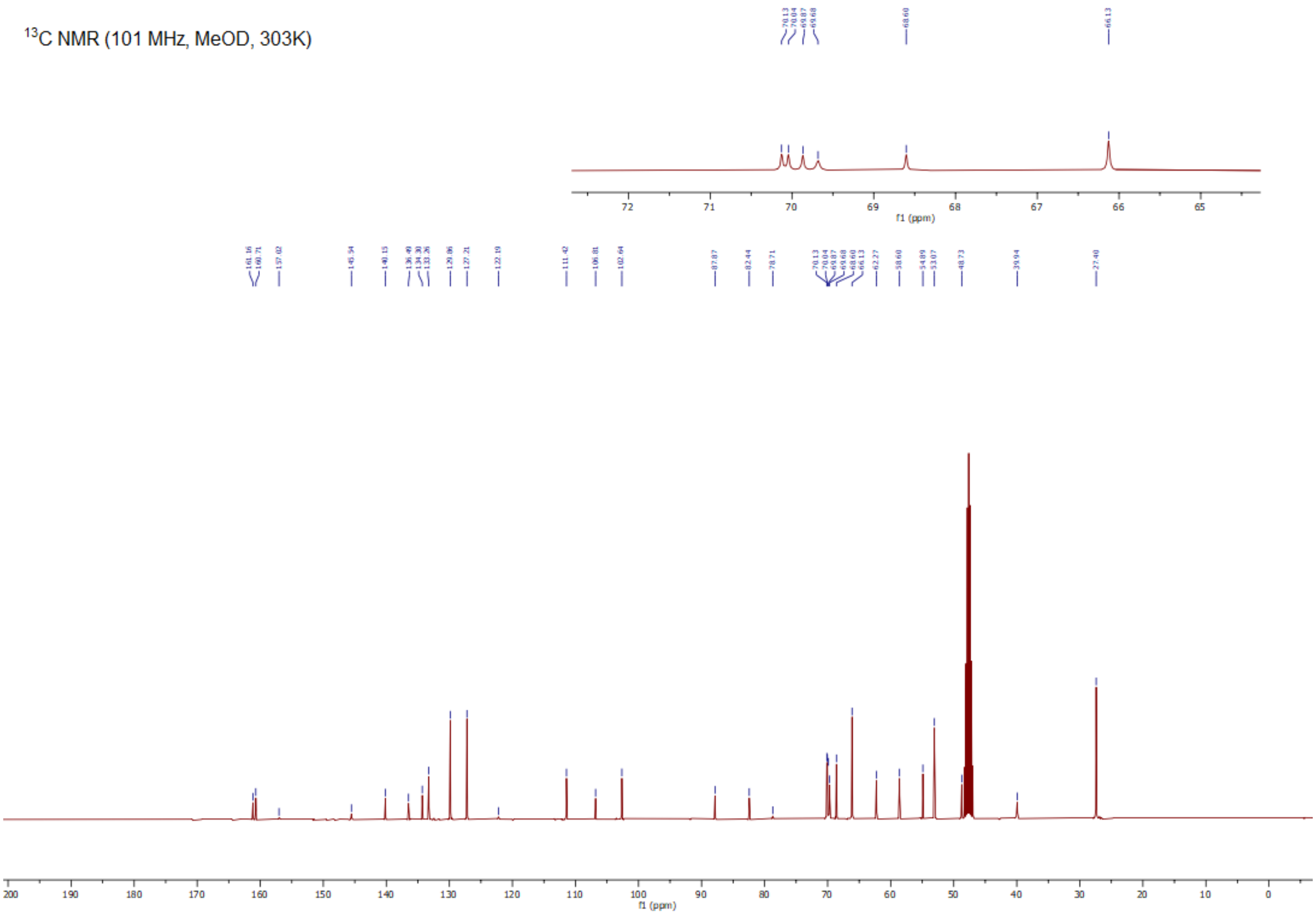

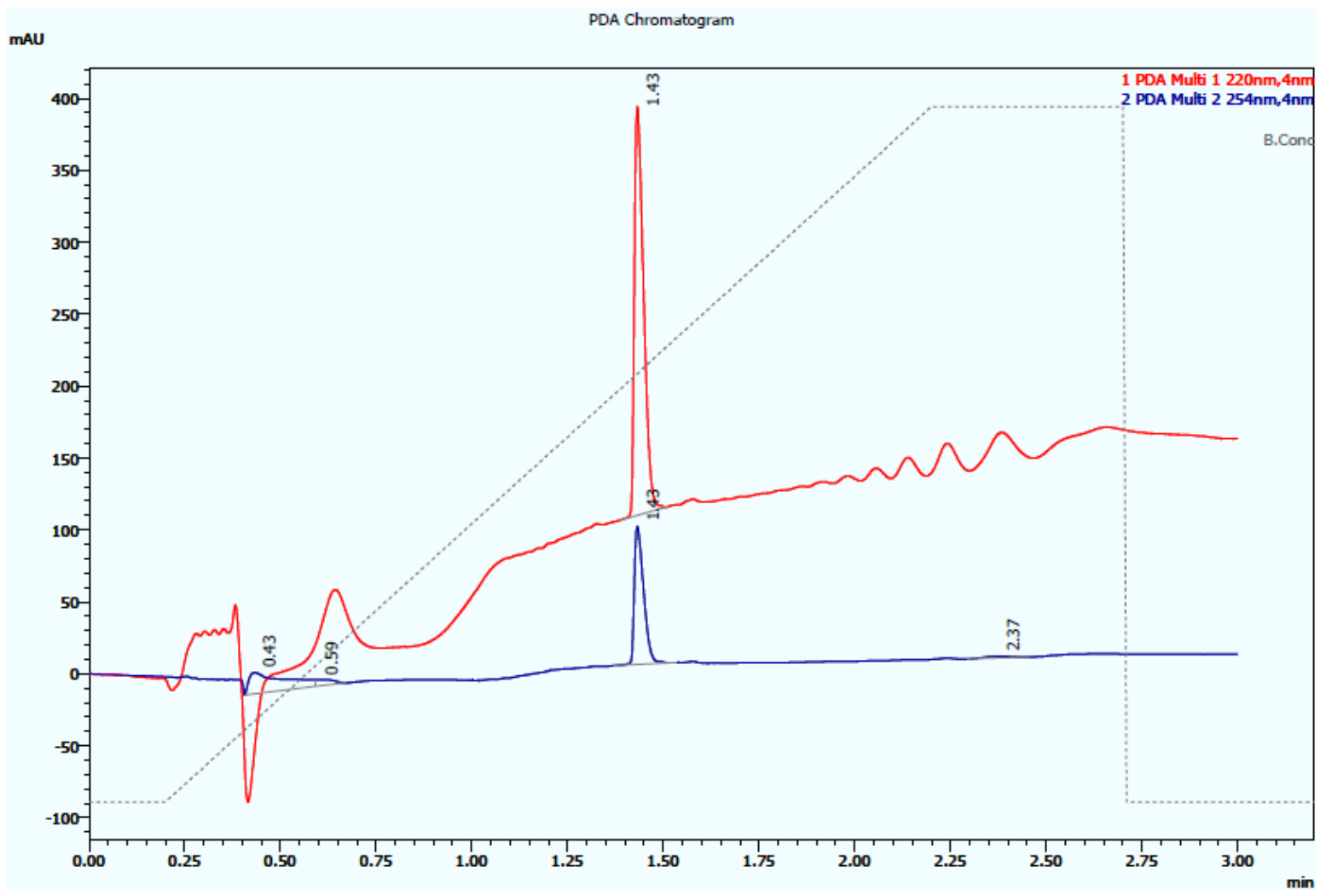

PDA Peak Table

PDA Ch1 220nm

Pk#	R.T.	Area	Height	Area%	UV max	S/N
3	1.43	476558	283916	100.0	215/311/290	5.2

PDA Peak Table

PDA Ch2 254nm

Pk#	R.T.	Area	Height	Area%	UV max	S/N
1	0.43	92692	14705	33.85	344/309/281	6.0
2	0.59	12118	4447	4.43	346/275	1.8
3	1.43	162966	95949	59.52	215/311/290	39.3
5	2.37	6020	1326	2.20	224/359/330	0.5

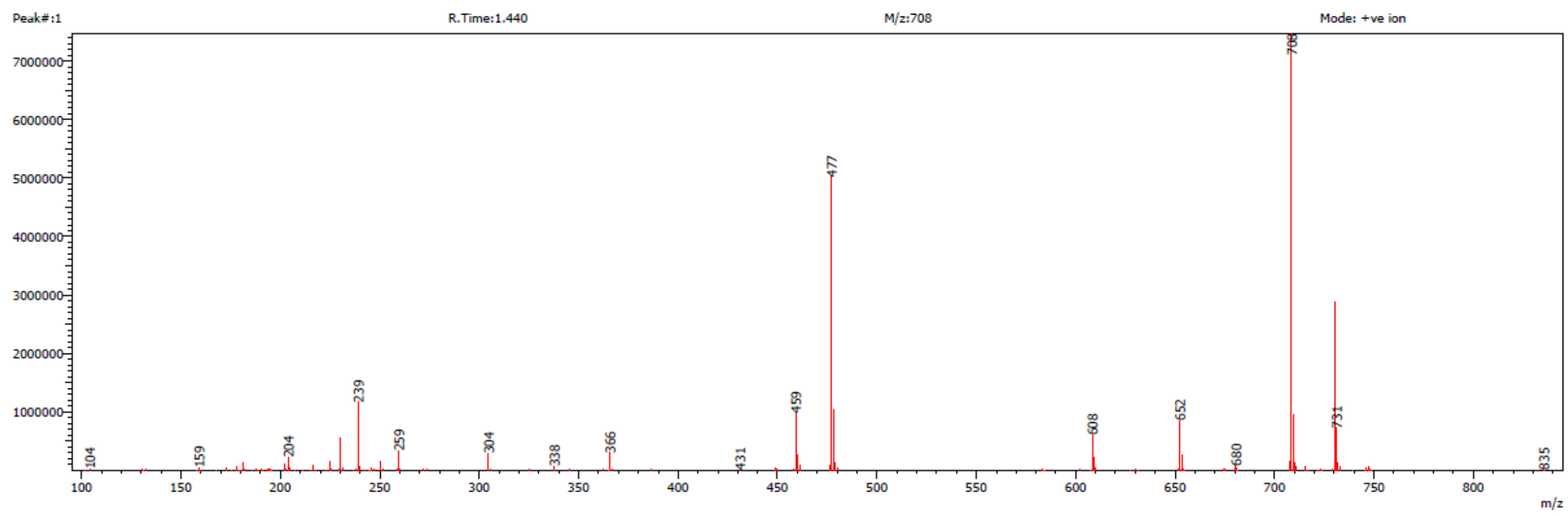

4-(4-((5-amino-4-(3-(2-methoxy-5-(5H-thianthren-5-ium-5-yl)phenyl)-1,2,4-oxadiazol-5-yl)-1H-1,2,3-triazol-1-yl)methyl)benzyl)morpholin-4-ium diformate (S3)

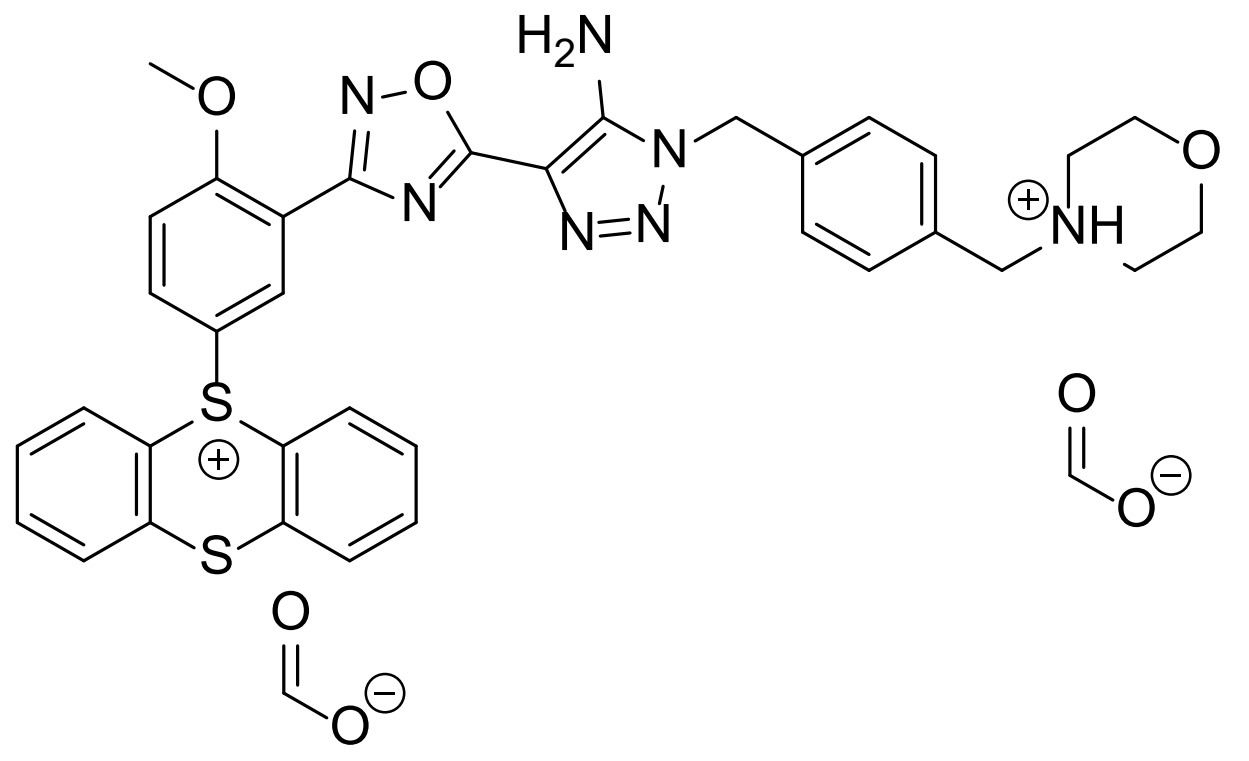

^{1}H NMR (500MHz, acetone-d_6, 303K)

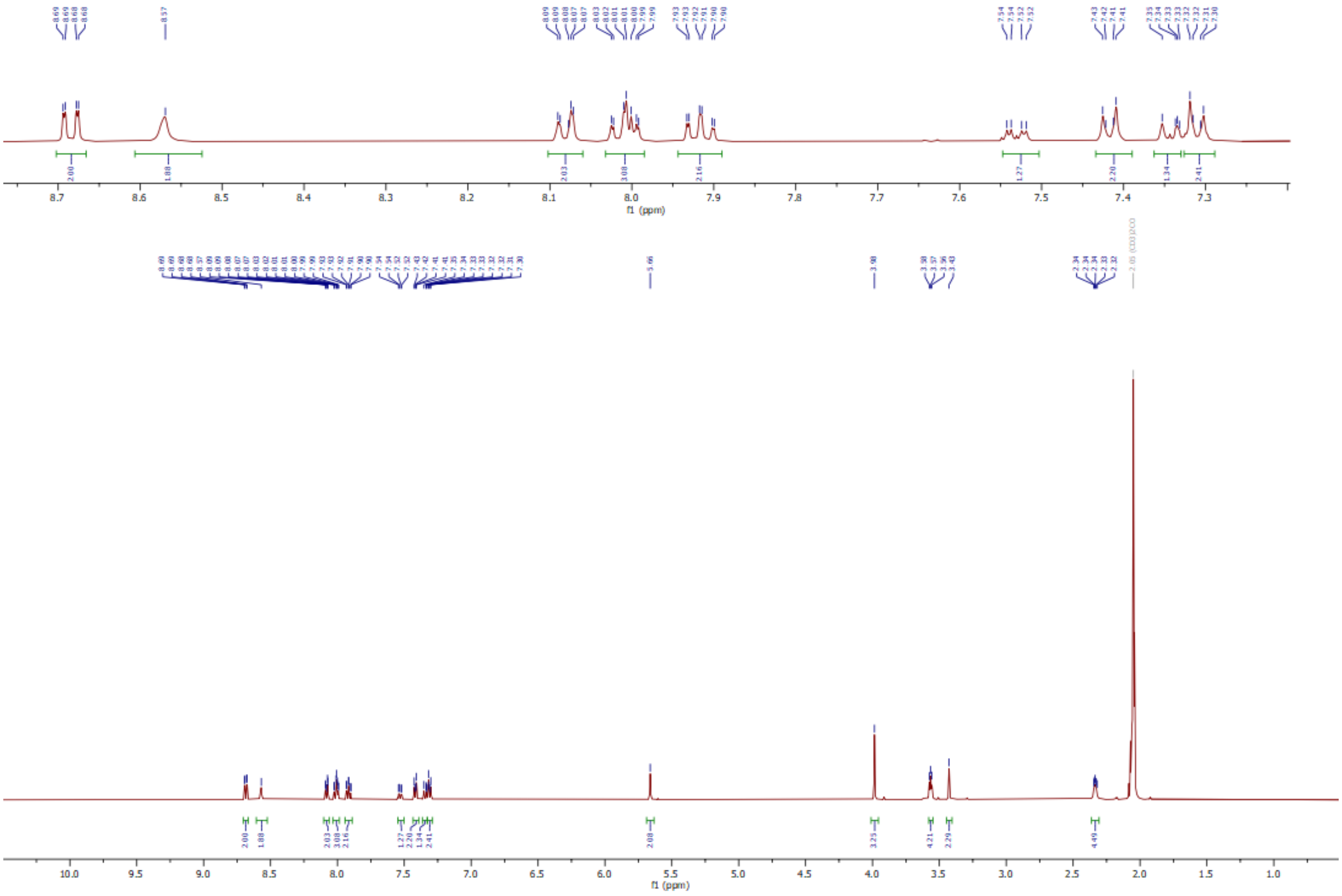

^{13}C NMR (126 MHz, acetone-d_6, 303K)

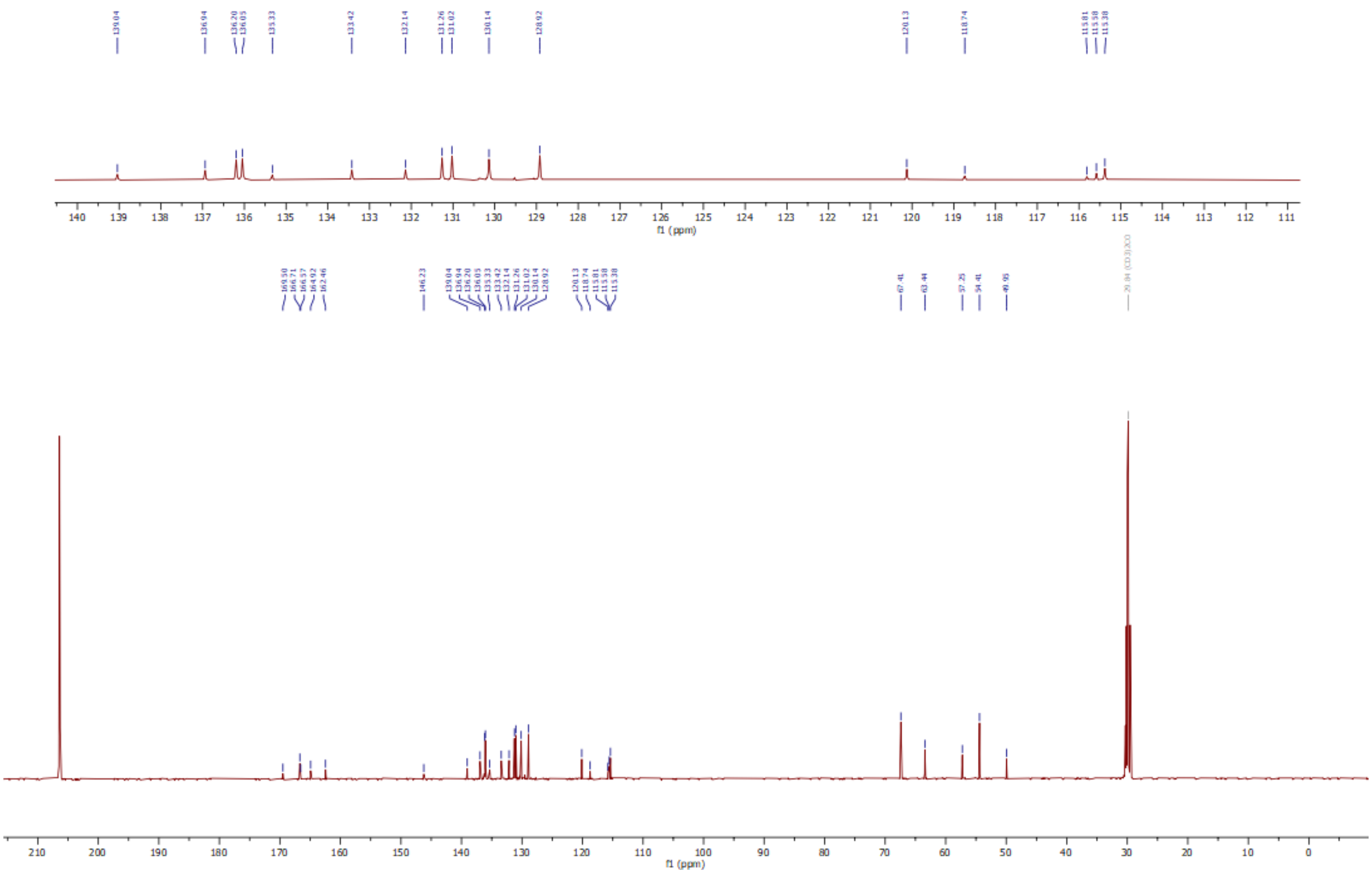

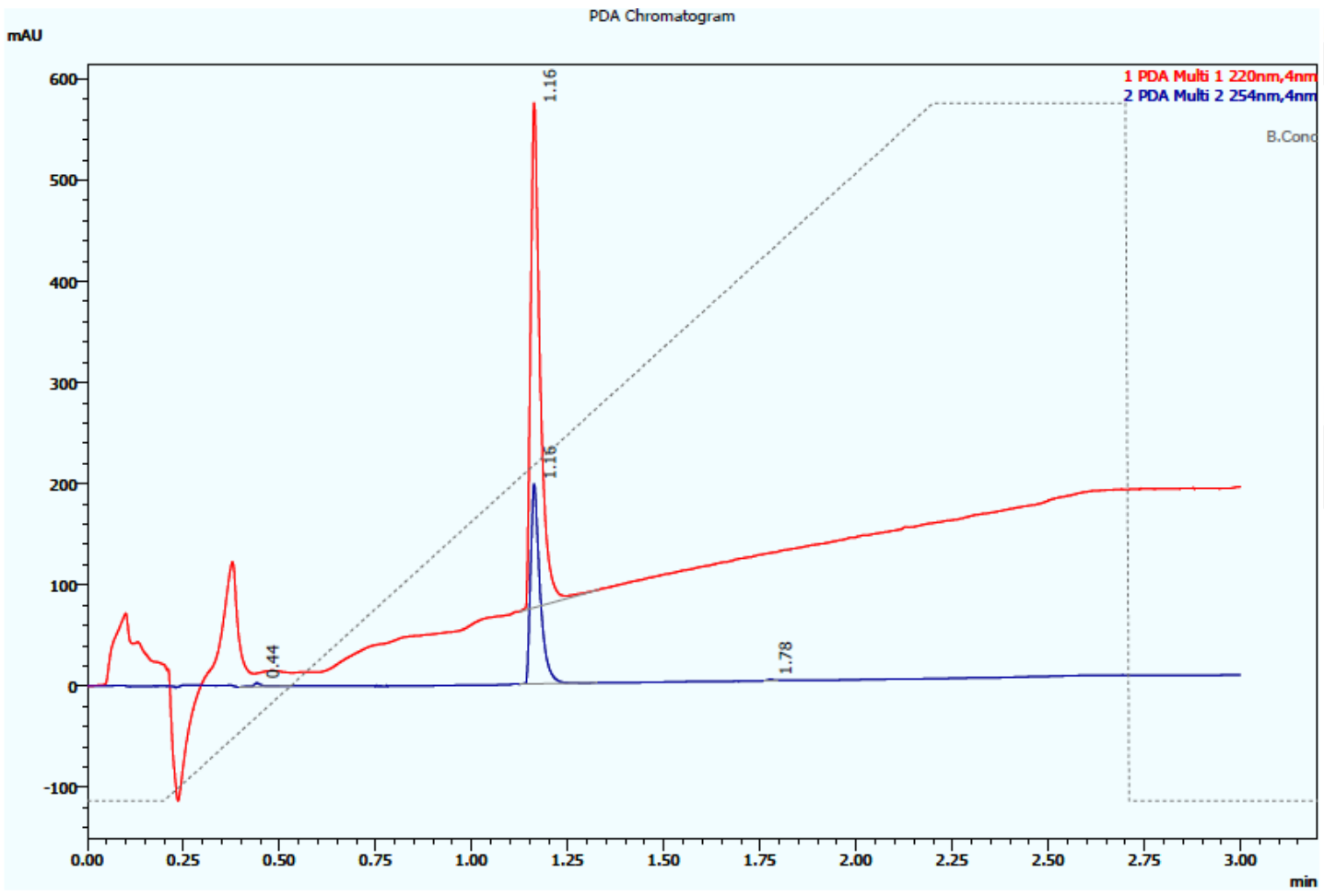

PDA Peak Table

PDA Ch1 220nm

Pk#	R.T.	Area	Height	Area%	UV max	S/N
2	1.16	859779	498651	100.0	219/293	9.6

PDA Peak Table

PDA Ch2 254nm

Pk#	R.T.	Area	Height	Area%	UV max	S/N
1	0.44	5829	3462	1.69	215/264	4.9
2	1.16	338763	197521	98.01	219/293	278.9
3	1.78	1065	1261	0.31	221	1.8

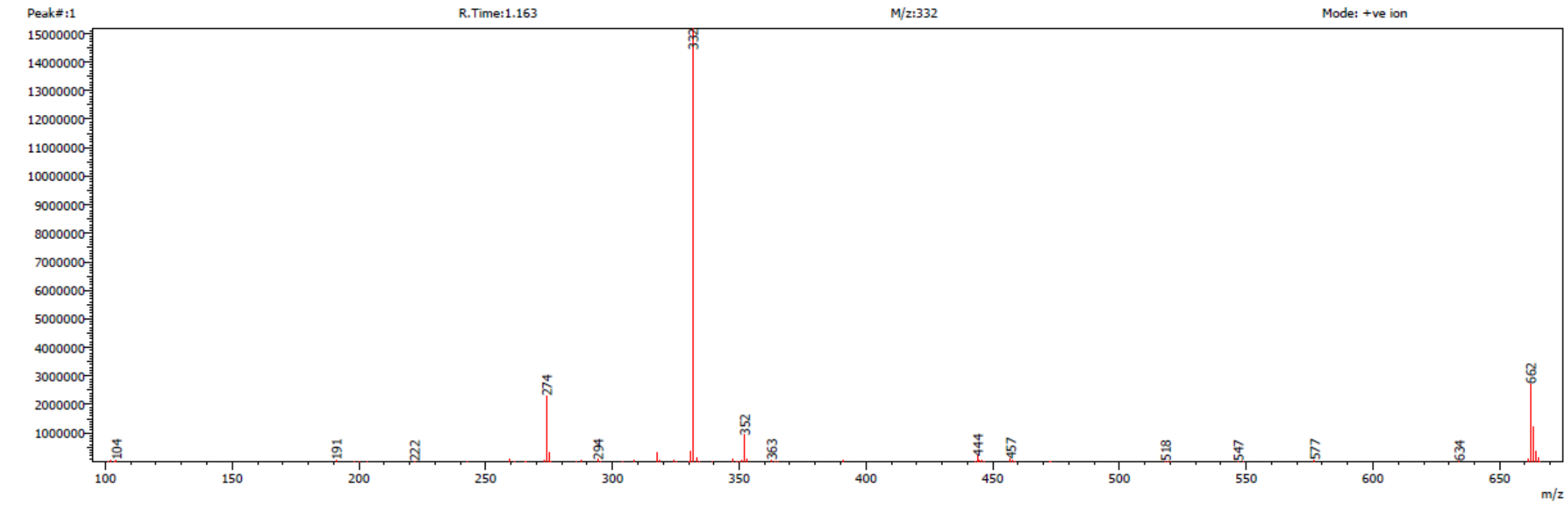

4-(4-((5-amino-4-(3-(5-(2,2-dimethyl-4-oxo-3,8,11,14-tetraoxa-5-azaheptadec-16-yn-17-yl)-2-methoxyphenyl)-1,2,4-oxadiazol-5-yl)-1H-1,2,3-triazol-1-yl)methyl)benzyl)morpholin-4-ium formate (29a)

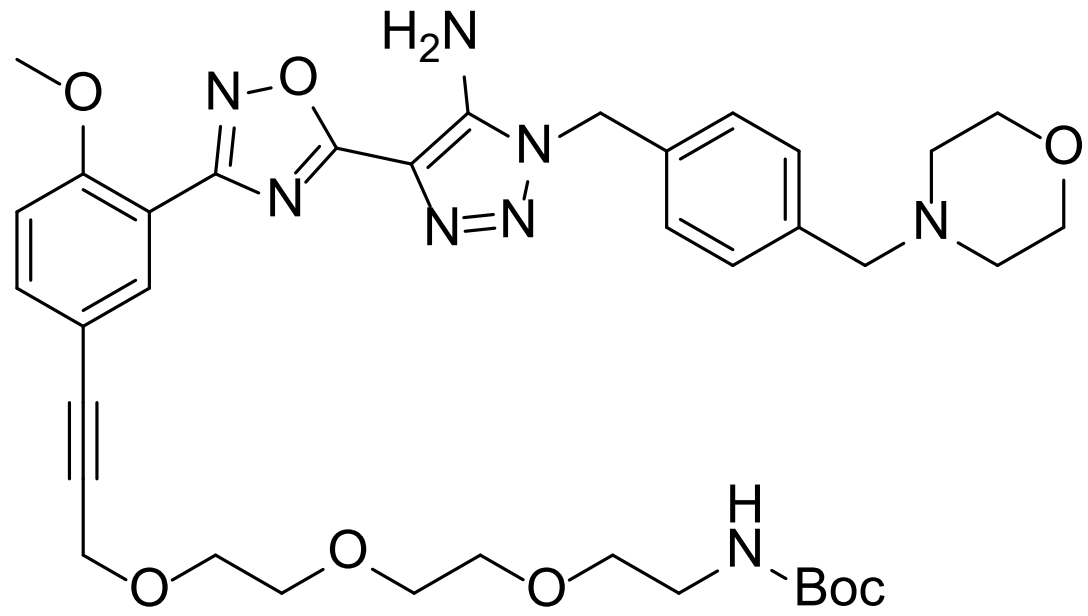

^{1}H NMR (500 MHz, $CDCl_3$, 303K)

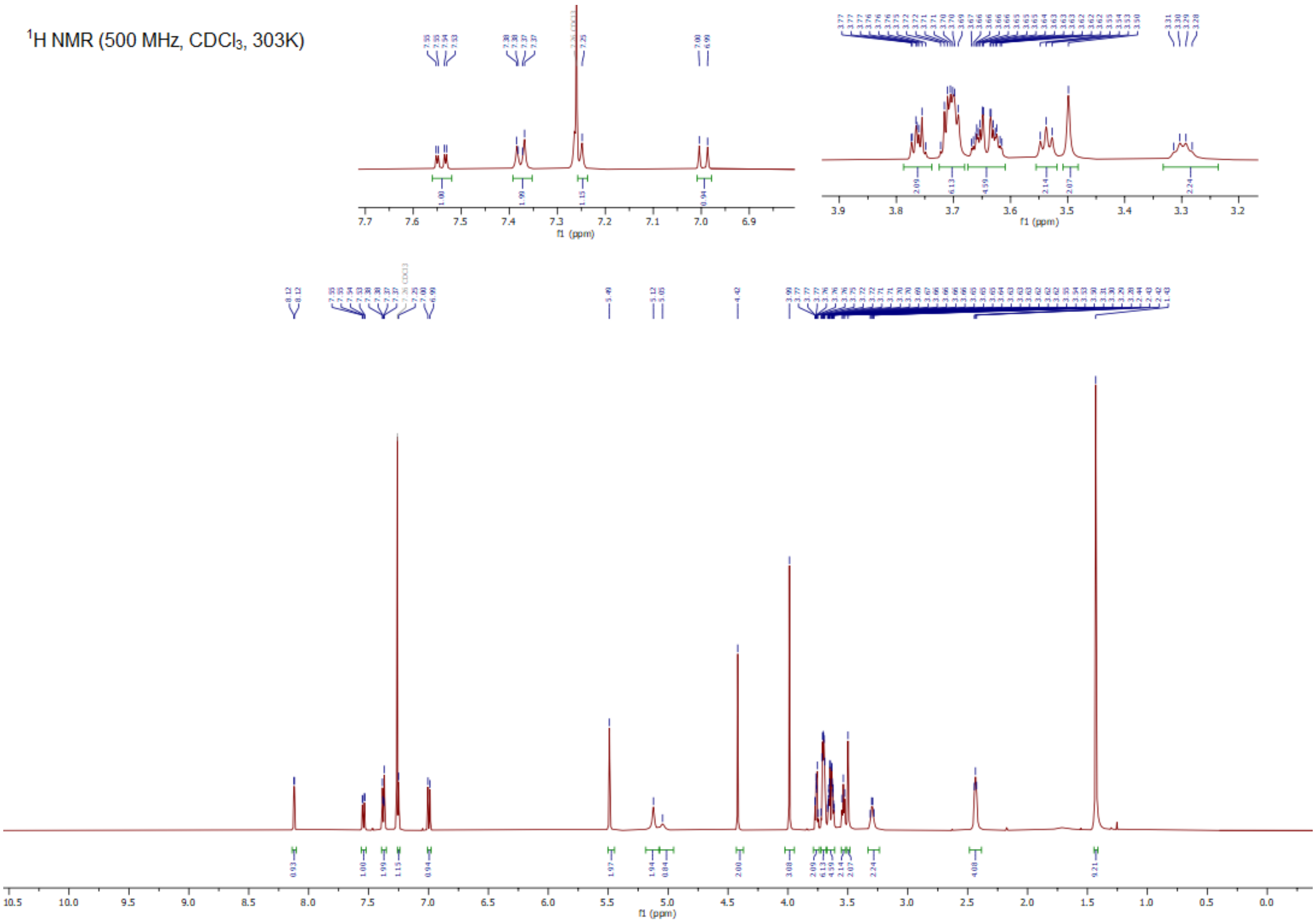

^{13}C NMR (126 MHz, CDCl$_3$, 303K)

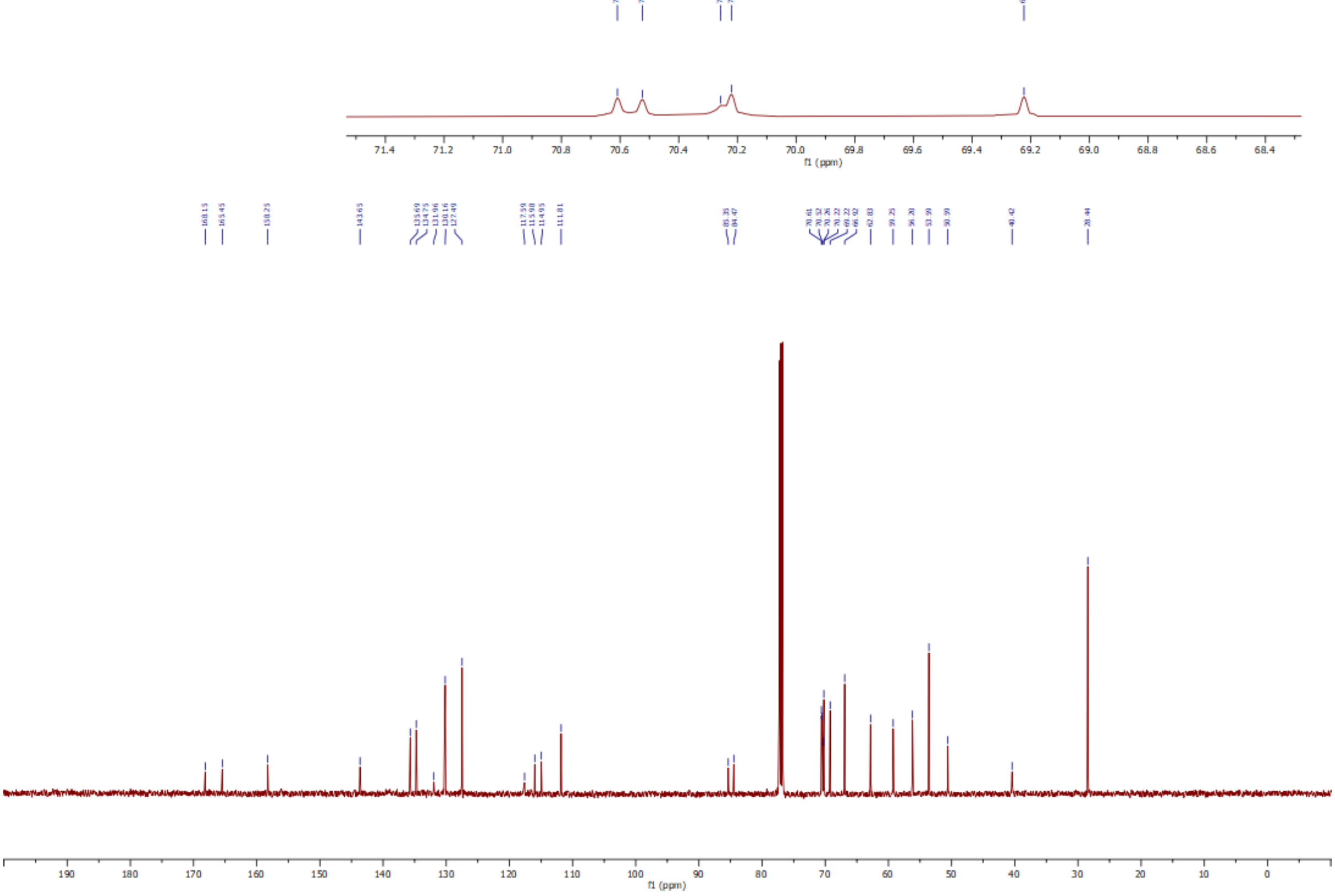

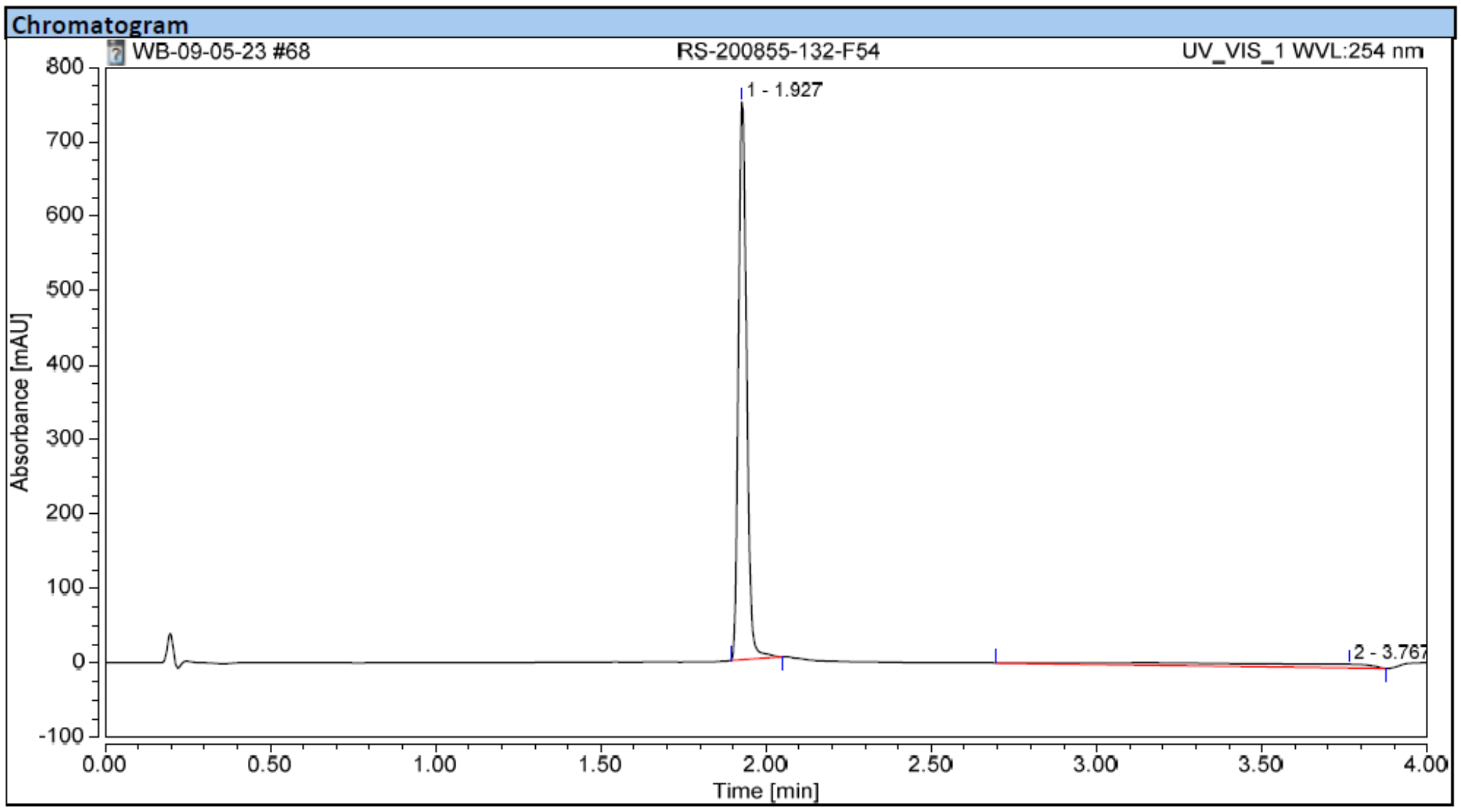

Integration Results

No.	Peak Name	Retention Time min	Area mAU*min	Height mAU	Relative Area %	Relative Height %	Amount n.a.
1		1.927	21.672	749.251	86.47	99.32	n.a.
2		3.767	3.390	5.095	13.53	0.68	n.a.
**Total:**			**25.062**	**754.346**	**100.00**	**100.00**	

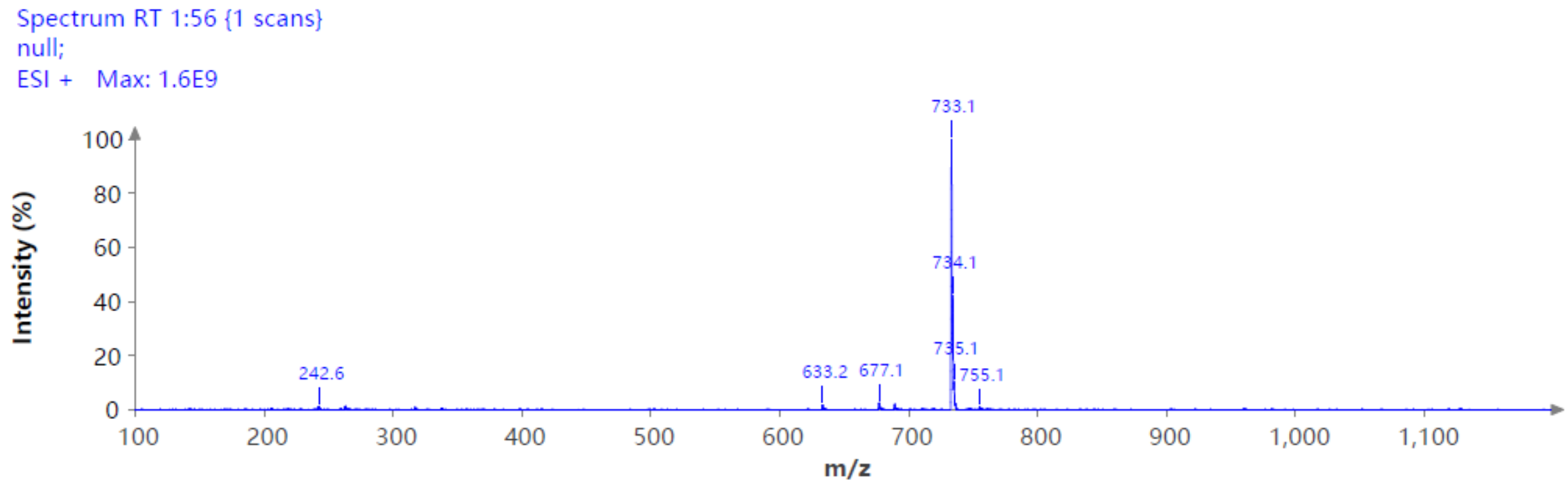
Spectrum RT 1:56 {1 scans}
null;
ESI + Max: 1.6E9
733.1
734.1
735.1
755.1
242.6
633.2
677.1
Intensity (%)
100
80
60
40
20
0
100
200
300
400
500
600
700
800
900
1,000
1,100
m/z

References

(1) Berger, F.; Plutschack, M. B.; Riegger, J.; Yu, W.; Speicher, S.; Ho, M.; Frank, N.; Ritter, T. Site-selective and versatile aromatic C−H functionalization by thianthrenation. *Nature* **2019**, *567* (7747), 223–228. DOI: https://doi.org/10.1038/s41586-019-0982-0.
(2) Woodland, A.; Grimaldi, R.; Luksch, T.; Cleghorn, L. A. T.; Ojo, K. K.; Van Voorhis, W. C.; Brenk, R.; Frearson, J. A.; Gilbert, I. H.; Wyatt, P. G. From On-Target to Off-Target Activity: Identification and Optimisation of Trypanosoma brucei GSK3 Inhibitors and Their Characterisation as Anti-Trypanosoma brucei Drug Discovery Lead Molecules. *ChemMedChem* **2013**, *8* (7), 1127–1137. DOI: https://doi.org/10.1002/cmdc.201300072.
(3) Smith, A.; Wall, R. J.; Patterson, S.; Rowan, T.; Rico Vidal, E.; Stojanovski, L.; Huggett, M.; Hampton, S. E.; Thomas, M. G.; Corpas Lopez, V.; et al. Repositioning of a Diaminothiazole Series Confirmed to Target the Cyclin-Dependent Kinase CRK12 for Use in the Treatment of African Animal Trypanosomiasis. *J. Med. Chem.* **2022**, *65* (7), 5606–5624. DOI: https://doi.org/10.1021/acs.jmedchem.1c02104.
(4) Wyllie, S.; Thomas, M.; Patterson, S.; Crouch, S.; De Rycker, M.; Lowe, R.; Gresham, S.; Urbaniak, M. D.; Otto, T. D.; Stojanovski, L.; et al. Cyclin-dependent kinase 12 is a drug target for visceral leishmaniasis. *Nature* **2018**, *560* (7717), 192–197. DOI: https://doi.org/10.1038/s41586-018-0356-z.
(5) Brand, S.; Ko, E. J.; Viayna, E.; Thompson, S.; Spinks, D.; Thomas, M.; Sandberg, L.; Francisco, A. F.; Jayawardhana, S.; Smith, V. C.; et al. Discovery and Optimization of 5-Amino-1,2,3-triazole-4-carboxamide Series against Trypanosoma cruzi. *J. Med. Chem.* **2017**, *60* (17), 7284–7299. DOI: https://doi.org/10.1021/acs.jmedchem.7b00463.
(6) Frearson, J. A.; Brand, S.; McElroy, S. P.; Cleghorn, L. A. T.; Smid, O.; Stojanovski, L.; Price, H. P.; Guther, M. L. S.; Torrie, L. S.; Robinson, D. A.; et al. N-myristoyltransferase inhibitors as new leads to treat sleeping sickness. *Nature* **2010**, *464* (7289), 728–732. DOI: https://doi.org/10.1038/nature08893.
(7) McGonagle, K.; Tarver, G. J.; Cantizani, J.; Cotillo, I.; Dodd, P. G.; Ferguson, L.; Gilbert, I. H.; Marco, M.; Miles, T.; Naylor, C.; et al. Identification and development of a series of disubstituted piperazines for the treatment of Chagas disease. *Eur. J. Med. Chem.* **2022**, *238*, 114421. DOI: https://doi.org/10.1016/j.ejmech.2022.114421.
(8) Smith, V. C.; Cleghorn, L. A. T.; Woodland, A.; Spinks, D.; Hallyburton, I.; Collie, I. T.; Yi Mok, N.; Norval, S.; Brenk, R.; Fairlamb, A. H.; et al. Optimisation of the Anti-Trypanosoma brucei Activity of the Opioid Agonist U50488. *ChemMedChem* **2011**, *6* (10), 1832–1840. DOI: https://doi.org/10.1002/cmdc.201100278.
(9) Cleghorn, L. A. T.; Albrecht, S.; Stojanovski, L.; Simeons, F. R. J.; Norval, S.; Kime, R.; Collie, I. T.; De Rycker, M.; Campbell, L.; Hallyburton, I.; et al. Discovery of Indoline-2-carboxamide Derivatives as a New Class of Brain-Penetrant Inhibitors of Trypanosoma brucei. *J. Med. Chem.* **2015**, *58* (19), 7695–7706. DOI: https://doi.org/10.1021/acs.jmedchem.5b00596.
(10) Smith, R. J.; Milne, R.; Lopez, V. C.; Wiedemar, N.; Dey, G.; Syed, A. J.; Patterson, S.; Wyllie, S. Chemical pulldown combined with mass spectrometry to identify the molecular targets of antimalarials in cell-free lysates. *STAR Protocols* **2023**, *4* (1), 102002. DOI: https://doi.org/10.1016/j.xpro.2022.102002.
(11) Goyard, S.; Segawa, H.; Gordon, J.; Showalter, M.; Duncan, R.; Turco, S. J.; Beverley, S. M. An in vitro system for developmental and genetic studies of Leishmania donovani phosphoglycans. *Mol Biochem Parasitol* **2003**, *130* (1), 31–42. DOI: https://doi.org/10.1016/s0166-6851(03)00142-7.
(12) Paradela, L. S.; Wall, R. J.; Carvalho, S.; Chemi, G.; Corpas-Lopez, V.; Moynihan, E.; Bello, D.; Patterson, S.; Güther, M. L. S.; Fairlamb, A. H.; et al. Multiple unbiased approaches identify oxidosqualene cyclase as the molecular target of a promising anti-leishmanial. *Cell Chem Biol* **2021**, *28* (5), 711–721.e718. DOI: https://doi.org/10.1016/j.chembiol.2021.02.008.
(13) HaileMariam, M.; Eguez, R. V.; Singh, H.; Bekele, S.; Ameni, G.; Pieper, R.; Yu, Y. S-Trap, an Ultrafast Sample-Preparation Approach for Shotgun Proteomics. *J. Proteome Res.* **2018**, *17* (9), 2917–2924. DOI: https://doi.org/10.1021/acs.jproteome.8b00505.

(14) Perez-Riverol, Y.; Bai, J.; Bandla, C.; García-Seisdedos, D.; Hewapathirana, S.; Kamatchinathan, S.; Kundu, Deepti J.; Prakash, A.; Frericks-Zipper, A.; Eisenacher, M.; et al. The PRIDE database resources in 2022: a hub for mass spectrometry-based proteomics evidences. *Nucleic Acids Res.* **2021**, *50* (D1), D543–D552. DOI: https://doi.org/10.1093/nar/gkab1038 (accessed 3/10/2026).
